# Integration of miRNA and gene expression profiles suggest a role for miRNAs in the pathobiological processes of acute *Trypanosoma cruzi* infection

**DOI:** 10.1038/s41598-017-18080-9

**Published:** 2017-12-21

**Authors:** Ludmila Rodrigues Pinto Ferreira, Frederico Moraes Ferreira, Laurie Laugier, Sandrine Cabantous, Isabela Cunha Navarro, Darlan da Silva Cândido, Vagner Carvalho Rigaud, Juliana Monte Real, Glaucia Vilar Pereira, Isabela Resende Pereira, Leonardo Ruivo, Ramendra Pati Pandey, Marilda Savoia, Jorge Kalil, Joseli Lannes-Vieira, Helder Nakaya, Christophe Chevillard, Edecio Cunha-Neto

**Affiliations:** 1 Laboratory of Immunology, Heart Institute (InCor), University of São Paulo School of Medicine, São Paulo, Brazil; 2 Division of Clinical Immunology and Allergy, University of São Paulo School of Medicine, São Paulo, Brazil; 3 Institute for Investigation in Immunology, iii-INCT, São Paulo, Brazil; 4 Departamento Morfologia, Instituto de Ciências Biológicas, Universidade Federal de Minas Gerais, Belo Horizonte, MG. Brazil; 5 INSERM, Aix-Marseille University AMU, Faculté de Médecine, Marseille, U1108 France; 6 TUCCA Association for Children and Adolescents with Cancer, Department of Pediatric Oncology, Santa Marcelina Hospital, São Paulo, Brazil; 7 Centro de Investigação Translacional em Oncologia, Instituto do Câncer do Estado de São Paulo, Universidade de São Paulo, São Paulo, Brazil; 8 Laboratory of Biology of Interactions, Oswaldo Cruz Institute – FIOCRUZ, Rio de Janeiro, Brazil; 9 Department of Pathophysiology and Toxicology, School of Pharmaceutical Sciences, University of São Paulo, São Paulo, 077010 Brazil; 10 Department of Pathology, Emory University School of Medicine, Atlanta, GA 30322 USA; 11 INSERM, Aix-Marseille University AMU, UMR1090, Parc Scientifique de Luminy case 928, 163 avenue de Luminy, Marseille, France

## Abstract

Chagas disease, caused by the parasite *Trypanosoma cruzi*, is endemic in Latin America. Its acute phase is associated with high parasitism, myocarditis and profound myocardial gene expression changes. A chronic phase ensues where 30% develop severe heart lesions. Mouse models of *T. cruzi* infection have been used to study heart damage in Chagas disease. The aim of this study was to provide an interactome between miRNAs and their targetome in Chagas heart disease by integrating gene and microRNA expression profiling data from hearts of *T. cruzi* infected mice. Gene expression profiling revealed enrichment in biological processes and pathways associated with immune response and metabolism. Pathways, functional and upstream regulator analysis of the intersections between predicted targets of differentially expressed microRNAs and differentially expressed mRNAs revealed enrichment in biological processes and pathways such as IFNγ, TNFα, NF-kB signaling signatures, CTL-mediated apoptosis, mitochondrial dysfunction, and Nrf2-modulated antioxidative responses. We also observed enrichment in other key heart disease-related processes like myocarditis, fibrosis, hypertrophy and arrhythmia. Our correlation study suggests that miRNAs may be implicated in the pathophysiological processes taking place the hearts of acutely *T. cruzi-*infected mice.

## Introduction

Chagas disease (American trypanosomiasis) is a deadly disease caused by an intracellular parasite called *Trypanosoma cruzi* which can be transmitted to the human host by blood-feeding triatomine insects in endemic areas from Argentina to Mexico^1,2^. The parasite can be also transmitted by blood transfusion, organ transplantation, congenitally, and by accidental ingestion. About 7 million people are infected in endemic areas^3^, and an estimated 700,000 infected people live Northern Hemisphere countries^2–4^, which turned Chagas disease into a global health proble^3–5^. The acute phase of infection in humans is mostly asymptomatic, but acute infection with high parasite numbers induce myocarditis and severe disease. Upon infection, *T. cruzi* invades the bloodstream^6^, infecting many cell types in different tissues including cardiac tissue. *T. cruzi* components trigger innate immunity^7^ causing intense inflammation that is partially counteracted by IL-10^7–10^. The potent innate and adaptive antibody and IFNγ-producing T cell response keeps parasitism in check, establishing a low-grade chronic infection. Most chronically infected patients (60%) remain healthy for life (the indeterminate/asymptomatic form)^11,12^. However, about 30% of chronically infected patients develop a life-threatening, symptomatic cardiac disease with inflammatory dilated cardiomyopathy up to 30 years after infection, Chronic Chagas disease Cardiomyopathy (CCC)^13–15^. A smaller proportion of Chagas disease patients can develop digestive system dilation. CCC is the major cause of death due to Chagas disease. Clinicopathological manifestations of CCC include myocarditis, fibrosis, hypertrophy, electrocardiogram abnormalities and arrhythmias^16–18^. Its pathogenesis is still a matter of debate. The Th1 T cell-rich myocardial inflammatory infiltrate with intense IFNγ production^19,20^ is correlated with disease progression and is considered one of the key factors initiating and perpetuating heart damage^21^. The susceptibility factors that lead to 30% of individuals to develop CCC after *T. cruzi* infection remain unknown, and evidence indicates a genetic component linked to immune-related gene polymorphisms in susceptibility to CCC development^22^. Gene expression profiling of myocardium from CCC patients^23^, as well as hearts of acutely and chronically *T. cruzi*- infected mice^24–26^ have shown a profound change in gene expression patterns. Differentially expressed genes included immune-related genes, energy metabolism/mitochondrial genes and cell stress/oxidative stress response genes, which are all important pathogenic factors in Chagas disease cardiomyopathy. Significantly, an IFNγ transcriptional signature was observed in CCC heart tissue, and we showed that IFNγ could directly induce expression of hypertrophy-related genes in cardiomyocytes. MicroRNAs (miRNAs or miRs) are noncoding single stranded RNA molecules (18–26 nt length) capable of silencing gene expression, by inhibiting protein translation or by inducing mRNA degradation after binding to complementary sequences at the 3′ untranslated region^27^. They play a key role in “fine tuning” gene expression in multiple physiological and pathological processes including the cardiovascular system^28–32^. Our group has observed dysregulated miRNA expression in heart samples from CCC patients^21^ and acute *T. cruzi* infection in mice^33^ including miR-133 and miR-208, which regulate heart genes related to cardiovascular disease^34–39^. In the mouse study, we found 113 differentially expressed miRs (DEMs) at 15, 30 and/or 45 days post infection. Several of the DEMs were significantly correlated with the clinically relevant parameters parasitemia and electrocardiography changes (QTc interval)^33^. Although those results were suggestive of a role for miRNA in gene regulation and disease parameters, the relevance of miRNA control of the overall gene expression in heart of infected mice was still unknown. In order to assess the role of miRNAs in the regulation of the transcriptional changes that occur during acute *T. cruzi* infection, we have performed an integrated genome-wide analysis of genes and miRNA expression changes in the hearts of acutely infected mice. To this end, we performed mRNA expression analysis and used sequence-based miRNA target prediction and negative correlation of differentially expressed miRNA and mRNA in *T. cruzi*-infected heart tissue to identify regulated pathways. We also performed pathways, functional enrichment and upstream regulator analysis of differentially expressed genes (DEGs) targeted by differentially expressed miRNAs (DEMs). Finally, DEMs-DEGs networks were built around the main pathophysiological parameters of Chagas heart disease.

## Results

### Identification of DEGs and gene set enrichment analysis reveals functional pathways modulated in hearts of acutely *T. cruzi* infected mice

**Figure 1:**
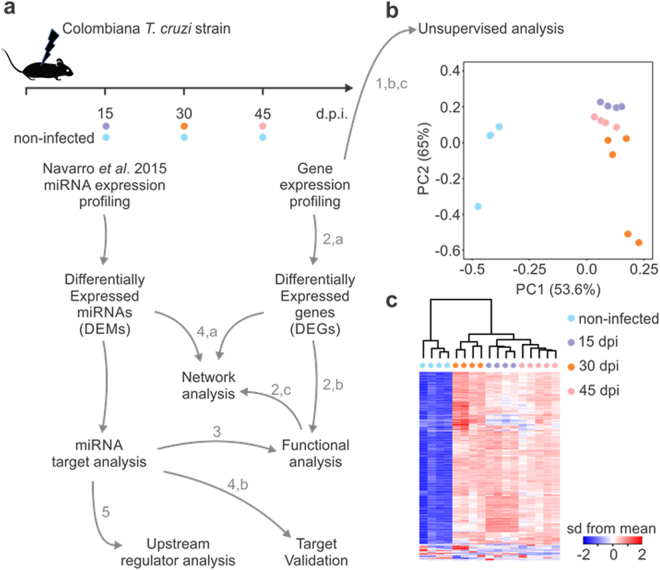
(**a**) Workflow of data processing and analysis used to identify DEMS-DEGs interactome: Mice were infected with 100 blood trypomastigotes of the Colombian strain of *T*. *cruzi* and the heart gene expression profiling was evaluated 15, 30 or 45 days after infection. The miRNA profile used in this study was from the same samples and previous published (Navarro *et al*. 2015). The numbers and letters in the workflow indicate each one of the analysis figures present in this paper. Gene expression, statistical analysis and identification of differentially expressed genes (DEG) was performed using Linear Model for microarray data (Limma) with adjustment for false discovery rate with the Benjamini-Hochberg method. (**b**) Principal component analysis (PCA) of gene expression was performed for all samples and all probe set, by using a median centering of the data set. The x-axis corresponds to principal component 1 (PC1) and the y-axis to the principal component 2 (PC2), the percentage of the variance is indicated between brackets. Based on their mRNA expression values, samples from each time group (non-infected, 15, 30 and 45 dpi) clustered together, thus confirming homogeneity of the gene expression profiles within each group. The group of infected samples clustered independently from the non-infected group. (**c**) Heatmap and hierarchical clustering. The clustering is performed on all the samples and all probe set, using squared Euclidean distance measure and Ward’s method for linkage analysis and Z score normalization. Each column represents one sample and each row one mRNA. The color-coded scale (blue: expression levels lower than the mean and red: expression level over the mean) illustrates the mRNA relative expression (ΔCt) after global normalization is indicated at the bottom right of the figure.

**Figure 2:**
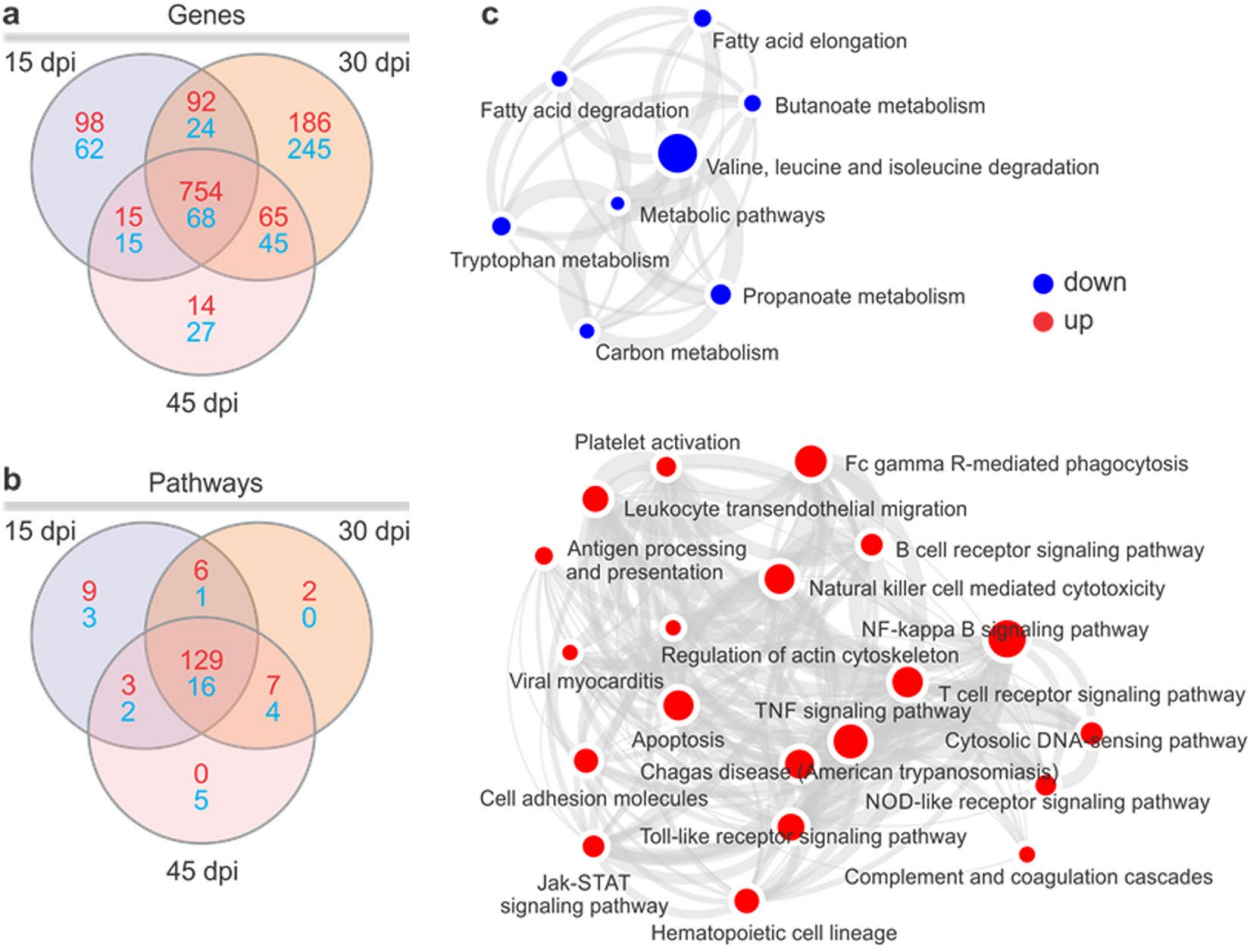
Venn diagram showing the number of differentially expressed (**a**) genes and (**b**) enriched canonical pathways in each time point. The enriched canonical pathways were identified by Ingenuity Pathway (IPA) analysis or (**c**) gene set enrichment analysis software (GSEA). (**c**) Enrichment map for experimental Chagas disease: Pre-ranked lists were analyzed for enrichment in sets of functionally related genes (pathways). Enrichment map was drawn representing the enriched pathways (nodes) as networks where the color corresponds to z-score based activation state prediction blue:downregulated and red:upregulated). Node size represents the gene set size in that pathway.

**Figure 3:**
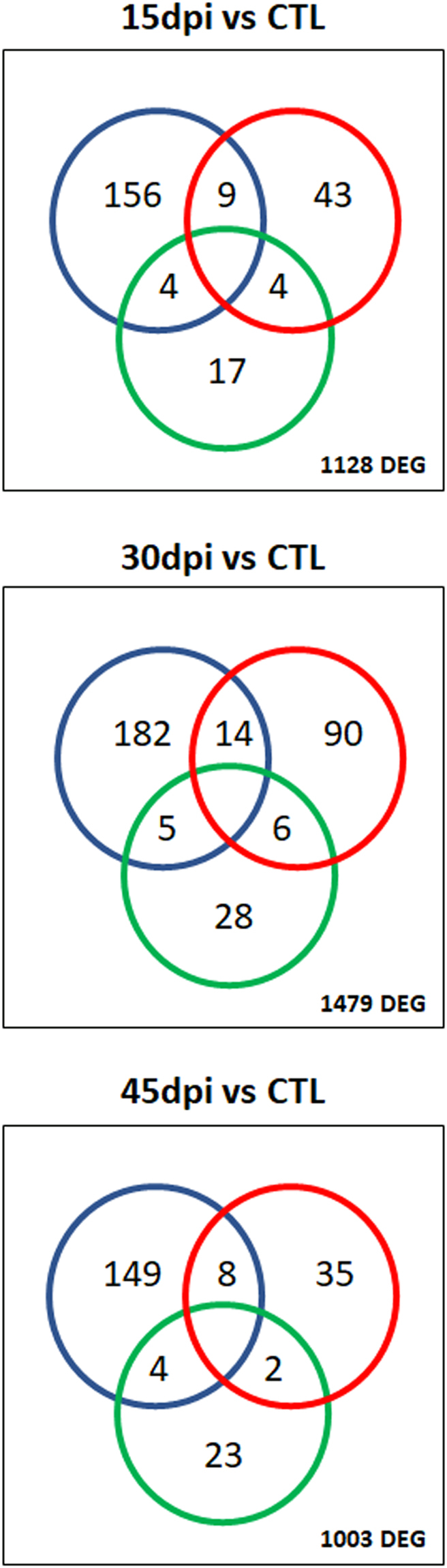
Venn diagrams showing the number of differentially expressed mitochondrial genes, and/or genes belonging to the IFNγ- and Nrf2- modulated transcriptional profile. Genes in each transcriptional profile were obtained from published data and mitochondrial genes were obtained from Gene Ontology as described in the Methods section. Graphs display the number of differentially expressed genes at the three time points. The Venn diagram includes three colored circles. Blue circles indicate the number of DEGs that are IFNγ-modulated, green circles indicate the number of Nrf2-modulated/antioxidant response genes and red circles indicate the number of mitochondrial genes.

Transcriptome analysis was performed in heart samples from acutely *T. cruzi*-infected mice at 15, 30 and 45 days, as well as in an uninfected control group. This mouse study was extensively described by Navarro *et al*.^33^. Infected mice were infected with the Colombiana *T*. *cruzi* strain. RNA extraction for mRNA expression profiling was performed the same tissue samples plus an extra one at the 45 dpi time point (Fig. 1a). Unsupervised analysis based on the top 500 genes with the higher expression variance showed segregation of the four groups both in principal component analysis (Fig. 1b; samples from each group clearly group together) and hierarchical clustering (1c), indicating gene expression profiles were specific to each time point. Analysis of differentially expressed genes revealed the highest number of gene expression changes at 30 dpi. The total number of DEGs was 1685. Supplemental Tables 1–3 depict the list of all DEGs at each time point. The number of up- or downregulated DEGs at each time point, as well as those shared between them are depicted in Fig. 2a and Supplemental Table 4. Figure 2b and Supplemental Table 5 indicate that multiple pathways are common to the 3 time points. Pathways analysis of the DEGs in all time points was performed with gene set enrichment analysis (GSEA). Metabolic pathways such as fatty acid and amino acid degradation are predicted to be downregulated (blue) and pathways/biological functions related to innate and acquired immune response are predicted to be upregulated (red) (Fig. 2c). Blood transcription module GSEA analysis was consistent with the gene expression signature of NK cells, monocytes, activated dendritic cells, T cells, and B cells in the hearts of *T. cruzi-*infected mice (Supplemental Fig. 1). Since IFNγ is the most upregulated cytokine in all time points (30-50-fold versus uninfected heart; Supplemental Tables 1–3), we matched our DEGs list to the genes into the IFNγ global transcriptional signature. We found that 219/1067 IFNγ target genes are differentially expressed in all time points - approximately 20% of the IFNγ signature (Supplemental Table 6). Myocardial oxidative stress^40^ and mitochondrial dysfunction are key processes in the pathogenesis as well as *in vitro* infection and *in vivo* murine models of Chagas disease^41^. To identify DEGs participating in the oxidative stress/antioxidant response processes, we matched the DEG lists with the transcriptional signature of nuclear factor-erythroid 2-related factor 2 (Nrf2), the master regulator of the antioxidant response^42^. Out of 341 Nrf2 target genes, we found 45 DEGs, or 13% of genes in the Nrf2 signature (Supplemental Table 6). We also matched the DEG list with the mitochondrion Gene Ontology classification. We found 123/1269 mitochondrial DEGs - approximately 10% of all mitochondrial genes - differentially expressed in the three time points (Supplemental Table 6). Several of the DEGs participate in more than one of these three processes (Fig. 3, Supplemental Table 7).

### DEM-DEG pairs and potential pathways regulated by miRNAs

**Figure 4:**
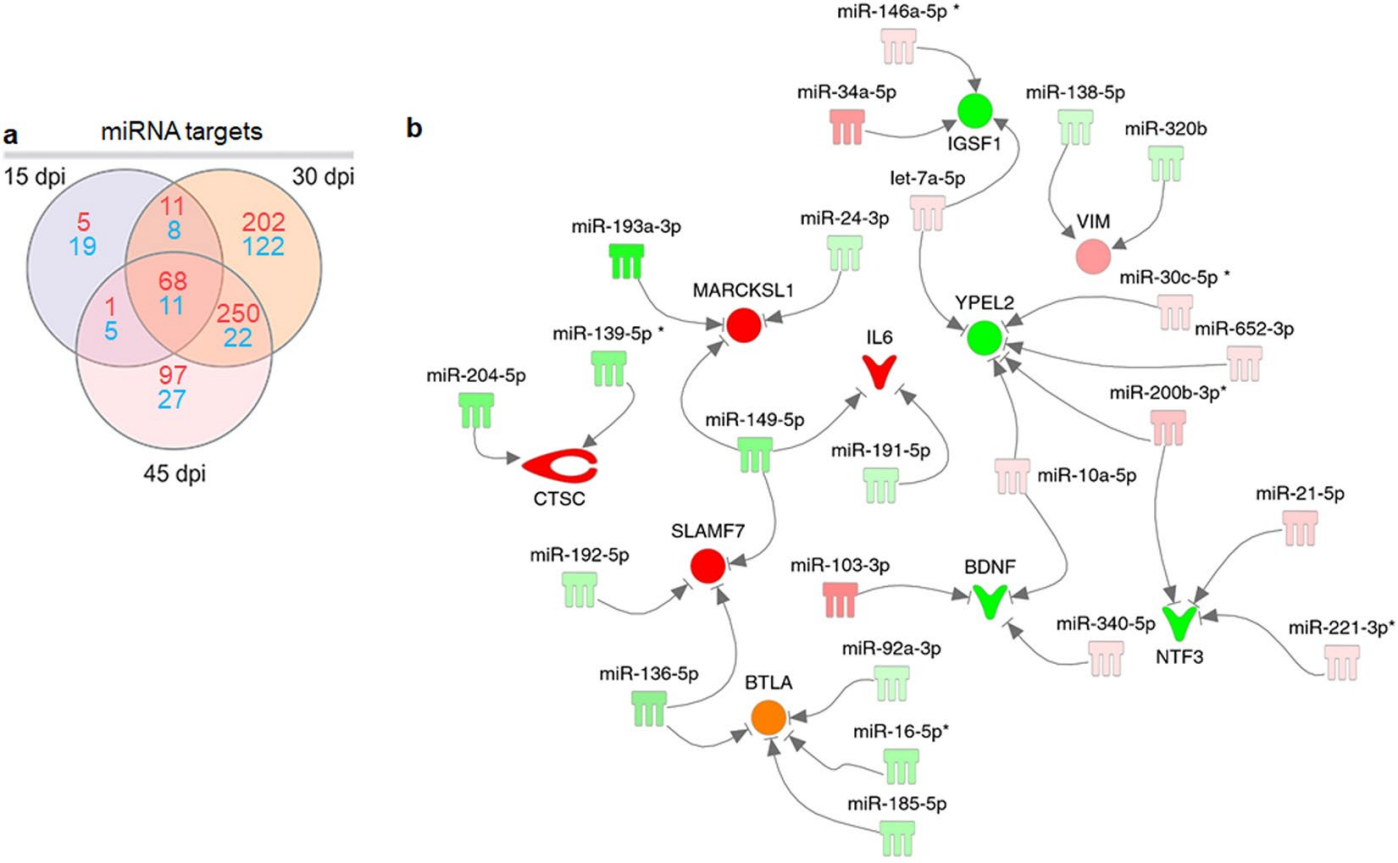
MiRNA target analysis and DEMs-DEGs Network. (**a**) Venn diagram showing the number of DEM targets (high predicted and experimentally validated targets) within the list of DEGs for each time point post infection as well as those that are shared among different time points. Red numbers indicate upregulated genes, blue numbers indicate downregulated genes. (**b**) A DEMs-DEGs network showing that individual targets associated with immunity, cardiovascular function or disease can be regulated by multiple miRNAs. The nodes are represented in graduation of red and green based on their fold change in expression at 30 dpi compared to the uninfected group.

**Figure 5:**
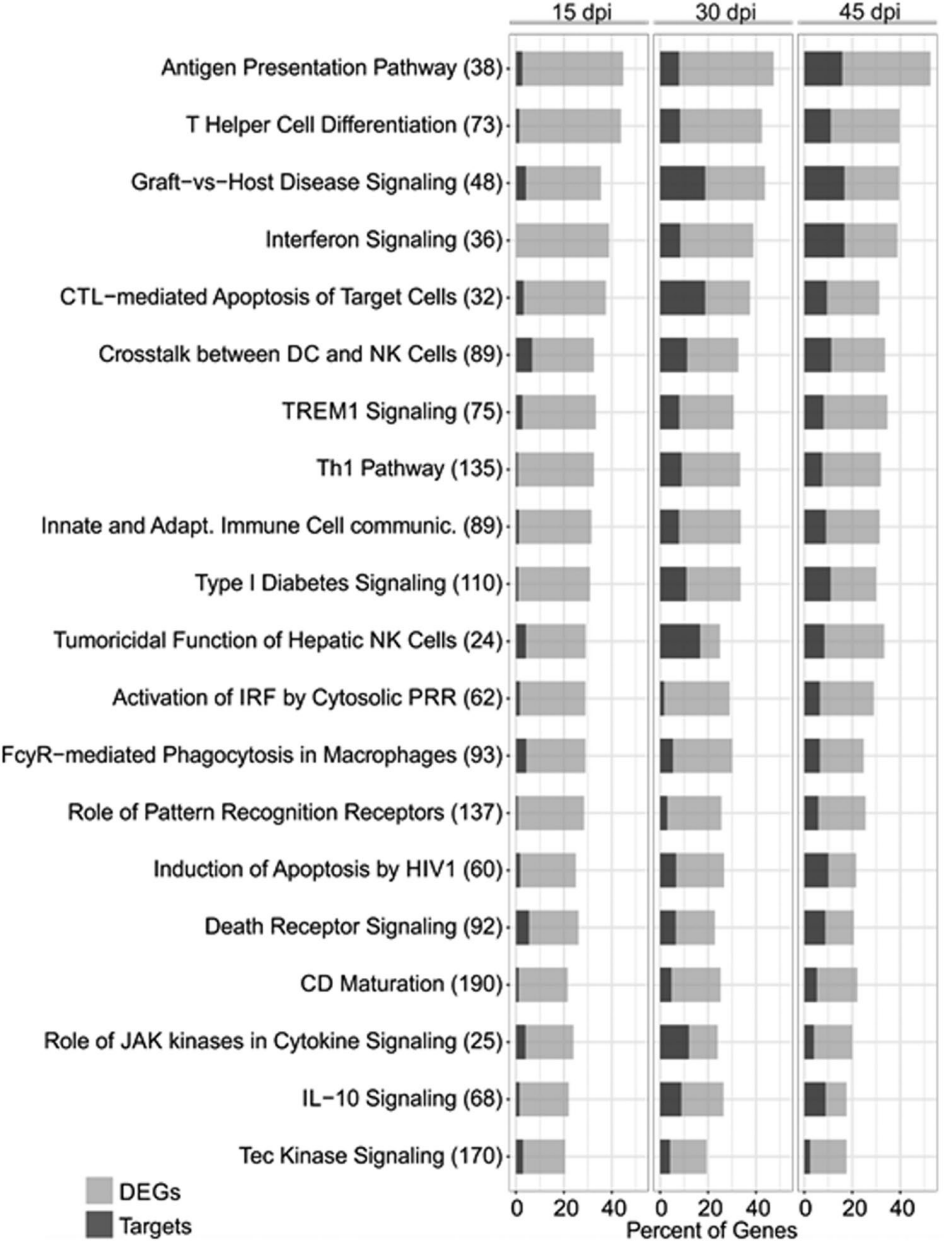
Ingenuity Pathway Analysis (IPA) canonical pathways most significantly enriched in the heart of *T. cruzi* infected mice. The stacked bar chart displays the percentage of target DEGs molecules present in each pathway. The numerical value in the parentheses in front each pathway name represents the total number of genes in that canonical pathway. The Benjamini-Hochberg (B.-H.) method was used to adjust the right-tailed Fisher’s exact test P-value, which was always <0.001.

**Figure 6:**
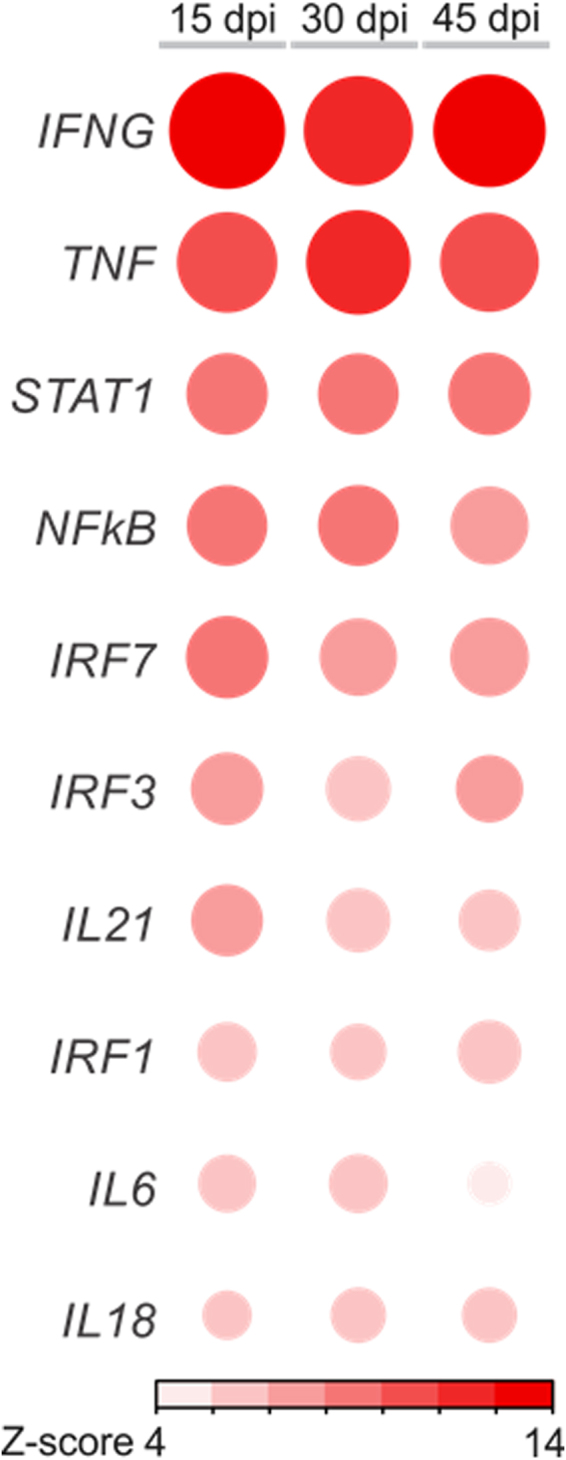
Upstream regulator analysis. Corrplot representation of the top10 upstream regulators. Cytokines and growth factors were only considered upstream regulators if were found to be differentially expressed and upregulated; otherwise they were excluded. Red or blue color represent activated or inhibited pathways, respectively, according to Z-score prediction a statistical calculation of the activation state.

Our previous results have shown 113 DEMs upon *T. cruzi* infection at 15, 30 and 45 days post infection^33^. The analysis of putative targets of DEMs within the DEGs list at each time point identified a total of 848 putative inversely paired targets of the 113 DEMs, using miRNA-target relationships predicted as high or experimentally validated (Fig. 4a). The top miRNAs (those with the highest number of targets in each time point) were miR-149-5p, miR-138-5p and miR-16-5p for 15, 30 and 45 dpi, with 14, 22 and 21 targets, respectively (targets described in Supplemental Tables 8–10). We also observed that several genes involved in cardiac physiology, pathophysiology and inflammation were simultaneously targeted by up to 5 distinct DEMs (Fig. 4b). Canonical pathways analysis of the 848 putative DEM targets was performed in each of the three time points. The most significantly enriched canonical pathways and the percentage of DEGs as well as DEGs that are targets of the DEMs in each pathway are shown in Fig. 5. We observed an enrichment in canonical pathways related to immune processes, like the Antigen Presentation Pathway, CTL-mediated Apoptosis of Target Cells, Role of Pattern Recognition Receptors and Th1 pathway and Interferon signaling. Upstream regulator analysis of the DEM targets indicated that IFNγ is the top upstream regulator at all time points, followed by other transcription factors and cytokines like TNFα, STAT1, NF-kB and IL-6 - which is itself a target of two DEMs at different time points (Fig. 6).

### DEM/DEG networks related to Chagas heart disease pathophysiological processes

**Figure 7:**
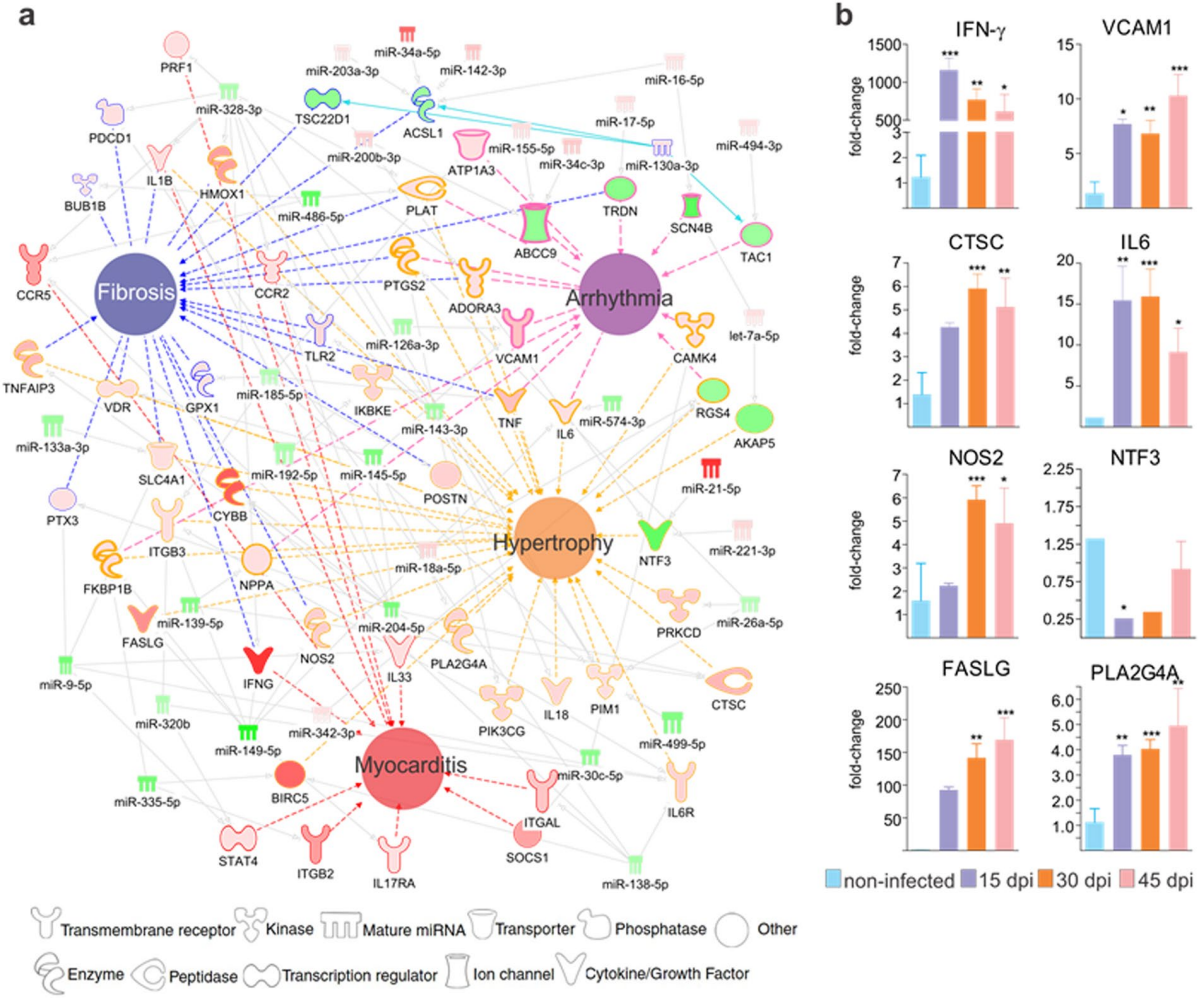
(**a**) DEMs-DEGs networks related to the main Chagas disease clinicopathogical features. Networks related to myocarditis, fibrosis, arrhythmia and hypertrophy were built using IPA software. Each built network contains molecules represented as nodes, and the biological relationship between two nodes is represented as an edge (line). All edges are supported by at least one reference from the literature, from a textbook, or from canonical information stored in the Ingenuity Knowledge Base (IKB). The molecules are represented in graduation of red and green based on their fold change in expression at 30 dpi compared to 0 dpi. Each node shape represents one type of molecule, as follows: (**b**) Validation of miRNA target expression by qPCR. Genes chosen for validation were network nodes which were direct miRNA targets belonging to each of the pathophysiology networks. The genes validated were IFN-g (interferon gamma), VCAM1 (vascular cell adhesion molecule 1), CTSC (cathepsin C), IL-6 (interleukin 6), NOS2 (nitric oxide synthase 2), NTF3 (Neurotrophin 3), FASLG (FAS Ligand) and PLA2G4A (Phospholipase A2, Group IVA). The expression was calculated as the mean ± s.d. for each group as individual data points using the relative expression (fold change over CONT) by the 2^−ΔΔct^ method, where Ct is the threshold cycle. Groups were compared by a non-parametrical test (Mann-Whitney Rank Sum Test) with GraphPad Prism software (version 5.0.4). Results were expressed as medians and interquartile ranges. *P-values were considered significant if <0.05.

In order to investigate the possible role of miRNAs in regulating the key pathophysiological processes in Chagas heart disease, we built networks with DEGs and DEMs in the 3 time points after *T. cruzi* infection, around each disease process (Arrhythmia, Fibrosis, Myocarditis and Hypertrophy of Heart). Figure 7a depicts the networks for the 30 dpi time point, which had the highest number of DEMs and DEGs. Significantly, several miRNA-targeted DEGs participate simultaneously in more than one pathophysiological process. Five out of the 35 DEMs in the network have targets involved in all four of the pathophysiological processes (miR-238-3p, miR-149-5p, miR-143-3p, miR-145-5p and miR-486-5p); an additional six DEMs target genes involved in three of the four processes (miR-138-5p, miR-9-5p, miR-26a-5pmiR-185-5p, miR-200b-3p and miR-335-5p). Similar observations were found in the pathophysiological process networks in the 15 and 45 dpi time points, and several of the DEMs and DEGs were the same as in the 30 dpi time point (data not shown). qPCR validation of microarray expression results analysis on 8 miRNA targets present in the different networks and time points confirmed differential expression. (Fig. 7b). To address the role of miRNAs in the regulation of mitochondrial genes, IFNγ or Nrf2-modulated genes in hearts of *T. cruzi*-infected mice, we performed target filtering analysis of DEMs and the mitochondrial DEGs. We found that 22 mitochondrial genes are targeted by 15 microRNAs in the three time points; 6 DEMs targeting 5 Nrf2-modulated DEGs; and 48 IFNγ -modulated genes were also targets of 32 DEMs (Supplemental Table 11).

## Discussion

The aim of this study was to provide the first interactome of miRNAs and their targetome in an acute *T. cruzi* infection animal model of Chagas heart disease. Using a systems biology approach, we integrated miRNA-mRNA expression profiles and identified the putative mRNA targets of differentially expressed miRNAs 15, 30 and 45 days after infection. This analysis was done between microRNA and mRNA - not protein - levels. So, our approach could hypothetically only find those gene targets where microRNA regulation of gene expression occurs at the mRNA degradation, rather than the translational level. A significant number of genes and miRNA were differentially expressed, and unsupervised analysis of gene expression profiles distinguished each time point group. Pathways analysis disclosed an enrichment in pathways related to the immune response, metabolism, mitochondria and the antioxidative stress response. Importantly, expression levels, pathways and upstream regulator analysis indicated that IFNγ is the key cytokine modulating transcriptional changes. In silico integration of differentially expressed miRNAs (DEMs) and genes (DEGs) revealed a large number of DEGs targeted by DEMs. Our data suggest that a few microRNAs may potentially regulate multiple key pathogenetically relevant pathways and processes, including fibrosis, hypertrophy, myocarditis and arrhythmia, IFNγ- and Nrf2-modulated and mitochondrial genes.

A significant number of genes and miRNA were differentially expressed, and unsupervised analysis of gene expression profiles distinguished each time point group. The finding that each time point after infection had a distinct gene expression profile, but roughly 50% of the DEGs are shared between the 3 time points indicates there are core DEGs related to infection. On the other hand, DEGs specific to each time point may indicate the existence of distinct functions that are selectively enriched in early or late points after infection. Among the enriched pathways and biological functions predicted to be inhibited and activated were those related to energy/fatty acid metabolism and immune system response to infection, respectivly. The finding that IFNγ is the most upregulated cytokine in the heart is in line with previous findings in murine infection and human Chagas cardiomyopathy studies^19,20,43^. In addition, the findings that IFNγ is the top upstream regulator, and that the IFN signaling pathway is enriched, corroborate IFNγ production/signaling are key factors in Chagas heart disease pathogenesis. Indeed, IFNγ produced by local activated Th1/Tc1 T cells and NK cells -whose transcriptional signature was found in our heart samples - induces NOS2 NO/peroxynitrite production and plays a crucial protective role against *T. cruzi* ^41^. This and other IFNγ-mediated gene changes^19,44^ also damage heart tissue and reduce cardiomyocyte function. NO/peroxynitrite production is involved in heart failure^40^. So, paradoxically, increased IFNγ levels essential for parasite control also cause heart tissue damage. Previous studies have described that fatty acids are the major energy sources in the normal heart, while a decreased fatty acid oxydation and shift to the glycolytic pathway - reducing ATP generation - has been observed in heart failure^45,46^. We and others have shown a dysregulation in gene, protein and activity of mitochondrial metabolism and control of oxidative stress, including reduced function of the electron transfer chain and creatine kinase system, key processes in cardiac ATP generation and oxidative stress; in the hearts of both *T. cruzi* infected mice^24–26^ and CCC patients^23,47,48^. Our results showed that *T. cruzi* infection powerfully modulates mitochondrial function genes involved in energy metabolism, oxidative stress, and apoptosis, in line with previous observations^41^. *In silico* integration of differentially expressed miRNAs (DEMs) and genes (DEGs) revealed a large number of DEGs targeted by DEMs. While the differential gene expression of immune-related and mitochondrial genes has already been described in the hearts of *T. cruzi*-infected mice^24–26^ and CCC patients^23^, our data are the first to indicate miRNAs may play a regulatory role in these transcriptome changes. The finding that multiple microRNAs target up to 30% of the differentially expressed genes in multiple important disease-associated pathways indicates a potentially important role of mRNAs in the control over each pathway. In addition, we found that some DEMs target multiple different targets, and that a relatively small set of DEMs target DEGs simultaneously involved in pathogenetically relevant processes like hypertrophy, fibrosis, arrhythmia, and myocarditis. DEM also targeted a significant number of mitochondrial DEGs, as well as DEGs that are modulated by IFNγ and Nrf2. Mitochondrial DEM targets participate in energy generation, apoptosis and oxidative stress. DEMs also play a putative role in regulating the Nrf2 antioxidant response pathway. miRNAs target several Nrf2-modulated genes (like the major effector of the antioxidant response heme oxygenase 1 HMOX1, targeted by miR-16-5p). In addition, miR-155-5p (upregulated in the 3 time points) and let-7a-5p (upregulated at 30 and 45 dpi) can increase the global Nrf2 transcriptional response by targeting translation of the transcriptional regulator BACH1^49^. This indicates that microRNAs comodulate the Nrf2 response with oxidative stress induced in *T. cruzi*-infected hearts. It has been described that chemical induction or Nrf2 or forced expression of Nrf2 or HMOX1 helps to control *T. cruzi* infection *in vitro* and *in vivo*, while Nrf2 of HMOX1 knockdown or treatment with antagonists increase parasitism^50^. This indicates that the parasite is adapted to the high oxidative environment that is part of the anti-infectious response and grows better in it^50^. Interestingly, the Nrf2 response plays a role in the overall cellular protection mechanisms. The Nrf2 pathway and HMOX1 have been reported to play a role in “tissue tolerance” - the ability of resist pathogen-, inflammation, or oxidative stress-mediated damage during infection or inflammation^51,52^.

Our study was performed in whole heart tissue, containing several cell types, including cardiomyocytes, fibroblasts, endothelial and infiltrating inflammatory cells. This means we cannot be certain that all these miRNA and mRNA changes occurred in the same cell type. For instance, it is likely that many of the immune response-associated DEGs were expressed in the infiltrating inflammatory cells, although infection and inflammation can trigger expression of inflammatory genes in heart parenchymal cells as well. However, it has been shown that miR-146a secreted in exosomes by endothelial cells, is captured by adjacent cardiomyocytes where it modulates gene expression and metabolic activity, suggesting miRNAs synthesized in a cell type could act on an adjacent cell^53^. An additional limitation of the present work is that it is a correlation study of miRNA and mRNA expression, without direct testing of regulatory function in appropriate gene expression systems. This correlation approach has been used to provide an overview of miRNA-mRNA networks that involve a high number of differentially expressed genes. Indeed, a broad systematic analysis is limited due to the fact that correlation is not proof of causality. Our results suggest that, by potentially targeting multiple genes in each of several disease pathways and pathobiologic processes, microRNAs may exert a combined regulatory effect that may be stronger than the effect of a microRNA targeting a single mRNA in a pathway. In addition, we found a small number of key “high-ranking” differentially expressed miRNAs - those with a high number of targets involved in several pathological processes. Our data identified specific molecular features of acute *T. cruzi* infection that may translate in the identification of novel therapeutic targets. *In vitro* and *in vivo* testing of targeting of key miRNA to induce the amplification of anti-*T. cruzi* and tissue protection mechanisms, like the Nrf2 pathway or HMOX1; or reduction of maladaptive responses, like mitochondrial dysfunction, may establish the functional or therapeutic relevance of miRNA regulation in the context of *T. cruzi* infection.

## Methods

### Ethics statement

This study was carried out in strict accordance with the recommendations in the Guide for the Care and Use of Laboratory Animals of the Brazilian National Council of Animal Experimentation (http://www.cobea.org.br/), the Guide for the Care and Use of Laboratory animals (NIH,2011) and the Brazilian Federal Law 11.794 (October 8, 2008). The Institutional Committee for Animal Ethics of Fiocruz (CEUA/Fiocruz, License 004/09) and the Institutional Committee for Animal Ethics of the University of São Paulo School of Medicine (CEUA/FMUSP, License 390/13) approved all the procedures used in this study. For euthanasia prior to excision of the hearts, mice were first tranquilized with diazepam (10 mg/kg, intraperitoneally) and then submitted to CO2 exposure.

### Mouse experimental infection and RNA isolation

Mouse, parasite strains and heart tissues used were the same as previously published^33^. Briefly, 36 C57BL6 mice (females; weight, 20 to 25 g), were intraperitoneally infected with 100 blood Colombiana *T. cruzi* Type I strain trypomastigotes for 15, 30 and 45 days. The level of parasitemia in tail blood was assessed daily by Brener’s method^54^. Therapeutic activity and criterion of cure on mice experimentally infected with *Trypanosoma cruzi*. The hearts of surviving animals that passed RNA quality control were used for gene and microRNA expression analysis. Heart ventricles from 16 mice (4 per group) were isolated for miRNA extraction using the mirVana™ miRNA Isolation Kit (Ambion, USA), following the manufacturers’ protocol (Fig. 1a). RNA extraction for mRNA expression profiling was performed the same tissue samples plus an extra one at the 45 dpi time point (Fig. 1a). Total RNA was isolated by mechanical disruption with the Precellys 24-bead-based homogenizer (Bertin Technologies, France) followed by extraction with the Trizol reagent (Life Technologies, USA) following the manufacturer’s protocol.

### miRNA and gene expression profiling

The miRNA profiling data was extracted from our group’s previous publication^33^. For gene expression profiling was done using the SurePrint G3 mouse Gene Expression v1 8 × 60 K arrays, the Low Input Quick Amp Labeling One-Color Kit (Agilent Technologies, USA) and the One-color RNA Spike-in Kit (Agilent Technologies) following the manufacturer’s standard protocol. 100 ng of total RNA input was used. After cDNA synthesis, amplification, labeling and purification, the reaction efficacy was checked using the NanoDrop (Thermo Scientific, USA). The hybridization was performed for 17 hours at 10 rpm and 65 °C in a Hybridization Oven (Robbins Scientific, USA). Slide wash was done according to the manufacturer’s recommendation. Cy3 intensities were detected by one-color scanning using Agilent DNA microarray scanner at 3 micron resolution. Raw data from scanned image files were extracted using Feature Extraction software (10.7.3.1). Spike-in used in the experiment gave reliable data and all arrays passed the quality control.

### Bioinformatic analysis

Sample raw fluorescence intensities were read, background corrected, log2 transformed and submitted to the quantile normalization with the LIMMA R package, following the Agilent probe to gene symbol annotation^55^. Probes replicates and matching the same gene were averaged to the highest mean across all samples. Data quality of the samples arrays and outliers removal were assessed with the array Quality Metrics R package^56^. Differentially expressed genes (DEG) between groups were determined by linear model fitting implemented in the LIMMA package. Comparisons p-values were submitted to false discovery rate adjustment according to the Benjamini-Hochberg method. Statistical significance threshold was defined as adjusted p-value ≤ 0.05. Genes were considered differentially expressed if presented an absolute fold change between groups greater than 2.0 (|FC| ≥2.0). The samples hierarchical clustering was carried out using the squared Euclidean distance of measure for the top 500 most variable genes. Principal component analysis (PCA) analysis was performed using all genes and the variance expression of the number of standard deviations from mean overall samples. Canonical pathways, networks analysis and Upstream regulator analysis were performed with Ingenuity Pathway Analysis (IPA, Qiagen). We also used published lists of IFNγ-induced/repressed genes^57^, Nrf2-induced/repressed genes and mitochondrion Gene Ontology classification to identify the corresponding DEGs. Pathways enrichment analysis was carried with the program Gene Set Enrichment Analysis^58^ to find induced or repressed pathways in the mice heart transcriptional profiles of each contrast between the infected and non-infected biological states. The normalized enrichment scores were calculated over 1000 of permutations using a FC pre-ranked list of genes and the Reactome gene sets^59^. The same experiment was also performed to find enriched immune cells using a previously described collection of gene sets of blood transcription modules^60^. The corresponding networks were drawn with the Cytoscape suite^61^, with a statistical significance threshold of adjusted p-value ≤ 0.01. The mRNA and miRNA expression profiles were integrated to identify potential functional interactions of miRNAs with their target genes using the IPA target filter tool based on the content of the 2016-03 release, incorporating the TargetScan, TarBase and miRecords algorithms. The lists of DEMs (miRNAs were considered differentially expressed if adjusted P < 0.05 and absolute fold change FC >1.5) in each time point were analyzed with the IPA software (2016-03) release. We thus obtained a list of high predicted and experimentally validated targets of the DEMs (considering the inverse pairing between DEMs and DEGs). IPA was also used to identify significantly enriched canonical pathways within the list of DEMs and their target DEGs (significance according to Fisher’s exact test). The same protocol was used to build DEMs-DEGs networks related to each of the main Chagas heart disease pathophysiological processes. DEMs regulating mitochondrial, IFNγ-dependent, and oxidative stress DEGs were also determined by inverse pairing with DEG lists belonging to each one of the processes.

## Electronic supplementary material

Supplementaray materials
