# Supplementary material for: Integration of miRNA and gene expression profiles suggest a role for miRNAs in the pathobiological processes of acute Trypanosoma cruzi infection
Source: Sci Rep. 2017 Dec 21;7:17990. doi: 10.1038/s41598-017-18080-9 (PMC5740174; doi:10.1038/s41598-017-18080-9)
Supplement: Supplementary file 1 — Supplementaray materials [file 41598_2017_18080_MOESM1_ESM.doc]

**Integration of miRNA and gene expression profiles suggest a role for miRNAs in the pathobiological processes of acute *Trypanosoma cruzi* infection**

Ludmila Rodrigues Pinto Ferreira^1,2,3,4,¥,*^, Frederico Moraes Ferreira^1,3,5,¥^, Laurie Laugier^6,¥^, Sandrine Cabantous^6^, Isabela Cunha Navarro^1,2,3^, Darlan da Silva Cândido^1,2,3^, Vagner Carvalho Rigaud^1,2,3^, Juliana Monte Real^7,8^, Glaucia Vilar Pereira^9^, Isabela Resende Pereira^9^, Leonardo Ruivo^9^, Ramendra Pati Pandey^1,2,3^, Marilda Savoia^1,2,3^, Jorge Kalil^1,2,3^, Joseli Lannes-Vieira^9^, Helder Nakaya^10,11^, Christophe Chevillard^6^,**^+^,**^🖃^, Edecio Cunha-Neto^1,2,3^,**^+,*^**

^¥^ Equal contribution, co-first author.

**^+^** Equal contribution, co-last author.

^1^ Laboratory of Immunology, Heart Institute (InCor), University of São Paulo School of Medicine, São Paulo, Brazil.

^2^ Division of Clinical Immunology and Allergy, University of São Paulo School of Medicine, São Paulo, Brazil.

^3^ Institute for Investigation in Immunology, iii-INCT, São Paulo, Brazil.

^4^ Departamento Morfologia, Instituto de Ciências Biológicas, Universidade Federal de Minas Gerais, Belo Horizonte, MG. Brazil.

^5^ Health Sciences, University of Santo Amaro, São Paulo, Brazil.

^6^ INSERM, U1108, Aix-Marseille University AMU, Faculté de Médecine, Marseille, France.

^7^ TUCCA Association for Children and Adolescents with Cancer, Department of Pediatric Oncology, Santa Marcelina Hospital, Sao Paulo, Brazil.

^8^ Centro de Investigação Translacional em Oncologia, Instituto do Câncer do Estado de São Paulo, Universidade de São Paulo, São Paulo, Brazil

^9^ Laboratory of Biology of Interactions, Oswaldo Cruz Institute – FIOCRUZ, Rio de Janeiro, Brazil.

^10^ Department of Pathophysiology and Toxicology, School of Pharmaceutical Sciences, University of São Paulo, 077010, São Paulo, Brazil

^11^ Department of Pathology, Emory University School of Medicine, Atlanta, GA 30322 U.S.A.

^*^ Correspondence to:

**Christophe Chevillard**, INSERM, U906, Aix-Marseille University AMU, Faculté de Médecine, Marseille, France email: christophe.chevillard@univ-amu.fr

**Ludmila Rodrigues P. Ferreira**, Laboratory of Immunology, Heart Institute (InCor), Avenida Dr. Enéas de Carvalho Aguiar, 44 – Bloco II, 9^o^ Andar. São Paulo, 05403-900 Brazil. e-mail: lucamargo@gmail.com

**Suplementary materials :**

**Supplemental. Figure 1** – Gene set enrichment analysis (GSEA). Corrplot analysis of gene expression results shows BTMs (Blood transcription modules) corresponding to immune cell types whose transcriptional signature was observed in the hearts of mice infected with *T.cruzi* for 15, 30 and 45 dpi.


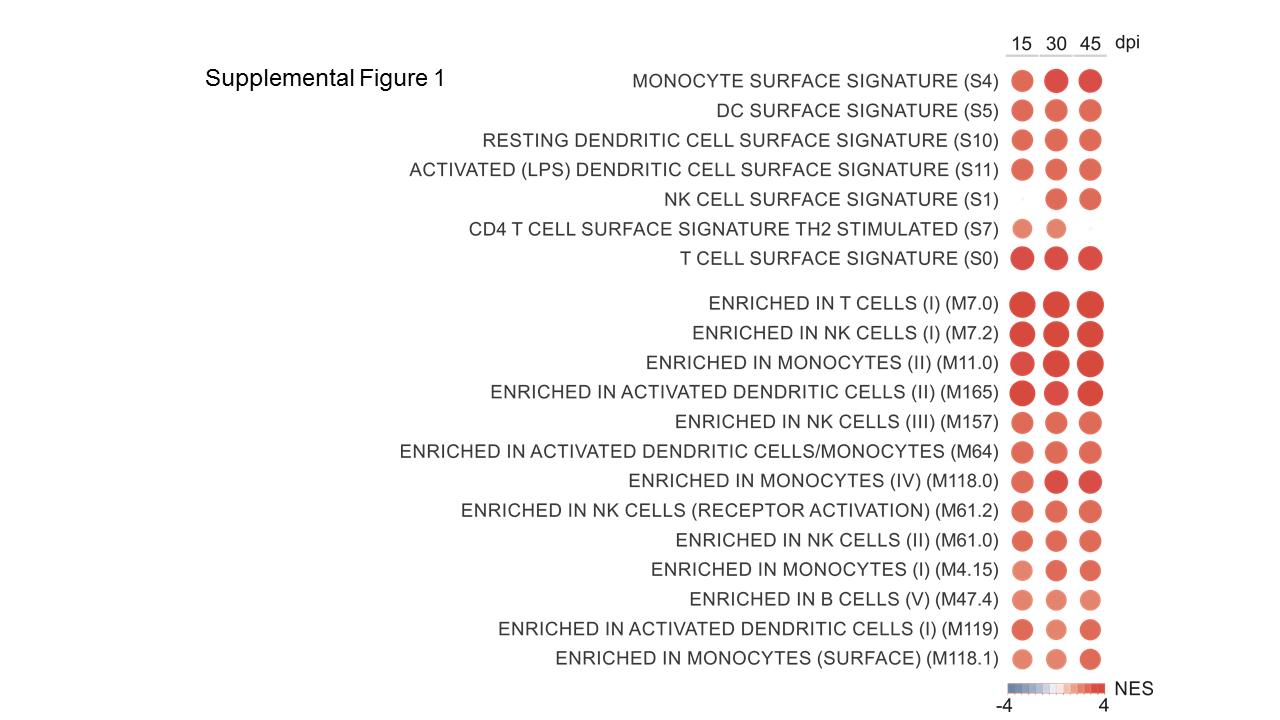


**Supplemental. Figure 1** – Gene set enrichment analysis (GSEA). Corrplot analysis of gene expression results shows BTMs (Blood transcription modules) corresponding to immune cell types whose transcriptional signature was observed in the hearts of mice infected with *T. cruzi* for 15, 30 and 45 dpi. Dot size represents the number of downstream genes, and intensity of red color indicates the statistical strength of the predicted activation state.

**Supplemental table 1:** List of all DEGs at 15 dpi.

| **Entrez Gene Name** | **Symbol** | **Expr p-value** | **Expr Fold Change** |
| --- | --- | --- | --- |
| granzyme B | Gzmb | 7,21E-16 | 228,68 |
| C-X-C motif chemokine ligand 10 | CXCL10 | 5,57E-13 | 178,46 |
| granzyme A | GZMA | 2,99E-13 | 178,03 |
| predicted gene 12250 | Gm12250 | 6,90E-14 | 146,19 |
| serine (or cysteine) peptidase inhibitor, clade A, member 3G | Serpina3g (includes others) | 1,75E-15 | 133,37 |
| chemokine (C-X-C motif) ligand 9 | Cxcl9 | 6,92E-13 | 110,4 |
| C-C motif chemokine ligand 5 | CCL5 | 5,06E-12 | 102,15 |
| ubiquitin D | UBD | 7,27E-14 | 95,435 |
| apolipoprotein L 9b | Apol9a/Apol9b | 1,15E-14 | 87,983 |
| chemokine (C-C motif) ligand 8 | Ccl8 | 1,86E-13 | 83,198 |
| serine (or cysteine) peptidase inhibitor, clade A, member 3G | Serpina3g (includes others) | 6,90E-14 | 76,418 |
| interferon gamma induced GTPase | Igtp | 1,36E-14 | 71,704 |
| apolipoprotein L 9b | Apol9a/Apol9b | 2,47E-15 | 71,213 |
| complement factor B | CFB | 6,65E-13 | 69,278 |
| guanylate binding protein 2 | GBP2 | 3,38E-12 | 67,484 |
| membrane-spanning 4-domains, subfamily A, member 4B | Ms4a4b (includes others) | 8,24E-14 | 64,608 |
| Z-DNA binding protein 1 | ZBP1 | 2,42E-14 | 64,124 |
| basic leucine zipper ATF-like transcription factor 2 | BATF2 | 1,36E-14 | 63,361 |
| chemokine (C-C motif) ligand 7 | Ccl7 | 4,76E-10 | 61,563 |
| interferon gamma inducible protein 47 | Ifi47 | 2,15E-13 | 57,821 |
| placenta specific 8 | PLAC8 | 7,06E-12 | 56,679 |
| CD274 molecule | CD274 | 3,19E-12 | 55,796 |
| T cell specific GTPase 1 | Tgtp1/Tgtp2 | 4,60E-11 | 54,383 |
| interferon gamma | IFNG | 5,46E-13 | 50,832 |
| chemokine (C-C motif) ligand 2 | Ccl2 | 1,12E-09 | 50,636 |
| interferon induced protein with tetratricopeptide repeats 3 | IFIT3 | 8,14E-15 | 48,917 |
| LCK proto-oncogene, Src family tyrosine kinase | LCK | 7,87E-13 | 48,08 |
| immunity-related GTPase family M member 1 | Irgm1 | 2,38E-15 | 47,045 |
| guanylate binding protein 4 | GBP4 | 6,78E-13 | 43,127 |
| T cell receptor beta, constant 2 | Trbc2 | 1,97E-12 | 38,309 |
| C-C motif chemokine ligand 4 | CCL4 | 2,83E-12 | 37,941 |
| suppressor of cytokine signaling 1 | SOCS1 | 2,36E-12 | 37,477 |
| T cell receptor beta, constant region 1 | Trbc1 | 1,00E-11 | 34,609 |
| immunity related GTPase M | IRGM | 2,56E-14 | 34,247 |
| Fc fragment of IgG receptor IIIa | FCGR3A/FCGR3B | 5,87E-14 | 32,501 |
| ISG15 ubiquitin-like modifier | ISG15 | 9,54E-13 | 30,494 |
| interferon inducible GTPase 1 | Iigp1 | 1,01E-14 | 29,777 |
| serum amyloid A 3 | Saa3 | 1,46E-06 | 29,307 |
| cholesterol 25-hydroxylase | CH25H | 2,14E-10 | 27,91 |
| CD6 molecule | CD6 | 2,58E-13 | 27,67 |
| proteasome subunit beta 8 | PSMB8 | 3,10E-15 | 27,635 |
| complement C2 | C2 | 1,04E-11 | 27,313 |
| integrin subunit beta 7 | ITGB7 | 1,97E-12 | 27,189 |
| interferon regulatory factor 7 | IRF7 | 1,01E-14 | 27,129 |
| guanylate binding protein 5 | GBP5 | 2,44E-10 | 26,961 |
| SLAM family member 8 | SLAMF8 | 2,04E-12 | 26,593 |
| CD3d molecule | CD3D | 3,91E-14 | 26,44 |
| CD2 molecule | CD2 | 2,24E-12 | 25,399 |
| CD5 molecule | CD5 | 1,14E-13 | 24,421 |
| predicted gene 9706 | Gm9706 | 1,08E-10 | 24,298 |
| predicted gene 4951 | Gm4951 | 4,86E-10 | 23,974 |
| zeta chain of T cell receptor associated protein kinase 70 | ZAP70 | 3,10E-15 | 23,629 |
| major histocompatibility complex, class II, DM beta | HLA-DMB | 2,03E-09 | 23,465 |
| transporter 1, ATP binding cassette subfamily B member | TAP1 | 1,86E-13 | 23,22 |
| histone cluster 1 H2A family member d | HIST1H2AD | 6,76E-11 | 23,18 |
| chromosome 15 open reading frame 48 | C15orf48 | 3,06E-08 | 23,042 |
| CD52 antigen | Cd52 | 2,37E-11 | 23,016 |
| proteasome subunit beta 9 | PSMB9 | 4,32E-13 | 21,924 |
| MHC class I family member | LOC547349 | 2,72E-16 | 20,933 |
| integrin subunit alpha X | ITGAX | 5,90E-11 | 20,874 |
| interleukin 12 receptor subunit beta 1 | IL12RB1 | 2,99E-13 | 20,215 |
| galectin 3 | LGALS3 | 2,53E-11 | 20,109 |
| BCL2 related protein A1 | BCL2A1 | 3,33E-10 | 19,361 |
| lymphotoxin beta | LTB | 5,24E-09 | 18,935 |
| interferon induced protein with tetratricopeptide repeats 2 | IFIT2 | 1,51E-11 | 18,889 |
| sialophorin | SPN | 2,66E-14 | 18,455 |
| GIMAP1-GIMAP5 readthrough | GIMAP1-GIMAP5 | 5,26E-10 | 18,114 |
| ras-related C3 botulinum toxin substrate 2 (rho family, small GTP binding protein Rac2) | RAC2 | 2,26E-13 | 17,805 |
| CD72 molecule | CD72 | 9,46E-11 | 17,666 |
| solute carrier family 2 member 6 | SLC2A6 | 4,01E-11 | 17,425 |
| C-X-C motif chemokine receptor 6 | CXCR6 | 4,88E-12 | 17,155 |
| C-C motif chemokine ligand 2 | CCL2 | 3,48E-09 | 16,87 |
| histocompatibility 2, Q region locus 8 | H2-Q8 | 6,00E-10 | 16,833 |
| interferon induced protein with tetratricopeptide repeats 1B | IFIT1B | 7,39E-12 | 16,044 |
| interferon regulatory factor 1 | IRF1 | 9,54E-13 | 15,946 |
| granzyme K | GZMK | 2,27E-12 | 15,909 |
| HCK proto-oncogene, Src family tyrosine kinase | HCK | 1,61E-11 | 15,827 |
| interleukin 2 receptor subunit beta | IL2RB | 1,37E-12 | 15,799 |
| cathepsin W | CTSW | 1,89E-13 | 15,601 |
| dual specificity phosphatase 2 | DUSP2 | 1,52E-09 | 15,28 |
| NLR family CARD domain containing 5 | NLRC5 | 7,69E-10 | 15,242 |
| CD3g molecule | CD3G | 8,56E-13 | 15,153 |
| protein tyrosine phosphatase, receptor type C associated protein | PTPRCAP | 3,48E-13 | 15,152 |
| T-box 21 | TBX21 | 1,27E-12 | 15,061 |
| family with sequence similarity 26 member F | FAM26F | 2,06E-12 | 14,8 |
| C-C motif chemokine receptor 5 (gene/pseudogene) | CCR5 | 1,73E-11 | 14,791 |
| macrophage receptor with collagenous structure | MARCO | 1,03E-06 | 14,734 |
| coronin 1A | CORO1A | 3,37E-11 | 14,663 |
| hexokinase 3 | HK3 | 6,58E-12 | 14,568 |
| poly(ADP-ribose) polymerase family member 14 | PARP14 | 1,97E-12 | 14,441 |
| src kinase associated phosphoprotein 1 | SKAP1 | 1,49E-11 | 14,384 |
| predicted gene 6252 | Gm6252 | 4,23E-13 | 14,255 |
| 2'-5' oligoadenylate synthetase-like 2 | Oasl2 | 5,89E-09 | 14,235 |
| interferon regulatory factor 8 | IRF8 | 6,65E-13 | 13,959 |
| major histocompatibility complex, class II, DM alpha | HLA-DMA | 6,48E-10 | 13,941 |
| serine (or cysteine) peptidase inhibitor, clade A, member 3H | Serpina3h | 5,03E-08 | 13,857 |
| acid phosphatase 5, tartrate resistant | Acp5 | 2,75E-11 | 13,73 |
| sodium voltage-gated channel alpha subunit 10 | SCN10A | 1,97E-12 | 13,718 |
| cathepsin S | CTSS | 2,81E-10 | 13,606 |
| allograft inflammatory factor 1 | AIF1 | 5,96E-10 | 13,506 |
| protein tyrosine phosphatase, receptor type C | PTPRC | 2,49E-12 | 13,364 |
| inducible T-cell costimulator | ICOS | 2,13E-09 | 13,305 |
| baculoviral IAP repeat containing 5 | BIRC5 | 1,16E-09 | 13,197 |
| myosin IG | MYO1G | 7,94E-12 | 13,098 |
| natural killer cell granule protein 7 | NKG7 | 2,93E-13 | 12,819 |
| XIAP associated factor 1 | XAF1 | 1,20E-13 | 12,686 |
| SAM and HD domain containing deoxynucleoside triphosphate triphosphohydrolase 1 | SAMHD1 | 2,17E-12 | 12,49 |
| CD74 molecule | CD74 | 2,80E-07 | 12,353 |
| IL2 inducible T-cell kinase | ITK | 1,97E-12 | 12,315 |
| linker for activation of T-cells | LAT | 4,56E-12 | 12,049 |
| C-X-C motif chemokine receptor 3 | CXCR3 | 1,00E-11 | 12,045 |
| toll-like receptor 12 | Tlr12 | 5,82E-11 | 11,935 |
| histocompatibility 2, Q region locus 5 | H2-Q5 | 2,26E-13 | 11,792 |
| integrin subunit beta 2 | ITGB2 | 4,18E-12 | 11,776 |
| interleukin 18 binding protein | IL18BP | 3,52E-13 | 11,728 |
| major histocompatibility complex, class I, E | HLA-E | 1,65E-13 | 11,597 |
| proteasome subunit beta 10 | PSMB10 | 1,07E-12 | 11,547 |
| bone marrow stromal cell antigen 1 | BST1 | 1,40E-11 | 11,463 |
| major histocompatibility complex, class II, DQ alpha 1 | HLA-DQA1 | 3,18E-07 | 11,388 |
| major histocompatibility complex, class II, DQ beta 1 | HLA-DQB1 | 1,07E-06 | 11,357 |
| cell division cycle associated 8 | CDCA8 | 3,58E-11 | 11,298 |
| TAP binding protein like | TAPBPL | 2,42E-14 | 11,278 |
| receptor transporter protein 4 | RTP4 | 4,17E-12 | 11,271 |
| bone marrow stromal cell antigen 2 | Bst2 | 6,18E-11 | 11,07 |
| killer cell lectin like receptor G1 | KLRG1 | 3,69E-12 | 10,948 |
| major histocompatibility complex, class II, DR beta 5 | HLA-DRB5 | 7,52E-07 | 10,822 |
| ubiquitin specific peptidase 18 | USP18 | 6,93E-12 | 10,819 |
| histocompatibility 2, T region locus 9 | H2-T9 | 2,34E-13 | 10,712 |
| macrophage expressed 1 | MPEG1 | 7,51E-12 | 10,643 |
| MX dynamin-like GTPase 1 | Mx1/Mx2 | 8,65E-10 | 10,617 |
| cystatin F | CST7 | 1,67E-11 | 10,556 |
| caspase 4 | CASP4 | 1,41E-10 | 10,511 |
| 2'-5'-oligoadenylate synthetase 1 | OAS1 | 5,64E-09 | 10,371 |
| 2'-5'-oligoadenylate synthetase like | OASL | 6,50E-10 | 10,35 |
| suppressor of cytokine signaling 3 | SOCS3 | 2,42E-09 | 10,336 |
| caspase recruitment domain family member 11 | CARD11 | 1,22E-13 | 10,294 |
| integrin subunit alpha L | ITGAL | 1,75E-11 | 10,23 |
| GTPase, very large interferon inducible 1 pseudogene | Gm4759 | 4,02E-10 | 10,081 |
| lymphocyte cytosolic protein 1 | LCP1 | 1,32E-10 | 10,017 |
| marker of proliferation Ki-67 | MKI67 | 1,66E-10 | 10,005 |
| histocompatibility 2, Q region locus 5 | H2-Q5 | 4,88E-12 | 9,981 |
| interferon induced protein 44 | IFI44 | 4,51E-11 | 9,736 |
| ADAM metallopeptidase domain 8 | ADAM8 | 5,80E-08 | 9,668 |
| histocompatibility 2, T region locus 10 | H2-T10 | 2,80E-12 | 9,557 |
| guanylate-binding protein 8 | Gbp8 | 5,36E-11 | 9,491 |
| coiled-coil domain containing 88B | CCDC88B | 1,50E-12 | 9,478 |
| vav guanine nucleotide exchange factor 1 | VAV1 | 1,73E-11 | 9,471 |
| IFI30, lysosomal thiol reductase | IFI30 | 3,47E-09 | 9,393 |
| killer cell lectin-like receptor, subfamily A, member 2 | Klra2 | 3,09E-12 | 9,367 |
| histone cluster 1 H2A family member b | HIST1H2AB | 3,27E-11 | 9,295 |
| protein tyrosine phosphatase, non-receptor type 6 | PTPN6 | 1,55E-09 | 9,281 |
| neutrophil cytosolic factor 4 | NCF4 | 1,42E-10 | 9,126 |
| lymphocyte antigen 86 | LY86 | 2,75E-09 | 9,118 |
| CD8b molecule | CD8B | 3,50E-08 | 9,052 |
| tumor necrosis factor | TNF | 4,56E-10 | 8,992 |
| E2F transcription factor 2 | E2F2 | 1,15E-10 | 8,886 |
| poly(ADP-ribose) polymerase family member 9 | PARP9 | 6,81E-12 | 8,794 |
| thymocyte selection associated family member 2 | THEMIS2 | 4,32E-12 | 8,776 |
| myosin IF | MYO1F | 9,07E-08 | 8,758 |
| vascular cell adhesion molecule 1 | VCAM1 | 1,37E-10 | 8,713 |
| S100 calcium binding protein A4 | S100A4 | 4,14E-10 | 8,662 |
| 2'-5' oligoadenylate synthetase 1F | Oas1f | 3,20E-10 | 8,619 |
| radical S-adenosyl methionine domain containing 2 | RSAD2 | 9,50E-09 | 8,58 |
| histone cluster 1, H1b | Hist1h1b | 1,94E-09 | 8,539 |
| apolipoprotein L 11a | Apol10a (includes others) | 3,57E-06 | 8,457 |
| deltex E3 ubiquitin ligase 3L | DTX3L | 7,53E-11 | 8,434 |
| interleukin 2 receptor subunit gamma | IL2RG | 3,62E-10 | 8,433 |
| 6-phosphofructo-2-kinase/fructose-2,6-biphosphatase 3 | PFKFB3 | 1,57E-09 | 8,364 |
| TIMP metallopeptidase inhibitor 1 | TIMP1 | 2,07E-06 | 8,313 |
| paired-Ig-like receptor A7 | Pira7 | 4,63E-10 | 8,269 |
| Fc fragment of IgE receptor Ig | FCER1G | 3,23E-10 | 8,235 |
| ring finger protein 19B | RNF19B | 1,17E-10 | 8,21 |
| polo like kinase 1 | PLK1 | 6,69E-10 | 8,023 |
| caspase 1 | CASP1 | 3,63E-10 | 7,99 |
| cyclin B2 | CCNB2 | 1,47E-08 | 7,968 |
| TNF receptor superfamily member 4 | TNFRSF4 | 2,47E-09 | 7,947 |
| olfactory receptor family 51 subfamily A member 7 | OR51A7 | 6,65E-13 | 7,935 |
| complement C3 | C3 | 7,37E-11 | 7,915 |
| ubiquitin like modifier activating enzyme 7 | UBA7 | 4,08E-11 | 7,891 |
| selectin P ligand | SELPLG | 1,15E-10 | 7,843 |
| solute carrier family 15 member 3 | SLC15A3 | 9,95E-10 | 7,816 |
| cytohesin 1 interacting protein | CYTIP | 1,95E-08 | 7,803 |
| guanylate binding protein family member 6 | GBP6 | 5,57E-11 | 7,789 |
| solute carrier family 16 member 3 | SLC16A3 | 1,78E-10 | 7,751 |
| coactosin like F-actin binding protein 1 | COTL1 | 8,80E-10 | 7,746 |
| C-X-C motif chemokine ligand 6 | CXCL6 | 8,28E-06 | 7,736 |
| 2'-5'-oligoadenylate synthetase 3 | OAS3 | 6,89E-11 | 7,734 |
| ring finger protein 213 | RNF213 | 5,55E-10 | 7,634 |
| SLAM family member 9 | SLAMF9 | 8,05E-11 | 7,605 |
| CD300 molecule like family member f | CD300LF | 8,41E-05 | 7,599 |
| LIM domain containing 2 | LIMD2 | 4,86E-10 | 7,588 |
| transmembrane protein 173 | TMEM173 | 3,44E-10 | 7,571 |
| G protein-coupled receptor 132 | GPR132 | 5,37E-09 | 7,552 |
| G protein-coupled receptor 171 | GPR171 | 3,58E-12 | 7,509 |
| TNF receptor superfamily member 18 | TNFRSF18 | 7,13E-12 | 7,478 |
| polypeptide N-acetylgalactosaminyltransferase 6 | GALNT6 | 7,17E-08 | 7,473 |
| CD40 molecule | CD40 | 5,19E-10 | 7,386 |
| complement C4B (Chido blood group) | C4A/C4B | 3,69E-12 | 7,35 |
| poly(ADP-ribose) polymerase family member 10 | PARP10 | 4,37E-13 | 7,344 |
| predicted gene 8369 | Gm8369 | 2,64E-08 | 7,324 |
| membrane spanning 4-domains A6A | MS4A6A | 7,45E-09 | 7,291 |
| histone cluster 1 H2A family member g | HIST1H2AG | 3,79E-11 | 7,262 |
| GLI pathogenesis related 1 | GLIPR1 | 2,83E-11 | 7,228 |
| FYN binding protein | FYB | 1,56E-11 | 7,226 |
| integrin subunit alpha 4 | ITGA4 | 4,34E-10 | 7,168 |
| transporter 2, ATP binding cassette subfamily B member | TAP2 | 8,93E-12 | 7,127 |
| basic leucine zipper ATF-like transcription factor 3 | BATF3 | 2,46E-08 | 7,072 |
| galectin 3 binding protein | LGALS3BP | 5,57E-12 | 7,064 |
| signal regulatory protein beta 1 | SIRPB1 | 6,37E-10 | 7,041 |
| SAM and SH3 domain containing 3 | SASH3 | 9,28E-12 | 7,034 |
| formyl peptide receptor 2 | FPR2 | 1,05E-05 | 6,982 |
| topoisomerase (DNA) II alpha | TOP2A | 3,59E-11 | 6,982 |
| sorting nexin 20 | SNX20 | 6,64E-10 | 6,974 |
| ubiquitin conjugating enzyme E2 L6 | UBE2L6 | 1,32E-10 | 6,949 |
| G protein subunit gamma 2 | GNG2 | 1,05E-10 | 6,904 |
| B cell leukemia/lymphoma 2 related protein A1c | Bcl2a1c | 7,33E-10 | 6,878 |
| protein tyrosine phosphatase, non-receptor type 22 | PTPN22 | 5,06E-09 | 6,867 |
| paired-Ig-like receptor A11 | Pira11 | 9,97E-10 | 6,836 |
| hematopoietic cell signal transducer | HCST | 8,05E-12 | 6,782 |
| colony stimulating factor 2 receptor beta common subunit | CSF2RB | 5,23E-08 | 6,754 |
| regulator of G-protein signaling 1 | RGS1 | 6,19E-11 | 6,724 |
| deoxyribonuclease 1 like 3 | DNASE1L3 | 2,13E-10 | 6,715 |
| C-type lectin domain family 4 member E | CLEC4E | 5,24E-05 | 6,695 |
| FCH domain only 1 | FCHO1 | 1,61E-10 | 6,666 |
| CD48 molecule | CD48 | 4,88E-11 | 6,661 |
| tripartite motif containing 21 | TRIM21 | 1,65E-11 | 6,656 |
| apolipoprotein L 11a | Apol10a (includes others) | 2,10E-08 | 6,655 |
| CD3e molecule | CD3E | 4,51E-11 | 6,644 |
| interleukin 21 receptor | IL21R | 1,24E-09 | 6,621 |
| leucine rich repeat containing 25 | LRRC25 | 5,96E-09 | 6,559 |
| tumor necrosis factor superfamily member 10 | TNFSF10 | 4,29E-08 | 6,556 |
| pleckstrin | PLEK | 2,43E-08 | 6,528 |
| C-C motif chemokine receptor 2 | CCR2 | 3,81E-10 | 6,522 |
| inhibitor of DNA binding 2, HLH protein | ID2 | 3,00E-08 | 6,494 |
| RAS guanyl releasing protein 1 | RASGRP1 | 2,18E-10 | 6,494 |
| beta-2-microglobulin | B2M | 8,42E-09 | 6,399 |
| cyclin A2 | CCNA2 | 1,48E-09 | 6,398 |
| centromere protein E | CENPE | 5,32E-10 | 6,371 |
| cytochrome P450 family 4 subfamily F member 2 | CYP4F2 | 2,57E-08 | 6,34 |
| gap junction protein delta 3 | GJD3 | 4,86E-10 | 6,331 |
| leukocyte immunoglobulin like receptor B3 | LILRB3 | 2,52E-10 | 6,301 |
| ras homolog family member F, filopodia associated | RHOF | 1,18E-10 | 6,274 |
| mitogen-activated protein kinase kinase kinase kinase 1 | MAP4K1 | 4,14E-12 | 6,259 |
| signal transducer and activator of transcription 2 | STAT2 | 6,58E-12 | 6,258 |
| histidine decarboxylase | HDC | 5,27E-07 | 6,216 |
| kinesin family member 21B | KIF21B | 1,86E-09 | 6,191 |
| leukocyte associated immunoglobulin like receptor 1 | LAIR1 | 2,63E-09 | 6,162 |
| CD8a molecule | CD8A | 3,45E-07 | 6,157 |
| uridine phosphorylase 1 | UPP1 | 5,34E-07 | 6,156 |
| Thy-1 cell surface antigen | THY1 | 2,39E-09 | 6,136 |
| proline-serine-threonine phosphatase interacting protein 1 | PSTPIP1 | 1,55E-09 | 6,125 |
| docking protein 2 | DOK2 | 1,21E-09 | 6,103 |
| SLAM family member 7 | SLAMF7 | 1,46E-11 | 6,097 |
| synaptotagmin like 3 | SYTL3 | 2,36E-08 | 6,096 |
| interleukin 10 receptor subunit alpha | IL10RA | 7,89E-10 | 6,079 |
| cytohesin 4 | CYTH4 | 1,99E-08 | 6,064 |
| schlafen 2 | Slfn2 | 3,61E-09 | 6,033 |
| Fc fragment of IgG receptor Ia | FCGR1A | 7,69E-10 | 6,013 |
| NFKB inhibitor delta | NFKBID | 1,41E-09 | 5,986 |
| TNF receptor associated factor 1 | TRAF1 | 2,38E-09 | 5,983 |
| hematopoietic cell-specific Lyn substrate 1 | HCLS1 | 3,59E-11 | 5,978 |
| tripartite motif-containing 30A | Trim30a/Trim30d | 4,21E-10 | 5,964 |
| junction adhesion molecule like | JAML | 4,59E-05 | 5,958 |
| interferon induced with helicase C domain 1 | IFIH1 | 2,85E-11 | 5,936 |
| solute carrier family 11 member 1 | SLC11A1 | 3,47E-09 | 5,926 |
| schlafen family member 13 | SLFN13 | 8,42E-11 | 5,919 |
| tryptophanyl-tRNA synthetase | WARS | 6,97E-12 | 5,918 |
| protein tyrosine phosphatase, non-receptor type 7 | PTPN7 | 8,42E-11 | 5,916 |
| DExD/H-box helicase 58 | DDX58 | 7,96E-11 | 5,875 |
| WD repeat and FYVE domain containing 1 | WDFY1 | 3,65E-08 | 5,866 |
| Rho GTPase activating protein 4 | ARHGAP4 | 4,49E-10 | 5,852 |
| tubulin beta 3 class III | TUBB3 | 2,15E-06 | 5,85 |
| CD300e molecule | CD300E | 3,30E-05 | 5,78 |
| mixed lineage kinase domain like pseudokinase | MLKL | 1,24E-10 | 5,778 |
| N-acylethanolamine acid amidase | NAAA | 8,68E-10 | 5,752 |
| killer cell lectin like receptor C1 | KLRC1 | 1,03E-08 | 5,735 |
| glia maturation factor gamma | GMFG | 5,76E-09 | 5,703 |
| basic leucine zipper ATF-like transcription factor | BATF | 1,79E-10 | 5,697 |
| B-cell CLL/lymphoma 3 | BCL3 | 8,75E-09 | 5,681 |
| protein C receptor | PROCR | 1,78E-09 | 5,648 |
| GTPase, IMAP family member 7 | GIMAP7 | 9,19E-09 | 5,627 |
| C-C motif chemokine ligand 3 like 3 | CCL3L3 | 1,12E-05 | 5,622 |
| signal transducer and activator of transcription 1 | STAT1 | 1,31E-10 | 5,617 |
| intercellular adhesion molecule 1 | ICAM1 | 6,30E-10 | 5,605 |
| ubiquitin like with PHD and ring finger domains 1 | UHRF1 | 3,65E-08 | 5,589 |
| GTPase, very large interferon inducible 1 | Gvin1 (includes others) | 1,03E-05 | 5,575 |
| major histocompatibility complex, class I, G | HLA-G | 1,98E-09 | 5,568 |
| pannexin 1 | PANX1 | 1,90E-09 | 5,567 |
| Rac GTPase activating protein 1 | RACGAP1 | 1,34E-10 | 5,551 |
| paired immunoglobin like type 2 receptor alpha | PILRA | 1,98E-08 | 5,548 |
| Rho GTPase activating protein 45 | ARHGAP45 | 2,92E-10 | 5,54 |
| cell division cycle associated 5 | CDCA5 | 9,34E-09 | 5,532 |
| Rho family GTPase 1 | RND1 | 7,47E-09 | 5,512 |
| lysosomal protein transmembrane 5 | LAPTM5 | 1,29E-08 | 5,471 |
| Src-like-adaptor | SLA | 8,64E-08 | 5,469 |
| parvin gamma | PARVG | 1,43E-09 | 5,409 |
| Fas ligand | FASLG | 8,55E-12 | 5,385 |
| kinesin family member 22 | KIF22 | 1,59E-07 | 5,369 |
| C-C motif chemokine ligand 19 | CCL19 | 5,63E-08 | 5,359 |
| cytochrome b-245 beta chain | CYBB | 2,46E-08 | 5,358 |
| RAB32, member RAS oncogene family | RAB32 | 1,94E-09 | 5,355 |
| semaphorin 4D | SEMA4D | 1,59E-08 | 5,351 |
| cathepsin C | CTSC | 3,48E-10 | 5,349 |
| ring finger protein 19B | RNF19B | 2,59E-06 | 5,335 |
| NFKB inhibitor epsilon | NFKBIE | 1,09E-09 | 5,334 |
| complement C1r | C1R | 2,51E-11 | 5,318 |
| GLI pathogenesis related 2 | GLIPR2 | 1,53E-09 | 5,279 |
| semaphorin 4A | SEMA4A | 1,50E-09 | 5,276 |
| membrane-spanning 4-domains, subfamily A, member 6C | Ms4a6c | 4,23E-07 | 5,273 |
| phospholipase D family member 4 | PLD4 | 9,63E-09 | 5,272 |
| phosphatidylinositol-4,5-bisphosphate 3-kinase catalytic subunit delta | PIK3CD | 1,25E-07 | 5,26 |
| C-type lectin domain family 4, member a3 | Clec4a3 | 7,57E-08 | 5,253 |
| zinc finger protein 705A | ZNF705A | 1,40E-09 | 5,238 |
| minichromosome maintenance complex component 5 | MCM5 | 1,43E-09 | 5,222 |
| eukaryotic translation initiation factor 2 alpha kinase 2 | EIF2AK2 | 4,94E-12 | 5,22 |
| cell division cycle associated 3 | CDCA3 | 2,56E-07 | 5,215 |
| ecotropic viral integration site 2A | EVI2A | 4,35E-07 | 5,182 |
| fermitin family member 3 | FERMT3 | 2,14E-10 | 5,173 |
| interferon induced protein 35 | IFI35 | 2,06E-12 | 5,158 |
| proteasome activator subunit 2 | PSME2 | 2,41E-12 | 5,135 |
| protein regulator of cytokinesis 1 | PRC1 | 3,50E-08 | 5,134 |
| ubiquitin conjugating enzyme E2 C | UBE2C | 1,21E-09 | 5,127 |
| G protein-coupled receptor 68 | GPR68 | 5,89E-08 | 5,107 |
| nucleoporin 210 | NUP210 | 4,02E-10 | 5,106 |
| interleukin 21 | IL21 | 5,04E-06 | 5,097 |
| DExH-box helicase 58 | DHX58 | 2,29E-08 | 5,064 |
| fibrinogen like 2 | FGL2 | 4,02E-10 | 5,064 |
| CD69 molecule | CD69 | 1,41E-10 | 5,058 |
| programmed cell death 1 ligand 2 | PDCD1LG2 | 1,15E-06 | 5,043 |
| sorting nexin 22 | SNX22 | 2,24E-06 | 5,036 |
| leupaxin | LPXN | 3,05E-07 | 5,004 |
| histocompatibility 2, M region locus 11 | H2-M11 | 8,89E-07 | 4,983 |
| neuralized E3 ubiquitin protein ligase 3 | NEURL3 | 9,91E-08 | 4,967 |
| RAB19, member RAS oncogene family | RAB19 | 8,80E-10 | 4,95 |
| zinc finger MYND-type containing 15 | ZMYND15 | 9,97E-10 | 4,943 |
| Ras association domain family member 5 | RASSF5 | 5,43E-08 | 4,874 |
| heme oxygenase 1 | HMOX1 | 1,00E-05 | 4,864 |
| G protein-coupled receptor 65 | GPR65 | 8,89E-10 | 4,852 |
| predicted gene 8995 | Gm8995 | 8,53E-05 | 4,851 |
| complement C1q A chain | C1QA | 1,56E-09 | 4,813 |
| NCK associated protein 1 like | NCKAP1L | 1,50E-08 | 4,812 |
| toll like receptor 2 | TLR2 | 3,10E-09 | 4,796 |
| cytidine/uridine monophosphate kinase 2 | CMPK2 | 1,08E-09 | 4,793 |
| insulin receptor related receptor | INSRR | 9,93E-10 | 4,783 |
| coronin 2A | CORO2A | 2,28E-09 | 4,769 |
| N-myc and STAT interactor | NMI | 6,66E-12 | 4,766 |
| transmembrane protein 140 | TMEM140 | 1,40E-08 | 4,764 |
| unc-13 homolog D | UNC13D | 5,08E-09 | 4,752 |
| complement C1q B chain | C1QB | 2,58E-09 | 4,751 |
| MYB proto-oncogene like 2 | MYBL2 | 6,40E-09 | 4,75 |
| TYRO protein tyrosine kinase binding protein | TYROBP | 1,08E-07 | 4,75 |
| membrane spanning 4-domains A7 | MS4A7 | 3,97E-07 | 4,748 |
| poly(ADP-ribose) polymerase family member 12 | PARP12 | 6,23E-10 | 4,747 |
| beta-1,4-N-acetyl-galactosaminyltransferase 1 | B4GALNT1 | 9,02E-10 | 4,725 |
| Rho GDP dissociation inhibitor beta | ARHGDIB | 2,52E-09 | 4,72 |
| MEFV, pyrin innate immunity regulator | MEFV | 4,70E-07 | 4,719 |
| regulator of G-protein signaling 14 | RGS14 | 1,58E-10 | 4,71 |
| growth factor independent 1 transcriptional repressor | GFI1 | 1,51E-10 | 4,687 |
| CD7 molecule | CD7 | 8,57E-06 | 4,677 |
| killer cell lectin-like receptor subfamily A, member 22 | Klra22 | 6,39E-08 | 4,665 |
| MX dynamin-like GTPase 1 | Mx1/Mx2 | 2,57E-09 | 4,659 |
| interleukin 18 receptor 1 | IL18R1 | 6,85E-09 | 4,64 |
| C-X-C motif chemokine ligand 16 | CXCL16 | 1,31E-08 | 4,636 |
| granzyme C | Gzmc | 5,46E-08 | 4,629 |
| methylenetetrahydrofolate dehydrogenase (NADP+ dependent) 2, methenyltetrahydrofolate cyclohydrolase | MTHFD2 | 3,37E-09 | 4,629 |
| Wiskott-Aldrich syndrome | WAS | 2,93E-10 | 4,622 |
| SPC25, NDC80 kinetochore complex component | SPC25 | 7,97E-09 | 4,621 |
| xanthine dehydrogenase | XDH | 3,12E-08 | 4,615 |
| heparanase | HPSE | 2,64E-08 | 4,604 |
| killer cell lectin like receptor D1 | KLRD1 | 3,40E-09 | 4,603 |
| centromere protein J | CENPJ | 2,08E-09 | 4,599 |
| RB transcriptional corepressor like 1 | RBL1 | 3,58E-10 | 4,587 |
| CD53 molecule | CD53 | 4,51E-10 | 4,586 |
| septin 1 | SEPT1 | 6,60E-11 | 4,576 |
| complement C1q C chain | C1QC | 9,47E-09 | 4,564 |
| absent in melanoma 1 | AIM1 | 1,82E-10 | 4,534 |
| SP140 nuclear body protein | SP140 | 7,50E-09 | 4,533 |
| interferon, alpha-inducible protein 27 like 2A | Ifi27l2a/Ifi27l2b | 2,83E-08 | 4,528 |
| interleukin 4 induced 1 | IL4I1 | 1,67E-06 | 4,522 |
| pleckstrin and Sec7 domain containing 4 | PSD4 | 4,81E-09 | 4,496 |
| Epstein-Barr virus induced 3 | EBI3 | 1,86E-05 | 4,46 |
| DENN domain containing 2D | DENND2D | 8,81E-12 | 4,458 |
| perforin 1 | PRF1 | 1,16E-09 | 4,457 |
| TAP binding protein | TAPBP | 1,56E-09 | 4,443 |
| BCL2 like 14 | BCL2L14 | 2,84E-11 | 4,442 |
| FYVE, RhoGEF and PH domain containing 2 | FGD2 | 1,33E-08 | 4,442 |
| myelin basic protein | MBP | 5,52E-11 | 4,439 |
| protein tyrosine phosphatase, non-receptor type 18 | PTPN18 | 5,27E-07 | 4,423 |
| DENN domain containing 1C | DENND1C | 9,85E-09 | 4,416 |
| argininosuccinate synthase 1 | ASS1 | 1,85E-08 | 4,375 |
| potassium calcium-activated channel subfamily N member 4 | KCNN4 | 6,17E-09 | 4,361 |
| FXYD domain containing ion transport regulator 5 | FXYD5 | 1,67E-08 | 4,359 |
| TNF alpha induced protein 3 | TNFAIP3 | 1,11E-06 | 4,359 |
| phosphoglycerate dehydrogenase | PHGDH | 1,09E-08 | 4,348 |
| membrane-spanning 4-domains, subfamily A, member 4B | Ms4a4b (includes others) | 5,10E-10 | 4,344 |
| fidgetin like 1 | FIGNL1 | 4,08E-07 | 4,323 |
| cyclin B1 | CCNB1 | 6,62E-06 | 4,318 |
| GTPase, IMAP family member 4 | GIMAP4 | 1,06E-05 | 4,312 |
| major histocompatibility complex, class I, A | HLA-A | 3,11E-05 | 4,31 |
| thymosin, beta 4, X chromosome | Tmsb4x (includes others) | 7,11E-08 | 4,308 |
| interferon regulatory factor 5 | IRF5 | 3,83E-09 | 4,307 |
| killer cell lectin-like receptor, subfamily A, member 16 | Klra16 | 3,10E-06 | 4,307 |
| capping actin protein, gelsolin like | CAPG | 2,46E-10 | 4,289 |
| leukotriene B4 receptor | LTB4R | 3,05E-04 | 4,264 |
| amyloid beta precursor protein binding family B member 1 interacting protein | APBB1IP | 9,98E-09 | 4,247 |
| C-X3-C motif chemokine receptor 1 | CX3CR1 | 2,29E-03 | 4,245 |
| phosphoglycerate dehydrogenase | PHGDH | 9,65E-09 | 4,243 |
| phosphoserine aminotransferase 1 | PSAT1 | 2,62E-08 | 4,243 |
| sialic acid binding Ig like lectin 1 | SIGLEC1 | 6,95E-09 | 4,235 |
| zinc finger and BTB domain containing 32 | ZBTB32 | 9,86E-10 | 4,235 |
| TPX2, microtubule nucleation factor | TPX2 | 6,36E-09 | 4,232 |
| toll like receptor 1 | TLR1 | 1,49E-07 | 4,222 |
| BH3 interacting domain death agonist | BID | 8,84E-10 | 4,217 |
| asparagine synthetase (glutamine-hydrolyzing) | ASNS | 5,35E-08 | 4,215 |
| lymphocyte activating 3 | LAG3 | 9,67E-10 | 4,215 |
| sorting nexin 10 | SNX10 | 1,64E-07 | 4,21 |
| signal transducer and activator of transcription 4 | STAT4 | 4,33E-10 | 4,204 |
| regulator of G-protein signaling 16 | RGS16 | 2,70E-06 | 4,202 |
| villin like | VILL | 3,93E-08 | 4,195 |
| Fc fragment of IgG receptor IIb | FCGR2B | 1,83E-06 | 4,191 |
| SH2 domain containing 1A | SH2D1A | 1,61E-08 | 4,182 |
| syntaxin 11 | STX11 | 6,48E-07 | 4,158 |
| lymphoid restricted membrane protein | LRMP | 2,47E-10 | 4,151 |
| phorbol-12-myristate-13-acetate-induced protein 1 | Pmaip1 | 3,03E-08 | 4,147 |
| CD44 molecule (Indian blood group) | CD44 | 1,60E-06 | 4,143 |
| ribonucleotide reductase regulatory subunit M2 | RRM2 | 2,10E-08 | 4,141 |
| LY6/PLAUR domain containing 6B | LYPD6B | 1,72E-09 | 4,134 |
| FGR proto-oncogene, Src family tyrosine kinase | FGR | 4,86E-08 | 4,128 |
| apolipoprotein L 7e | Apol7e (includes others) | 3,09E-10 | 4,119 |
| receptor interacting serine/threonine kinase 3 | RIPK3 | 7,57E-08 | 4,117 |
| transforming growth factor beta induced | TGFBI | 1,81E-05 | 4,114 |
| SH3 domain binding protein 2 | SH3BP2 | 1,48E-09 | 4,113 |
| pentraxin 3 | PTX3 | 2,29E-04 | 4,109 |
| C-C motif chemokine receptor like 2 | CCRL2 | 1,43E-10 | 4,102 |
| complement factor properdin | CFP | 4,50E-07 | 4,077 |
| CD68 molecule | CD68 | 1,72E-03 | 4,073 |
| transmembrane protein 56 | TMEM56 | 1,22E-07 | 4,066 |
| phosphoinositide-3-kinase regulatory subunit 5 | PIK3R5 | 3,20E-09 | 4,04 |
| deltex E3 ubiquitin ligase 3L | DTX3L | 1,01E-07 | 4,039 |
| AT-rich interaction domain 5A | ARID5A | 6,03E-05 | 4,038 |
| interferon induced transmembrane protein 3 | IFITM3 | 8,13E-10 | 4,026 |
| podocan like 1 | PODNL1 | 1,01E-08 | 4,006 |
| cyclin F | CCNF | 8,68E-10 | 3,992 |
| early growth response 2 | EGR2 | 4,23E-07 | 3,991 |
| Ras and Rab interactor like | RINL | 1,80E-08 | 3,98 |
| dedicator of cytokinesis 2 | DOCK2 | 4,70E-10 | 3,964 |
| AT-hook transcription factor | AKNA | 1,82E-09 | 3,942 |
| activating transcription factor 3 | ATF3 | 1,04E-04 | 3,935 |
| high mobility group box 2 | HMGB2 | 7,93E-06 | 3,927 |
| T cell receptor beta, variable 13-2 | Trbv13-2 | 6,16E-10 | 3,926 |
| TRAF interacting protein with forkhead associated domain | TIFA | 1,21E-05 | 3,921 |
| Rho GTPase activating protein 30 | ARHGAP30 | 4,64E-08 | 3,918 |
| lymphocyte antigen 6 complex pseudogene | 9030619P08Rik | 1,54E-09 | 3,909 |
| transmembrane protein 106A | TMEM106A | 4,21E-07 | 3,905 |
| serpin family G member 1 | SERPING1 | 3,33E-10 | 3,886 |
| gasdermin D | GSDMD | 2,84E-11 | 3,881 |
| acyloxyacyl hydrolase | AOAH | 1,31E-07 | 3,875 |
| DNA damage inducible transcript 3 | DDIT3 | 7,52E-10 | 3,873 |
| F-box and WD-40 domain protein 17 | Fbxw17 | 9,85E-10 | 3,854 |
| adenosine deaminase, RNA specific | ADAR | 1,41E-10 | 3,844 |
| kelch repeat and BTB domain containing 11 | KBTBD11 | 6,09E-08 | 3,838 |
| proteasome activator subunit 1 | PSME1 | 6,53E-11 | 3,82 |
| thromboxane A synthase 1 | TBXAS1 | 4,22E-08 | 3,818 |
| chromosome 16 open reading frame 54 | C16orf54 | 4,47E-09 | 3,815 |
| formyl peptide receptor 1 | FPR1 | 1,27E-06 | 3,812 |
| ubiquitin associated and SH3 domain containing B | UBASH3B | 3,41E-06 | 3,812 |
| histone cluster 2 H2B family member f | HIST2H2BF | 1,00E-06 | 3,808 |
| minichromosome maintenance complex component 6 | MCM6 | 2,92E-10 | 3,795 |
| plasminogen activator, urokinase receptor | PLAUR | 1,31E-06 | 3,795 |
| toll-like receptor 13 | Tlr13 | 7,48E-07 | 3,782 |
| unc-93 homolog B1 (C. elegans) | UNC93B1 | 1,24E-08 | 3,781 |
| component of Sp100-rs | Csprs (includes others) | 3,11E-05 | 3,778 |
| interleukin 6 | IL6 | 1,20E-04 | 3,774 |
| aurora kinase A | AURKA | 2,85E-07 | 3,772 |
| BCL2 antagonist/killer 1 | BAK1 | 6,01E-09 | 3,771 |
| TNF alpha induced protein 2 | TNFAIP2 | 2,60E-06 | 3,759 |
| macrophage scavenger receptor 1 | MSR1 | 8,84E-09 | 3,756 |
| nucleolar and spindle associated protein 1 | NUSAP1 | 5,88E-09 | 3,754 |
| tetraspanin 32 | TSPAN32 | 1,57E-08 | 3,747 |
| NLR family, pyrin domain containing 1A | Nlrp1a | 6,01E-09 | 3,74 |
| glycine amidinotransferase | GATM | 3,17E-07 | 3,734 |
| beta-ureidopropionase 1 | UPB1 | 5,96E-09 | 3,713 |
| apolipoprotein L 11a | Apol10a (includes others) | 3,58E-08 | 3,68 |
| T cell receptor alpha constant | Trac | 8,80E-10 | 3,68 |
| G-protein signaling modulator 3 | GPSM3 | 2,07E-08 | 3,678 |
| arginase 1 | ARG1 | 2,22E-02 | 3,677 |
| linker for activation of T-cells family member 2 | LAT2 | 1,78E-07 | 3,676 |
| SH3 domain binding glutamate rich protein like 3 | SH3BGRL3 | 9,59E-09 | 3,671 |
| shugoshin 1 | SGO1 | 1,24E-08 | 3,666 |
| C-type lectin domain family 9 member A | CLEC9A | 7,04E-07 | 3,662 |
| SAM domain, SH3 domain and nuclear localization signals 1 | SAMSN1 | 2,49E-07 | 3,655 |
| histocompatibility 2, T region locus 24 | H2-T24 | 9,17E-10 | 3,653 |
| SHC binding and spindle associated 1 | SHCBP1 | 1,59E-07 | 3,64 |
| microsomal glutathione S-transferase 2 | MGST2 | 4,71E-08 | 3,63 |
| myeloid differentiation primary response 88 | MYD88 | 3,06E-09 | 3,628 |
| transforming acidic coiled-coil containing protein 3 | TACC3 | 1,95E-07 | 3,61 |
| solute carrier family 39 member 4 | SLC39A4 | 5,88E-08 | 3,609 |
| mab-21 like 3 | MAB21L3 | 1,40E-08 | 3,607 |
| G protein-coupled receptor 18 | GPR18 | 4,16E-09 | 3,603 |
| ATPase phospholipid transporting 8B4 (putative) | ATP8B4 | 4,38E-08 | 3,602 |
| CD86 molecule | CD86 | 2,47E-09 | 3,593 |
| complement C1r | C1R | 1,64E-06 | 3,585 |
| CD84 molecule | CD84 | 1,40E-05 | 3,574 |
| histone cluster 1 H2A family member a | HIST1H2AA | 8,63E-10 | 3,565 |
| dipeptidase 2 | DPEP2 | 1,54E-05 | 3,562 |
| selectin L | SELL | 4,66E-04 | 3,541 |
| EF-hand domain family member D2 | EFHD2 | 1,45E-07 | 3,533 |
| peptidyl arginine deiminase 2 | PADI2 | 6,69E-09 | 3,53 |
| chromatin licensing and DNA replication factor 1 | CDT1 | 3,87E-05 | 3,526 |
| histone cluster 1 H2A family member h | HIST1H2AH | 1,35E-09 | 3,517 |
| abnormal spindle microtubule assembly | ASPM | 2,76E-08 | 3,515 |
| actin beta | ACTB | 1,35E-08 | 3,496 |
| dedicator of cytokinesis 10 | DOCK10 | 2,14E-08 | 3,494 |
| brain abundant membrane attached signal protein 1 | BASP1 | 1,16E-06 | 3,486 |
| F-box protein 5 | FBXO5 | 6,40E-09 | 3,48 |
| lymphocyte cytosolic protein 2 | LCP2 | 1,02E-06 | 3,477 |
| zinc finger CCCH-type containing 12D | ZC3H12D | 1,40E-06 | 3,475 |
| E2F transcription factor 1 | E2F1 | 6,94E-07 | 3,473 |
| pyrophosphatase (inorganic) 1 | PPA1 | 7,84E-08 | 3,452 |
| CD96 molecule | CD96 | 1,45E-09 | 3,431 |
| syntaxin binding protein 2 | STXBP2 | 8,66E-08 | 3,43 |
| Lck interacting transmembrane adaptor 1 | LIME1 | 4,66E-08 | 3,429 |
| shisa family member 5 | SHISA5 | 6,79E-10 | 3,427 |
| haptoglobin | HP | 9,40E-03 | 3,415 |
| 2'-5'-oligoadenylate synthetase 2 | OAS2 | 8,94E-07 | 3,41 |
| capping protein (actin filament), gelsolin-like pseudogene | 5730416F02Rik | 3,79E-08 | 3,403 |
| acyl-CoA synthetase long-chain family member 5 | ACSL5 | 1,17E-10 | 3,4 |
| neutrophil cytosolic factor 1 | NCF1 | 9,86E-10 | 3,391 |
| indoleamine 2,3-dioxygenase 1 | IDO1 | 1,06E-05 | 3,387 |
| FYVE, RhoGEF and PH domain containing 3 | FGD3 | 3,84E-07 | 3,384 |
| predicted gene 5150 | Gm5150 | 7,85E-07 | 3,382 |
| methylenetetrahydrofolate dehydrogenase (NADP+ dependent) 1-like | MTHFD1L | 1,24E-05 | 3,381 |
| DNA damage regulated autophagy modulator 1 | DRAM1 | 2,43E-07 | 3,375 |
| matrix metallopeptidase 25 | MMP25 | 1,21E-08 | 3,372 |
| Fc fragment of IgG receptor IIa | FCGR2A | 1,15E-06 | 3,367 |
| minichromosome maintenance complex component 3 | MCM3 | 6,91E-08 | 3,357 |
| translocator protein | TSPO | 3,22E-09 | 3,353 |
| transmembrane protein 229B | TMEM229B | 1,78E-07 | 3,35 |
| Rho GTPase activating protein 9 | ARHGAP9 | 7,63E-08 | 3,349 |
| non-SMC condensin I complex subunit H | NCAPH | 1,05E-05 | 3,348 |
| interferon stimulated exonuclease gene 20 | ISG20 | 9,81E-07 | 3,33 |
| serum amyloid A1 | SAA1 | 1,39E-02 | 3,322 |
| B-cell CLL/lymphoma 11B | BCL11B | 4,63E-09 | 3,321 |
| RNA binding motif protein 43 | RBM43 | 2,53E-10 | 3,319 |
| cathepsin Z | CTSZ | 5,63E-08 | 3,304 |
| insulin like 6 | INSL6 | 8,09E-07 | 3,292 |
| G protein-coupled receptor 84 | GPR84 | 3,48E-05 | 3,29 |
| family with sequence similarity 167 member B | FAM167B | 4,76E-06 | 3,288 |
| phosphatidylinositol-3,4,5-trisphosphate dependent Rac exchange factor 1 | PREX1 | 3,15E-07 | 3,271 |
| hepatitis A virus cellular receptor 2 | HAVCR2 | 3,84E-07 | 3,269 |
| transmembrane and tetratricopeptide repeat containing 4 | TMTC4 | 1,93E-09 | 3,269 |
| tripartite motif containing 59 | TRIM59 | 5,37E-08 | 3,262 |
| inositol polyphosphate-5-phosphatase D | INPP5D | 4,57E-08 | 3,252 |
| Src like adaptor 2 | SLA2 | 4,42E-08 | 3,251 |
| napsin A aspartic peptidase | NAPSA | 2,61E-07 | 3,25 |
| predicted gene 10134 | Gm10134 | 1,35E-07 | 3,233 |
| anti-silencing function 1B histone chaperone | ASF1B | 5,50E-08 | 3,23 |
| programmed cell death 1 | PDCD1 | 4,80E-08 | 3,23 |
| schlafen 1 | Slfn1 | 2,49E-07 | 3,228 |
| cyclin E1 | CCNE1 | 3,73E-06 | 3,212 |
| purinergic receptor P2X 7 | P2RX7 | 2,25E-06 | 3,202 |
| phosphatase and actin regulator 1 | PHACTR1 | 1,58E-08 | 3,193 |
| SH2 domain containing 2A | SH2D2A | 4,45E-06 | 3,192 |
| WDFY family member 4 | WDFY4 | 2,24E-06 | 3,187 |
| Bruton tyrosine kinase | BTK | 9,34E-09 | 3,176 |
| NFAT activating protein with ITAM motif 1 | NFAM1 | 1,28E-05 | 3,167 |
| proline rich 5 like | PRR5L | 3,98E-06 | 3,158 |
| family with sequence similarity 107 member B | FAM107B | 3,62E-08 | 3,147 |
| epithelial membrane protein 3 | EMP3 | 3,47E-06 | 3,145 |
| adaptor related protein complex 1 sigma 3 subunit | AP1S3 | 2,49E-09 | 3,144 |
| minichromosome maintenance 10 replication initiation factor | MCM10 | 1,26E-07 | 3,144 |
| membrane spanning 4-domains A8 | MS4A8 | 4,65E-04 | 3,144 |
| lymphocyte antigen 6 complex, locus E | LY6E | 7,47E-09 | 3,13 |
| interleukin 4 receptor | IL4R | 2,02E-07 | 3,123 |
| histone cluster 1 H2A family member j | HIST1H2AJ | 4,93E-09 | 3,114 |
| lymphocyte antigen 6 complex, locus A | Ly6a (includes others) | 7,00E-08 | 3,113 |
| inner centromere protein | INCENP | 7,84E-08 | 3,108 |
| RAD51 recombinase | RAD51 | 1,03E-08 | 3,086 |
| ADP ribosylation factor like GTPase 5C | ARL5C | 1,14E-04 | 3,08 |
| purinergic receptor P2Y10 | P2RY10 | 3,57E-08 | 3,08 |
| src kinase associated phosphoprotein 2 | SKAP2 | 2,83E-07 | 3,076 |
| aldehyde dehydrogenase 1 family member A2 | ALDH1A2 | 9,31E-05 | 3,075 |
| ATP binding cassette subfamily G member 1 | ABCG1 | 1,88E-06 | 3,072 |
| thyroid hormone receptor interactor 13 | TRIP13 | 1,31E-06 | 3,072 |
| family with sequence similarity 105 member A | FAM105A | 1,06E-06 | 3,07 |
| Fanconi anemia complementation group A | FANCA | 1,59E-06 | 3,068 |
| structural maintenance of chromosomes 2 | SMC2 | 7,01E-07 | 3,059 |
| C-X-C motif chemokine ligand 2 | CXCL2 | 2,67E-05 | 3,057 |
| TNF alpha induced protein 8 like 2 | TNFAIP8L2 | 1,26E-07 | 3,056 |
| phospholipase A2 group VII | PLA2G7 | 5,61E-05 | 3,054 |
| p21 (RAC1) activated kinase 1 | PAK1 | 1,45E-08 | 3,05 |
| chromosome 14 open reading frame 80 | C14orf80 | 1,20E-08 | 3,041 |
| histone cluster 2 H2A family member c | HIST2H2AC | 2,13E-09 | 3,041 |
| killer cell lectin-like receptor, subfamily A, member 4 | Klra7 (includes others) | 4,14E-08 | 3,041 |
| phospholipase A2 group IVA | PLA2G4A | 8,33E-07 | 3,036 |
| C-type lectin domain family 2, member d | Clec2d (includes others) | 1,84E-03 | 3,035 |
| torsin family 3 member A | TOR3A | 1,31E-08 | 3,031 |
| tripartite motif containing 14 | TRIM14 | 2,39E-07 | 3,03 |
| protamine 1 | Prm1 | 2,97E-04 | 3,018 |
| tropomyosin 3 | TPM3 | 2,28E-08 | 3,018 |
| runt related transcription factor 1 | RUNX1 | 5,22E-04 | 3,017 |
| spindle and kinetochore associated complex subunit 1 | SKA1 | 1,19E-06 | 3,014 |
| protein tyrosine kinase 2 beta | PTK2B | 1,44E-06 | 3,012 |
| arachidonate 5-lipoxygenase activating protein | ALOX5AP | 5,41E-07 | 3,009 |
| colony stimulating factor 2 receptor alpha subunit | CSF2RA | 5,63E-08 | 3,008 |
| strawberry notch homolog 2 | SBNO2 | 3,16E-07 | 3,005 |
| MARCKS like 1 | MARCKSL1 | 1,74E-05 | 3,003 |
| toll like receptor 3 | TLR3 | 1,28E-09 | 2,997 |
| cell division cycle 20 | CDC20 | 1,07E-06 | 2,994 |
| sterile alpha motif domain containing 9 like | SAMD9L | 1,59E-06 | 2,994 |
| 2-cell-stage, variable group, member 3 | Tcstv3 | 2,06E-07 | 2,991 |
| arrestin beta 2 | ARRB2 | 5,95E-07 | 2,988 |
| lamin B1 | LMNB1 | 9,00E-06 | 2,981 |
| cysteinyl leukotriene receptor 2 | CYSLTR2 | 1,93E-07 | 2,977 |
| GRB2-related adaptor protein 2 | GRAP2 | 5,29E-07 | 2,972 |
| C-type lectin domain family 6 member A | CLEC6A | 1,00E-05 | 2,97 |
| T cell receptor beta, variable 16 | Trbv16 | 4,09E-05 | 2,968 |
| Mov10 RISC complex RNA helicase | MOV10 | 6,35E-07 | 2,964 |
| C-type lectin domain family 10 member A | CLEC10A | 3,46E-08 | 2,957 |
| lymphocyte antigen 9 | LY9 | 1,83E-05 | 2,954 |
| interleukin 18 receptor accessory protein | IL18RAP | 2,77E-05 | 2,952 |
| chloride intracellular channel 1 | CLIC1 | 3,52E-06 | 2,946 |
| adenylate cyclase associated protein 1 | CAP1 | 3,22E-07 | 2,927 |
| GTPase, IMAP family member 9 | Gimap9 | 8,70E-06 | 2,923 |
| aldolase, fructose-bisphosphate C | ALDOC | 3,15E-06 | 2,922 |
| lipopolysaccharide induced TNF factor | LITAF | 1,83E-07 | 2,911 |
| galectin 9B | LGALS9B | 9,38E-08 | 2,91 |
| poly(ADP-ribose) polymerase family member 3 | PARP3 | 1,17E-06 | 2,908 |
| C-X3-C motif chemokine ligand 1 | CX3CL1 | 6,07E-06 | 2,892 |
| PYD and CARD domain containing | PYCARD | 3,10E-08 | 2,891 |
| ETS variant 6 | ETV6 | 3,03E-08 | 2,877 |
| carcinoembryonic antigen related cell adhesion molecule 16 | CEACAM16 | 2,83E-08 | 2,87 |
| SERTA domain containing 2 | SERTAD2 | 1,44E-04 | 2,869 |
| mitochondrial calcium uniporter dominant negative beta subunit | MCUB | 3,06E-06 | 2,863 |
| T cell receptor beta, variable 5 | Trbv5 | 4,96E-07 | 2,863 |
| signaling lymphocytic activation molecule family member 1 | SLAMF1 | 4,14E-08 | 2,857 |
| actin related protein 2/3 complex subunit 1B | ARPC1B | 3,17E-07 | 2,854 |
| von Willebrand factor A domain containing 5A | VWA5A | 5,89E-10 | 2,854 |
| embigin | EMB | 5,20E-03 | 2,852 |
| interferon regulatory factor 9 | IRF9 | 2,83E-08 | 2,839 |
| docking protein 1 | DOK1 | 8,61E-07 | 2,837 |
| cytochrome b-245 alpha chain | CYBA | 5,61E-08 | 2,834 |
| DEP domain containing 1B | DEPDC1B | 8,94E-08 | 2,831 |
| ring finger protein 31 | RNF31 | 2,17E-08 | 2,829 |
| cytokine receptor-like factor 2 | CRLF2 | 3,39E-07 | 2,827 |
| peptidoglycan recognition protein 2 | PGLYRP2 | 3,40E-08 | 2,827 |
| Rho GTPase activating protein 15 | ARHGAP15 | 1,84E-05 | 2,826 |
| pyrimidinergic receptor P2Y6 | P2RY6 | 2,46E-06 | 2,823 |
| C-C motif chemokine ligand 1 | CCL1 | 2,57E-05 | 2,819 |
| killer cell lectin-like receptor subfamily A, member 23 | Klra23 | 4,47E-08 | 2,814 |
| ribosomal protein S6 kinase A1 | RPS6KA1 | 3,03E-08 | 2,805 |
| extra spindle pole bodies like 1, separase | ESPL1 | 2,67E-09 | 2,802 |
| family with sequence similarity 111 member A | FAM111A | 3,93E-05 | 2,8 |
| apolipoprotein L 7e | Apol7e (includes others) | 1,11E-05 | 2,796 |
| interferon gamma inducible protein 16 | IFI16 | 1,22E-04 | 2,794 |
| serpin family B member 9 | SERPINB9 | 3,38E-07 | 2,794 |
| fucosyltransferase 7 | FUT7 | 3,16E-07 | 2,79 |
| opioid growth factor receptor | OGFR | 6,10E-10 | 2,785 |
| C-type lectin domain family 4 member C | CLEC4C | 5,04E-05 | 2,783 |
| serglycin | Srgn | 3,65E-06 | 2,773 |
| sperm associated antigen 5 | SPAG5 | 7,64E-07 | 2,769 |
| T-cell immune regulator 1, ATPase H+ transporting V0 subunit a3 | TCIRG1 | 5,99E-07 | 2,767 |
| aldehyde dehydrogenase 3 family member B1 | ALDH3B1 | 7,21E-07 | 2,765 |
| helicase with zinc finger 2 | HELZ2 | 2,55E-07 | 2,765 |
| chromosome 19 open reading frame 38 | C19orf38 | 9,16E-05 | 2,76 |
| receptor interacting serine/threonine kinase 1 | RIPK1 | 3,84E-09 | 2,757 |
| heat shock protein family A (Hsp70) member 1A | HSPA1A/HSPA1B | 2,33E-02 | 2,755 |
| adenylate cyclase 7 | ADCY7 | 1,84E-04 | 2,75 |
| zinc finger NFX1-type containing 1 | ZNFX1 | 7,29E-09 | 2,746 |
| leukocyte specific transcript 1 | Lst1 | 1,94E-05 | 2,737 |
| POU class 2 homeobox 2 | POU2F2 | 1,41E-05 | 2,735 |
| protein kinase C beta | PRKCB | 1,46E-06 | 2,733 |
| ankyrin repeat domain 1 | ANKRD1 | 6,69E-04 | 2,73 |
| kinesin family member C1 | KIFC1 | 1,50E-05 | 2,727 |
| POU domain, class 3, transcription factor 1 | Pou3f1 | 6,15E-06 | 2,717 |
| solute carrier family 13 member 3 | SLC13A3 | 2,53E-06 | 2,716 |
| microtubule interacting and trafficking domain containing 1 | MITD1 | 2,45E-07 | 2,715 |
| cyclin dependent kinase 1 | CDK1 | 1,01E-06 | 2,705 |
| caspase 8 | CASP8 | 5,76E-09 | 2,698 |
| TXK tyrosine kinase | TXK | 2,30E-05 | 2,695 |
| C-type lectin domain family 7 member A | CLEC7A | 2,30E-03 | 2,688 |
| solute carrier family 7 member 7 | SLC7A7 | 1,32E-07 | 2,685 |
| signaling threshold regulating transmembrane adaptor 1 | SIT1 | 1,87E-08 | 2,679 |
| cofilin 1 | CFL1 | 1,26E-05 | 2,678 |
| interleukin 27 receptor subunit alpha | IL27RA | 3,27E-05 | 2,678 |
| T cell receptor beta, variable 13-3 | Trbv13-3 | 2,65E-04 | 2,677 |
| predicted gene 6377 | Gm6377 | 6,39E-07 | 2,676 |
| inositol polyphosphate-1-phosphatase | INPP1 | 5,01E-08 | 2,673 |
| 3-phosphoglycerate dehydrogenase pseudogene | Gm8096 | 1,27E-06 | 2,667 |
| tubulin beta 2B class IIb | TUBB2B | 1,06E-04 | 2,641 |
| mucolipin 2 | MCOLN2 | 3,04E-04 | 2,637 |
| vimentin | VIM | 3,32E-05 | 2,636 |
| G protein-coupled receptor 174 | GPR174 | 7,59E-08 | 2,634 |
| apolipoprotein B mRNA editing enzyme catalytic subunit 1 | APOBEC1 | 1,30E-04 | 2,633 |
| drebrin like | DBNL | 2,46E-08 | 2,633 |
| guanine deaminase | GDA | 1,22E-04 | 2,628 |
| interferon induced transmembrane protein 1 | Ifitm1 | 3,85E-04 | 2,606 |
| RNA binding motif protein 47 | RBM47 | 5,30E-06 | 2,605 |
| endoplasmic reticulum aminopeptidase 1 | ERAP1 | 3,89E-09 | 2,604 |
| thymidylate synthase, pseudogene | Tyms-ps | 1,32E-06 | 2,603 |
| regulator of chromosome condensation 2 | RCC2 | 8,57E-07 | 2,6 |
| complement C5a receptor 1 | C5AR1 | 1,48E-06 | 2,597 |
| neutrophil cytosolic factor 2 | NCF2 | 1,79E-05 | 2,571 |
| dedicator of cytokinesis 11 | DOCK11 | 7,90E-07 | 2,563 |
| transient receptor potential cation channel subfamily V member 2 | TRPV2 | 2,22E-07 | 2,558 |
| GEM interacting protein | GMIP | 1,17E-06 | 2,554 |
| major vault protein | MVP | 3,09E-06 | 2,553 |
| zinc finger CCCH-type containing, antiviral 1 | ZC3HAV1 | 3,46E-05 | 2,553 |
| CD200 receptor 1 like | CD200R1L | 1,89E-05 | 2,549 |
| CD247 molecule | CD247 | 5,80E-07 | 2,548 |
| immunoglobulin superfamily member 6 | IGSF6 | 2,49E-04 | 2,545 |
| germ cell associated 2, haspin | GSG2 | 1,31E-08 | 2,544 |
| RAB43, member RAS oncogene family | RAB43 | 2,49E-05 | 2,542 |
| neurofilament heavy polypeptide | NEFH | 6,84E-07 | 2,54 |
| SLC9A3 regulator 1 | SLC9A3R1 | 2,88E-06 | 2,54 |
| meiosis specific nuclear structural 1 | MNS1 | 4,72E-07 | 2,528 |
| PDZ binding kinase | PBK | 8,13E-08 | 2,523 |
| NLR family apoptosis inhibitory protein | NAIP | 1,87E-06 | 2,514 |
| glucose-6-phosphate dehydrogenase | G6PD | 9,34E-09 | 2,508 |
| pleckstrin homology domain containing O2 | PLEKHO2 | 3,75E-06 | 2,508 |
| 2'-5' oligoadenylate synthetase 1D | Oas1d (includes others) | 3,29E-06 | 2,507 |
| regulator of G-protein signaling 19 | RGS19 | 1,96E-05 | 2,504 |
| transgelin 2 | TAGLN2 | 6,19E-06 | 2,496 |
| janus kinase and microtubule interacting protein 1 | JAKMIP1 | 7,55E-07 | 2,49 |
| ATPase phospholipid transporting 10A (putative) | ATP10A | 5,55E-08 | 2,488 |
| IQ motif containing GTPase activating protein 1 | IQGAP1 | 1,40E-06 | 2,488 |
| thymosin, beta 4, X chromosome | Tmsb4x (includes others) | 1,49E-06 | 2,483 |
| kinesin family member 20A | KIF20A | 1,14E-06 | 2,478 |
| SH3 domain binding protein 1 | SH3BP1 | 1,87E-05 | 2,477 |
| CD300 molecule like family member d | CD300LD | 5,57E-05 | 2,474 |
| C-C motif chemokine receptor 7 | CCR7 | 5,38E-05 | 2,469 |
| phosphofructokinase, platelet | PFKP | 9,19E-09 | 2,467 |
| nucleotide binding oligomerization domain containing 1 | NOD1 | 9,68E-08 | 2,46 |
| sphingomyelin phosphodiesterase acid like 3B | SMPDL3B | 5,73E-05 | 2,459 |
| beta-1,4-galactosyltransferase 5 | B4GALT5 | 8,34E-06 | 2,457 |
| BUB1 mitotic checkpoint serine/threonine kinase B | BUB1B | 8,42E-07 | 2,451 |
| centromere protein H | CENPH | 5,78E-06 | 2,451 |
| paired immunoglobin-like type 2 receptor beta | PILRB | 1,32E-06 | 2,449 |
| synaptonemal complex central element protein 2 | SYCE2 | 1,46E-05 | 2,447 |
| cell adhesion molecule 1 | CADM1 | 4,59E-07 | 2,445 |
| centromere protein K | CENPK | 1,27E-06 | 2,443 |
| primase (DNA) subunit 1 | PRIM1 | 7,63E-08 | 2,443 |
| endothelin converting enzyme 2 | ECE2 | 2,00E-06 | 2,439 |
| DNA ligase 1 | LIG1 | 1,03E-06 | 2,438 |
| protein kinase C theta | PRKCQ | 4,81E-09 | 2,437 |
| interleukin 3 receptor subunit alpha | IL3RA | 7,54E-06 | 2,432 |
| DEF6, guanine nucleotide exchange factor | DEF6 | 1,14E-06 | 2,43 |
| lymphocyte-specific protein 1 | LSP1 | 2,30E-07 | 2,428 |
| phospholipase C gamma 2 | PLCG2 | 3,27E-06 | 2,421 |
| S100 calcium binding protein A6 | S100A6 | 2,69E-06 | 2,42 |
| annexin A2 | ANXA2 | 7,16E-07 | 2,412 |
| killer cell lectin like receptor B1 | KLRB1 | 9,66E-07 | 2,412 |
| nerve growth factor receptor | NGFR | 2,57E-06 | 2,408 |
| solute carrier family 2 member 3 | SLC2A3 | 6,67E-06 | 2,408 |
| adenosine deaminase | ADA | 2,51E-05 | 2,407 |
| cell division cycle associated 2 | CDCA2 | 8,89E-07 | 2,406 |
| CNDP dipeptidase 2 (metallopeptidase M20 family) | CNDP2 | 1,74E-06 | 2,404 |
| LFNG O-fucosylpeptide 3-beta-N-acetylglucosaminyltransferase | LFNG | 3,83E-06 | 2,399 |
| chromatin assembly factor 1 subunit B | CHAF1B | 4,83E-04 | 2,398 |
| contactin 5 | CNTN5 | 5,12E-05 | 2,395 |
| IKAROS family zinc finger 1 | IKZF1 | 1,54E-06 | 2,394 |
| exonuclease 1 | EXO1 | 9,72E-06 | 2,391 |
| collapsin response mediator protein 1 | CRMP1 | 5,56E-07 | 2,385 |
| cyclin dependent kinase inhibitor 2A | CDKN2A | 1,05E-05 | 2,38 |
| caspase 12 | Casp12 | 2,92E-06 | 2,379 |
| phosphoinositide-3-kinase adaptor protein 1 | PIK3AP1 | 6,97E-06 | 2,371 |
| tumor necrosis factor superfamily member 14 | TNFSF14 | 1,21E-06 | 2,364 |
| small G protein signaling modulator 2 | SGSM2 | 1,50E-05 | 2,363 |
| cell division cycle 25B | CDC25B | 5,95E-07 | 2,356 |
| TNF alpha induced protein 6 | TNFAIP6 | 2,49E-05 | 2,354 |
| phosphatidylinositol-4,5-bisphosphate 3-kinase catalytic subunit gamma | PIK3CG | 5,42E-07 | 2,349 |
| apolipoprotein D | APOD | 7,54E-04 | 2,348 |
| ADP ribosylation factor like GTPase 6 interacting protein 1 | ARL6IP1 | 2,63E-08 | 2,348 |
| three prime repair exonuclease 1 | TREX1 | 7,92E-07 | 2,346 |
| UDP glucuronosyltransferase family 1 member A6 | UGT1A6 | 4,51E-03 | 2,345 |
| granulin precursor | GRN | 2,85E-06 | 2,343 |
| thymocyte selection associated | THEMIS | 4,64E-06 | 2,341 |
| kinetochore associated 1 | KNTC1 | 1,09E-05 | 2,339 |
| GATA binding protein 3 | GATA3 | 9,42E-06 | 2,336 |
| ER degradation enhancing alpha-mannosidase like protein 1 | EDEM1 | 2,71E-05 | 2,333 |
| G protein subunit alpha 13 | GNA13 | 7,20E-07 | 2,333 |
| syndecan 3 | SDC3 | 6,12E-07 | 2,331 |
| chromosome 17 open reading frame 62 | C17orf62 | 2,01E-05 | 2,33 |
| selectin P | SELP | 1,77E-03 | 2,329 |
| zinc finger and BTB domain containing 5 | ZBTB5 | 2,09E-05 | 2,325 |
| inhibitor of kappa light polypeptide gene enhancer in B-cells, kinase epsilon | IKBKE | 1,19E-06 | 2,322 |
| secreted and transmembrane 1 | SECTM1 | 2,80E-07 | 2,322 |
| lysozyme | LYZ | 7,45E-04 | 2,321 |
| sphingosine-1-phosphate receptor 4 | S1PR4 | 1,57E-04 | 2,321 |
| growth differentiation factor 15 | GDF15 | 9,91E-03 | 2,32 |
| inositol-trisphosphate 3-kinase A | ITPKA | 1,39E-08 | 2,32 |
| glutaredoxin | GLRX | 5,57E-05 | 2,313 |
| bromodomain adjacent to zinc finger domain 1A | BAZ1A | 6,04E-08 | 2,31 |
| death domain associated protein | DAXX | 7,48E-06 | 2,309 |
| serine carboxypeptidase 1 | SCPEP1 | 1,04E-06 | 2,307 |
| tumor necrosis factor superfamily member 13b | TNFSF13B | 8,51E-06 | 2,307 |
| RELB proto-oncogene, NF-kB subunit | RELB | 9,13E-06 | 2,305 |
| killer cell lectin-like receptor subfamily B member 1F | Klrb1f | 5,02E-05 | 2,304 |
| potassium two pore domain channel subfamily K member 5 | KCNK5 | 1,13E-05 | 2,302 |
| protein kinase, membrane associated tyrosine/threonine 1 | PKMYT1 | 2,46E-06 | 2,3 |
| oncostatin M receptor | OSMR | 1,57E-05 | 2,295 |
| T cell receptor beta, variable 10 | Trbv4 | 2,93E-04 | 2,293 |
| multiple C2 and transmembrane domain containing 2 | MCTP2 | 5,82E-04 | 2,292 |
| prostaglandin-endoperoxide synthase 2 | PTGS2 | 9,56E-05 | 2,291 |
| ADAM metallopeptidase with thrombospondin type 1 motif 4 | ADAMTS4 | 7,10E-05 | 2,287 |
| transmembrane protein 51 | TMEM51 | 8,05E-05 | 2,281 |
| hydrogen voltage gated channel 1 | HVCN1 | 4,40E-04 | 2,279 |
| CASP8 and FADD like apoptosis regulator | CFLAR | 3,95E-04 | 2,272 |
| Pim-1 proto-oncogene, serine/threonine kinase | PIM1 | 5,75E-04 | 2,272 |
| complement C3a receptor 1 | C3AR1 | 1,68E-06 | 2,269 |
| nuclear protein 1, transcriptional regulator | NUPR1 | 5,45E-04 | 2,266 |
| protein kinase C delta | PRKCD | 1,55E-06 | 2,266 |
| sterol O-acyltransferase 2 | SOAT2 | 9,39E-07 | 2,266 |
| actin related protein 2/3 complex subunit 5 | ARPC5 | 8,11E-06 | 2,265 |
| BRCA1 associated RING domain 1 | BARD1 | 1,02E-06 | 2,265 |
| PRELI domain containing 1 | PRELID1 | 3,10E-05 | 2,259 |
| RAB8B, member RAS oncogene family | RAB8B | 2,17E-06 | 2,251 |
| stress associated endoplasmic reticulum protein 1 | SERP1 | 4,12E-05 | 2,25 |
| protein tyrosine phosphatase, receptor type E | PTPRE | 5,08E-05 | 2,248 |
| PQ loop repeat containing 3 | PQLC3 | 6,36E-05 | 2,247 |
| interleukin 17 receptor A | IL17RA | 8,72E-04 | 2,246 |
| T-cell immunoglobulin and mucin domain containing 4 | TIMD4 | 6,33E-05 | 2,242 |
| maternal embryonic leucine zipper kinase | MELK | 2,15E-06 | 2,239 |
| chemokine like factor | CKLF | 7,85E-07 | 2,233 |
| cyclin D1 | CCND1 | 3,18E-04 | 2,231 |
| profilin 1 | PFN1 | 2,95E-05 | 2,231 |
| angiopoietin 2 | ANGPT2 | 1,04E-03 | 2,228 |
| NUF2, NDC80 kinetochore complex component | NUF2 | 1,43E-05 | 2,228 |
| serine/threonine kinase 17b | STK17B | 1,34E-04 | 2,222 |
| signal transducer and activator of transcription 3 | STAT3 | 6,60E-07 | 2,221 |
| T cell receptor beta, variable 29 | Trbv29 | 6,53E-03 | 2,22 |
| filamin A | FLNA | 3,85E-06 | 2,219 |
| proteasome maturation protein | POMP | 6,86E-09 | 2,219 |
| alanyl aminopeptidase, membrane | ANPEP | 2,50E-04 | 2,218 |
| tumor necrosis factor (ligand) superfamily, member 9 | Tnfsf9 | 2,15E-06 | 2,218 |
| TRAF-type zinc finger domain containing 1 | TRAFD1 | 4,20E-05 | 2,218 |
| RAS p21 protein activator 4 | RASA4 | 2,91E-06 | 2,216 |
| TNF receptor superfamily member 1B | TNFRSF1B | 1,84E-06 | 2,216 |
| interleukin 18 | IL18 | 4,73E-05 | 2,212 |
| ras homolog family member G | RHOG | 6,85E-07 | 2,211 |
| calponin 3 | CNN3 | 2,49E-07 | 2,207 |
| high mobility group AT-hook 2, pseudogene 1 | Hmga2-ps1 | 8,06E-05 | 2,204 |
| JunB proto-oncogene, AP-1 transcription factor subunit | JUNB | 1,82E-05 | 2,2 |
| thymidine kinase 1 | TK1 | 2,33E-04 | 2,2 |
| T cell receptor beta, variable 2 | Trbv2 | 1,59E-05 | 2,196 |
| chromosome X open reading frame 21 | CXorf21 | 2,80E-07 | 2,195 |
| nitric oxide synthase 2 | NOS2 | 8,40E-03 | 2,195 |
| TRAF3 interacting protein 3 | TRAF3IP3 | 4,76E-06 | 2,191 |
| serine peptidase inhibitor, Kunitz type 1 | SPINT1 | 9,39E-07 | 2,188 |
| potassium two pore domain channel subfamily K member 13 | KCNK13 | 1,84E-05 | 2,187 |
| polypeptide N-acetylgalactosaminyltransferase 12 | GALNT12 | 1,05E-07 | 2,183 |
| CD82 molecule | CD82 | 9,93E-06 | 2,178 |
| tubulin beta class I | TUBB | 9,54E-05 | 2,177 |
| MAF bZIP transcription factor | MAF | 8,27E-06 | 2,175 |
| actin gamma 1 | ACTG1 | 1,09E-03 | 2,174 |
| leucine rich repeat containing 4 | LRRC4 | 4,17E-05 | 2,174 |
| reticulocalbin 1 | RCN1 | 9,27E-06 | 2,174 |
| IKAROS family zinc finger 4 | IKZF4 | 8,29E-07 | 2,173 |
| poly(ADP-ribose) polymerase family member 11 | PARP11 | 8,88E-07 | 2,166 |
| cell division cycle associated 7 | CDCA7 | 2,31E-05 | 2,164 |
| RAB3A interacting protein like 1 | RAB3IL1 | 1,98E-04 | 2,162 |
| H2A histone family member J | H2AFJ | 1,79E-07 | 2,161 |
| keratin 85 | Krt85 | 1,68E-03 | 2,161 |
| signal regulatory protein alpha | SIRPA | 2,99E-04 | 2,158 |
| FES proto-oncogene, tyrosine kinase | FES | 4,04E-06 | 2,157 |
| membrane-spanning 4-domains, subfamily A, member 6B | Ms4a6b | 4,83E-05 | 2,156 |
| ubiquitin associated and SH3 domain containing A | UBASH3A | 9,72E-08 | 2,156 |
| killer cell lectin-like receptor family E member 1 | Klre1 | 6,08E-03 | 2,153 |
| BTG anti-proliferation factor 1 | BTG1 | 1,02E-04 | 2,15 |
| toll like receptor 6 | TLR6 | 3,04E-05 | 2,148 |
| annexin A4 | ANXA4 | 1,80E-06 | 2,147 |
| chloride intracellular channel 4 | CLIC4 | 2,30E-07 | 2,147 |
| ceruloplasmin | CP | 1,74E-05 | 2,146 |
| C-X-C motif chemokine ligand 13 | CXCL13 | 4,51E-03 | 2,146 |
| T-cell immunoreceptor with Ig and ITIM domains | TIGIT | 9,37E-04 | 2,146 |
| S100 calcium binding protein A11 | S100a11 | 2,03E-05 | 2,145 |
| T cell receptor beta, variable 12-2 | Trbv12-2 | 2,96E-06 | 2,145 |
| archaelysin family metallopeptidase 1 | AMZ1 | 1,34E-03 | 2,144 |
| eukaryotic translation initiation factor 4E family member 3 | EIF4E3 | 6,39E-07 | 2,144 |
| transketolase | TKT | 2,53E-06 | 2,142 |
| MAX dimerization protein 3 | MXD3 | 4,77E-06 | 2,14 |
| histone cluster 2 H2A family member a3 | HIST2H2AA3/HIST2H2AA4 | 6,12E-07 | 2,139 |
| Rho GTPase activating protein 8 | ARHGAP8/PRR5-ARHGAP8 | 5,60E-03 | 2,138 |
| ADP ribosylation factor 3 | ARF3 | 2,41E-05 | 2,135 |
| cytotoxic T-lymphocyte associated protein 4 | CTLA4 | 4,63E-04 | 2,133 |
| histocompatibility 2, M region locus 2 | H2-M2 | 5,86E-05 | 2,133 |
| PAS domain containing serine/threonine kinase | PASK | 6,53E-04 | 2,129 |
| B-box and SPRY domain containing | BSPRY | 3,27E-06 | 2,128 |
| nucleotide binding protein 1 | NUBP1 | 2,94E-06 | 2,126 |
| colony stimulating factor 1 | CSF1 | 6,17E-04 | 2,119 |
| lymphoid enhancer binding factor 1 | LEF1 | 4,44E-06 | 2,117 |
| tubulin alpha 1b | TUBA1B | 3,26E-03 | 2,117 |
| TGFB induced factor homeobox 1 | TGIF1 | 2,12E-05 | 2,115 |
| CDP-diacylglycerol synthase 1 | CDS1 | 1,22E-06 | 2,112 |
| CDC42 small effector 1 | CDC42SE1 | 1,45E-06 | 2,11 |
| NIMA related kinase 2 | NEK2 | 6,09E-06 | 2,108 |
| uncoupling protein 2 | UCP2 | 4,39E-03 | 2,106 |
| CD300c molecule | CD300C | 9,74E-05 | 2,104 |
| CDC28 protein kinase regulatory subunit 1B | CKS1B | 4,38E-05 | 2,104 |
| lysozyme | LYZ | 3,66E-04 | 2,101 |
| T-cell receptor alpha locus | TRA | 5,93E-06 | 2,098 |
| extended synaptotagmin 1 | ESYT1 | 1,17E-06 | 2,095 |
| dynactin subunit 6 | DCTN6 | 1,59E-07 | 2,094 |
| CD47 molecule | CD47 | 1,24E-05 | 2,093 |
| unc-5 family C-terminal like | UNC5CL | 5,26E-05 | 2,091 |
| T cell receptor beta, variable 13-1 | Trbv13-1 | 2,04E-03 | 2,089 |
| ABI family member 3 | ABI3 | 1,39E-03 | 2,083 |
| myelin associated glycoprotein | MAG | 8,89E-07 | 2,083 |
| CD226 molecule | CD226 | 2,86E-05 | 2,082 |
| LYN proto-oncogene, Src family tyrosine kinase | LYN | 1,03E-05 | 2,076 |
| chromosome 7 open reading frame 43 | C7orf43 | 7,20E-06 | 2,075 |
| apolipoprotein B mRNA editing enzyme catalytic subunit 3B | APOBEC3B | 5,35E-06 | 2,072 |
| granzyme K | GZMK | 1,42E-04 | 2,071 |
| legumain | LGMN | 2,59E-04 | 2,07 |
| GINS complex subunit 2 | GINS2 | 6,19E-05 | 2,065 |
| zinc finger CCCH-type containing, antiviral 1 | ZC3HAV1 | 6,39E-07 | 2,065 |
| kinesin family member 20B | KIF20B | 2,82E-05 | 2,059 |
| tropomyosin 4 | Tpm4 | 3,36E-04 | 2,059 |
| B and T lymphocyte associated | BTLA | 7,25E-06 | 2,057 |
| proteasome subunit beta 9 | PSMB9 | 4,43E-05 | 2,057 |
| reticulon 4 | RTN4 | 1,03E-04 | 2,053 |
| arachidonate 15-lipoxygenase | ALOX15 | 2,67E-05 | 2,052 |
| Ras association domain family member 2 | RASSF2 | 9,23E-04 | 2,052 |
| ribonuclease A family member k6 | RNASE6 | 6,79E-05 | 2,052 |
| T cell receptor beta, variable 15 | Trbv15 | 1,87E-02 | 2,051 |
| pleckstrin homology domain containing A4 | PLEKHA4 | 2,37E-05 | 2,05 |
| cytoskeleton associated protein 2 like | CKAP2L | 7,95E-06 | 2,049 |
| CD37 molecule | CD37 | 9,59E-05 | 2,047 |
| RAP1B, member of RAS oncogene family | RAP1B | 2,15E-05 | 2,046 |
| solute carrier organic anion transporter family member 4A1 | SLCO4A1 | 4,78E-05 | 2,046 |
| epithelial cell transforming 2 | ECT2 | 7,95E-06 | 2,045 |
| platelet activating factor receptor | PTAFR | 6,26E-05 | 2,042 |
| TNF receptor superfamily member 1A | TNFRSF1A | 1,61E-05 | 2,041 |
| SLAIN motif family member 1 | SLAIN1 | 1,74E-05 | 2,039 |
| SID1 transmembrane family member 1 | SIDT1 | 4,30E-06 | 2,038 |
| eukaryotic translation initiation factor 1A, X-linked | EIF1AX | 1,23E-05 | 2,037 |
| flap structure-specific endonuclease 1 | FEN1 | 1,81E-03 | 2,037 |
| histone cluster 1 H3 family member c | HIST1H3C | 4,37E-05 | 2,036 |
| IQ motif containing GTPase activating protein 3 | IQGAP3 | 1,64E-04 | 2,036 |
| TNF receptor superfamily member 11b | TNFRSF11B | 1,71E-04 | 2,036 |
| ARP3 actin related protein 3 homolog | ACTR3 | 6,94E-05 | 2,035 |
| kinesin family member 11 | KIF11 | 2,67E-05 | 2,035 |
| acyl-CoA synthetase medium-chain family member 5 | ACSM5 | 7,86E-04 | 2,034 |
| syntaxin binding protein 1 | STXBP1 | 1,61E-05 | 2,033 |
| synaptotagmin like 2 | SYTL2 | 2,99E-05 | 2,033 |
| proteasome subunit alpha 5 | PSMA5 | 1,16E-06 | 2,031 |
| transcriptional regulating factor 1 | TRERF1 | 4,81E-04 | 2,029 |
| GTPase, IMAP family member 8 | GIMAP8 | 2,24E-02 | 2,027 |
| bridging integrator 1 | BIN1 | 1,25E-04 | 2,019 |
| zwilch kinetochore protein | ZWILCH | 2,59E-05 | 2,015 |
| carbonic anhydrase 13 | CA13 | 3,90E-05 | 2,013 |
| LIM and SH3 protein 1 | LASP1 | 2,89E-04 | 2,013 |
| purinergic receptor P2Y13 | P2RY13 | 5,22E-05 | 2,012 |
| growth arrest and DNA damage inducible gamma | GADD45G | 3,31E-02 | 2,007 |
| ganglioside-induced differentiation-associated-protein 10 | Gdap10 | 1,20E-03 | 2,005 |
| inositol polyphosphate-5-phosphatase B | INPP5B | 2,74E-06 | 2,003 |
| interleukin 10 | IL10 | 6,72E-05 | 2,002 |
| olfactomedin 1 | OLFM1 | 9,00E-05 | 2,002 |
| interaction protein for cytohesin exchange factors 1 | IPCEF1 | 2,86E-05 | 2,001 |
| KIAA1456 | KIAA1456 | 1,58E-06 | -2,001 |
| transferrin | TF | 6,82E-05 | -2,001 |
| carbohydrate sulfotransferase 1 | CHST1 | 6,01E-05 | -2,005 |
| insulin like growth factor binding protein 6 | IGFBP6 | 3,61E-05 | -2,005 |
| C-type lectin domain family 18 member B | CLEC18B | 1,28E-02 | -2,01 |
| pleckstrin homology and RhoGEF domain containing G5 | PLEKHG5 | 4,94E-04 | -2,016 |
| potassium voltage-gated channel subfamily H member 2 | KCNH2 | 2,40E-03 | -2,022 |
| cDNA sequence BC024137 | BC024137 | 3,97E-05 | -2,024 |
| peroxisomal membrane protein 4 | PXMP4 | 3,75E-06 | -2,025 |
| small nucleolar RNA, H/ACA box 74A | SNORA74A | 2,69E-03 | -2,025 |
| prostaglandin-endoperoxide synthase 1 | PTGS1 | 1,13E-03 | -2,027 |
| whirlin | WHRN | 2,72E-04 | -2,028 |
| solute carrier family 38 member 3 | SLC38A3 | 1,91E-04 | -2,031 |
| transcription elongation factor A like 7 | TCEAL7 | 3,04E-05 | -2,032 |
| 4-aminobutyrate aminotransferase | ABAT | 8,26E-05 | -2,033 |
| glycine C-acetyltransferase | GCAT | 1,88E-04 | -2,035 |
| family with sequence similarity 19 member A5, C-C motif chemokine like | FAM19A5 | 5,63E-06 | -2,038 |
| nitrilase 1 | NIT1 | 1,83E-02 | -2,038 |
| tetraspanin 18 | TSPAN18 | 1,33E-03 | -2,038 |
| heat shock factor binding protein 1 like 1 | HSBP1L1 | 4,93E-05 | -2,042 |
| sodium voltage-gated channel beta subunit 4 | SCN4B | 2,35E-02 | -2,044 |
| predicted gene 428 | Gm428 (includes others) | 1,45E-03 | -2,045 |
| Purkinje cell protein 4-like 1 | Pcp4l1 | 4,32E-03 | -2,046 |
| protocadherin 12 | PCDH12 | 2,08E-02 | -2,048 |
| angiotensin I converting enzyme 2 | ACE2 | 9,76E-05 | -2,049 |
| scavenger receptor class A member 5 | SCARA5 | 1,15E-03 | -2,049 |
| heparan sulfate-glucosamine 3-sulfotransferase 5 | HS3ST5 | 2,36E-03 | -2,051 |
| frequently rearranged in advanced T-cell lymphomas 1 | FRAT1 | 7,83E-03 | -2,052 |
| potassium two pore domain channel subfamily K member 3 | KCNK3 | 5,48E-05 | -2,054 |
| myosin VIIA and Rab interacting protein | MYRIP | 5,73E-03 | -2,065 |
| regulator of G-protein signaling 5 | RGS5 | 2,47E-05 | -2,065 |
| SRY-box 4 | SOX4 | 6,42E-03 | -2,073 |
| ADP ribosylation factor like GTPase 4D | ARL4D | 3,70E-03 | -2,076 |
| FXYD domain containing ion transport regulator 6 | FXYD6 | 2,59E-06 | -2,089 |
| ras related dexamethasone induced 1 | RASD1 | 4,71E-02 | -2,093 |
| glutamate metabotropic receptor 1 | GRM1 | 8,25E-04 | -2,096 |
| leucine rich repeat and Ig domain containing 3 | LINGO3 | 6,59E-04 | -2,104 |
| family with sequence similarity 181 member B | FAM181B | 1,51E-05 | -2,114 |
| MAGI family member, X-linked | MAGIX | 8,13E-04 | -2,114 |
| protein tyrosine phosphatase, receptor type R | PTPRR | 3,63E-05 | -2,117 |
| aquaporin 8 | AQP8 | 1,25E-02 | -2,118 |
| EPH receptor B1 | EPHB1 | 2,24E-05 | -2,12 |
| bone morphogenetic protein 6 | BMP6 | 1,24E-03 | -2,121 |
| SRY-box 17 | SOX17 | 1,04E-03 | -2,121 |
| serum/glucocorticoid regulated kinase 1 | SGK1 | 2,62E-03 | -2,122 |
| glucokinase | GCK | 9,86E-03 | -2,123 |
| septin 8 | SEPT8 | 9,37E-06 | -2,128 |
| MLX interacting protein like | MLXIPL | 1,11E-05 | -2,133 |
| ribonuclease A family member 10 (inactive) | RNASE10 | 2,25E-02 | -2,135 |
| armadillo repeat containing 2 | ARMC2 | 5,32E-04 | -2,136 |
| stabilin 2 | STAB2 | 1,99E-03 | -2,136 |
| predicted gene 428 | Gm428 (includes others) | 2,42E-04 | -2,14 |
| guanylate cyclase 1 soluble subunit alpha | GUCY1A3 | 2,03E-06 | -2,144 |
| Y-box binding protein 2 | YBX2 | 5,33E-05 | -2,154 |
| harakiri, BCL2 interacting protein (contains only BH3 domain) | Hrk | 1,83E-05 | -2,156 |
| ATP-binding cassette, sub-family A (ABC1), member 8a | Abca8a | 7,72E-04 | -2,158 |
| adipogenin | Adig | 2,56E-06 | -2,158 |
| solute carrier family 40 member 1 | SLC40A1 | 1,85E-03 | -2,158 |
| syntaxin binding protein 6 | STXBP6 | 6,15E-06 | -2,162 |
| Kazal type serine peptidase inhibitor domain 1 | KAZALD1 | 2,51E-05 | -2,169 |
| interleukin 17D | IL17D | 6,78E-06 | -2,172 |
| aldo-keto reductase family 1, member C19 | Akr1c19 | 5,47E-05 | -2,173 |
| ATPase Na+/K+ transporting subunit beta 2 | ATP1B2 | 1,24E-05 | -2,18 |
| TIMP metallopeptidase inhibitor 4 | TIMP4 | 9,16E-03 | -2,194 |
| G protein-coupled receptor 22 | GPR22 | 8,05E-04 | -2,198 |
| von Willebrand factor A domain containing 3A | VWA3A | 1,01E-04 | -2,198 |
| polo like kinase 5 | PLK5 | 2,51E-05 | -2,199 |
| retinol binding protein 7 | RBP7 | 3,67E-03 | -2,203 |
| purinergic receptor P2X 3 | P2RX3 | 5,58E-03 | -2,205 |
| catenin alpha like 1 | CTNNAL1 | 6,31E-03 | -2,21 |
| sprouty RTK signaling antagonist 1 | SPRY1 | 3,61E-05 | -2,214 |
| A-kinase anchoring protein 5 | AKAP5 | 1,40E-02 | -2,221 |
| potassium voltage-gated channel modifier subfamily V member 2 | KCNV2 | 4,52E-05 | -2,223 |
| paraneoplastic Ma antigen 2 | PNMA2 | 1,54E-02 | -2,258 |
| insulin like growth factor binding protein 3 | IGFBP3 | 1,70E-03 | -2,26 |
| apolipoprotein E | APOE | 2,79E-07 | -2,262 |
| SPARC related modular calcium binding 2 | SMOC2 | 5,29E-05 | -2,262 |
| GRB2 associated binding protein 3 | GAB3 | 2,84E-02 | -2,263 |
| chromosome 4 open reading frame 19 | C4orf19 | 1,02E-04 | -2,265 |
| glycine N-methyltransferase | GNMT | 2,84E-04 | -2,27 |
| CD163 molecule | CD163 | 5,68E-06 | -2,284 |
| CKLF like MARVEL transmembrane domain containing 8 | CMTM8 | 1,76E-04 | -2,284 |
| brain derived neurotrophic factor | BDNF | 9,91E-05 | -2,295 |
| actin, alpha 1, skeletal muscle | ACTA1 | 1,66E-02 | -2,31 |
| cystatin E/M | CST6 | 1,02E-03 | -2,311 |
| C-C motif chemokine ligand 24 | CCL24 | 1,29E-04 | -2,313 |
| synaptopodin | SYNPO | 2,63E-05 | -2,319 |
| RIKEN cDNA B020031M17 gene | B020031M17Rik | 1,25E-02 | -2,325 |
| dopachrome tautomerase | DCT | 9,30E-03 | -2,328 |
| ATP binding cassette subfamily B member 1 | ABCB1 | 2,57E-06 | -2,34 |
| solute carrier family 22 member 1 | SLC22A1 | 9,14E-04 | -2,34 |
| leucine rich repeat containing 4B | LRRC4B | 5,85E-03 | -2,344 |
| predicted gene 10635 | Gm10635 | 3,33E-04 | -2,346 |
| predicted gene 1673 | Gm1673 | 6,60E-05 | -2,351 |
| solute carrier family 26, member 10 | Slc26a10 | 1,64E-03 | -2,353 |
| C-X-C motif chemokine ligand 14 | CXCL14 | 1,84E-04 | -2,354 |
| enoyl-CoA hydratase and 3-hydroxyacyl CoA dehydrogenase | EHHADH | 4,87E-03 | -2,354 |
| protein tyrosine phosphatase, receptor type B | PTPRB | 5,27E-05 | -2,36 |
| HR, lysine demethylase and nuclear receptor corepressor | HR | 3,92E-05 | -2,362 |
| amylase, alpha 2A (pancreatic) | AMY2A | 9,23E-04 | -2,376 |
| anoctamin 10 | ANO10 | 6,31E-05 | -2,376 |
| erythropoietin receptor | EPOR | 2,78E-07 | -2,387 |
| calcium/calmodulin dependent protein kinase II inhibitor 1 | CAMK2N1 | 5,75E-07 | -2,392 |
| ADAMTS like 2 | ADAMTSL2 | 6,83E-05 | -2,395 |
| cold shock domain containing C2 | CSDC2 | 1,73E-03 | -2,399 |
| forkhead box O6 | FOXO6 | 3,59E-04 | -2,401 |
| synemin | SYNM | 1,69E-03 | -2,41 |
| DENN domain containing 5B | DENND5B | 2,93E-06 | -2,416 |
| immunoglobulin superfamily member 1 | IGSF1 | 6,16E-04 | -2,426 |
| hes related family bHLH transcription factor with YRPW motif-like | HEYL | 2,94E-03 | -2,427 |
| glial cells missing homolog 1 | GCM1 | 4,36E-02 | -2,441 |
| latent transforming growth factor beta binding protein 4 | LTBP4 | 1,32E-05 | -2,446 |
| tumor protein D52-like 1 | TPD52L1 | 1,81E-05 | -2,45 |
| kelch like family member 34 | KLHL34 | 1,00E-06 | -2,455 |
| proenkephalin | PENK | 2,25E-05 | -2,457 |
| Wnt family member 5B | WNT5B | 1,48E-05 | -2,468 |
| fin bud initiation factor homolog (zebrafish) | FIBIN | 5,27E-05 | -2,472 |
| ermin | ERMN | 3,03E-02 | -2,48 |
| Nanog homeobox | NANOG | 1,43E-06 | -2,486 |
| glutathione S-transferase mu 2 | GSTM2 | 1,78E-05 | -2,505 |
| pyruvate dehyrogenase phosphatase catalytic subunit 2 | PDP2 | 3,87E-06 | -2,516 |
| neuroligin 3 | NLGN3 | 4,38E-05 | -2,522 |
| RIKEN cDNA 2310002L09 gene | 2310002L09Rik | 1,49E-05 | -2,53 |
| myosin binding protein C, fast type | MYBPC2 | 2,25E-06 | -2,555 |
| ST6 N-acetylgalactosaminide alpha-2,6-sialyltransferase 2 | ST6GALNAC2 | 2,93E-07 | -2,572 |
| cartilage oligomeric matrix protein | COMP | 5,04E-05 | -2,596 |
| feline leukemia virus subgroup C cellular receptor family member 2 | FLVCR2 | 1,39E-03 | -2,596 |
| DDB1 and CUL4 associated factor 12-like 1 | Dcaf12l1 | 1,13E-06 | -2,605 |
| fibulin 5 | FBLN5 | 1,68E-08 | -2,605 |
| RAS like family 10 member A | RASL10A | 3,21E-05 | -2,617 |
| syntaxin binding protein 4 | STXBP4 | 5,02E-04 | -2,617 |
| dopamine beta-hydroxylase | DBH | 3,07E-04 | -2,625 |
| methionine sulfoxide reductase B2 | MSRB2 | 2,73E-05 | -2,636 |
| integrin subunit alpha 11 | ITGA11 | 1,01E-04 | -2,695 |
| dual specificity phosphatase 1 | DUSP1 | 1,15E-04 | -2,701 |
| protein tyrosine phosphatase, receptor type U | PTPRU | 6,79E-05 | -2,72 |
| secretoglobin, family 1C, member 1 | Scgb1c1 | 4,12E-05 | -2,726 |
| RIKEN cDNA 6230415J03 gene | 6230415J03Rik | 3,14E-06 | -2,729 |
| alcohol dehydrogenase 1C (class I), gamma polypeptide | ADH1C | 1,63E-03 | -2,75 |
| stathmin 2 | STMN2 | 8,98E-07 | -2,753 |
| myotilin | MYOT | 1,13E-04 | -2,819 |
| ephrin B3 | EFNB3 | 9,59E-04 | -2,828 |
| fumarylacetoacetate hydrolase | FAH | 9,27E-04 | -2,848 |
| amphiphysin | AMPH | 3,59E-07 | -2,853 |
| potassium voltage-gated channel subfamily A member 1 | KCNA1 | 1,35E-05 | -2,866 |
| WNT inhibitory factor 1 | WIF1 | 1,48E-05 | -2,889 |
| mal, T-cell differentiation protein | MAL | 5,41E-08 | -2,89 |
| serine/arginine repetitive matrix 4 | SRRM4 | 7,35E-04 | -2,906 |
| 3-hydroxy-3-methylglutaryl-CoA synthase 2 | HMGCS2 | 2,10E-02 | -2,915 |
| hydroxyprostaglandin dehydrogenase 15-(NAD) | HPGD | 2,82E-06 | -2,932 |
| brain expressed X-linked 1 | Bex1 | 1,81E-04 | -2,966 |
| MAP6 domain containing 1 | MAP6D1 | 1,71E-06 | -3,021 |
| tachykinin precursor 1 | TAC1 | 6,03E-05 | -3,038 |
| chromosome 10 open reading frame 10 | C10orf10 | 2,53E-05 | -3,111 |
| cytochrome P450 family 26 subfamily B member 1 | CYP26B1 | 3,21E-07 | -3,159 |
| zinc finger protein 488 | ZNF488 | 1,55E-06 | -3,202 |
| predicted gene 4956 | Gm4956 | 1,66E-06 | -3,205 |
| 6-phosphofructo-2-kinase/fructose-2,6-biphosphatase 1 | PFKFB1 | 4,62E-05 | -3,221 |
| neurotrophin 3 | NTF3 | 2,06E-07 | -3,279 |
| cytokine like 1 | CYTL1 | 2,24E-07 | -3,333 |
| aquaporin 1 (Colton blood group) | AQP1 | 2,48E-07 | -3,539 |
| DNA damage inducible transcript 4 | DDIT4 | 1,49E-04 | -3,55 |
| cyclin-dependent kinase inhibitor 1C (P57) | Cdkn1c | 8,76E-09 | -3,631 |
| Fc receptor-like S, scavenger receptor | Fcrls | 1,15E-06 | -3,704 |
| GTP cyclohydrolase I feedback regulator | GCHFR | 3,21E-07 | -3,838 |
| resistin like alpha | Retnla | 9,23E-05 | -5,169 |
| aldolase, fructose-bisphosphate B | ALDOB | 5,10E-05 | -5,203 |
| H19, imprinted maternally expressed transcript (non-protein coding) | H19 | 9,72E-09 | -5,244 |
| indolethylamine N-methyltransferase | INMT | 5,95E-07 | -7,954 |

**Supplemental table 2:** List of all DEGs at 30 dpi.

| **Entrez Gene Name** | **Symbol** | **Expr p-value** | **Expr Fold Change** |
| --- | --- | --- | --- |
| C-C motif chemokine ligand 5 | CCL5 | 4,27E-13 | 185,224 |
| serum amyloid A 3 | Saa3 | 1,25E-09 | 168,35 |
| granzyme B | Gzmb | 2,38E-15 | 130,802 |
| granzyme A | GZMA | 6,23E-13 | 105,918 |
| ubiquitin D | UBD | 4,07E-14 | 96,157 |
| chemokine (C-C motif) ligand 8 | Ccl8 | 7,85E-14 | 86,403 |
| complement factor B | CFB | 1,68E-13 | 85,37 |
| C-X-C motif chemokine ligand 10 | CXCL10 | 2,33E-12 | 83,999 |
| serine (or cysteine) peptidase inhibitor, clade A, member 3G | Serpina3g (includes others) | 3,92E-15 | 83,032 |
| apolipoprotein L 9b | Apol9a/Apol9b | 1,55E-14 | 66,409 |
| Z-DNA binding protein 1 | ZBP1 | 1,38E-14 | 63,492 |
| macrophage receptor with collagenous structure | MARCO | 7,13E-10 | 62,614 |
| placenta specific 8 | PLAC8 | 2,54E-12 | 60,319 |
| membrane-spanning 4-domains, subfamily A, member 4B | Ms4a4b (includes others) | 5,78E-14 | 59,788 |
| chemokine (C-X-C motif) ligand 9 | Cxcl9 | 2,96E-12 | 55,449 |
| LCK proto-oncogene, Src family tyrosine kinase | LCK | 3,51E-13 | 48,726 |
| T cell receptor beta, constant 2 | Trbc2 | 4,16E-13 | 46,178 |
| C-C motif chemokine ligand 4 | CCL4 | 9,04E-13 | 41,384 |
| Fc fragment of IgG receptor IIIa | FCGR3A/FCGR3B | 1,38E-14 | 41,292 |
| interferon gamma induced GTPase | Igtp | 5,78E-14 | 40,285 |
| galectin 3 | LGALS3 | 5,66E-13 | 39,845 |
| integrin subunit beta 7 | ITGB7 | 2,12E-13 | 37,99 |
| T cell receptor beta, constant region 1 | Trbc1 | 3,57E-12 | 36,885 |
| acid phosphatase 5, tartrate resistant | Acp5 | 1,37E-13 | 36,384 |
| haptoglobin | HP | 1,84E-08 | 36,067 |
| C-type lectin domain family 4 member E | CLEC4E | 3,75E-09 | 34,345 |
| interferon gamma | IFNG | 1,10E-12 | 34,189 |
| immunity-related GTPase family M member 1 | Irgm1 | 3,92E-15 | 34,101 |
| basic leucine zipper ATF-like transcription factor 2 | BATF2 | 7,85E-14 | 33,006 |
| CD3d molecule | CD3D | 1,38E-14 | 30,19 |
| SLAM family member 8 | SLAMF8 | 5,38E-13 | 29,976 |
| guanylate binding protein 2 | GBP2 | 3,45E-11 | 29,744 |
| C-X-C motif chemokine receptor 6 | CXCR6 | 1,98E-13 | 29,296 |
| lymphotoxin beta | LTB | 2,78E-10 | 28,865 |
| CD6 molecule | CD6 | 1,13E-13 | 28,777 |
| interferon gamma inducible protein 47 | Ifi47 | 1,68E-12 | 27,481 |
| major histocompatibility complex, class II, DM beta | HLA-DMB | 4,35E-10 | 26,936 |
| CD2 molecule | CD2 | 8,56E-13 | 26,255 |
| CD52 antigen | Cd52 | 5,78E-12 | 25,892 |
| BCL2 related protein A1 | BCL2A1 | 3,34E-11 | 25,854 |
| cathepsin S | CTSS | 4,62E-12 | 25,799 |
| complement C2 | C2 | 6,08E-12 | 25,673 |
| interferon regulatory factor 7 | IRF7 | 1,09E-14 | 24,714 |
| T cell specific GTPase 1 | Tgtp1/Tgtp2 | 5,05E-10 | 24,566 |
| CD300 molecule like family member f | CD300LF | 8,24E-08 | 24,337 |
| zeta chain of T cell receptor associated protein kinase 70 | ZAP70 | 2,59E-15 | 24,069 |
| CD274 molecule | CD274 | 5,67E-11 | 22,691 |
| macrophage expressed 1 | MPEG1 | 7,85E-14 | 22,564 |
| major histocompatibility complex, class II, DQ alpha 1 | HLA-DQA1 | 3,24E-09 | 22,307 |
| guanylate binding protein 4 | GBP4 | 5,52E-12 | 21,618 |
| dual specificity phosphatase 2 | DUSP2 | 1,11E-10 | 21,346 |
| CD74 molecule | CD74 | 6,09E-09 | 21,052 |
| cathepsin W | CTSW | 2,60E-14 | 20,959 |
| integrin subunit alpha X | ITGAX | 2,93E-11 | 20,029 |
| CD3g molecule | CD3G | 1,37E-13 | 19,091 |
| C-type lectin domain family 4 member D | CLEC4D | 2,28E-08 | 18,982 |
| sialophorin | SPN | 1,38E-14 | 18,892 |
| interferon induced protein with tetratricopeptide repeats 3 | IFIT3 | 1,74E-13 | 18,8 |
| CD5 molecule | CD5 | 1,74E-13 | 18,644 |
| solute carrier family 2 member 6 | SLC2A6 | 1,30E-11 | 18,243 |
| proteasome subunit beta 8 | PSMB8 | 1,38E-14 | 18,008 |
| cytochrome P450 family 4 subfamily F member 2 | CYP4F2 | 1,31E-11 | 17,947 |
| granzyme K | GZMK | 5,87E-13 | 17,777 |
| MHC class I family member | LOC547349 | 3,11E-16 | 17,542 |
| HCK proto-oncogene, Src family tyrosine kinase | HCK | 4,31E-12 | 17,435 |
| ras-related C3 botulinum toxin substrate 2 (rho family, small GTP binding protein Rac2) | RAC2 | 1,29E-13 | 17,4 |
| ADAM metallopeptidase domain 8 | ADAM8 | 7,59E-10 | 17,345 |
| major histocompatibility complex, class II, DM alpha | HLA-DMA | 9,41E-11 | 16,791 |
| CD72 molecule | CD72 | 5,19E-11 | 16,72 |
| major histocompatibility complex, class II, DR beta 5 | HLA-DRB5 | 2,66E-08 | 16,501 |
| formyl peptide receptor 2 | FPR2 | 2,34E-08 | 16,492 |
| GIMAP1-GIMAP5 readthrough | GIMAP1-GIMAP5 | 3,49E-10 | 16,387 |
| integrin subunit beta 2 | ITGB2 | 3,52E-13 | 16,144 |
| bone marrow stromal cell antigen 1 | BST1 | 9,13E-13 | 16,129 |
| toll-like receptor 12 | Tlr12 | 4,69E-12 | 16,066 |
| TIMP metallopeptidase inhibitor 1 | TIMP1 | 1,63E-08 | 15,993 |
| coronin 1A | CORO1A | 8,64E-12 | 15,96 |
| protein tyrosine phosphatase, receptor type C | PTPRC | 4,61E-13 | 15,908 |
| src kinase associated phosphoprotein 1 | SKAP1 | 3,97E-12 | 15,827 |
| phospholipase A2 group VII | PLA2G7 | 6,35E-11 | 15,745 |
| C-C motif chemokine receptor 5 (gene/pseudogene) | CCR5 | 6,08E-12 | 15,345 |
| immunity related GTPase M | IRGM | 4,27E-13 | 15,326 |
| allograft inflammatory factor 1 | AIF1 | 1,25E-10 | 15,178 |
| protein tyrosine phosphatase, receptor type C associated protein | PTPRCAP | 1,74E-13 | 15,168 |
| suppressor of cytokine signaling 1 | SOCS1 | 6,82E-11 | 15,114 |
| chemokine (C-C motif) ligand 2 | Ccl2 | 1,09E-07 | 15,046 |
| hematopoietic cell signal transducer | HCST | 4,00E-14 | 14,902 |
| chemokine (C-C motif) ligand 7 | Ccl7 | 1,10E-07 | 14,604 |
| guanylate binding protein family member 6 | GBP6 | 5,32E-14 | 14,558 |
| CD300c molecule | CD300C | 2,17E-10 | 14,428 |
| C-X-C motif chemokine receptor 3 | CXCR3 | 1,77E-12 | 14,399 |
| signal regulatory protein beta 1 | SIRPB1 | 2,76E-12 | 14,284 |
| junction adhesion molecule like | JAML | 9,08E-08 | 14,15 |
| interleukin 12 receptor subunit beta 1 | IL12RB1 | 8,55E-13 | 14,089 |
| major histocompatibility complex, class II, DQ beta 1 | HLA-DQB1 | 1,25E-07 | 14,028 |
| transporter 1, ATP binding cassette subfamily B member | TAP1 | 9,13E-13 | 13,963 |
| apolipoprotein C2 | APOC2 | 7,52E-09 | 13,642 |
| interleukin 2 receptor subunit beta | IL2RB | 1,33E-12 | 13,613 |
| IL2 inducible T-cell kinase | ITK | 5,38E-13 | 13,369 |
| natural killer cell granule protein 7 | NKG7 | 1,19E-13 | 13,346 |
| major histocompatibility complex, class I, A | HLA-A | 7,70E-14 | 13,295 |
| C-X-C motif chemokine ligand 6 | CXCL6 | 1,28E-07 | 13,211 |
| paired-Ig-like receptor A7 | Pira7 | 9,87E-12 | 13,164 |
| membrane spanning 4-domains A7 | MS4A7 | 9,29E-11 | 13,02 |
| interferon inducible GTPase 1 | Iigp1 | 2,12E-13 | 12,953 |
| killer cell lectin-like receptor, subfamily A, member 2 | Klra2 | 2,12E-13 | 12,878 |
| serum amyloid A1 | SAA1 | 3,54E-06 | 12,874 |
| family with sequence similarity 26 member F | FAM26F | 1,98E-12 | 12,84 |
| histocompatibility 2, M region locus 2 | H2-M2 | 8,07E-13 | 12,678 |
| histone cluster 1 H2A family member d | HIST1H2AD | 6,70E-10 | 12,626 |
| WD repeat and FYVE domain containing 1 | WDFY1 | 6,87E-11 | 12,605 |
| dipeptidase 2 | DPEP2 | 3,17E-10 | 12,465 |
| histocompatibility 2, Q region locus 8 | H2-Q8 | 1,30E-09 | 12,384 |
| cholesterol 25-hydroxylase | CH25H | 5,48E-09 | 12,298 |
| guanylate binding protein family member 6 | GBP6 | 7,73E-11 | 12,274 |
| S100 calcium binding protein A4 | S100A4 | 1,90E-11 | 12,155 |
| T-box 21 | TBX21 | 1,88E-12 | 12,102 |
| complement C3 | C3 | 2,26E-12 | 11,925 |
| ISG15 ubiquitin-like modifier | ISG15 | 4,79E-11 | 11,775 |
| myosin IG | MYO1G | 6,41E-12 | 11,758 |
| triggering receptor expressed on myeloid cells 2 | TREM2 | 6,35E-11 | 11,726 |
| leukocyte immunoglobulin like receptor B3 | LILRB3 | 2,39E-12 | 11,69 |
| solute carrier family 11 member 1 | SLC11A1 | 1,21E-11 | 11,598 |
| linker for activation of T-cells | LAT | 2,54E-12 | 11,593 |
| hexokinase 3 | HK3 | 1,04E-11 | 11,589 |
| guanylate binding protein 5 | GBP5 | 8,36E-09 | 11,415 |
| cytochrome b-245 beta chain | CYBB | 4,12E-11 | 11,33 |
| neutrophil cytosolic factor 4 | NCF4 | 1,45E-11 | 11,326 |
| C-type lectin domain family 7 member A | CLEC7A | 1,56E-08 | 11,271 |
| cystatin F | CST7 | 4,98E-12 | 11,271 |
| paired-Ig-like receptor A11 | Pira11 | 1,35E-11 | 11,239 |
| inhibitor of DNA binding 2, HLH protein | ID2 | 2,56E-10 | 11,212 |
| proteasome subunit beta 9 | PSMB9 | 6,24E-12 | 11,207 |
| histocompatibility 2, T region locus 9 | H2-T9 | 1,02E-13 | 11,052 |
| Fc fragment of IgE receptor Ig | FCER1G | 2,14E-11 | 10,786 |
| baculoviral IAP repeat containing 5 | BIRC5 | 1,56E-09 | 10,762 |
| histocompatibility 2, Q region locus 5 | H2-Q5 | 1,91E-13 | 10,675 |
| major histocompatibility complex, class I, A | HLA-A | 2,28E-09 | 10,64 |
| leukocyte immunoglobulin like receptor B3 | LILRB3 | 7,43E-13 | 10,619 |
| inducible T-cell costimulator | ICOS | 3,50E-09 | 10,502 |
| major histocompatibility complex, class I, E | HLA-E | 1,37E-13 | 10,45 |
| lymphocyte antigen 86 | LY86 | 4,84E-10 | 10,359 |
| G protein-coupled receptor 132 | GPR132 | 2,48E-10 | 10,328 |
| FYN binding protein | FYB | 6,32E-13 | 10,314 |
| selectin P ligand | SELPLG | 7,62E-12 | 10,227 |
| phospholipase D family member 4 | PLD4 | 2,61E-11 | 10,21 |
| major histocompatibility complex, class I, A | HLA-A | 2,61E-11 | 10,195 |
| complement C4B (Chido blood group) | C4A/C4B | 2,01E-13 | 10,169 |
| colony stimulating factor 2 receptor beta common subunit | CSF2RB | 2,62E-10 | 10,162 |
| CD7 molecule | CD7 | 1,04E-08 | 10,089 |
| caspase recruitment domain family member 11 | CARD11 | 7,70E-14 | 10,083 |
| MEFV, pyrin innate immunity regulator | MEFV | 5,07E-10 | 10,055 |
| IFI30, lysosomal thiol reductase | IFI30 | 8,98E-10 | 10,046 |
| membrane spanning 4-domains A6A | MS4A6A | 3,28E-10 | 9,963 |
| CD8b molecule | CD8B | 7,02E-09 | 9,959 |
| polypeptide N-acetylgalactosaminyltransferase 6 | GALNT6 | 3,82E-09 | 9,931 |
| killer cell lectin like receptor C1 | KLRC1 | 7,73E-11 | 9,787 |
| lymphocyte cytosolic protein 1 | LCP1 | 6,34E-11 | 9,761 |
| histocompatibility 2, T region locus 10 | H2-T10 | 1,10E-12 | 9,751 |
| protein tyrosine phosphatase, non-receptor type 22 | PTPN22 | 1,66E-10 | 9,746 |
| sodium voltage-gated channel alpha subunit 10 | SCN10A | 6,14E-12 | 9,716 |
| interleukin 18 binding protein | IL18BP | 5,38E-13 | 9,666 |
| CD8a molecule | CD8A | 5,11E-09 | 9,615 |
| proline-serine-threonine phosphatase interacting protein 1 | PSTPIP1 | 2,42E-11 | 9,548 |
| NLR family CARD domain containing 5 | NLRC5 | 5,03E-09 | 9,526 |
| protein tyrosine phosphatase, non-receptor type 6 | PTPN6 | 5,30E-10 | 9,521 |
| interferon regulatory factor 8 | IRF8 | 2,76E-12 | 9,503 |
| integrin subunit alpha L | ITGAL | 1,21E-11 | 9,489 |
| leukocyte immunoglobulin like receptor B3 | LILRB3 | 5,44E-12 | 9,488 |
| vav guanine nucleotide exchange factor 1 | VAV1 | 7,86E-12 | 9,363 |
| arginase 1 | ARG1 | 1,09E-04 | 9,337 |
| coiled-coil domain containing 88B | CCDC88B | 7,31E-13 | 9,308 |
| glia maturation factor gamma | GMFG | 5,95E-11 | 9,278 |
| major histocompatibility complex, class I, A | HLA-A | 1,33E-12 | 9,246 |
| LIM domain containing 2 | LIMD2 | 5,19E-11 | 9,191 |
| major histocompatibility complex, class I, A | HLA-A | 3,05E-11 | 9,15 |
| GLI pathogenesis related 1 | GLIPR1 | 2,33E-12 | 9,148 |
| leupaxin | LPXN | 1,16E-09 | 9,004 |
| lysosomal protein transmembrane 5 | LAPTM5 | 1,12E-10 | 8,987 |
| histocompatibility 2, Q region locus 5 | H2-Q5 | 4,41E-12 | 8,978 |
| kinesin family member 21B | KIF21B | 5,11E-11 | 8,927 |
| cell division cycle associated 8 | CDCA8 | 6,82E-11 | 8,876 |
| guanylate binding protein family member 6 | GBP6 | 1,10E-09 | 8,827 |
| complement C1q B chain | C1QB | 8,31E-12 | 8,672 |
| colony stimulating factor 2 receptor beta common subunit | CSF2RB | 3,24E-09 | 8,656 |
| myosin IF | MYO1F | 3,86E-08 | 8,646 |
| marker of proliferation Ki-67 | MKI67 | 1,80E-10 | 8,62 |
| leukocyte associated immunoglobulin like receptor 1 | LAIR1 | 8,61E-11 | 8,602 |
| E2F transcription factor 2 | E2F2 | 5,96E-11 | 8,576 |
| cyclin B2 | CCNB2 | 3,61E-09 | 8,485 |
| sorting nexin 20 | SNX20 | 6,65E-11 | 8,451 |
| solute carrier family 15 member 3 | SLC15A3 | 2,37E-10 | 8,437 |
| guanylate-binding protein 8 | Gbp8 | 5,02E-11 | 8,419 |
| caspase 1 | CASP1 | 9,92E-11 | 8,413 |
| glycoprotein nmb | GPNMB | 1,64E-08 | 8,409 |
| TYRO protein tyrosine kinase binding protein | TYROBP | 4,08E-10 | 8,401 |
| Ras association domain family member 5 | RASSF5 | 2,81E-10 | 8,317 |
| small proline rich protein 1A | SPRR1A | 1,38E-08 | 8,316 |
| CD84 molecule | CD84 | 3,96E-09 | 8,301 |
| RAB32, member RAS oncogene family | RAB32 | 2,56E-11 | 8,267 |
| CD68 molecule | CD68 | 6,35E-11 | 8,259 |
| pleckstrin | PLEK | 1,59E-09 | 8,254 |
| paired immunoglobin like type 2 receptor alpha | PILRA | 3,41E-10 | 8,253 |
| interleukin 10 receptor subunit alpha | IL10RA | 3,14E-11 | 8,25 |
| G protein subunit gamma 2 | GNG2 | 1,18E-11 | 8,224 |
| CD3e molecule | CD3E | 4,14E-12 | 8,211 |
| parvin gamma | PARVG | 2,21E-11 | 8,18 |
| interferon induced protein with tetratricopeptide repeats 1B | IFIT1B | 1,98E-10 | 8,157 |
| Src-like-adaptor | SLA | 1,43E-09 | 8,153 |
| lysozyme | LYZ | 1,93E-09 | 8,146 |
| B cell leukemia/lymphoma 2 related protein A1c | Bcl2a1c | 8,48E-11 | 8,127 |
| FCH domain only 1 | FCHO1 | 1,57E-11 | 8,077 |
| cytohesin 1 interacting protein | CYTIP | 5,82E-09 | 8,06 |
| serine (or cysteine) peptidase inhibitor, clade A, member 3H | Serpina3h | 5,55E-07 | 8,042 |
| semaphorin 4D | SEMA4D | 2,48E-10 | 8,028 |
| integrin subunit alpha 4 | ITGA4 | 7,73E-11 | 8,005 |
| CD48 molecule | CD48 | 5,48E-12 | 7,983 |
| thymocyte selection associated family member 2 | THEMIS2 | 3,87E-12 | 7,957 |
| interferon induced protein with tetratricopeptide repeats 2 | IFIT2 | 1,10E-09 | 7,946 |
| immunoglobulin superfamily member 6 | IGSF6 | 2,18E-09 | 7,923 |
| synaptotagmin like 3 | SYTL3 | 1,21E-09 | 7,92 |
| complement C1q A chain | C1QA | 1,13E-11 | 7,899 |
| G protein-coupled receptor 65 | GPR65 | 7,18E-12 | 7,826 |
| Fc fragment of IgG receptor IIb | FCGR2B | 3,77E-09 | 7,778 |
| complement C1q C chain | C1QC | 4,39E-11 | 7,777 |
| myelin basic protein | MBP | 2,39E-13 | 7,754 |
| SAM and SH3 domain containing 3 | SASH3 | 2,17E-12 | 7,744 |
| zinc finger protein 705A | ZNF705A | 2,42E-11 | 7,741 |
| Fas ligand | FASLG | 2,40E-13 | 7,732 |
| suppressor of cytokine signaling 3 | SOCS3 | 7,10E-09 | 7,678 |
| interferon regulatory factor 1 | IRF1 | 3,55E-11 | 7,666 |
| proteasome subunit beta 10 | PSMB10 | 6,35E-12 | 7,654 |
| RAS guanyl releasing protein 1 | RASGRP1 | 2,49E-11 | 7,646 |
| solute carrier family 16 member 3 | SLC16A3 | 7,92E-11 | 7,639 |
| fermitin family member 3 | FERMT3 | 3,97E-12 | 7,623 |
| toll-like receptor 13 | Tlr13 | 5,77E-10 | 7,584 |
| caspase 4 | CASP4 | 5,29E-10 | 7,554 |
| transforming growth factor beta induced | TGFBI | 5,15E-08 | 7,54 |
| heparanase | HPSE | 1,62E-10 | 7,536 |
| tumor necrosis factor | TNF | 6,28E-10 | 7,534 |
| XIAP associated factor 1 | XAF1 | 1,38E-12 | 7,491 |
| acyloxyacyl hydrolase | AOAH | 1,44E-10 | 7,477 |
| membrane-spanning 4-domains, subfamily A, member 6C | Ms4a6c | 9,59E-09 | 7,467 |
| killer cell lectin like receptor G1 | KLRG1 | 2,12E-11 | 7,425 |
| C-C motif chemokine ligand 3 like 3 | CCL3L3 | 5,96E-07 | 7,394 |
| regulator of G-protein signaling 1 | RGS1 | 1,26E-11 | 7,383 |
| coactosin like F-actin binding protein 1 | COTL1 | 5,33E-10 | 7,315 |
| G protein-coupled receptor 171 | GPR171 | 1,98E-12 | 7,302 |
| SLAM family member 9 | SLAMF9 | 4,74E-11 | 7,264 |
| Rho GTPase activating protein 45 | ARHGAP45 | 1,35E-11 | 7,231 |
| major histocompatibility complex, class I, A | HLA-A | 2,94E-10 | 7,219 |
| Thy-1 cell surface antigen | THY1 | 2,94E-10 | 7,122 |
| cytohesin 4 | CYTH4 | 2,18E-09 | 7,105 |
| major histocompatibility complex, class I, A | HLA-A | 5,20E-11 | 7,104 |
| Fc fragment of IgG receptor IIa | FCGR2A | 3,93E-10 | 7,089 |
| interferon regulatory factor 5 | IRF5 | 2,12E-11 | 7,031 |
| protein tyrosine phosphatase, non-receptor type 18 | PTPN18 | 3,67E-09 | 7,012 |
| napsin A aspartic peptidase | NAPSA | 6,19E-11 | 6,981 |
| schlafen 2 | Slfn2 | 4,70E-10 | 6,952 |
| embigin | EMB | 3,62E-06 | 6,9 |
| G protein-coupled receptor 68 | GPR68 | 2,03E-09 | 6,823 |
| pannexin 1 | PANX1 | 1,62E-10 | 6,728 |
| TRAF interacting protein with forkhead associated domain | TIFA | 4,75E-08 | 6,69 |
| hydrogen voltage gated channel 1 | HVCN1 | 2,68E-09 | 6,675 |
| heme oxygenase 1 | HMOX1 | 3,35E-07 | 6,643 |
| C-X-C motif chemokine ligand 16 | CXCL16 | 2,30E-10 | 6,629 |
| 2'-5'-oligoadenylate synthetase 1 | OAS1 | 5,13E-08 | 6,619 |
| vascular cell adhesion molecule 1 | VCAM1 | 4,33E-10 | 6,58 |
| basic leucine zipper ATF-like transcription factor 3 | BATF3 | 1,63E-08 | 6,579 |
| beta-1,4-N-acetyl-galactosaminyltransferase 1 | B4GALNT1 | 2,13E-11 | 6,567 |
| hematopoietic cell-specific Lyn substrate 1 | HCLS1 | 7,45E-12 | 6,5 |
| polo like kinase 1 | PLK1 | 1,35E-09 | 6,465 |
| killer cell lectin-like receptor subfamily A, member 22 | Klra22 | 1,43E-09 | 6,458 |
| 2'-5'-oligoadenylate synthetase 3 | OAS3 | 1,13E-10 | 6,442 |
| C-type lectin domain family 4, member a3 | Clec4a3 | 5,18E-09 | 6,44 |
| villin like | VILL | 3,41E-10 | 6,361 |
| interleukin 2 receptor subunit gamma | IL2RG | 1,17E-09 | 6,354 |
| leucine rich repeat containing 25 | LRRC25 | 2,99E-09 | 6,342 |
| ecotropic viral integration site 2A | EVI2A | 3,06E-08 | 6,341 |
| NCK associated protein 1 like | NCKAP1L | 5,30E-10 | 6,333 |
| thromboxane A synthase 1 | TBXAS1 | 1,42E-10 | 6,332 |
| lymphocyte antigen 6 complex, locus A | Ly6a (includes others) | 7,72E-07 | 6,324 |
| toll like receptor 2 | TLR2 | 1,12E-10 | 6,321 |
| interleukin 21 receptor | IL21R | 7,32E-10 | 6,316 |
| regulator of G-protein signaling 14 | RGS14 | 5,24E-12 | 6,311 |
| mitogen-activated protein kinase kinase kinase kinase 1 | MAP4K1 | 1,82E-12 | 6,307 |
| 6-phosphofructo-2-kinase/fructose-2,6-biphosphatase 3 | PFKFB3 | 5,43E-09 | 6,279 |
| beta-2-microglobulin | B2M | 3,74E-09 | 6,274 |
| MX dynamin-like GTPase 1 | Mx1/Mx2 | 1,47E-08 | 6,252 |
| galectin 3 binding protein | LGALS3BP | 6,35E-12 | 6,224 |
| serpin family A member 3 | SERPINA3 | 3,47E-04 | 6,218 |
| Epstein-Barr virus induced 3 | EBI3 | 5,05E-07 | 6,211 |
| CD96 molecule | CD96 | 1,94E-12 | 6,209 |
| C-C motif chemokine ligand 2 | CCL2 | 8,04E-07 | 6,206 |
| arachidonate 5-lipoxygenase activating protein | ALOX5AP | 1,25E-10 | 6,164 |
| histidine decarboxylase | HDC | 2,17E-07 | 6,137 |
| C-type lectin domain family 6 member A | CLEC6A | 2,67E-09 | 6,106 |
| Rho GTPase activating protein 9 | ARHGAP9 | 7,73E-11 | 6,09 |
| semaphorin 4A | SEMA4A | 1,79E-10 | 6,088 |
| FXYD domain containing ion transport regulator 5 | FXYD5 | 3,10E-10 | 6,085 |
| histone cluster 1, H1b | Hist1h1b | 1,04E-08 | 6,044 |
| apolipoprotein L 11a | Apol10a (includes others) | 1,73E-08 | 6,042 |
| SLAM family member 7 | SLAMF7 | 6,51E-12 | 6,035 |
| cyclin A2 | CCNA2 | 9,96E-10 | 6,005 |
| Wiskott-Aldrich syndrome | WAS | 1,18E-11 | 5,979 |
| receptor transporter protein 4 | RTP4 | 1,61E-10 | 5,957 |
| amyloid beta precursor protein binding family B member 1 interacting protein | APBB1IP | 1,75E-10 | 5,951 |
| major histocompatibility complex, class I, G | HLA-G | 4,90E-10 | 5,899 |
| killer cell lectin like receptor D1 | KLRD1 | 1,49E-10 | 5,884 |
| CD53 molecule | CD53 | 1,98E-11 | 5,86 |
| SH3 domain binding protein 2 | SH3BP2 | 2,21E-11 | 5,843 |
| TNF receptor superfamily member 18 | TNFRSF18 | 2,18E-11 | 5,822 |
| complement C1r | C1R | 5,07E-12 | 5,8 |
| bone marrow stromal cell antigen 2 | Bst2 | 2,58E-09 | 5,795 |
| solute carrier family 39 member 4 | SLC39A4 | 2,37E-10 | 5,741 |
| phosphatidylinositol-4,5-bisphosphate 3-kinase catalytic subunit delta | PIK3CD | 2,24E-08 | 5,733 |
| interferon gamma inducible protein 16 | IFI16 | 3,61E-11 | 5,705 |
| EF-hand domain family member D2 | EFHD2 | 4,74E-10 | 5,702 |
| killer cell lectin-like receptor, subfamily A, member 4 | Klra7 (includes others) | 1,17E-09 | 5,7 |
| phorbol-12-myristate-13-acetate-induced protein 1 | Pmaip1 | 6,88E-10 | 5,593 |
| histone cluster 1 H2A family member b | HIST1H2AB | 6,41E-10 | 5,56 |
| lymphocyte antigen 6 complex, locus A | Ly6a (includes others) | 3,78E-07 | 5,52 |
| GTPase, very large interferon inducible 1 pseudogene | Gm4759 | 1,41E-08 | 5,508 |
| protein tyrosine phosphatase, non-receptor type 7 | PTPN7 | 6,35E-11 | 5,504 |
| kinesin family member 22 | KIF22 | 5,02E-08 | 5,488 |
| WDFY family member 4 | WDFY4 | 3,35E-09 | 5,464 |
| SAM and HD domain containing deoxynucleoside triphosphate triphosphohydrolase 1 | SAMHD1 | 3,01E-10 | 5,458 |
| ATP binding cassette subfamily G member 1 | ABCG1 | 1,77E-09 | 5,448 |
| basic leucine zipper ATF-like transcription factor | BATF | 1,14E-10 | 5,397 |
| unc-93 homolog B1 (C. elegans) | UNC93B1 | 1,52E-10 | 5,363 |
| lymphoid restricted membrane protein | LRMP | 8,54E-12 | 5,359 |
| G-protein signaling modulator 3 | GPSM3 | 1,88E-10 | 5,348 |
| growth factor independent 1 transcriptional repressor | GFI1 | 1,87E-11 | 5,34 |
| NFKB inhibitor delta | NFKBID | 1,48E-09 | 5,337 |
| tetraspanin 32 | TSPAN32 | 1,82E-10 | 5,333 |
| ras homolog family member F, filopodia associated | RHOF | 1,89E-10 | 5,319 |
| N-acylethanolamine acid amidase | NAAA | 6,94E-10 | 5,31 |
| Rho GTPase activating protein 30 | ARHGAP30 | 9,11E-10 | 5,308 |
| protein regulator of cytokinesis 1 | PRC1 | 1,02E-08 | 5,285 |
| WNT1 inducible signaling pathway protein 2 | WISP2 | 5,17E-08 | 5,277 |
| apolipoprotein L 7e | Apol7e (includes others) | 1,17E-11 | 5,261 |
| TNF alpha induced protein 8 like 2 | TNFAIP8L2 | 1,38E-10 | 5,261 |
| potassium calcium-activated channel subfamily N member 4 | KCNN4 | 4,25E-10 | 5,238 |
| GTPase, IMAP family member 7 | GIMAP7 | 6,54E-09 | 5,236 |
| cytokine receptor like factor 1 | CRLF1 | 3,10E-06 | 5,223 |
| transmembrane protein 173 | TMEM173 | 2,73E-09 | 5,21 |
| 2'-5' oligoadenylate synthetase 1F | Oas1f | 6,46E-09 | 5,201 |
| protein C receptor | PROCR | 1,46E-09 | 5,195 |
| C-C motif chemokine ligand 19 | CCL19 | 2,95E-08 | 5,189 |
| chromosome 16 open reading frame 54 | C16orf54 | 8,62E-11 | 5,148 |
| centromere protein E | CENPE | 1,39E-09 | 5,102 |
| ring finger protein 19B | RNF19B | 2,09E-09 | 5,095 |
| capping actin protein, gelsolin like | CAPG | 1,89E-11 | 5,092 |
| C-C motif chemokine receptor 2 | CCR2 | 1,21E-09 | 5,09 |
| ubiquitin conjugating enzyme E2 C | UBE2C | 5,30E-10 | 5,077 |
| leukotriene B4 receptor | LTB4R | 3,18E-05 | 5,074 |
| inositol polyphosphate-5-phosphatase D | INPP5D | 1,62E-10 | 5,04 |
| TAP binding protein like | TAPBPL | 3,01E-12 | 5,03 |
| cathepsin C | CTSC | 2,39E-10 | 5,027 |
| cell division cycle associated 3 | CDCA3 | 1,36E-07 | 5,016 |
| FYVE, RhoGEF and PH domain containing 2 | FGD2 | 1,64E-09 | 5 |
| Fc fragment of IgG receptor Ia | FCGR1A | 1,51E-09 | 4,998 |
| TNF alpha induced protein 3 | TNFAIP3 | 1,20E-07 | 4,998 |
| UDP glucuronosyltransferase family 1 member A6 | UGT1A6 | 2,09E-06 | 4,997 |
| NFAT activating protein with ITAM motif 1 | NFAM1 | 5,06E-08 | 4,972 |
| lymphocyte activating 3 | LAG3 | 7,97E-11 | 4,956 |
| ADP ribosylation factor like GTPase 5C | ARL5C | 5,06E-07 | 4,954 |
| argininosuccinate synthase 1 | ASS1 | 2,14E-09 | 4,953 |
| 2'-5'-oligoadenylate synthetase like | OASL | 7,24E-08 | 4,953 |
| topoisomerase (DNA) II alpha | TOP2A | 2,61E-10 | 4,949 |
| Rho GDP dissociation inhibitor beta | ARHGDIB | 6,51E-10 | 4,946 |
| cathepsin Z | CTSZ | 3,01E-10 | 4,929 |
| dedicator of cytokinesis 2 | DOCK2 | 2,16E-11 | 4,917 |
| histocompatibility 2, M region locus 11 | H2-M11 | 3,79E-07 | 4,915 |
| secretory leukocyte peptidase inhibitor | SLPI | 3,72E-04 | 4,915 |
| ring finger protein 19B | RNF19B | 1,97E-06 | 4,904 |
| tumor necrosis factor superfamily member 13b | TNFSF13B | 3,15E-10 | 4,885 |
| DENN domain containing 1C | DENND1C | 1,43E-09 | 4,884 |
| sorting nexin 10 | SNX10 | 1,46E-08 | 4,881 |
| CD69 molecule | CD69 | 7,89E-11 | 4,88 |
| poly(ADP-ribose) polymerase family member 14 | PARP14 | 1,69E-09 | 4,867 |
| docking protein 2 | DOK2 | 3,61E-09 | 4,848 |
| phosphatidylinositol-3,4,5-trisphosphate dependent Rac exchange factor 1 | PREX1 | 1,71E-09 | 4,843 |
| killer cell lectin-like receptor, subfamily A, member 16 | Klra16 | 4,21E-07 | 4,837 |
| syntaxin binding protein 2 | STXBP2 | 8,79E-10 | 4,837 |
| lysozyme | LYZ | 7,18E-09 | 4,818 |
| guanylate binding protein family member 6 | GBP6 | 1,18E-09 | 4,808 |
| dedicator of cytokinesis 10 | DOCK10 | 2,84E-10 | 4,804 |
| FYVE, RhoGEF and PH domain containing 3 | FGD3 | 3,50E-09 | 4,796 |
| TNF receptor superfamily member 4 | TNFRSF4 | 6,36E-08 | 4,786 |
| SH3 domain binding glutamate rich protein like 3 | SH3BGRL3 | 2,43E-10 | 4,776 |
| family with sequence similarity 105 member A | FAM105A | 3,11E-09 | 4,773 |
| coronin 2A | CORO2A | 9,47E-10 | 4,75 |
| unc-13 homolog D | UNC13D | 2,00E-09 | 4,744 |
| pleckstrin and Sec7 domain containing 4 | PSD4 | 1,13E-09 | 4,735 |
| NFKB inhibitor epsilon | NFKBIE | 1,36E-09 | 4,711 |
| interleukin 18 receptor 1 | IL18R1 | 2,28E-09 | 4,706 |
| C-X-C motif chemokine ligand 13 | CXCL13 | 8,28E-07 | 4,689 |
| TNF receptor associated factor 1 | TRAF1 | 8,23E-09 | 4,685 |
| tubulin beta 3 class III | TUBB3 | 4,90E-06 | 4,67 |
| docking protein 1 | DOK1 | 1,07E-09 | 4,664 |
| linker for activation of T-cells family member 2 | LAT2 | 5,63E-09 | 4,654 |
| receptor interacting serine/threonine kinase 3 | RIPK3 | 8,59E-09 | 4,639 |
| fibrinogen like 2 | FGL2 | 3,72E-10 | 4,622 |
| membrane spanning 4-domains A8 | MS4A8 | 6,31E-06 | 4,622 |
| AT-hook transcription factor | AKNA | 1,45E-10 | 4,621 |
| deoxyribonuclease 1 like 3 | DNASE1L3 | 2,15E-09 | 4,615 |
| Rho GTPase activating protein 4 | ARHGAP4 | 1,48E-09 | 4,611 |
| T cell receptor beta, variable 13-2 | Trbv13-2 | 5,03E-11 | 4,598 |
| schlafen family member 13 | SLFN13 | 3,34E-10 | 4,588 |
| guanine deaminase | GDA | 1,30E-07 | 4,583 |
| neutrophil cytosolic factor 1 | NCF1 | 1,55E-11 | 4,579 |
| Rac GTPase activating protein 1 | RACGAP1 | 3,15E-10 | 4,567 |
| Rho GTPase activating protein 15 | ARHGAP15 | 3,93E-08 | 4,549 |
| ubiquitin conjugating enzyme E2 L6 | UBE2L6 | 2,20E-09 | 4,506 |
| zinc finger MYND-type containing 15 | ZMYND15 | 1,02E-09 | 4,49 |
| ubiquitin specific peptidase 18 | USP18 | 2,68E-09 | 4,485 |
| arrestin beta 2 | ARRB2 | 2,20E-09 | 4,47 |
| formyl peptide receptor 1 | FPR1 | 1,02E-07 | 4,46 |
| peptidyl arginine deiminase 2 | PADI2 | 2,10E-10 | 4,459 |
| septin 1 | SEPT1 | 3,66E-11 | 4,444 |
| T cell receptor alpha constant | Trac | 5,17E-11 | 4,42 |
| aldolase, fructose-bisphosphate C | ALDOC | 1,12E-08 | 4,417 |
| purinergic receptor P2X 7 | P2RX7 | 2,67E-08 | 4,413 |
| intercellular adhesion molecule 1 | ICAM1 | 2,35E-09 | 4,396 |
| microsomal glutathione S-transferase 2 | MGST2 | 2,35E-09 | 4,384 |
| apolipoprotein B mRNA editing enzyme catalytic subunit 1 | APOBEC1 | 2,22E-07 | 4,382 |
| DNA damage inducible transcript 3 | DDIT3 | 8,38E-11 | 4,371 |
| cell division cycle associated 5 | CDCA5 | 3,29E-08 | 4,369 |
| poly(ADP-ribose) polymerase family member 9 | PARP9 | 9,07E-10 | 4,364 |
| protein tyrosine kinase 2 beta | PTK2B | 8,42E-09 | 4,344 |
| BH3 interacting domain death agonist | BID | 2,65E-10 | 4,337 |
| RAB19, member RAS oncogene family | RAB19 | 1,32E-09 | 4,314 |
| T cell receptor beta, variable 16 | Trbv16 | 3,32E-07 | 4,31 |
| histone cluster 1 H2A family member g | HIST1H2AG | 1,41E-09 | 4,307 |
| deltex E3 ubiquitin ligase 3L | DTX3L | 8,42E-09 | 4,297 |
| interleukin 21 | IL21 | 7,89E-06 | 4,288 |
| G protein-coupled receptor 18 | GPR18 | 2,56E-10 | 4,273 |
| 2'-5' oligoadenylate synthetase-like 2 | Oasl2 | 1,08E-05 | 4,262 |
| ATP binding cassette subfamily C member 3 | ABCC3 | 2,93E-09 | 4,261 |
| RNA binding motif protein 47 | RBM47 | 5,44E-09 | 4,246 |
| CD300e molecule | CD300E | 1,08E-04 | 4,243 |
| T-cell immune regulator 1, ATPase H+ transporting V0 subunit a3 | TCIRG1 | 1,30E-09 | 4,235 |
| uridine phosphorylase 1 | UPP1 | 4,52E-06 | 4,228 |
| solute carrier family 13 member 3 | SLC13A3 | 4,86E-09 | 4,221 |
| activating transcription factor 7 | ATF7 | 3,67E-09 | 4,208 |
| neutrophil cytosolic factor 2 | NCF2 | 2,00E-08 | 4,205 |
| FGR proto-oncogene, Src family tyrosine kinase | FGR | 1,55E-08 | 4,201 |
| ATPase phospholipid transporting 8B4 (putative) | ATP8B4 | 3,15E-09 | 4,193 |
| pyrimidinergic receptor P2Y6 | P2RY6 | 8,97E-09 | 4,185 |
| secreted frizzled related protein 2 | SFRP2 | 9,18E-07 | 4,184 |
| major histocompatibility complex, class II, DO alpha | HLA-DOA | 7,66E-10 | 4,172 |
| major histocompatibility complex, class I, A | HLA-A | 1,52E-05 | 4,164 |
| lymphocyte antigen 9 | LY9 | 1,74E-07 | 4,156 |
| SP140 nuclear body protein | SP140 | 6,96E-09 | 4,152 |
| src kinase associated phosphoprotein 2 | SKAP2 | 3,24E-09 | 4,149 |
| T cell receptor beta, variable 13-3 | Trbv13-3 | 1,17E-06 | 4,144 |
| NLR family apoptosis inhibitory protein | NAIP | 1,31E-09 | 4,14 |
| plasminogen activator, urokinase receptor | PLAUR | 2,08E-07 | 4,139 |
| Lck interacting transmembrane adaptor 1 | LIME1 | 2,20E-09 | 4,132 |
| C-X3-C motif chemokine receptor 1 | CX3CR1 | 1,08E-03 | 4,122 |
| ring finger protein 149 | RNF149 | 5,06E-05 | 4,12 |
| family with sequence similarity 107 member B | FAM107B | 6,15E-10 | 4,119 |
| NLR family, pyrin domain containing 1A | Nlrp1a | 8,45E-10 | 4,112 |
| GLI pathogenesis related 2 | GLIPR2 | 6,92E-09 | 4,101 |
| hepatitis A virus cellular receptor 2 | HAVCR2 | 1,12E-08 | 4,093 |
| protein kinase C beta | PRKCB | 4,02E-09 | 4,092 |
| cell division cycle 20 | CDC20 | 1,09E-08 | 4,076 |
| cyclin B1 | CCNB1 | 4,19E-06 | 4,071 |
| runt related transcription factor 1 | RUNX1 | 1,35E-05 | 4,064 |
| colony stimulating factor 2 receptor alpha subunit | CSF2RA | 6,28E-10 | 4,055 |
| nucleolar and spindle associated protein 1 | NUSAP1 | 9,90E-10 | 4,054 |
| beta-ureidopropionase 1 | UPB1 | 9,07E-10 | 4,047 |
| insulin like 6 | INSL6 | 3,13E-08 | 4,035 |
| B-cell CLL/lymphoma 3 | BCL3 | 8,77E-08 | 4,03 |
| ubiquitin like modifier activating enzyme 7 | UBA7 | 6,32E-09 | 3,983 |
| nucleoporin 210 | NUP210 | 1,85E-09 | 3,975 |
| RNA binding motif (RNP1, RRM) protein 3 | RBM3 | 1,27E-06 | 3,959 |
| early growth response 2 | EGR2 | 1,74E-07 | 3,957 |
| transporter 2, ATP binding cassette subfamily B member | TAP2 | 7,59E-10 | 3,955 |
| C-type lectin domain family 4 member C | CLEC4C | 4,38E-07 | 3,94 |
| arrestin domain containing 4 | ARRDC4 | 1,30E-04 | 3,933 |
| tumor necrosis factor superfamily member 14 | TNFSF14 | 5,21E-10 | 3,927 |
| zinc finger CCCH-type containing 12D | ZC3H12D | 1,45E-07 | 3,919 |
| SAM domain, SH3 domain and nuclear localization signals 1 | SAMSN1 | 4,49E-08 | 3,913 |
| C-type lectin domain family 9 member A | CLEC9A | 1,34E-07 | 3,9 |
| ubiquitin associated and SH3 domain containing B | UBASH3B | 1,07E-06 | 3,892 |
| SPC25, NDC80 kinetochore complex component | SPC25 | 1,80E-08 | 3,888 |
| p21 (RAC1) activated kinase 1 | PAK1 | 3,16E-10 | 3,871 |
| toll like receptor 1 | TLR1 | 1,40E-07 | 3,87 |
| CD86 molecule | CD86 | 4,35E-10 | 3,865 |
| component of Sp100-rs | Csprs (includes others) | 9,71E-06 | 3,855 |
| SH2 domain containing 1A | SH2D1A | 1,50E-08 | 3,851 |
| xanthine dehydrogenase | XDH | 7,84E-08 | 3,85 |
| natriuretic peptide type B | Nppb | 3,78E-02 | 3,846 |
| tripartite motif containing 59 | TRIM59 | 3,11E-09 | 3,831 |
| tripartite motif containing 21 | TRIM21 | 1,13E-09 | 3,83 |
| vimentin | VIM | 1,69E-07 | 3,827 |
| serglycin | Srgn | 2,94E-08 | 3,82 |
| programmed cell death 1 ligand 2 | PDCD1LG2 | 5,36E-06 | 3,813 |
| ribosomal protein S6 kinase A1 | RPS6KA1 | 2,48E-10 | 3,808 |
| cell adhesion molecule 1 | CADM1 | 4,59E-10 | 3,806 |
| signal transducer and activator of transcription 4 | STAT4 | 5,28E-10 | 3,785 |
| radical S-adenosyl methionine domain containing 2 | RSAD2 | 3,37E-06 | 3,779 |
| nuclear protein 1, transcriptional regulator | NUPR1 | 6,25E-07 | 3,771 |
| lymphocyte antigen 6 complex, locus A | Ly6a (includes others) | 6,18E-11 | 3,77 |
| membrane-spanning 4-domains, subfamily A, member 4B | Ms4a4b (includes others) | 9,42E-10 | 3,762 |
| neuralized E3 ubiquitin protein ligase 3 | NEURL3 | 5,88E-07 | 3,761 |
| chloride intracellular channel 1 | CLIC1 | 7,91E-08 | 3,758 |
| POU domain, class 3, transcription factor 1 | Pou3f1 | 4,75E-08 | 3,746 |
| aldehyde dehydrogenase 1 family member A2 | ALDH1A2 | 4,93E-06 | 3,744 |
| transmembrane protein 106A | TMEM106A | 2,51E-07 | 3,742 |
| GTPase, very large interferon inducible 1 | Gvin1 (includes others) | 8,37E-05 | 3,734 |
| signal regulatory protein alpha | SIRPA | 1,33E-07 | 3,72 |
| gasdermin D | GSDMD | 1,89E-11 | 3,718 |
| ubiquitin like with PHD and ring finger domains 1 | UHRF1 | 7,00E-07 | 3,718 |
| complement factor properdin | CFP | 4,49E-07 | 3,715 |
| poly(ADP-ribose) polymerase family member 10 | PARP10 | 8,15E-11 | 3,713 |
| regulator of G-protein signaling 16 | RGS16 | 3,44E-06 | 3,703 |
| epithelial membrane protein 3 | EMP3 | 2,13E-07 | 3,699 |
| cytochrome b-245 alpha chain | CYBA | 7,59E-10 | 3,694 |
| mucolipin 2 | MCOLN2 | 3,46E-06 | 3,69 |
| BCL2 like 14 | BCL2L14 | 8,26E-11 | 3,686 |
| LY6/PLAUR domain containing 6B | LYPD6B | 2,42E-09 | 3,68 |
| PQ loop repeat containing 3 | PQLC3 | 4,29E-08 | 3,675 |
| absent in melanoma 1 | AIM1 | 6,88E-10 | 3,67 |
| alanyl aminopeptidase, membrane | ANPEP | 1,98E-07 | 3,669 |
| pentraxin 3 | PTX3 | 2,03E-04 | 3,66 |
| abnormal spindle microtubule assembly | ASPM | 6,54E-09 | 3,658 |
| tripartite motif-containing 30A | Trim30a/Trim30d | 1,94E-08 | 3,649 |
| thymosin, beta 4, X chromosome | Tmsb4x (includes others) | 1,63E-07 | 3,645 |
| CD40 molecule | CD40 | 1,30E-07 | 3,643 |
| paired immunoglobin-like type 2 receptor beta | PILRB | 2,38E-09 | 3,639 |
| phosphoinositide-3-kinase regulatory subunit 5 | PIK3R5 | 3,97E-09 | 3,635 |
| syntaxin 11 | STX11 | 9,87E-07 | 3,627 |
| cysteinyl leukotriene receptor 2 | CYSLTR2 | 6,24E-09 | 3,619 |
| cytokine receptor-like factor 2 | CRLF2 | 5,61E-09 | 3,612 |
| signaling threshold regulating transmembrane adaptor 1 | SIT1 | 1,45E-10 | 3,608 |
| CD200 receptor 1 like | CD200R1L | 1,02E-07 | 3,601 |
| adenylate cyclase 7 | ADCY7 | 4,34E-06 | 3,588 |
| predicted gene 8995 | Gm8995 | 2,97E-04 | 3,585 |
| Src like adaptor 2 | SLA2 | 5,18E-09 | 3,582 |
| C-type lectin domain family 10 member A | CLEC10A | 1,21E-09 | 3,57 |
| phosphoglycerate dehydrogenase | PHGDH | 3,77E-08 | 3,56 |
| DENN domain containing 2D | DENND2D | 4,41E-11 | 3,557 |
| gap junction protein delta 3 | GJD3 | 5,02E-08 | 3,556 |
| purinergic receptor P2Y10 | P2RY10 | 2,28E-09 | 3,556 |
| potassium two pore domain channel subfamily K member 13 | KCNK13 | 8,10E-09 | 3,553 |
| ring finger protein 213 | RNF213 | 2,33E-07 | 3,553 |
| CD300 molecule like family member d | CD300LD | 2,69E-07 | 3,552 |
| TGFB induced factor homeobox 1 | TGIF1 | 5,03E-09 | 3,552 |
| CD44 molecule (Indian blood group) | CD44 | 2,84E-06 | 3,549 |
| shugoshin 1 | SGO1 | 6,99E-09 | 3,543 |
| tryptophanyl-tRNA synthetase | WARS | 4,90E-10 | 3,524 |
| fucosyltransferase 7 | FUT7 | 5,76E-09 | 3,521 |
| mitochondrial calcium uniporter dominant negative beta subunit | MCUB | 9,69E-08 | 3,518 |
| high mobility group box 2 | HMGB2 | 8,56E-06 | 3,511 |
| macrophage scavenger receptor 1 | MSR1 | 7,31E-09 | 3,508 |
| TPX2, microtubule nucleation factor | TPX2 | 1,97E-08 | 3,502 |
| apolipoprotein E | APOE | 1,85E-10 | 3,501 |
| CCAAT/enhancer binding protein alpha | CEBPA | 7,05E-08 | 3,501 |
| protein kinase C delta | PRKCD | 1,18E-09 | 3,496 |
| signal transducer and activator of transcription 2 | STAT2 | 8,34E-10 | 3,479 |
| E2F transcription factor 1 | E2F1 | 2,71E-07 | 3,463 |
| glucose-6-phosphate dehydrogenase | G6PD | 4,43E-11 | 3,454 |
| glycine amidinotransferase | GATM | 2,90E-07 | 3,452 |
| lymphocyte-specific protein 1 | LSP1 | 6,41E-10 | 3,44 |
| lipocalin 2 | LCN2 | 3,41E-03 | 3,438 |
| aurora kinase A | AURKA | 3,06E-07 | 3,437 |
| methylenetetrahydrofolate dehydrogenase (NADP+ dependent) 1-like | MTHFD1L | 3,99E-06 | 3,427 |
| DNA damage regulated autophagy modulator 1 | DRAM1 | 7,77E-08 | 3,424 |
| colony stimulating factor 1 receptor | CSF1R | 1,70E-07 | 3,421 |
| Ras and Rab interactor like | RINL | 3,93E-08 | 3,418 |
| regulator of G-protein signaling 10 | RGS10 | 3,52E-07 | 3,416 |
| C-C motif chemokine ligand 1 | CCL1 | 1,10E-06 | 3,413 |
| ankyrin repeat domain 1 | ANKRD1 | 2,85E-05 | 3,412 |
| NPC intracellular cholesterol transporter 2 | NPC2 | 6,67E-09 | 3,404 |
| translocator protein | TSPO | 1,08E-09 | 3,399 |
| methylenetetrahydrofolate dehydrogenase (NADP+ dependent) 2, methenyltetrahydrofolate cyclohydrolase | MTHFD2 | 3,89E-08 | 3,392 |
| perforin 1 | PRF1 | 9,29E-09 | 3,386 |
| chromatin licensing and DNA replication factor 1 | CDT1 | 2,19E-05 | 3,365 |
| nitric oxide synthase 2 | NOS2 | 6,37E-05 | 3,364 |
| killer cell lectin-like receptor subfamily A, member 23 | Klra23 | 1,62E-09 | 3,361 |
| RAS p21 protein activator 4 | RASA4 | 2,64E-09 | 3,347 |
| aldehyde dehydrogenase 3 family member B1 | ALDH3B1 | 2,23E-08 | 3,345 |
| transforming acidic coiled-coil containing protein 3 | TACC3 | 1,77E-07 | 3,345 |
| spermidine/spermine N1-acetyltransferase 1 | SAT1 | 8,95E-07 | 3,323 |
| AT-rich interaction domain 5A | ARID5A | 1,18E-04 | 3,317 |
| dickkopf WNT signaling pathway inhibitor 3 | DKK3 | 8,43E-08 | 3,304 |
| brain abundant membrane attached signal protein 1 | BASP1 | 8,37E-07 | 3,296 |
| Bruton tyrosine kinase | BTK | 2,28E-09 | 3,29 |
| CNDP dipeptidase 2 (metallopeptidase M20 family) | CNDP2 | 8,42E-09 | 3,272 |
| SH3 domain binding protein 1 | SH3BP1 | 2,01E-07 | 3,264 |
| MYB proto-oncogene like 2 | MYBL2 | 1,45E-07 | 3,263 |
| phospholipase A1 member A | PLA1A | 3,32E-07 | 3,257 |
| serpin family G member 1 | SERPING1 | 1,03E-09 | 3,257 |
| ATPase Na+/K+ transporting subunit alpha 3 | ATP1A3 | 2,58E-05 | 3,236 |
| interferon induced protein 44 | IFI44 | 3,18E-07 | 3,236 |
| DExD/H-box helicase 58 | DDX58 | 1,45E-08 | 3,231 |
| phosphoglycerate dehydrogenase | PHGDH | 8,69E-08 | 3,219 |
| Rho family GTPase 1 | RND1 | 7,27E-07 | 3,216 |
| serine/threonine kinase 17b | STK17B | 4,23E-07 | 3,213 |
| IQ motif containing GTPase activating protein 3 | IQGAP3 | 1,12E-07 | 3,198 |
| chloride channel accessory 1 | CLCA1 | 5,29E-05 | 3,194 |
| interleukin 3 receptor subunit alpha | IL3RA | 7,34E-08 | 3,183 |
| TAP binding protein | TAPBP | 2,74E-08 | 3,181 |
| adenylate cyclase associated protein 1 | CAP1 | 4,29E-08 | 3,174 |
| 2'-5' oligoadenylate synthetase 1D | Oas1d (includes others) | 5,04E-08 | 3,174 |
| GTPase, IMAP family member 4 | GIMAP4 | 6,28E-05 | 3,172 |
| proteasome activator subunit 2 | PSME2 | 1,72E-10 | 3,171 |
| T cell receptor beta, variable 14 | Trbv14 | 3,22E-05 | 3,169 |
| minichromosome maintenance complex component 5 | MCM5 | 1,20E-07 | 3,163 |
| SLC9A3 regulator 1 | SLC9A3R1 | 5,70E-08 | 3,159 |
| cystatin B | CSTB | 7,83E-07 | 3,151 |
| interleukin 6 | IL6 | 2,08E-04 | 3,148 |
| shisa family member 5 | SHISA5 | 8,48E-10 | 3,133 |
| T cell receptor beta, variable 29 | Trbv29 | 9,99E-05 | 3,125 |
| protein tyrosine phosphatase, receptor type, f polypeptide (PTPRF), interacting protein (liprin), alpha 4 | Ppfia4 | 4,65E-07 | 3,121 |
| TNF alpha induced protein 2 | TNFAIP2 | 7,04E-06 | 3,119 |
| extra spindle pole bodies like 1, separase | ESPL1 | 2,51E-10 | 3,115 |
| periostin | POSTN | 4,12E-05 | 3,115 |
| non-SMC condensin I complex subunit H | NCAPH | 8,65E-06 | 3,104 |
| adaptor related protein complex 1 sigma 3 subunit | AP1S3 | 1,17E-09 | 3,103 |
| FK506 binding protein 1B | FKBP1B | 1,93E-06 | 3,097 |
| interleukin 17 receptor A | IL17RA | 8,29E-06 | 3,096 |
| myosin heavy chain 7 | MYH7 | 7,82E-04 | 3,096 |
| T cell receptor beta, variable 15 | Trbv15 | 2,03E-04 | 3,088 |
| tumor necrosis factor superfamily member 10 | TNFSF10 | 1,71E-05 | 3,085 |
| G protein-coupled receptor 84 | GPR84 | 2,53E-05 | 3,077 |
| selectin L | SELL | 5,46E-04 | 3,076 |
| leukocyte specific transcript 1 | Lst1 | 1,81E-06 | 3,074 |
| transmembrane protein 229B | TMEM229B | 2,00E-07 | 3,066 |
| complement C5a receptor 1 | C5AR1 | 5,90E-08 | 3,062 |
| RAB43, member RAS oncogene family | RAB43 | 8,96E-07 | 3,062 |
| interferon induced protein 35 | IFI35 | 2,37E-10 | 3,057 |
| B-cell CLL/lymphoma 11B | BCL11B | 5,18E-09 | 3,056 |
| uncoupling protein 2 | UCP2 | 3,27E-05 | 3,055 |
| anillin actin binding protein | ANLN | 2,25E-09 | 3,043 |
| BTG anti-proliferation factor 1 | BTG1 | 3,42E-07 | 3,031 |
| killer cell lectin like receptor B1 | KLRB1 | 3,64E-08 | 3,029 |
| actin related protein 2/3 complex subunit 1B | ARPC1B | 5,40E-08 | 3,028 |
| protein tyrosine phosphatase, receptor type O | PTPRO | 2,16E-07 | 3,022 |
| phospholipase A2 group IVA | PLA2G4A | 3,48E-07 | 3,014 |
| DEF6, guanine nucleotide exchange factor | DEF6 | 1,84E-08 | 3,013 |
| cytochrome P450 family 4 subfamily V member 2 | CYP4V2 | 3,26E-09 | 3,011 |
| synaptotagmin like 1 | SYTL1 | 9,02E-09 | 3,007 |
| cathepsin H | CTSH | 1,69E-09 | 3,005 |
| fibronectin type III domain containing 1 | Fndc1 | 1,53E-04 | 3 |
| mixed lineage kinase domain like pseudokinase | MLKL | 5,12E-08 | 2,998 |
| family with sequence similarity 46 member B | FAM46B | 2,82E-02 | 2,997 |
| GEM interacting protein | GMIP | 4,75E-08 | 2,997 |
| latent transforming growth factor beta binding protein 2 | LTBP2 | 2,46E-04 | 2,996 |
| acyl-CoA synthetase bubblegum family member 1 | ACSBG1 | 7,26E-06 | 2,995 |
| MER proto-oncogene, tyrosine kinase | MERTK | 8,36E-07 | 2,994 |
| interleukin 6 receptor | IL6R | 3,37E-07 | 2,989 |
| RB transcriptional corepressor like 1 | RBL1 | 1,89E-08 | 2,985 |
| tripartite motif containing 14 | TRIM14 | 1,11E-07 | 2,985 |
| granulin precursor | GRN | 3,15E-08 | 2,984 |
| lymphocyte antigen 6 complex pseudogene | 9030619P08Rik | 1,63E-08 | 2,983 |
| S100 calcium binding protein A6 | S100A6 | 5,13E-08 | 2,981 |
| chemokine (C-C motif) ligand 6 | Ccl6 | 1,09E-06 | 2,98 |
| major histocompatibility complex, class II, DM beta | HLA-DMB | 1,85E-07 | 2,974 |
| IKAROS family zinc finger 1 | IKZF1 | 1,02E-09 | 2,973 |
| olfactory receptor family 51 subfamily A member 7 | OR51A7 | 3,47E-09 | 2,967 |
| phospholipase C gamma 2 | PLCG2 | 7,03E-08 | 2,964 |
| DExH-box helicase 58 | DHX58 | 2,61E-06 | 2,96 |
| calcium/calmodulin dependent protein kinase ID | CAMK1D | 1,35E-08 | 2,959 |
| complement C3a receptor 1 | C3AR1 | 1,12E-08 | 2,957 |
| UDP glucuronosyltransferase family 1 member A10 | UGT1A7 (includes others) | 1,50E-04 | 2,957 |
| family with sequence similarity 64 member A | FAM64A | 4,21E-07 | 2,956 |
| kelch repeat and BTB domain containing 11 | KBTBD11 | 5,38E-07 | 2,956 |
| TNF receptor superfamily member 1B | TNFRSF1B | 8,23E-09 | 2,95 |
| minichromosome maintenance complex component 6 | MCM6 | 2,67E-09 | 2,937 |
| C-X-C motif chemokine receptor 4 | CXCR4 | 5,02E-08 | 2,934 |
| regulator of G-protein signaling 19 | RGS19 | 9,39E-07 | 2,933 |
| ribonuclease A family member k6 | RNASE6 | 1,31E-07 | 2,931 |
| phosphoinositide-3-kinase adaptor protein 1 | PIK3AP1 | 1,30E-07 | 2,927 |
| G protein subunit alpha 13 | GNA13 | 1,17E-03 | 2,926 |
| solute carrier family 2 member 3 | SLC2A3 | 1,68E-07 | 2,917 |
| legumain | LGMN | 8,93E-07 | 2,916 |
| transmembrane protein 140 | TMEM140 | 1,26E-06 | 2,914 |
| RAB8B, member RAS oncogene family | RAB8B | 1,56E-08 | 2,913 |
| WD repeat domain 92 | WDR92 | 1,76E-03 | 2,913 |
| stefin A2 | Stfa2/Stfa2l1 | 1,11E-02 | 2,909 |
| transient receptor potential cation channel subfamily V member 2 | TRPV2 | 1,27E-08 | 2,904 |
| inhibitor of kappa light polypeptide gene enhancer in B-cells, kinase epsilon | IKBKE | 1,55E-08 | 2,898 |
| inner centromere protein | INCENP | 7,87E-08 | 2,897 |
| F-box and WD-40 domain protein 17 | Fbxw17 | 1,36E-08 | 2,893 |
| lipopolysaccharide induced TNF factor | LITAF | 7,75E-08 | 2,89 |
| keratin 85 | Krt85 | 2,26E-05 | 2,887 |
| interleukin 18 receptor accessory protein | IL18RAP | 1,33E-05 | 2,883 |
| interleukin 7 receptor | IL7R | 2,23E-09 | 2,883 |
| mannosidase alpha class 2B member 1 | MAN2B1 | 2,89E-09 | 2,876 |
| cyclin F | CCNF | 1,90E-08 | 2,873 |
| phosphatidylinositol-4,5-bisphosphate 3-kinase catalytic subunit gamma | PIK3CG | 9,05E-09 | 2,87 |
| collapsin response mediator protein 1 | CRMP1 | 1,24E-08 | 2,867 |
| docking protein 3 | DOK3 | 1,29E-07 | 2,867 |
| ras homolog family member G | RHOG | 4,09E-09 | 2,859 |
| CD37 molecule | CD37 | 2,69E-07 | 2,858 |
| interferon induced with helicase C domain 1 | IFIH1 | 2,99E-08 | 2,856 |
| LFNG O-fucosylpeptide 3-beta-N-acetylglucosaminyltransferase | LFNG | 1,19E-07 | 2,856 |
| chloride channel accessory 2 | CLCA2 | 6,15E-05 | 2,854 |
| BUB1 mitotic checkpoint serine/threonine kinase B | BUB1B | 3,32E-08 | 2,853 |
| Ras association domain family member 2 | RASSF2 | 5,19E-06 | 2,853 |
| ORAI calcium release-activated calcium modulator 2 | ORAI2 | 3,87E-06 | 2,852 |
| CD226 molecule | CD226 | 9,02E-08 | 2,851 |
| GM2 ganglioside activator | GM2A | 3,97E-09 | 2,849 |
| serine carboxypeptidase 1 | SCPEP1 | 1,56E-08 | 2,841 |
| matrix metallopeptidase 14 | MMP14 | 7,85E-06 | 2,836 |
| actin beta | ACTB | 7,95E-08 | 2,835 |
| phosphoserine aminotransferase 1 | PSAT1 | 1,10E-06 | 2,835 |
| predicted gene 6377 | Gm6377 | 1,12E-07 | 2,825 |
| killer cell lectin like receptor B1 | KLRB1 | 3,43E-08 | 2,822 |
| hydroxysteroid 11-beta dehydrogenase 1 | HSD11B1 | 1,21E-05 | 2,815 |
| BCL2 antagonist/killer 1 | BAK1 | 9,42E-08 | 2,813 |
| CD82 molecule | CD82 | 7,49E-08 | 2,813 |
| podocan like 1 | PODNL1 | 2,98E-07 | 2,811 |
| C-X-C motif chemokine ligand 2 | CXCL2 | 2,56E-05 | 2,802 |
| fibronectin 1 | FN1 | 1,22E-04 | 2,794 |
| CD160 molecule | CD160 | 1,46E-07 | 2,793 |
| protein tyrosine phosphatase, receptor type J | PTPRJ | 3,74E-07 | 2,793 |
| lymphoid enhancer binding factor 1 | LEF1 | 4,55E-07 | 2,786 |
| schlafen 1 | Slfn1 | 6,65E-07 | 2,777 |
| killer cell lectin-like receptor subfamily B member 1F | Klrb1f | 1,50E-06 | 2,776 |
| IQ motif containing GTPase activating protein 1 | IQGAP1 | 1,13E-07 | 2,768 |
| FES proto-oncogene, tyrosine kinase | FES | 2,95E-08 | 2,767 |
| IKAROS family zinc finger 1 | IKZF1 | 6,73E-08 | 2,767 |
| transmembrane protein 51 | TMEM51 | 2,25E-06 | 2,767 |
| thyroid hormone receptor interactor 13 | TRIP13 | 1,90E-06 | 2,766 |
| proline rich 5 like | PRR5L | 7,44E-06 | 2,761 |
| interleukin 4 receptor | IL4R | 4,07E-07 | 2,754 |
| interleukin 18 | IL18 | 8,08E-07 | 2,75 |
| spindle and kinetochore associated complex subunit 1 | SKA1 | 1,50E-06 | 2,748 |
| glutathione peroxidase 1 | GPX1 | 4,75E-08 | 2,746 |
| MARCKS like 1 | MARCKSL1 | 1,80E-05 | 2,742 |
| unc-5 family C-terminal like | UNC5CL | 3,50E-07 | 2,742 |
| acyl-CoA synthetase long-chain family member 5 | ACSL5 | 8,53E-10 | 2,733 |
| family with sequence similarity 46 member C | FAM46C | 1,95E-06 | 2,73 |
| phosphotyrosine interaction domain containing 1 | PID1 | 1,45E-08 | 2,724 |
| chemokine (C-C motif) ligand 9 | Ccl9 | 9,31E-06 | 2,721 |
| lymphocyte antigen 6 complex, locus E | LY6E | 2,02E-08 | 2,72 |
| tropomyosin 3 | TPM3 | 3,88E-08 | 2,717 |
| selectin P | SELP | 1,31E-04 | 2,715 |
| carcinoembryonic antigen related cell adhesion molecule 16 | CEACAM16 | 2,44E-08 | 2,714 |
| eukaryotic translation initiation factor 2 alpha kinase 2 | EIF2AK2 | 3,68E-09 | 2,709 |
| toll like receptor 6 | TLR6 | 3,64E-07 | 2,707 |
| chromosome X open reading frame 21 | CXorf21 | 3,23E-09 | 2,704 |
| receptor interacting serine/threonine kinase 1 | RIPK1 | 2,03E-09 | 2,703 |
| cyclin dependent kinase inhibitor 2A | CDKN2A | 6,59E-07 | 2,699 |
| histone cluster 2 H2B family member f | HIST2H2BF | 1,73E-05 | 2,699 |
| ZFP36 ring finger protein like 2 | ZFP36L2 | 6,86E-07 | 2,698 |
| CKLF like MARVEL transmembrane domain containing 7 | CMTM7 | 1,55E-08 | 2,696 |
| cytochrome P450 family 1 subfamily B member 1 | CYP1B1 | 2,29E-03 | 2,696 |
| Rho GTPase activating protein 25 | ARHGAP25 | 6,80E-07 | 2,689 |
| interferon induced transmembrane protein 1 | Ifitm1 | 9,99E-05 | 2,685 |
| T cell receptor beta, variable 12-2 | Trbv12-2 | 3,00E-08 | 2,681 |
| extracellular matrix protein 1 | ECM1 | 1,82E-07 | 2,674 |
| adenosine deaminase, RNA specific | ADAR | 5,95E-09 | 2,672 |
| SH2 domain containing 2A | SH2D2A | 1,35E-05 | 2,672 |
| dipeptidase 1 (renal) | DPEP1 | 2,70E-05 | 2,663 |
| transmembrane 6 superfamily member 1 | TM6SF1 | 4,35E-06 | 2,659 |
| AXL receptor tyrosine kinase | AXL | 2,80E-08 | 2,657 |
| T cell receptor beta, variable 5 | Trbv5 | 5,48E-07 | 2,65 |
| carboxypeptidase X, M14 family member 1 | CPXM1 | 5,70E-05 | 2,649 |
| tetraspanin 33 | TSPAN33 | 9,31E-08 | 2,648 |
| pleckstrin homology domain containing O2 | PLEKHO2 | 6,70E-07 | 2,647 |
| dedicator of cytokinesis 11 | DOCK11 | 1,85E-07 | 2,646 |
| adenosine deaminase | ADA | 2,63E-06 | 2,642 |
| natriuretic peptide A | NPPA | 8,34E-06 | 2,635 |
| CD5 molecule like | CD5L | 1,16E-03 | 2,634 |
| transmembrane protein 128 | TMEM128 | 2,90E-03 | 2,632 |
| fidgetin like 1 | FIGNL1 | 3,22E-05 | 2,63 |
| serine peptidase inhibitor, Kunitz type 1 | SPINT1 | 1,66E-08 | 2,627 |
| granzyme C | Gzmc | 1,18E-05 | 2,622 |
| multiple C2 and transmembrane domain containing 2 | MCTP2 | 4,26E-05 | 2,622 |
| G protein-coupled receptor 174 | GPR174 | 3,19E-08 | 2,621 |
| GTPase, IMAP family member 9 | Gimap9 | 1,23E-05 | 2,618 |
| aspartic peptidase, retroviral-like 1 | ASPRV1 | 1,18E-02 | 2,614 |
| T cell receptor beta, variable 10 | Trbv4 | 2,07E-05 | 2,614 |
| ferritin light chain | FTL | 3,99E-08 | 2,607 |
| F-box protein 5 | FBXO5 | 1,25E-07 | 2,603 |
| high mobility group AT-hook 2, pseudogene 1 | Hmga2-ps1 | 2,89E-06 | 2,602 |
| TXK tyrosine kinase | TXK | 1,31E-05 | 2,602 |
| NIMA related kinase 6 | NEK6 | 4,09E-06 | 2,599 |
| transcriptional regulating factor 1 | TRERF1 | 5,85E-06 | 2,599 |
| signaling lymphocytic activation molecule family member 1 | SLAMF1 | 6,55E-08 | 2,597 |
| SHC binding and spindle associated 1 | SHCBP1 | 4,05E-06 | 2,59 |
| NLR family, apoptosis inhibitory protein 1 | Naip1 (includes others) | 6,96E-07 | 2,589 |
| actin related protein 2/3 complex subunit 5 | ARPC5 | 4,07E-07 | 2,584 |
| cyclin dependent kinase 1 | CDK1 | 7,36E-07 | 2,584 |
| proline rich protein HaeIII subfamily 1 | Prh1/Prp2 | 3,86E-06 | 2,571 |
| N-myc and STAT interactor | NMI | 4,78E-09 | 2,569 |
| annexin A4 | ANXA4 | 3,46E-08 | 2,567 |
| sperm associated antigen 5 | SPAG5 | 8,50E-07 | 2,566 |
| TBC1 domain family member 9 | TBC1D9 | 1,18E-08 | 2,566 |
| solute carrier organic anion transporter family member 4A1 | SLCO4A1 | 5,31E-07 | 2,564 |
| sphingomyelin phosphodiesterase acid like 3B | SMPDL3B | 1,28E-05 | 2,557 |
| signal transducer and activator of transcription 1 | STAT1 | 4,10E-07 | 2,554 |
| MAF bZIP transcription factor B | MAFB | 2,23E-07 | 2,552 |
| interleukin 1 beta | IL1B | 7,53E-03 | 2,551 |
| lamin B1 | LMNB1 | 2,15E-05 | 2,55 |
| T cell receptor associated transmembrane adaptor 1 | TRAT1 | 2,83E-06 | 2,546 |
| annexin A1 | ANXA1 | 1,11E-05 | 2,544 |
| killer cell lectin like receptor B1 | KLRB1 | 1,74E-07 | 2,538 |
| SH2 domain containing 1B | SH2D1B | 2,77E-07 | 2,538 |
| ER degradation enhancing alpha-mannosidase like protein 1 | EDEM1 | 3,10E-06 | 2,536 |
| GRB2-related adaptor protein 2 | GRAP2 | 1,76E-06 | 2,533 |
| transketolase | TKT | 5,90E-08 | 2,531 |
| mesothelin | MSLN | 1,31E-03 | 2,53 |
| archaelysin family metallopeptidase 1 | AMZ1 | 6,94E-05 | 2,526 |
| prolylcarboxypeptidase | PRCP | 1,98E-06 | 2,525 |
| proteasome activator subunit 1 | PSME1 | 6,24E-09 | 2,525 |
| family with sequence similarity 167 member B | FAM167B | 3,83E-05 | 2,524 |
| minichromosome maintenance complex component 3 | MCM3 | 1,23E-06 | 2,524 |
| microfibrillar associated protein 5 | MFAP5 | 7,87E-05 | 2,517 |
| kinesin family member C1 | KIFC1 | 1,56E-05 | 2,511 |
| RAB5C, member RAS oncogene family | RAB5C | 5,29E-06 | 2,503 |
| TNF receptor superfamily member 11b | TNFRSF11B | 3,13E-06 | 2,494 |
| cyclin E1 | CCNE1 | 2,97E-05 | 2,488 |
| major vault protein | MVP | 1,74E-06 | 2,487 |
| protein tyrosine phosphatase, receptor type E | PTPRE | 4,55E-06 | 2,483 |
| solute carrier family 7 member 8 | SLC7A8 | 4,09E-04 | 2,483 |
| solute carrier family 7 member 7 | SLC7A7 | 1,74E-07 | 2,477 |
| kelch like family member 6 | KLHL6 | 1,09E-05 | 2,473 |
| N-acylsphingosine amidohydrolase 1 | ASAH1 | 1,07E-06 | 2,467 |
| ribonucleotide reductase regulatory subunit M2 | RRM2 | 4,66E-06 | 2,467 |
| sortilin related receptor 1 | SORL1 | 2,94E-07 | 2,467 |
| cytotoxic and regulatory T-cell molecule | CRTAM | 1,15E-04 | 2,463 |
| sphingosine-1-phosphate lyase 1 | SGPL1 | 9,83E-08 | 2,463 |
| cartilage intermediate layer protein | CILP | 4,64E-05 | 2,458 |
| protein phosphatase 2 regulatory subunit Bgamma | PPP2R2C | 1,52E-05 | 2,457 |
| janus kinase and microtubule interacting protein 1 | JAKMIP1 | 3,58E-07 | 2,455 |
| phosphofructokinase, platelet | PFKP | 3,79E-09 | 2,454 |
| killer cell lectin-like receptor, subfamily A, member 4 | Klra7 (includes others) | 3,59E-04 | 2,452 |
| zinc finger CCCH-type containing, antiviral 1 | ZC3HAV1 | 2,13E-05 | 2,452 |
| thymosin, beta 4, X chromosome | Tmsb4x (includes others) | 6,99E-07 | 2,451 |
| torsin family 3 member A | TOR3A | 1,25E-07 | 2,442 |
| interferon gamma inducible protein 16 | IFI16 | 8,85E-09 | 2,441 |
| ArfGAP with dual PH domains 2 | ADAP2 | 9,03E-06 | 2,432 |
| interferon gamma inducible protein 16 | IFI16 | 2,09E-04 | 2,432 |
| lymphocyte cytosolic protein 2 | LCP2 | 2,85E-05 | 2,431 |
| SH2B adaptor protein 2 | SH2B2 | 6,00E-06 | 2,431 |
| sphingosine-1-phosphate receptor 4 | S1PR4 | 3,13E-05 | 2,429 |
| ADP ribosylation factor 3 | ARF3 | 1,22E-06 | 2,428 |
| PDZ binding kinase | PBK | 6,02E-08 | 2,425 |
| germ cell associated 2, haspin | GSG2 | 1,13E-08 | 2,421 |
| syndecan 3 | SDC3 | 1,26E-07 | 2,417 |
| transmembrane protease, serine 4 | TMPRSS4 | 2,48E-06 | 2,416 |
| renin binding protein | RENBP | 7,58E-07 | 2,415 |
| eukaryotic translation elongation factor 1 alpha 1 pseudogene | Gm6548 | 5,75E-09 | 2,413 |
| mitogen-activated protein kinase 13 | MAPK13 | 4,41E-06 | 2,413 |
| CD101 molecule | CD101 | 5,25E-06 | 2,412 |
| peptidase domain containing associated with muscle regeneration 1 | PAMR1 | 1,15E-06 | 2,408 |
| potassium voltage-gated channel subfamily J member 10 | KCNJ10 | 1,40E-06 | 2,407 |
| myelin associated glycoprotein | MAG | 2,56E-08 | 2,403 |
| NIMA related kinase 2 | NEK2 | 2,54E-07 | 2,403 |
| tubulin alpha 1c | TUBA1C | 2,93E-05 | 2,402 |
| asparagine synthetase (glutamine-hydrolyzing) | ASNS | 1,69E-05 | 2,401 |
| poly(ADP-ribose) polymerase family member 12 | PARP12 | 1,11E-06 | 2,399 |
| stress associated endoplasmic reticulum protein 1 | SERP1 | 6,04E-06 | 2,398 |
| T cell receptor beta, variable 13-1 | Trbv13-1 | 1,50E-04 | 2,396 |
| family with sequence similarity 49 member A | FAM49A | 8,82E-07 | 2,395 |
| kinesin family member 20A | KIF20A | 7,40E-07 | 2,395 |
| syndecan 4 | SDC4 | 8,71E-05 | 2,391 |
| histone cluster 1 H2A family member h | HIST1H2AH | 1,32E-07 | 2,379 |
| mannosidase alpha class 2A member 1 | MAN2A1 | 3,67E-05 | 2,379 |
| profilin 1 | PFN1 | 4,26E-06 | 2,378 |
| transmembrane protein 119 | TMEM119 | 1,04E-03 | 2,378 |
| collagen type III alpha 1 chain | COL3A1 | 5,70E-03 | 2,377 |
| UDP-N-acetylglucosamine pyrophosphorylase 1 like 1 | UAP1L1 | 1,19E-08 | 2,375 |
| myeloid differentiation primary response 88 | MYD88 | 4,29E-07 | 2,372 |
| CD38 molecule | CD38 | 2,98E-06 | 2,37 |
| zyxin | ZYX | 7,89E-06 | 2,364 |
| sterol O-acyltransferase 2 | SOAT2 | 1,76E-07 | 2,362 |
| interferon regulatory factor 9 | IRF9 | 1,89E-07 | 2,359 |
| apolipoprotein D | APOD | 2,66E-04 | 2,358 |
| solute carrier family 25 member 45 | SLC25A45 | 3,81E-07 | 2,358 |
| copine 2 | CPNE2 | 2,85E-05 | 2,355 |
| POU class 2 homeobox 2 | POU2F2 | 3,52E-05 | 2,354 |
| activating transcription factor 3 | ATF3 | 3,50E-03 | 2,35 |
| PRELI domain containing 1 | PRELID1 | 6,53E-06 | 2,348 |
| anti-silencing function 1B histone chaperone | ASF1B | 1,96E-06 | 2,344 |
| chromatin assembly factor 1 subunit B | CHAF1B | 2,34E-04 | 2,339 |
| platelet activating factor receptor | PTAFR | 2,75E-06 | 2,338 |
| ubiquitin associated and SH3 domain containing A | UBASH3A | 8,42E-09 | 2,336 |
| histone cluster 1 H2A family member a | HIST1H2AA | 1,35E-07 | 2,335 |
| centromere protein K | CENPK | 1,02E-06 | 2,331 |
| chondroitin polymerizing factor 2 | CHPF2 | 6,22E-04 | 2,327 |
| G protein-coupled receptor 141 | GPR141 | 6,58E-05 | 2,327 |
| prostaglandin-endoperoxide synthase 2 | PTGS2 | 2,80E-05 | 2,326 |
| structural maintenance of chromosomes 2 | SMC2 | 1,07E-05 | 2,326 |
| insulin receptor related receptor | INSRR | 2,88E-06 | 2,325 |
| C-C motif chemokine receptor like 2 | CCRL2 | 1,22E-07 | 2,319 |
| interferon induced transmembrane protein 3 | IFITM3 | 5,28E-07 | 2,319 |
| family with sequence similarity 189 member B | FAM189B | 1,98E-06 | 2,317 |
| microfibrillar associated protein 4 | MFAP4 | 1,97E-03 | 2,317 |
| ETS variant 6 | ETV6 | 3,39E-07 | 2,315 |
| glutaredoxin | GLRX | 2,02E-05 | 2,315 |
| thymocyte selection associated | THEMIS | 2,14E-06 | 2,312 |
| B and T lymphocyte associated | BTLA | 3,65E-07 | 2,311 |
| caspase 8 | CASP8 | 2,79E-08 | 2,311 |
| lysyl oxidase | LOX | 8,37E-03 | 2,311 |
| programmed cell death 1 | PDCD1 | 2,15E-06 | 2,31 |
| reticulon 4 | RTN4 | 6,27E-06 | 2,308 |
| potassium two pore domain channel subfamily K member 5 | KCNK5 | 4,06E-06 | 2,307 |
| lymphocyte antigen 6 complex, locus A | Ly6a (includes others) | 2,03E-06 | 2,307 |
| solute carrier family 41 member 2 | SLC41A2 | 2,48E-06 | 2,302 |
| olfactomedin 1 | OLFM1 | 3,62E-06 | 2,3 |
| glucosylceramidase beta | GBA | 1,30E-06 | 2,299 |
| keratin 18 | KRT18 | 1,06E-04 | 2,296 |
| polypeptide N-acetylgalactosaminyltransferase 12 | GALNT12 | 1,59E-08 | 2,295 |
| PYD and CARD domain containing | PYCARD | 4,36E-07 | 2,291 |
| C-type lectin domain family 2, member d | Clec2d (includes others) | 7,70E-03 | 2,29 |
| transmembrane protein 176A | TMEM176A | 1,40E-06 | 2,29 |
| piggyBac transposable element derived 5 | PGBD5 | 2,92E-06 | 2,287 |
| TNF alpha induced protein 6 | TNFAIP6 | 1,39E-05 | 2,286 |
| strawberry notch homolog 2 | SBNO2 | 5,74E-06 | 2,284 |
| regulator of chromosome condensation 2 | RCC2 | 2,34E-06 | 2,282 |
| epithelial stromal interaction 1 | EPSTI1 | 2,07E-06 | 2,279 |
| collagen type I alpha 1 chain | COL1A1 | 3,37E-03 | 2,277 |
| centromere protein H | CENPH | 6,61E-06 | 2,273 |
| serine incorporator 2 | SERINC2 | 1,45E-04 | 2,271 |
| cofilin 1 | CFL1 | 4,10E-05 | 2,27 |
| S100 calcium binding protein A11 | S100a11 | 3,11E-06 | 2,268 |
| 5', 3'-nucleotidase, cytosolic | NT5C | 1,48E-08 | 2,267 |
| eukaryotic translation elongation factor 1 alpha 1 | EEF1A1 | 1,20E-07 | 2,266 |
| cysteine rich C-terminal 1 | CRCT1 | 2,38E-04 | 2,261 |
| CD200 receptor 1 | CD200R1 | 5,75E-07 | 2,258 |
| SLAIN motif family member 1 | SLAIN1 | 1,17E-06 | 2,256 |
| peptidoglycan recognition protein 2 | PGLYRP2 | 4,63E-07 | 2,255 |
| interleukin 33 | IL33 | 1,94E-04 | 2,249 |
| pyrophosphatase (inorganic) 1 | PPA1 | 9,90E-06 | 2,249 |
| vasodilator-stimulated phosphoprotein | VASP | 1,36E-05 | 2,248 |
| RAP1B, member of RAS oncogene family | RAP1B | 1,70E-06 | 2,246 |
| sorting nexin 22 | SNX22 | 1,76E-03 | 2,243 |
| WAP four-disulfide core domain 8 | WFDC8 | 1,47E-04 | 2,24 |
| lipolysis stimulated lipoprotein receptor | LSR | 2,22E-05 | 2,239 |
| TRAF3 interacting protein 3 | TRAF3IP3 | 1,27E-06 | 2,239 |
| synaptonemal complex central element protein 2 | SYCE2 | 1,95E-05 | 2,235 |
| tubulin beta 2B class IIb | TUBB2B | 2,84E-04 | 2,235 |
| transmembrane protein 176B | TMEM176B | 8,08E-07 | 2,23 |
| 3-phosphoglycerate dehydrogenase pseudogene | Gm8096 | 6,88E-06 | 2,229 |
| synaptotagmin like 2 | SYTL2 | 2,43E-06 | 2,228 |
| C-C motif chemokine receptor 7 | CCR7 | 7,67E-05 | 2,227 |
| annexin A2 | ANXA2 | 1,01E-06 | 2,226 |
| sterile alpha motif domain containing 9 like | SAMD9L | 3,20E-05 | 2,224 |
| collagen type I alpha 2 chain | COL1A2 | 5,41E-04 | 2,22 |
| major facilitator superfamily domain containing 1 | MFSD1 | 3,41E-05 | 2,22 |
| CD247 molecule | CD247 | 1,97E-06 | 2,217 |
| membrane-spanning 4-domains, subfamily A, member 6B | Ms4a6b | 1,18E-05 | 2,216 |
| CD14 molecule | CD14 | 5,33E-03 | 2,213 |
| FERM domain containing 8 | FRMD8 | 1,65E-07 | 2,21 |
| syntaxin 3 | STX3 | 3,36E-04 | 2,209 |
| fms related tyrosine kinase 3 | FLT3 | 1,04E-06 | 2,204 |
| TNF receptor superfamily member 1A | TNFRSF1A | 1,67E-06 | 2,2 |
| CD302 molecule | CD302 | 3,87E-05 | 2,199 |
| cell division cycle 25B | CDC25B | 7,30E-07 | 2,198 |
| RAB3A interacting protein like 1 | RAB3IL1 | 5,93E-05 | 2,192 |
| TNF receptor superfamily member 12A | TNFRSF12A | 4,27E-02 | 2,191 |
| rhophilin associated tail protein 1 like | ROPN1L | 3,99E-06 | 2,19 |
| baculoviral IAP repeat containing 3 | BIRC3 | 1,16E-03 | 2,189 |
| caspase 12 | Casp12 | 4,17E-06 | 2,189 |
| protein tyrosine phosphatase, non-receptor type 1 | PTPN1 | 1,34E-05 | 2,189 |
| carbonic anhydrase 9 | CA9 | 8,02E-06 | 2,188 |
| transmembrane protein 45A | TMEM45A | 8,89E-05 | 2,188 |
| plasminogen activator, tissue type | PLAT | 1,81E-07 | 2,185 |
| chromosome 4 open reading frame 32 | C4orf32 | 1,84E-04 | 2,183 |
| polo like kinase 3 | PLK3 | 4,78E-05 | 2,179 |
| BMP2 inducible kinase | BMP2K | 9,49E-04 | 2,177 |
| leucine rich repeats and calponin homology domain containing 4 | LRCH4 | 6,94E-07 | 2,176 |
| arrestin domain containing 1 | ARRDC1 | 2,85E-06 | 2,174 |
| ceruloplasmin | CP | 5,26E-06 | 2,173 |
| non-SMC condensin II complex subunit G2 | NCAPG2 | 7,53E-08 | 2,173 |
| killer cell lectin-like receptor, subfamily A, member 4 | Klra7 (includes others) | 2,55E-06 | 2,172 |
| chromosome 7 open reading frame 43 | C7orf43 | 1,24E-06 | 2,169 |
| solute carrier family 39 member 11 | SLC39A11 | 4,59E-04 | 2,169 |
| T-cell immunoreceptor with Ig and ITIM domains | TIGIT | 3,05E-04 | 2,169 |
| prostaglandin E receptor 4 | PTGER4 | 1,09E-07 | 2,168 |
| transgelin 2 | TAGLN2 | 1,81E-05 | 2,168 |
| thrombospondin 3 | THBS3 | 3,28E-06 | 2,168 |
| adenosine A3 receptor | ADORA3 | 1,55E-05 | 2,166 |
| tumor protein D52 | TPD52 | 9,09E-07 | 2,165 |
| vitamin D (1,25- dihydroxyvitamin D3) receptor | VDR | 7,88E-04 | 2,165 |
| potassium voltage-gated channel subfamily A member 3 | KCNA3 | 3,65E-08 | 2,164 |
| Rho GTPase activating protein 22 | ARHGAP22 | 4,11E-04 | 2,163 |
| chromosome 17 open reading frame 62 | C17orf62 | 2,22E-05 | 2,163 |
| paired immunoglobin-like type 2 receptor beta | PILRB | 6,69E-05 | 2,162 |
| eukaryotic translation initiation factor 1A domain containing | EIF1AD | 2,18E-05 | 2,161 |
| meiosis specific nuclear structural 1 | MNS1 | 2,24E-06 | 2,161 |
| CDC28 protein kinase regulatory subunit 2 | CKS2 | 1,09E-07 | 2,16 |
| annexin A8-like 1 | ANXA8/ANXA8L1 | 1,53E-03 | 2,159 |
| Cas scaffolding protein family member 4 | CASS4 | 1,48E-06 | 2,159 |
| matrix metallopeptidase 19 | MMP19 | 5,58E-05 | 2,159 |
| RAD51 recombinase | RAD51 | 1,04E-06 | 2,155 |
| LYL1, basic helix-loop-helix family member | LYL1 | 3,88E-04 | 2,154 |
| 2-cell-stage, variable group, member 3 | Tcstv3 | 9,41E-06 | 2,154 |
| histone cluster 1 H2A family member j | HIST1H2AJ | 6,03E-07 | 2,151 |
| extended synaptotagmin 1 | ESYT1 | 2,78E-07 | 2,149 |
| granzyme K | GZMK | 2,93E-05 | 2,149 |
| glycolipid transfer protein | GLTP | 6,74E-07 | 2,145 |
| GATA binding protein 3 | GATA3 | 1,29E-05 | 2,144 |
| pyruvate dehydrogenase kinase 3 | PDK3 | 9,63E-07 | 2,141 |
| lymphocyte antigen 6 complex, locus G5B | LY6G5B | 2,18E-05 | 2,139 |
| T cell-interacting, activating receptor on myeloid cells 1 | TARM1 | 2,20E-03 | 2,137 |
| coronin 7 | CORO7/CORO7-PAM16 | 9,75E-06 | 2,136 |
| aldo-keto reductase family 1, member C13 | Akr1c12/Akr1c13 | 7,65E-07 | 2,133 |
| PAS domain containing serine/threonine kinase | PASK | 2,36E-04 | 2,131 |
| interleukin 1 receptor antagonist | IL1RN | 2,28E-04 | 2,13 |
| ankyrin repeat domain 44 | ANKRD44 | 1,45E-07 | 2,128 |
| activated leukocyte cell adhesion molecule | ALCAM | 1,01E-06 | 2,123 |
| tumor necrosis factor (ligand) superfamily, member 9 | Tnfsf9 | 1,76E-06 | 2,122 |
| G protein subunit gamma transducin 2 | GNGT2 | 6,04E-06 | 2,119 |
| immunoglobulin heavy constant gamma 2C | Ighg2c | 2,19E-02 | 2,119 |
| yippee like 5 | YPEL5 | 8,05E-03 | 2,118 |
| beta-1,4-galactosyltransferase 5 | B4GALT5 | 2,84E-05 | 2,114 |
| biglycan | BGN | 4,04E-04 | 2,112 |
| notch 2 | NOTCH2 | 1,06E-05 | 2,112 |
| tweety family member 3 | TTYH3 | 1,06E-03 | 2,108 |
| C-type lectin domain family 4 member C | CLEC4C | 7,59E-04 | 2,106 |
| X-linked lymphocyte-regulated 4A | Xlr4a (includes others) | 5,49E-05 | 2,106 |
| SERTA domain containing 2 | SERTAD2 | 1,60E-03 | 2,105 |
| MAF bZIP transcription factor | MAF | 5,55E-06 | 2,099 |
| ADP ribosylation factor like GTPase 6 interacting protein 1 | ARL6IP1 | 8,26E-08 | 2,097 |
| interferon induced transmembrane protein 10 | Ifitm10 | 4,54E-04 | 2,097 |
| Ras association domain family member 4 | RASSF4 | 1,81E-03 | 2,097 |
| ADP ribosylation factor 6 | ARF6 | 2,53E-06 | 2,096 |
| C-X3-C motif chemokine ligand 1 | CX3CL1 | 1,51E-04 | 2,096 |
| G protein subunit alpha 13 | GNA13 | 1,76E-06 | 2,095 |
| poly(A) binding protein cytoplasmic 1 | PABPC1 | 3,24E-06 | 2,095 |
| transmembrane protein 37 | TMEM37 | 1,71E-03 | 2,095 |
| mab-21 like 3 | MAB21L3 | 1,14E-05 | 2,094 |
| lipin 2 | LPIN2 | 2,07E-05 | 2,093 |
| secreted phosphoprotein 1 | SPP1 | 9,94E-05 | 2,093 |
| galectin 9B | LGALS9B | 5,87E-06 | 2,09 |
| cytoskeleton associated protein 2 | CKAP2 | 4,42E-06 | 2,089 |
| thymidylate synthase, pseudogene | Tyms-ps | 1,44E-05 | 2,089 |
| RNA binding motif protein 43 | RBM43 | 1,36E-07 | 2,088 |
| NLR family pyrin domain containing 1 | NLRP1 | 6,60E-06 | 2,087 |
| CREB binding protein | CREBBP | 2,31E-06 | 2,086 |
| secreted and transmembrane 1 | SECTM1 | 7,33E-07 | 2,085 |
| cytidine/uridine monophosphate kinase 2 | CMPK2 | 1,82E-05 | 2,077 |
| syndecan binding protein | SDCBP | 5,76E-05 | 2,077 |
| glutamate ionotropic receptor NMDA type subunit 2C | GRIN2C | 7,68E-05 | 2,076 |
| ABI family member 3 | ABI3 | 5,55E-04 | 2,074 |
| lymphoid enhancer binding factor 1 | LEF1 | 2,43E-06 | 2,073 |
| tetraspanin 4 | TSPAN4 | 1,60E-05 | 2,073 |
| integrin subunit beta 3 | ITGB3 | 4,65E-07 | 2,072 |
| histone cluster 2 H2A family member c | HIST2H2AC | 4,29E-07 | 2,071 |
| armadillo repeat containing 7 | ARMC7 | 4,56E-06 | 2,069 |
| family with sequence similarity 129 member A | FAM129A | 8,19E-07 | 2,068 |
| kinesin family member 20B | KIF20B | 9,78E-06 | 2,068 |
| cell division cycle associated 2 | CDCA2 | 4,35E-06 | 2,064 |
| killer cell lectin-like receptor, subfamily A, member 15 | Klra15 | 4,42E-05 | 2,064 |
| Rho GTPase activating protein 17 | ARHGAP17 | 4,54E-06 | 2,063 |
| peptidoglycan recognition protein 1 | PGLYRP1 | 1,78E-06 | 2,063 |
| calcium/calmodulin dependent protein kinase IV | CAMK4 | 2,13E-04 | 2,061 |
| hexosaminidase subunit beta | HEXB | 2,48E-06 | 2,059 |
| LIM and SH3 protein 1 | LASP1 | 7,40E-05 | 2,059 |
| actin related protein 2/3 complex subunit 4 | ARPC4 | 2,12E-05 | 2,058 |
| epithelial cell transforming 2 | ECT2 | 2,61E-06 | 2,058 |
| B-box and SPRY domain containing | BSPRY | 2,36E-06 | 2,053 |
| B-cell linker | BLNK | 3,82E-03 | 2,052 |
| retinoic acid receptor responder 2 | RARRES2 | 2,43E-05 | 2,051 |
| family with sequence similarity 111 member A | FAM111A | 6,59E-04 | 2,05 |
| ATPase H+ transporting accessory protein 2 | ATP6AP2 | 1,26E-05 | 2,047 |
| bridging integrator 1 | BIN1 | 3,65E-05 | 2,047 |
| AU RNA binding methylglutaconyl-CoA hydratase | AUH | 1,42E-05 | 2,045 |
| C-C motif chemokine ligand 17 | CCL17 | 3,98E-04 | 2,044 |
| nucleotide binding protein 1 | NUBP1 | 2,27E-06 | 2,044 |
| CKLF like MARVEL transmembrane domain containing 3 | CMTM3 | 1,53E-04 | 2,042 |
| 1-acylglycerol-3-phosphate O-acyltransferase 4 | AGPAT4 | 1,96E-06 | 2,034 |
| makorin ring finger protein 1 | MKRN1 | 9,56E-06 | 2,033 |
| sushi repeat containing protein, X-linked | SRPX | 1,12E-04 | 2,032 |
| apolipoprotein B mRNA editing enzyme catalytic subunit 3B | APOBEC3B | 2,92E-06 | 2,03 |
| uncharacterized LOC728392 | LOC728392 | 5,69E-06 | 2,027 |
| chemokine like factor | CKLF | 1,76E-06 | 2,025 |
| filamin A | FLNA | 6,95E-06 | 2,025 |
| NUAK family kinase 2 | NUAK2 | 6,06E-04 | 2,024 |
| alkaline ceramidase 3 | ACER3 | 1,36E-05 | 2,021 |
| ARP3 actin related protein 3 homolog | ACTR3 | 2,84E-05 | 2,021 |
| T-cell receptor alpha locus | TRA | 4,35E-06 | 2,021 |
| growth differentiation factor 15 | GDF15 | 1,57E-02 | 2,02 |
| ladinin 1 | LAD1 | 2,57E-04 | 2,016 |
| EYA transcriptional coactivator and phosphatase 2 | EYA2 | 1,68E-04 | 2,015 |
| predicted gene 4907 | Gm4907 | 1,48E-03 | 2,014 |
| synuclein alpha | SNCA | 3,17E-03 | 2,014 |
| sorcin | SRI | 1,24E-07 | 2,014 |
| podoplanin | PDPN | 5,69E-04 | 2,013 |
| telomerase associated protein 1 | TEP1 | 8,89E-07 | 2,012 |
| palmitoyl-protein thioesterase 1 | PPT1 | 5,98E-07 | 2,01 |
| Pim-1 proto-oncogene, serine/threonine kinase | PIM1 | 9,95E-04 | 2,008 |
| CDC42 small effector 1 | CDC42SE1 | 1,44E-06 | 2,006 |
| tensin 3 | TNS3 | 1,26E-07 | 2,006 |
| solute carrier family 43 member 2 | SLC43A2 | 1,35E-03 | 2,003 |
| solute carrier family 4 member 1 (Diego blood group) | SLC4A1 | 1,04E-03 | 2,003 |
| collagen type XIV alpha 1 chain | COL14A1 | 3,34E-03 | 2,001 |
| phosphatidylinositol-5-phosphate 4-kinase type 2 alpha | PIP4K2A | 1,21E-05 | 2 |
| carboxymethylenebutenolidase homolog | CMBL | 2,36E-05 | -2,001 |
| PR/SET domain 16 | PRDM16 | 3,78E-04 | -2,001 |
| acetyl-CoA acyltransferase 2 | ACAA2 | 6,71E-05 | -2,002 |
| Fas activated serine/threonine kinase | FASTK | 2,72E-05 | -2,002 |
| Riken cDNA A430046D13 gene | A430046D13Rik | 1,83E-04 | -2,003 |
| actin, alpha 1, skeletal muscle | ACTA1 | 2,63E-02 | -2,005 |
| DNA methyltransferase 3 alpha | DNMT3A | 1,45E-02 | -2,005 |
| solute carrier family 22 member 5 | SLC22A5 | 1,86E-04 | -2,005 |
| valyl-tRNA synthetase 2, mitochondrial | VARS2 | 2,64E-07 | -2,006 |
| transmembrane protein 201 | TMEM201 | 2,40E-05 | -2,007 |
| zinc finger protein 606 | ZNF606 | 1,26E-03 | -2,008 |
| homer scaffolding protein 2 | HOMER2 | 1,29E-05 | -2,009 |
| isovaleryl-CoA dehydrogenase | IVD | 4,64E-06 | -2,009 |
| MACRO domain containing 2 | MACROD2 | 2,40E-05 | -2,01 |
| zinc finger and SCAN domain containing 22 | ZSCAN22 | 1,17E-03 | -2,012 |
| alanyl-tRNA synthetase 2, mitochondrial | AARS2 | 1,57E-07 | -2,013 |
| signal recognition particle 54 | SRP54 | 1,34E-06 | -2,014 |
| glutamic--pyruvic transaminase 2 | GPT2 | 1,34E-05 | -2,015 |
| bromodomain and WD repeat domain containing 1 | BRWD1 | 1,95E-05 | -2,016 |
| GSG1 like | GSG1L | 1,47E-06 | -2,018 |
| tetratricopeptide repeat domain 21B | TTC21B | 3,56E-04 | -2,019 |
| tescalcin | TESC | 3,26E-05 | -2,02 |
| acyl-CoA synthetase long-chain family member 1 | ACSL1 | 3,57E-07 | -2,021 |
| myosin light chain 1 | MYL1 | 3,73E-04 | -2,021 |
| sorbitol dehydrogenase | SORD | 1,49E-06 | -2,021 |
| G protein-coupled receptor associated sorting protein 2 | Gprasp2 | 2,96E-06 | -2,022 |
| heat shock factor binding protein 1 like 1 | HSBP1L1 | 2,10E-05 | -2,023 |
| fin bud initiation factor homolog (zebrafish) | FIBIN | 2,80E-04 | -2,027 |
| NADH:ubiquinone oxidoreductase subunit A1 | NDUFA1 | 4,83E-06 | -2,03 |
| platelet factor 4 | PF4 | 1,45E-03 | -2,032 |
| receptor accessory protein 6 | REEP6 | 3,35E-06 | -2,034 |
| lectin, galactose binding, soluble 6 | Lgals6 | 1,06E-03 | -2,035 |
| solute carrier family 39 member 3 | SLC39A3 | 1,08E-04 | -2,04 |
| mitochondrial trans-2-enoyl-CoA reductase | MECR | 1,78E-06 | -2,041 |
| coenzyme Q9 | COQ9 | 1,33E-05 | -2,042 |
| HEAT repeat containing 5B | HEATR5B | 2,17E-03 | -2,042 |
| insulin receptor substrate 1 | IRS1 | 1,45E-05 | -2,042 |
| myosin light chain kinase family member 4 | MYLK4 | 7,72E-05 | -2,043 |
| secernin 2 | SCRN2 | 3,90E-06 | -2,044 |
| predicted gene 428 | Gm428 (includes others) | 1,67E-04 | -2,045 |
| receptor accessory protein 5 | REEP5 | 4,00E-06 | -2,045 |
| ring finger protein 207 | RNF207 | 9,94E-04 | -2,046 |
| tachykinin precursor 1 | TAC1 | 1,83E-03 | -2,046 |
| ubiquitin conjugating enzyme E2 N | UBE2N | 1,04E-04 | -2,047 |
| hydroxyacyl-CoA dehydrogenase/3-ketoacyl-CoA thiolase/enoyl-CoA hydratase (trifunctional protein), beta subunit | HADHB | 1,75E-05 | -2,05 |
| solute carrier family 24 member 3 | SLC24A3 | 2,05E-04 | -2,05 |
| transforming acidic coiled-coil containing protein 2 | TACC2 | 9,42E-03 | -2,051 |
| NHL repeat containing E3 ubiquitin protein ligase 1 | NHLRC1 | 1,35E-05 | -2,052 |
| neuritin 1 | NRN1 | 3,02E-04 | -2,052 |
| peroxisomal membrane protein 4 | PXMP4 | 1,06E-05 | -2,055 |
| regulator of G-protein signaling 4 | RGS4 | 4,87E-04 | -2,056 |
| actin binding Rho activating protein | ABRA | 1,51E-04 | -2,057 |
| alcohol dehydrogenase, iron containing 1 | ADHFE1 | 3,85E-06 | -2,06 |
| CCHC-type zinc finger nucleic acid binding protein | CNBP | 8,89E-05 | -2,06 |
| threonine aldolase 1 | Tha1 | 1,08E-05 | -2,06 |
| Wnt family member 9A | WNT9A | 1,08E-06 | -2,061 |
| coiled-coil-helix-coiled-coil-helix domain containing 7 | CHCHD7 | 4,17E-05 | -2,062 |
| ring finger protein 150 | RNF150 | 2,31E-03 | -2,062 |
| harakiri, BCL2 interacting protein (contains only BH3 domain) | Hrk | 1,38E-05 | -2,063 |
| chromosome 8 open reading frame 4 | C8orf4 | 7,80E-05 | -2,065 |
| kelch like family member 30 | KLHL30 | 1,13E-04 | -2,069 |
| solute carrier family 5 member 6 | SLC5A6 | 1,49E-04 | -2,069 |
| cytokine like 1 | CYTL1 | 5,48E-05 | -2,071 |
| zinc finger and BTB domain containing 20 | ZBTB20 | 2,81E-05 | -2,072 |
| 4-aminobutyrate aminotransferase | ABAT | 2,15E-05 | -2,074 |
| crystallin beta B1 | CRYBB1 | 2,65E-06 | -2,077 |
| protease, serine 36 | PRSS36 | 5,11E-06 | -2,078 |
| 3-hydroxyisobutyryl-CoA hydrolase | HIBCH | 6,95E-06 | -2,08 |
| abhydrolase domain containing 14A | ABHD14A | 6,22E-05 | -2,081 |
| glucokinase | GCK | 5,52E-03 | -2,082 |
| myosin XVIIIB | MYO18B | 6,86E-07 | -2,085 |
| Rho related BTB domain containing 3 | RHOBTB3 | 2,97E-05 | -2,086 |
| GDNF family receptor alpha 3 | GFRA3 | 5,49E-05 | -2,087 |
| methylmalonyl-CoA epimerase | MCEE | 9,35E-06 | -2,087 |
| aldehyde oxidase 1 | AOX1 | 4,37E-04 | -2,088 |
| glycerol-3-phosphate acyltransferase, mitochondrial | GPAM | 5,69E-05 | -2,088 |
| Ly6/neurotoxin 1 | LYNX1 | 7,58E-05 | -2,088 |
| ATP binding cassette subfamily B member 6 (Langereis blood group) | ABCB6 | 1,24E-05 | -2,09 |
| chromosome 7 open reading frame 49 | C7orf49 | 9,88E-04 | -2,091 |
| cDNA sequence BC030870 | BC030870 | 8,90E-04 | -2,092 |
| fumarylacetoacetate hydrolase domain containing 2B | FAHD2B | 3,99E-06 | -2,092 |
| ras homolog family member T2 | RHOT2 | 5,06E-06 | -2,092 |
| chromosome 15 open reading frame 52 | C15orf52 | 2,85E-04 | -2,094 |
| PDZ domain containing ring finger 4 | PDZRN4 | 4,33E-05 | -2,094 |
| RIC8 guanine nucleotide exchange factor B | RIC8B | 2,57E-05 | -2,097 |
| small VCP interacting protein | SVIP | 2,43E-06 | -2,098 |
| potassium voltage-gated channel subfamily D member 3 | KCND3 | 5,70E-07 | -2,099 |
| disco interacting protein 2 homolog C | DIP2C | 3,02E-06 | -2,102 |
| prolactin receptor | PRLR | 8,06E-05 | -2,103 |
| dihydrouridine synthase 4 like | DUS4L | 3,07E-03 | -2,104 |
| cytochrome b5 domain containing 2 | CYB5D2 | 8,19E-05 | -2,105 |
| kyphoscoliosis peptidase | KY | 5,67E-04 | -2,106 |
| coiled-coil domain containing 28A | CCDC28A | 3,52E-05 | -2,107 |
| obscurin, cytoskeletal calmodulin and titin-interacting RhoGEF | OBSCN | 3,54E-05 | -2,107 |
| transmembrane protein 25 | TMEM25 | 9,36E-04 | -2,111 |
| dihydroorotate dehydrogenase (quinone) | DHODH | 5,69E-07 | -2,112 |
| biphenyl hydrolase like | BPHL | 9,70E-06 | -2,116 |
| oxysterol binding protein 2 | OSBP2 | 1,61E-05 | -2,117 |
| erythropoietin receptor | EPOR | 8,37E-07 | -2,118 |
| fibrinogen like 1 | FGL1 | 1,01E-05 | -2,119 |
| RAB3A, member RAS oncogene family | RAB3A | 3,39E-05 | -2,119 |
| cytoplasmic linker associated protein 1 | CLASP1 | 5,06E-05 | -2,121 |
| MAS-related GPR, member H | Mrgprh | 1,24E-05 | -2,122 |
| DNA polymerase gamma, catalytic subunit | POLG | 1,42E-04 | -2,122 |
| transmembrane protein 143 | TMEM143 | 1,80E-04 | -2,125 |
| ral guanine nucleotide dissociation stimulator like 3 | RGL3 | 2,96E-04 | -2,126 |
| nitrilase 1 | NIT1 | 6,10E-03 | -2,127 |
| exocyst complex component 8 | EXOC8 | 4,15E-05 | -2,13 |
| G protein-coupled receptor associated sorting protein 1 | GPRASP1 | 2,23E-06 | -2,13 |
| synaptopodin | SYNPO | 3,28E-05 | -2,134 |
| heparan sulfate-glucosamine 3-sulfotransferase 5 | HS3ST5 | 5,58E-04 | -2,136 |
| sulfite oxidase | SUOX | 1,50E-06 | -2,138 |
| Tu translation elongation factor, mitochondrial | TUFM | 1,33E-05 | -2,14 |
| hydroxyacyl-CoA dehydrogenase | HADH | 2,46E-05 | -2,141 |
| phosphodiesterase 4A | PDE4A | 1,57E-05 | -2,141 |
| methylcrotonoyl-CoA carboxylase 1 | MCCC1 | 2,81E-07 | -2,142 |
| tetratricopeptide repeat domain 30B | TTC30B | 1,63E-06 | -2,143 |
| lactation elevated 1 | LACE1 | 9,13E-06 | -2,146 |
| inturned planar cell polarity protein | INTU | 1,47E-04 | -2,148 |
| pyruvate dehyrogenase phosphatase catalytic subunit 1 | PDP1 | 1,92E-06 | -2,149 |
| chromosome 10 open reading frame 107 | C10orf107 | 6,31E-05 | -2,152 |
| ankyrin repeat and SOCS box containing 5 | ASB5 | 6,96E-05 | -2,155 |
| cut like homeobox 2 | CUX2 | 3,83E-06 | -2,156 |
| alpha kinase 2 | ALPK2 | 8,70E-06 | -2,159 |
| transmembrane protein 41B | TMEM41B | 4,16E-07 | -2,159 |
| RAD52 motif containing 1 | RDM1 | 1,65E-04 | -2,16 |
| Kruppel like factor 12 | KLF12 | 3,54E-06 | -2,161 |
| sushi, nidogen and EGF like domains 1 | SNED1 | 1,10E-06 | -2,161 |
| hydroxysteroid dehydrogenase like 2 | Hsdl2 | 1,11E-06 | -2,162 |
| transcription factor B1, mitochondrial | TFB1M | 1,63E-05 | -2,164 |
| RIKEN cDNA E330011O21 gene | E330011O21Rik | 1,93E-06 | -2,168 |
| MAGI family member, X-linked | MAGIX | 2,18E-04 | -2,168 |
| WNT inhibitory factor 1 | WIF1 | 1,93E-04 | -2,168 |
| aquaporin 8 | AQP8 | 4,65E-03 | -2,171 |
| predicted gene 826 | Gm826 | 3,79E-07 | -2,174 |
| ST6 N-acetylgalactosaminide alpha-2,6-sialyltransferase 2 | ST6GALNAC2 | 1,69E-06 | -2,174 |
| acyl-CoA dehydrogenase family member 11 | ACAD11 | 9,63E-07 | -2,175 |
| ribosomal protein L3 like | RPL3L | 2,28E-04 | -2,176 |
| electron transfer flavoprotein dehydrogenase | ETFDH | 7,36E-06 | -2,179 |
| antisense Igf2r RNA | Airn | 9,03E-05 | -2,183 |
| centromere protein V | CENPV | 9,92E-07 | -2,184 |
| heat shock protein family D (Hsp60) member 1 | HSPD1 | 1,36E-06 | -2,186 |
| latent transforming growth factor beta binding protein 4 | LTBP4 | 2,43E-05 | -2,186 |
| protein kinase (cAMP-dependent, catalytic) inhibitor alpha | PKIA | 2,55E-06 | -2,188 |
| aldehyde dehydrogenase 4 family member A1 | ALDH4A1 | 2,23E-05 | -2,193 |
| H19, imprinted maternally expressed transcript (non-protein coding) | H19 | 7,72E-05 | -2,196 |
| L-2-hydroxyglutarate dehydrogenase | L2HGDH | 4,76E-07 | -2,196 |
| enoyl-CoA hydratase 1 | ECH1 | 6,31E-05 | -2,198 |
| aldehyde dehydrogenase 6 family member A1 | ALDH6A1 | 1,19E-06 | -2,199 |
| zinc finger protein 536 | ZNF536 | 1,30E-05 | -2,199 |
| amyloid beta precursor protein binding family B member 1 | APBB1 | 3,72E-05 | -2,2 |
| creatine kinase, M-type | CKM | 5,08E-04 | -2,201 |
| pleckstrin homology, MyTH4 and FERM domain containing H1 | PLEKHH1 | 3,42E-02 | -2,201 |
| angiopoietin like 3 | ANGPTL3 | 2,30E-06 | -2,204 |
| caprin family member 2 | CAPRIN2 | 4,47E-08 | -2,206 |
| solute carrier family 26 member 6 | SLC26A6 | 3,66E-07 | -2,206 |
| heart and neural crest derivatives expressed 2 | HAND2 | 2,51E-04 | -2,207 |
| dihydrodiol dehydrogenase | DHDH | 3,65E-05 | -2,213 |
| solute carrier family 25 member 33 | SLC25A33 | 9,54E-05 | -2,214 |
| ChaC glutathione specific gamma-glutamylcyclotransferase 1 | CHAC1 | 2,26E-02 | -2,216 |
| NCBP2 antisense RNA 2 (head to head) | NCBP2-AS2 | 8,18E-05 | -2,219 |
| forkhead box O6 | FOXO6 | 3,33E-04 | -2,221 |
| Y-box binding protein 2 | YBX2 | 1,23E-05 | -2,221 |
| potassium voltage-gated channel subfamily A member 7 | KCNA7 | 9,59E-05 | -2,222 |
| MAM domain containing glycosylphosphatidylinositol anchor 1 | MDGA1 | 3,07E-04 | -2,222 |
| cyclin H | CCNH | 1,41E-06 | -2,225 |
| proteolipid protein 1 | PLP1 | 5,48E-06 | -2,225 |
| dopa decarboxylase | DDC | 1,59E-06 | -2,229 |
| cholinergic receptor nicotinic alpha 2 subunit | CHRNA2 | 1,08E-03 | -2,23 |
| enoyl-CoA hydratase domain containing 3 | ECHDC3 | 1,14E-05 | -2,232 |
| leucine rich repeat containing 39 | LRRC39 | 7,54E-06 | -2,234 |
| ATP binding cassette subfamily C member 9 | ABCC9 | 5,05E-05 | -2,236 |
| SH3-binding domain glutamic acid-rich protein | Sh3bgr | 4,71E-05 | -2,236 |
| cartilage oligomeric matrix protein | COMP | 1,20E-04 | -2,239 |
| serine/arginine repetitive matrix 4 | SRRM4 | 3,31E-03 | -2,242 |
| fructosamine 3 kinase | FN3K | 3,23E-05 | -2,244 |
| amylo-alpha-1, 6-glucosidase, 4-alpha-glucanotransferase | AGL | 1,40E-06 | -2,246 |
| transmembrane protein 132A | TMEM132A | 2,96E-05 | -2,249 |
| galectin 4 | LGALS4 | 8,32E-04 | -2,25 |
| synemin | SYNM | 1,32E-03 | -2,254 |
| RIKEN cDNA 1110019D14 gene | 1110019D14Rik | 1,85E-04 | -2,256 |
| transmembrane protein 65 | TMEM65 | 5,76E-05 | -2,259 |
| very low density lipoprotein receptor | VLDLR | 1,66E-07 | -2,259 |
| pro-platelet basic protein | Ppbp | 4,81E-03 | -2,263 |
| kelch like family member 31 | KLHL31 | 1,39E-05 | -2,264 |
| cyclin dependent kinase 5 | CDK5 | 2,15E-05 | -2,266 |
| fibronectin type III domain containing 5 | FNDC5 | 2,15E-05 | -2,27 |
| polycystin 2 like 2, transient receptor potential cation channel | PKD2L2 | 4,00E-06 | -2,272 |
| methylmalonyl-CoA mutase | MUT | 9,83E-06 | -2,273 |
| fructose-bisphosphatase 2 | FBP2 | 7,24E-05 | -2,274 |
| BCL2 like 13 | BCL2L13 | 4,35E-06 | -2,275 |
| DDB1 and CUL4 associated factor 4 | DCAF4 | 5,44E-07 | -2,275 |
| RIKEN cDNA 1810014B01 gene | 1810014B01Rik | 9,77E-06 | -2,277 |
| repulsive guidance molecule family member a | RGMA | 1,34E-05 | -2,278 |
| dihydrolipoamide S-acetyltransferase | DLAT | 8,40E-06 | -2,28 |
| RIKEN cDNA A630039O03 gene | A630039O03Rik | 5,63E-06 | -2,285 |
| major facilitator superfamily domain containing 4B3 | Mfsd4b3 | 4,68E-04 | -2,287 |
| ependymin related 1 | EPDR1 | 3,78E-07 | -2,288 |
| DNA polymerase N | Poln | 3,87E-03 | -2,288 |
| solute carrier family 38 member 3 | SLC38A3 | 1,18E-05 | -2,289 |
| TSC22 domain family member 1 | TSC22D1 | 7,56E-06 | -2,291 |
| hydroxyacyl-CoA dehydrogenase/3-ketoacyl-CoA thiolase/enoyl-CoA hydratase (trifunctional protein), alpha subunit | HADHA | 2,54E-05 | -2,292 |
| RIKEN cDNA 9330102E08 gene | 9330102E08Rik | 6,94E-07 | -2,295 |
| beta-1,3-galactosyltransferase 2 | B3GALT2 | 3,34E-03 | -2,297 |
| growth factor receptor bound protein 14 | GRB14 | 5,51E-06 | -2,299 |
| chromosome 19 open reading frame 68 | C19orf68 | 5,69E-08 | -2,31 |
| defective in cullin neddylation 1 domain containing 2 | DCUN1D2 | 2,99E-04 | -2,31 |
| RIKEN cDNA A930018M24 gene | A930018M24Rik | 9,08E-05 | -2,311 |
| acyl-CoA thioesterase 2 | ACOT2 | 4,51E-06 | -2,311 |
| oxoglutarate dehydrogenase-like | OGDHL | 5,49E-04 | -2,311 |
| dihydrolipoamide branched chain transacylase E2 | DBT | 5,66E-06 | -2,321 |
| calreticulin 3 | CALR3 | 1,51E-04 | -2,323 |
| Ly6/Plaur domain containing 2 | Lypd2 | 5,98E-06 | -2,328 |
| caprin family member 2 | CAPRIN2 | 2,12E-07 | -2,329 |
| cytochrome b5 reductase like | CYB5RL | 2,24E-05 | -2,329 |
| meiosis/spermiogenesis associated 1 | MEIG1 | 1,05E-04 | -2,329 |
| protocadherin 12 | PCDH12 | 3,13E-03 | -2,333 |
| expressed sequence AI426330 | AI426330 | 7,55E-07 | -2,335 |
| leucine rich repeats and transmembrane domains 1 | LRTM1 | 2,16E-04 | -2,337 |
| lactate dehydrogenase D | LDHD | 1,47E-06 | -2,342 |
| malonyl-CoA decarboxylase | MLYCD | 2,37E-06 | -2,344 |
| sperm flagellar 1 | SPEF1 | 2,58E-04 | -2,344 |
| 3-oxoacid CoA-transferase 1 | OXCT1 | 6,56E-06 | -2,348 |
| notch 1 | NOTCH1 | 6,47E-05 | -2,353 |
| FK506 binding protein 4 | FKBP4 | 1,12E-05 | -2,354 |
| intraflagellar transport 81 | IFT81 | 2,79E-06 | -2,359 |
| peroxisomal biogenesis factor 6 | PEX6 | 1,33E-05 | -2,36 |
| amylase, alpha 2A (pancreatic) | AMY2A | 3,70E-04 | -2,361 |
| retinol saturase | RETSAT | 5,78E-06 | -2,367 |
| fibroblast growth factor 9 | FGF9 | 2,32E-03 | -2,371 |
| protein O-linked mannose N-acetylglucosaminyltransferase 2 (beta 1,4-) | POMGNT2 | 2,33E-06 | -2,371 |
| kelch like family member 38 | KLHL38 | 1,33E-04 | -2,372 |
| chromosome 14 open reading frame 37 | C14orf37 | 1,25E-06 | -2,373 |
| activin A receptor type 2B | ACVR2B | 7,61E-05 | -2,375 |
| syntaxin binding protein 6 | STXBP6 | 4,93E-07 | -2,376 |
| zinc finger protein pseudogene | BC025920 | 1,28E-05 | -2,379 |
| progestin and adipoQ receptor family member 9 | PAQR9 | 8,54E-05 | -2,387 |
| transmembrane channel like 7 | TMC7 | 2,69E-05 | -2,394 |
| family with sequence similarity 179 member A | FAM179A | 7,46E-05 | -2,395 |
| required for meiotic nuclear division 1 homolog | RMND1 | 1,57E-05 | -2,4 |
| coproporphyrinogen oxidase | CPOX | 5,74E-05 | -2,403 |
| NME/NM23 nucleoside diphosphate kinase 4 | NME4 | 7,83E-07 | -2,41 |
| TBC1 domain family member 4 | TBC1D4 | 6,87E-05 | -2,412 |
| DnaJ heat shock protein family (Hsp40) member C28 | DNAJC28 | 7,67E-05 | -2,418 |
| hes related family bHLH transcription factor with YRPW motif-like | HEYL | 1,22E-03 | -2,419 |
| hydroxyprostaglandin dehydrogenase 15-(NAD) | HPGD | 1,28E-05 | -2,424 |
| pleckstrin homology domain containing B1 | PLEKHB1 | 1,67E-06 | -2,424 |
| histone cluster 1 H2B family member j | HIST1H2BJ | 1,76E-06 | -2,431 |
| troponin T1, slow skeletal type | TNNT1 | 1,15E-07 | -2,438 |
| triadin | TRDN | 8,11E-08 | -2,439 |
| Josephin domain containing 2 | JOSD2 | 2,58E-04 | -2,443 |
| fumarylacetoacetate hydrolase | FAH | 1,47E-03 | -2,446 |
| chondroadherin like | CHADL | 5,73E-07 | -2,453 |
| enoyl-CoA hydratase and 3-hydroxyacyl CoA dehydrogenase | EHHADH | 1,38E-03 | -2,453 |
| C1q and tumor necrosis factor related protein 4 | C1QTNF4 | 1,22E-06 | -2,457 |
| RAB6B, member RAS oncogene family | RAB6B | 2,23E-05 | -2,459 |
| Rho GTPase activating protein 20 | ARHGAP20 | 4,23E-04 | -2,472 |
| solute carrier family 7 member 4 | SLC7A4 | 2,85E-05 | -2,473 |
| pescadillo ribosomal biogenesis factor 1 | PES1 | 3,47E-06 | -2,474 |
| chromosome 4 open reading frame 33 | C4orf33 | 2,89E-06 | -2,48 |
| peroxisomal biogenesis factor 11 alpha | PEX11A | 3,24E-06 | -2,486 |
| predicted gene 14290 | Gm14290 | 2,53E-06 | -2,487 |
| A-kinase anchoring protein 5 | AKAP5 | 2,35E-03 | -2,496 |
| HRAS like suppressor | HRASLS | 3,95E-05 | -2,498 |
| fibronectin type III domain containing 4 | FNDC4 | 4,46E-06 | -2,502 |
| transient receptor potential cation channel subfamily M member 1 | TRPM1 | 9,11E-05 | -2,503 |
| glutathione S-transferase mu 2 | GSTM2 | 6,59E-06 | -2,504 |
| crystallin beta A4 | CRYBA4 | 4,81E-06 | -2,505 |
| uroporphyrinogen III synthase | UROS | 7,52E-07 | -2,507 |
| synaptopodin 2 | SYNPO2 | 4,74E-08 | -2,509 |
| RIKEN cDNA 2700097O09 gene | 2700097O09Rik | 2,31E-06 | -2,511 |
| troponin T1, slow skeletal type | TNNT1 | 8,02E-08 | -2,517 |
| glutathione S-transferase kappa 1 | GSTK1 | 1,51E-06 | -2,518 |
| glycerol-3-phosphate acyltransferase 3 | GPAT3 | 4,45E-05 | -2,519 |
| histidine rich calcium binding protein | Hrc | 1,06E-05 | -2,524 |
| feline leukemia virus subgroup C cellular receptor family member 2 | FLVCR2 | 6,88E-04 | -2,527 |
| nephrocan | Nepn | 9,83E-04 | -2,528 |
| glutaryl-CoA dehydrogenase | GCDH | 3,52E-08 | -2,529 |
| catenin alpha like 1 | CTNNAL1 | 6,66E-04 | -2,541 |
| Purkinje cell protein 4 | PCP4 | 7,24E-08 | -2,545 |
| peroxisomal membrane protein 2 | PXMP2 | 1,63E-05 | -2,549 |
| MLX interacting protein like | MLXIPL | 2,37E-07 | -2,551 |
| cerebral dopamine neurotrophic factor | CDNF | 6,25E-05 | -2,555 |
| phosphorylase kinase catalytic subunit gamma 1 | PHKG1 | 1,07E-04 | -2,562 |
| predicted gene 1673 | Gm1673 | 7,54E-06 | -2,568 |
| dopachrome tautomerase | DCT | 1,71E-03 | -2,575 |
| solute carrier family 36 member 2 | SLC36A2 | 3,87E-08 | -2,575 |
| DNA polymerase theta | POLQ | 2,46E-05 | -2,584 |
| cDNA sequence BC062258 | BC062258 | 2,53E-05 | -2,586 |
| chromosome 14 open reading frame 159 | C14orf159 | 3,52E-07 | -2,587 |
| transmembrane protein 82 | TMEM82 | 1,46E-04 | -2,591 |
| cysteinyl-tRNA synthetase 2, mitochondrial (putative) | CARS2 | 1,12E-07 | -2,593 |
| EPH receptor B1 | EPHB1 | 3,69E-07 | -2,594 |
| adipogenin | Adig | 4,65E-08 | -2,597 |
| CD163 molecule | CD163 | 2,98E-07 | -2,599 |
| extracellular matrix protein 2 | ECM2 | 9,69E-05 | -2,61 |
| cytochrome c oxidase subunit 6B2 | COX6B2 | 4,35E-06 | -2,612 |
| unc-45 myosin chaperone B | UNC45B | 4,76E-07 | -2,612 |
| crystallin beta A4 | CRYBA4 | 2,43E-07 | -2,621 |
| granzyme M | GZMM | 8,67E-07 | -2,638 |
| kelch like family member 34 | KLHL34 | 1,26E-07 | -2,64 |
| indolethylamine N-methyltransferase | INMT | 1,29E-03 | -2,65 |
| immunoglobulin superfamily DCC subclass member 4 | IGDCC4 | 1,45E-05 | -2,657 |
| methylcrotonoyl-CoA carboxylase 2 | MCCC2 | 2,22E-08 | -2,663 |
| EPH receptor A4 | EPHA4 | 2,61E-04 | -2,68 |
| solute carrier family 25 member 26 | SLC25A26 | 1,43E-06 | -2,684 |
| endonuclease/exonuclease/phosphatase family domain containing 1 | EEPD1 | 8,60E-08 | -2,696 |
| polo like kinase 5 | PLK5 | 4,56E-07 | -2,701 |
| transmembrane protein 177 | TMEM177 | 1,25E-08 | -2,706 |
| methylmalonic aciduria (cobalamin deficiency) cblA type | MMAA | 5,49E-07 | -2,711 |
| kelch domain containing 1 | KLHDC1 | 7,55E-06 | -2,712 |
| acyl-CoA thioesterase 5 | Acot5 | 4,40E-04 | -2,718 |
| myotilin | MYOT | 5,95E-05 | -2,724 |
| RNA binding motif protein 33 | Rbm33 | 9,00E-05 | -2,748 |
| McKusick-Kaufman syndrome | MKKS | 3,03E-04 | -2,755 |
| secreted frizzled related protein 5 | SFRP5 | 1,32E-05 | -2,769 |
| protein phosphatase 1 regulatory subunit 9A | PPP1R9A | 1,04E-06 | -2,8 |
| potassium voltage-gated channel subfamily A member 1 | KCNA1 | 6,34E-06 | -2,807 |
| DDB1 and CUL4 associated factor 12-like 1 | Dcaf12l1 | 1,43E-07 | -2,813 |
| cyclin-dependent kinase inhibitor 1C (P57) | Cdkn1c | 8,82E-08 | -2,814 |
| microtubule associated protein tau | MAPT | 1,79E-06 | -2,821 |
| RAS like family 10 member A | RASL10A | 4,66E-06 | -2,823 |
| dephospho-CoA kinase domain containing | DCAKD | 3,18E-06 | -2,839 |
| bolA family member 1 | BOLA1 | 1,16E-06 | -2,843 |
| solute carrier family 25 member 42 | SLC25A42 | 2,94E-06 | -2,852 |
| zinc finger protein 23 | ZNF23 | 8,84E-07 | -2,858 |
| talin 2 | TLN2 | 1,67E-08 | -2,863 |
| chromosome 4 open reading frame 19 | C4orf19 | 1,59E-06 | -2,88 |
| syntaxin binding protein 4 | STXBP4 | 6,51E-05 | -2,886 |
| glycine C-acetyltransferase | GCAT | 4,88E-07 | -2,887 |
| family with sequence similarity 19 member A5, C-C motif chemokine like | FAM19A5 | 6,57E-09 | -2,904 |
| receptor accessory protein 1 | REEP1 | 2,53E-07 | -2,92 |
| family with sequence similarity 131 member A | FAM131A | 7,58E-07 | -2,927 |
| brain derived neurotrophic factor | BDNF | 1,24E-06 | -2,98 |
| methionine sulfoxide reductase B2 | MSRB2 | 2,26E-06 | -2,981 |
| carnitine palmitoyltransferase 2 | CPT2 | 5,90E-09 | -2,983 |
| HR, lysine demethylase and nuclear receptor corepressor | HR | 6,16E-07 | -3,005 |
| butyrylcholinesterase | BCHE | 1,87E-06 | -3,02 |
| proenkephalin | PENK | 5,17E-07 | -3,042 |
| armadillo repeat containing 2 | ARMC2 | 2,01E-06 | -3,082 |
| leucine rich repeat and Ig domain containing 3 | LINGO3 | 2,02E-06 | -3,087 |
| integrin subunit beta 6 | ITGB6 | 8,11E-03 | -3,221 |
| acyl-CoA thioesterase 1 | Acot1 | 1,96E-04 | -3,222 |
| polymeric immunoglobulin receptor | PIGR | 5,86E-04 | -3,223 |
| distal-less homeobox 3 | DLX3 | 3,46E-06 | -3,239 |
| pyruvate dehyrogenase phosphatase catalytic subunit 2 | PDP2 | 4,86E-08 | -3,244 |
| D-aspartate oxidase | DDO | 4,29E-09 | -3,251 |
| neurotrophin 3 | NTF3 | 7,69E-08 | -3,286 |
| dopamine beta-hydroxylase | DBH | 8,85E-06 | -3,343 |
| amphiphysin | AMPH | 1,63E-08 | -3,353 |
| predicted gene 4956 | Gm4956 | 3,78E-07 | -3,354 |
| pyruvate dehydrogenase kinase 4 | PDK4 | 1,18E-02 | -3,419 |
| sodium voltage-gated channel beta subunit 4 | SCN4B | 1,15E-04 | -3,427 |
| C-type lectin domain family 18 member B | CLEC18B | 2,69E-05 | -3,456 |
| DCC netrin 1 receptor | DCC | 1,19E-03 | -3,533 |
| arachidonate 5-lipoxygenase | ALOX5 | 2,25E-06 | -3,555 |
| angiopoietin like 4 | ANGPTL4 | 2,91E-03 | -3,567 |
| brain expressed X-linked 1 | Bex1 | 1,06E-05 | -3,58 |
| immunoglobulin superfamily member 1 | IGSF1 | 3,37E-06 | -3,6 |
| 6-phosphofructo-2-kinase/fructose-2,6-biphosphatase 1 | PFKFB1 | 4,62E-06 | -3,676 |
| Fas activated serine/threonine kinase | FASTK | 6,29E-05 | -3,689 |
| purinergic receptor P2Y1 | P2RY1 | 6,06E-06 | -3,702 |
| solute carrier family 22 member 1 | SLC22A1 | 2,31E-06 | -3,741 |
| GRB2 associated binding protein 3 | GAB3 | 3,10E-04 | -3,747 |
| solute carrier family 40 member 1 | SLC40A1 | 1,36E-06 | -3,835 |
| myosin binding protein C, fast type | MYBPC2 | 3,11E-09 | -3,983 |
| potassium voltage-gated channel modifier subfamily V member 2 | KCNV2 | 8,53E-09 | -4,006 |
| paraneoplastic Ma antigen 2 | PNMA2 | 5,77E-05 | -4,028 |
| GTP cyclohydrolase I feedback regulator | GCHFR | 5,89E-08 | -4,115 |
| glial cells missing homolog 1 | GCM1 | 7,89E-04 | -4,145 |
| ermin | ERMN | 4,35E-04 | -4,208 |
| C-C motif chemokine ligand 24 | CCL24 | 3,89E-08 | -4,232 |
| aquaporin 1 (Colton blood group) | AQP1 | 1,09E-08 | -4,31 |
| Fc receptor-like S, scavenger receptor | Fcrls | 8,39E-08 | -4,354 |
| whirlin | WHRN | 5,20E-09 | -4,432 |
| ephrin B3 | EFNB3 | 4,55E-06 | -4,658 |
| reproductive homeobox 8 | Rhox8 | 2,66E-04 | -4,666 |
| carbonyl reductase 2 | Cbr2 | 9,96E-10 | -4,675 |
| 3-hydroxy-3-methylglutaryl-CoA synthase 2 | HMGCS2 | 5,11E-04 | -4,757 |
| anoctamin 10 | ANO10 | 6,51E-09 | -4,833 |
| aldolase, fructose-bisphosphate B | ALDOB | 1,12E-05 | -5,599 |
| resistin like alpha | Retnla | 5,84E-07 | -9,674 |

**Supplemental table 3:** List of all DEGs at 45 dpi.

| **Entrez Gene Name** | **Symbol** | **Expr p-value** | **Expr Fold Change** |
| --- | --- | --- | --- |
| C-C motif chemokine ligand 5 | CCL5 | 5,26E-12 | 114,559 |
| granzyme B | Gzmb | 6,78E-15 | 108,985 |
| apolipoprotein L 9b | Apol9a/Apol9b | 1,26E-14 | 106,089 |
| ubiquitin D | UBD | 8,34E-14 | 101,539 |
| granzyme A | GZMA | 3,48E-12 | 89,029 |
| apolipoprotein L 9b | Apol9a/Apol9b | 4,25E-15 | 83,966 |
| chemokine (C-C motif) ligand 8 | Ccl8 | 3,36E-13 | 77,752 |
| serine (or cysteine) peptidase inhibitor, clade A, member 3G | Serpina3g (includes others) | 1,26E-14 | 75,601 |
| C-X-C motif chemokine ligand 10 | CXCL10 | 1,95E-11 | 62,955 |
| chemokine (C-X-C motif) ligand 9 | Cxcl9 | 8,30E-12 | 57,151 |
| Z-DNA binding protein 1 | ZBP1 | 7,07E-14 | 53,026 |
| interferon gamma induced GTPase | Igtp | 7,07E-14 | 51,275 |
| complement factor B | CFB | 3,01E-12 | 50,39 |
| basic leucine zipper ATF-like transcription factor 2 | BATF2 | 7,07E-14 | 46,161 |
| membrane-spanning 4-domains, subfamily A, member 4B | Ms4a4b (includes others) | 3,92E-13 | 45,214 |
| T cell specific GTPase 1 | Tgtp1/Tgtp2 | 2,09E-10 | 39,649 |
| LCK proto-oncogene, Src family tyrosine kinase | LCK | 3,11E-12 | 36,947 |
| placenta specific 8 | PLAC8 | 5,63E-11 | 36,457 |
| interferon gamma inducible protein 47 | Ifi47 | 2,40E-12 | 33,302 |
| Fc fragment of IgG receptor IIIa | FCGR3A/FCGR3B | 7,95E-14 | 33,004 |
| T cell receptor beta, constant 2 | Trbc2 | 4,92E-12 | 32,572 |
| CD274 molecule | CD274 | 4,76E-11 | 30,744 |
| immunity-related GTPase family M member 1 | Irgm1 | 1,73E-14 | 30,313 |
| integrin subunit beta 7 | ITGB7 | 1,98E-12 | 29,531 |
| C-X-C motif chemokine receptor 6 | CXCR6 | 6,23E-13 | 29,307 |
| guanylate binding protein 2 | GBP2 | 1,29E-10 | 28,922 |
| lymphotoxin beta | LTB | 1,05E-09 | 27,956 |
| major histocompatibility complex, class II, DM beta | HLA-DMB | 1,52E-09 | 26,526 |
| SLAM family member 8 | SLAMF8 | 3,03E-12 | 26,325 |
| C-C motif chemokine ligand 4 | CCL4 | 1,95E-11 | 25,992 |
| T cell receptor beta, constant region 1 | Trbc1 | 5,45E-11 | 25,66 |
| immunity related GTPase M | IRGM | 1,76E-13 | 23,611 |
| serum amyloid A 3 | Saa3 | 4,19E-06 | 23,583 |
| macrophage receptor with collagenous structure | MARCO | 1,30E-07 | 23,328 |
| guanylate binding protein 4 | GBP4 | 1,29E-11 | 22,943 |
| chemokine (C-C motif) ligand 7 | Ccl7 | 3,68E-08 | 22,724 |
| interferon induced protein with tetratricopeptide repeats 3 | IFIT3 | 2,69E-13 | 22,321 |
| CD3d molecule | CD3D | 1,28E-13 | 22,015 |
| interferon gamma | IFNG | 2,98E-11 | 21,023 |
| BCL2 related protein A1 | BCL2A1 | 2,79E-10 | 20,983 |
| complement C2 | C2 | 6,32E-11 | 20,038 |
| interferon regulatory factor 7 | IRF7 | 7,07E-14 | 19,86 |
| major histocompatibility complex, class II, DQ alpha 1 | HLA-DQA1 | 1,91E-08 | 19,718 |
| CD52 antigen | Cd52 | 6,71E-11 | 19,645 |
| CD2 molecule | CD2 | 1,00E-11 | 19,564 |
| toll-like receptor 12 | Tlr12 | 5,74E-12 | 19,406 |
| integrin subunit alpha X | ITGAX | 1,32E-10 | 18,857 |
| galectin 3 | LGALS3 | 4,91E-11 | 18,746 |
| CD3g molecule | CD3G | 4,27E-13 | 18,701 |
| major histocompatibility complex, class II, DM alpha | HLA-DMA | 1,86E-10 | 18,618 |
| chromosome 15 open reading frame 48 | C15orf48 | 1,08E-07 | 18,616 |
| CD74 molecule | CD74 | 3,71E-08 | 18,565 |
| acid phosphatase 5, tartrate resistant | Acp5 | 7,62E-12 | 18,416 |
| interferon inducible GTPase 1 | Iigp1 | 1,31E-13 | 18,073 |
| zeta chain of T cell receptor associated protein kinase 70 | ZAP70 | 2,03E-14 | 17,744 |
| cathepsin W | CTSW | 1,46E-13 | 17,685 |
| CD6 molecule | CD6 | 3,01E-12 | 17,655 |
| proteasome subunit beta 8 | PSMB8 | 3,48E-14 | 17,652 |
| GIMAP1-GIMAP5 readthrough | GIMAP1-GIMAP5 | 7,91E-10 | 17,643 |
| chemokine (C-C motif) ligand 2 | Ccl2 | 1,61E-07 | 17,341 |
| suppressor of cytokine signaling 1 | SOCS1 | 1,36E-10 | 16,623 |
| MHC class I family member | LOC547349 | 1,32E-15 | 16,384 |
| sialophorin | SPN | 7,79E-14 | 16,316 |
| transporter 1, ATP binding cassette subfamily B member | TAP1 | 1,41E-12 | 16,255 |
| guanylate binding protein family member 6 | GBP6 | 8,00E-14 | 16,077 |
| CD72 molecule | CD72 | 2,19E-10 | 15,846 |
| C-C motif chemokine receptor 5 (gene/pseudogene) | CCR5 | 1,78E-11 | 15,628 |
| guanylate binding protein family member 6 | GBP6 | 6,66E-11 | 15,566 |
| proteasome subunit beta 9 | PSMB9 | 3,52E-12 | 15,393 |
| cathepsin S | CTSS | 1,99E-10 | 15,186 |
| major histocompatibility complex, class II, DR beta 5 | HLA-DRB5 | 1,34E-07 | 15,161 |
| interleukin 2 receptor subunit beta | IL2RB | 2,97E-12 | 14,696 |
| dual specificity phosphatase 2 | DUSP2 | 2,46E-09 | 14,654 |
| macrophage expressed 1 | MPEG1 | 2,26E-12 | 14,195 |
| guanylate binding protein family member 6 | GBP6 | 2,12E-10 | 13,963 |
| guanylate binding protein 5 | GBP5 | 8,57E-09 | 13,852 |
| allograft inflammatory factor 1 | AIF1 | 8,00E-10 | 13,438 |
| HCK proto-oncogene, Src family tyrosine kinase | HCK | 5,63E-11 | 13,41 |
| ras-related C3 botulinum toxin substrate 2 (rho family, small GTP binding protein Rac2) | RAC2 | 1,45E-12 | 13,381 |
| major histocompatibility complex, class II, DQ beta 1 | HLA-DQB1 | 5,40E-07 | 13,199 |
| histocompatibility 2, M region locus 2 | H2-M2 | 2,26E-12 | 13,076 |
| CD7 molecule | CD7 | 6,93E-09 | 13,063 |
| interleukin 12 receptor subunit beta 1 | IL12RB1 | 4,21E-12 | 13,023 |
| ISG15 ubiquitin-like modifier | ISG15 | 8,69E-11 | 12,961 |
| protein tyrosine phosphatase, receptor type C associated protein | PTPRCAP | 1,20E-12 | 12,958 |
| C-X-C motif chemokine receptor 3 | CXCR3 | 9,87E-12 | 12,794 |
| src kinase associated phosphoprotein 1 | SKAP1 | 4,97E-11 | 12,364 |
| family with sequence similarity 26 member F | FAM26F | 7,89E-12 | 12,212 |
| cholesterol 25-hydroxylase | CH25H | 1,86E-08 | 12,193 |
| killer cell lectin-like receptor, subfamily A, member 2 | Klra2 | 9,33E-13 | 12,146 |
| sodium voltage-gated channel alpha subunit 10 | SCN10A | 5,33E-12 | 12,116 |
| major histocompatibility complex, class I, A | HLA-A | 3,49E-13 | 11,998 |
| haptoglobin | HP | 9,83E-06 | 11,989 |
| hexokinase 3 | HK3 | 2,82E-11 | 11,984 |
| histocompatibility 2, Q region locus 8 | H2-Q8 | 5,12E-09 | 11,977 |
| protein tyrosine phosphatase, receptor type C | PTPRC | 6,68E-12 | 11,932 |
| granzyme K | GZMK | 1,56E-11 | 11,924 |
| CD5 molecule | CD5 | 5,46E-12 | 11,922 |
| NLR family CARD domain containing 5 | NLRC5 | 3,97E-09 | 11,902 |
| solute carrier family 2 member 6 | SLC2A6 | 5,74E-10 | 11,399 |
| coronin 1A | CORO1A | 1,99E-10 | 11,36 |
| killer cell lectin-like receptor subfamily A, member 22 | Klra22 | 1,05E-10 | 11,182 |
| guanylate-binding protein 8 | Gbp8 | 2,85E-11 | 10,98 |
| natural killer cell granule protein 7 | NKG7 | 1,00E-12 | 10,968 |
| hematopoietic cell signal transducer | HCST | 5,57E-13 | 10,765 |
| histone cluster 1 H2A family member d | HIST1H2AD | 5,85E-09 | 10,691 |
| integrin subunit beta 2 | ITGB2 | 1,02E-11 | 10,677 |
| histocompatibility 2, T region locus 9 | H2-T9 | 3,69E-13 | 10,571 |
| signal regulatory protein beta 1 | SIRPB1 | 5,59E-11 | 10,484 |
| myosin IG | MYO1G | 4,46E-11 | 10,476 |
| IL2 inducible T-cell kinase | ITK | 6,86E-12 | 10,432 |
| CD8a molecule | CD8A | 1,06E-08 | 10,28 |
| bone marrow stromal cell antigen 1 | BST1 | 3,88E-11 | 10,273 |
| T-box 21 | TBX21 | 1,66E-11 | 10,142 |
| killer cell lectin like receptor C1 | KLRC1 | 2,18E-10 | 9,999 |
| lymphocyte antigen 86 | LY86 | 1,94E-09 | 9,989 |
| histocompatibility 2, Q region locus 5 | H2-Q5 | 9,13E-13 | 9,937 |
| interferon regulatory factor 8 | IRF8 | 6,74E-12 | 9,91 |
| junction adhesion molecule like | JAML | 2,16E-06 | 9,872 |
| C-type lectin domain family 4 member E | CLEC4E | 6,09E-06 | 9,801 |
| cytochrome P450 family 4 subfamily F member 2 | CYP4F2 | 1,45E-09 | 9,743 |
| interferon induced protein with tetratricopeptide repeats 1B | IFIT1B | 1,99E-10 | 9,725 |
| serine (or cysteine) peptidase inhibitor, clade A, member 3H | Serpina3h | 5,26E-07 | 9,658 |
| major histocompatibility complex, class I, E | HLA-E | 6,66E-13 | 9,64 |
| histocompatibility 2, T region locus 10 | H2-T10 | 4,18E-12 | 9,513 |
| killer cell lectin-like receptor, subfamily A, member 4 | Klra7 (includes others) | 8,67E-11 | 9,492 |
| interferon regulatory factor 1 | IRF1 | 3,32E-11 | 9,195 |
| C-C motif chemokine ligand 2 | CCL2 | 1,75E-07 | 9,061 |
| cystatin F | CST7 | 6,32E-11 | 9,035 |
| complement C3 | C3 | 4,19E-11 | 9,01 |
| paired-Ig-like receptor A7 | Pira7 | 3,25E-10 | 8,986 |
| lymphocyte cytosolic protein 1 | LCP1 | 3,59E-10 | 8,915 |
| apolipoprotein L 11a | Apol10a (includes others) | 3,07E-06 | 8,876 |
| neutrophil cytosolic factor 4 | NCF4 | 2,26E-10 | 8,856 |
| apolipoprotein L 11a | Apol10a (includes others) | 3,36E-09 | 8,794 |
| complement C4B (Chido blood group) | C4A/C4B | 1,67E-12 | 8,75 |
| G protein-coupled receptor 132 | GPR132 | 2,49E-09 | 8,675 |
| CD300 molecule like family member f | CD300LF | 4,88E-05 | 8,617 |
| CD300c molecule | CD300C | 1,72E-08 | 8,56 |
| CD8b molecule | CD8B | 7,34E-08 | 8,391 |
| coiled-coil domain containing 88B | CCDC88B | 4,90E-12 | 8,315 |
| C-X-C motif chemokine ligand 6 | CXCL6 | 5,96E-06 | 8,314 |
| protein tyrosine phosphatase, non-receptor type 6 | PTPN6 | 4,27E-09 | 8,293 |
| integrin subunit alpha L | ITGAL | 1,00E-10 | 8,275 |
| histocompatibility 2, Q region locus 5 | H2-Q5 | 2,50E-11 | 8,251 |
| linker for activation of T-cells | LAT | 7,33E-11 | 8,228 |
| ADAM metallopeptidase domain 8 | ADAM8 | 2,17E-07 | 8,215 |
| solute carrier family 11 member 1 | SLC11A1 | 3,80E-10 | 8,157 |
| WD repeat and FYVE domain containing 1 | WDFY1 | 3,56E-09 | 8,152 |
| membrane spanning 4-domains A7 | MS4A7 | 5,90E-09 | 8,15 |
| interferon induced protein with tetratricopeptide repeats 2 | IFIT2 | 2,93E-09 | 8,148 |
| caspase recruitment domain family member 11 | CARD11 | 8,11E-13 | 8,078 |
| G protein-coupled receptor 65 | GPR65 | 2,16E-11 | 7,943 |
| myosin IF | MYO1F | 2,23E-07 | 7,921 |
| proteasome subunit beta 10 | PSMB10 | 1,70E-11 | 7,877 |
| caspase 1 | CASP1 | 5,21E-10 | 7,856 |
| killer cell lectin-like receptor, subfamily A, member 16 | Klra16 | 2,91E-08 | 7,767 |
| interleukin 18 binding protein | IL18BP | 7,18E-12 | 7,703 |
| poly(ADP-ribose) polymerase family member 14 | PARP14 | 1,44E-10 | 7,602 |
| paired-Ig-like receptor A11 | Pira11 | 6,02E-10 | 7,586 |
| FYN binding protein | FYB | 1,59E-11 | 7,536 |
| XIAP associated factor 1 | XAF1 | 4,31E-12 | 7,497 |
| S100 calcium binding protein A4 | S100A4 | 1,58E-09 | 7,426 |
| Fc fragment of IgE receptor Ig | FCER1G | 8,97E-10 | 7,395 |
| vav guanine nucleotide exchange factor 1 | VAV1 | 1,37E-10 | 7,382 |
| TIMP metallopeptidase inhibitor 1 | TIMP1 | 5,43E-06 | 7,336 |
| paired immunoglobin like type 2 receptor alpha | PILRA | 2,63E-09 | 7,318 |
| thymocyte selection associated family member 2 | THEMIS2 | 2,21E-11 | 7,312 |
| phospholipase D family member 4 | PLD4 | 8,96E-10 | 7,266 |
| receptor transporter protein 4 | RTP4 | 1,12E-10 | 7,252 |
| GLI pathogenesis related 1 | GLIPR1 | 3,83E-11 | 7,246 |
| protein tyrosine phosphatase, non-receptor type 22 | PTPN22 | 4,13E-09 | 7,243 |
| selectin P ligand | SELPLG | 2,71E-10 | 7,203 |
| SAM and HD domain containing deoxynucleoside triphosphate triphosphohydrolase 1 | SAMHD1 | 1,15E-10 | 7,138 |
| 2'-5' oligoadenylate synthetase-like 2 | Oasl2 | 6,17E-07 | 7,095 |
| formyl peptide receptor 2 | FPR2 | 1,08E-05 | 7,085 |
| C-type lectin domain family 7 member A | CLEC7A | 1,10E-06 | 7,057 |
| inhibitor of DNA binding 2, HLH protein | ID2 | 1,98E-08 | 7,027 |
| basic leucine zipper ATF-like transcription factor 3 | BATF3 | 3,68E-08 | 6,893 |
| leukocyte associated immunoglobulin like receptor 1 | LAIR1 | 1,48E-09 | 6,857 |
| baculoviral IAP repeat containing 5 | BIRC5 | 1,16E-07 | 6,851 |
| GTPase, very large interferon inducible 1 pseudogene | Gm4759 | 7,56E-09 | 6,846 |
| Fas ligand | FASLG | 1,99E-12 | 6,818 |
| expressed sequence AI661384 | AI661384 | 1,66E-09 | 6,746 |
| TAP binding protein like | TAPBPL | 9,33E-13 | 6,742 |
| caspase 4 | CASP4 | 3,96E-09 | 6,738 |
| vascular cell adhesion molecule 1 | VCAM1 | 1,22E-09 | 6,703 |
| RAS guanyl releasing protein 1 | RASGRP1 | 2,24E-10 | 6,684 |
| SLAM family member 9 | SLAMF9 | 2,80E-10 | 6,658 |
| LIM domain containing 2 | LIMD2 | 1,85E-09 | 6,569 |
| interleukin 10 receptor subunit alpha | IL10RA | 5,55E-10 | 6,558 |
| cytochrome b-245 beta chain | CYBB | 5,75E-09 | 6,541 |
| CD3e molecule | CD3E | 7,62E-11 | 6,453 |
| cytohesin 4 | CYTH4 | 1,54E-08 | 6,418 |
| myelin basic protein | MBP | 3,11E-12 | 6,415 |
| kinesin family member 21B | KIF21B | 1,83E-09 | 6,404 |
| integrin subunit alpha 4 | ITGA4 | 1,37E-09 | 6,397 |
| G protein subunit gamma 2 | GNG2 | 2,51E-10 | 6,37 |
| killer cell lectin like receptor D1 | KLRD1 | 2,50E-10 | 6,369 |
| leukocyte immunoglobulin like receptor B3 | LILRB3 | 2,97E-10 | 6,343 |
| CD48 molecule | CD48 | 9,82E-11 | 6,332 |
| IFI30, lysosomal thiol reductase | IFI30 | 7,95E-08 | 6,325 |
| G protein-coupled receptor 68 | GPR68 | 1,19E-08 | 6,315 |
| B cell leukemia/lymphoma 2 related protein A1c | Bcl2a1c | 1,88E-09 | 6,294 |
| heparanase | HPSE | 2,08E-09 | 6,287 |
| 2'-5'-oligoadenylate synthetase 1 | OAS1 | 2,56E-07 | 6,246 |
| C-X3-C motif chemokine receptor 1 | CX3CR1 | 2,83E-04 | 6,227 |
| complement C1q B chain | C1QB | 3,09E-10 | 6,21 |
| proline-serine-threonine phosphatase interacting protein 1 | PSTPIP1 | 1,94E-09 | 6,146 |
| colony stimulating factor 2 receptor beta common subunit | CSF2RB | 3,22E-08 | 6,135 |
| sorting nexin 20 | SNX20 | 2,39E-09 | 6,123 |
| marker of proliferation Ki-67 | MKI67 | 7,67E-09 | 6,092 |
| bone marrow stromal cell antigen 2 | Bst2 | 5,54E-09 | 6,09 |
| lysosomal protein transmembrane 5 | LAPTM5 | 6,52E-09 | 6,079 |
| solute carrier family 16 member 3 | SLC16A3 | 1,53E-09 | 6,068 |
| G protein-coupled receptor 171 | GPR171 | 2,64E-11 | 6,057 |
| 6-phosphofructo-2-kinase/fructose-2,6-biphosphatase 3 | PFKFB3 | 2,34E-08 | 6,05 |
| interleukin 2 receptor subunit gamma | IL2RG | 5,90E-09 | 6,01 |
| complement C1q A chain | C1QA | 3,04E-10 | 5,954 |
| deoxyribonuclease 1 like 3 | DNASE1L3 | 7,25E-10 | 5,95 |
| CD96 molecule | CD96 | 8,82E-12 | 5,886 |
| regulator of G-protein signaling 1 | RGS1 | 2,51E-10 | 5,846 |
| Epstein-Barr virus induced 3 | EBI3 | 2,46E-06 | 5,833 |
| coactosin like F-actin binding protein 1 | COTL1 | 1,04E-08 | 5,822 |
| RAB32, member RAS oncogene family | RAB32 | 1,27E-09 | 5,806 |
| SAM and SH3 domain containing 3 | SASH3 | 6,17E-11 | 5,805 |
| beta-2-microglobulin | B2M | 2,29E-08 | 5,801 |
| component of Sp100-rs | Csprs (includes others) | 9,35E-07 | 5,788 |
| CD300e molecule | CD300E | 4,00E-05 | 5,764 |
| ubiquitin specific peptidase 18 | USP18 | 8,98E-10 | 5,762 |
| 2'-5'-oligoadenylate synthetase 3 | OAS3 | 9,23E-10 | 5,757 |
| E2F transcription factor 2 | E2F2 | 4,07E-09 | 5,714 |
| C-type lectin domain family 4, member a3 | Clec4a3 | 4,69E-08 | 5,692 |
| semaphorin 4D | SEMA4D | 1,18E-08 | 5,67 |
| cell division cycle associated 8 | CDCA8 | 6,65E-09 | 5,647 |
| lysozyme | LYZ | 1,08E-07 | 5,644 |
| Src-like-adaptor | SLA | 8,28E-08 | 5,635 |
| 2'-5' oligoadenylate synthetase 1F | Oas1f | 1,06E-08 | 5,614 |
| TYRO protein tyrosine kinase binding protein | TYROBP | 3,11E-08 | 5,574 |
| interleukin 21 receptor | IL21R | 6,64E-09 | 5,563 |
| thromboxane A synthase 1 | TBXAS1 | 1,38E-09 | 5,549 |
| killer cell lectin like receptor G1 | KLRG1 | 6,99E-10 | 5,548 |
| major histocompatibility complex, class I, G | HLA-G | 2,63E-09 | 5,547 |
| Ras association domain family member 5 | RASSF5 | 2,08E-08 | 5,546 |
| killer cell lectin-like receptor subfamily A, member 23 | Klra23 | 3,32E-11 | 5,543 |
| pleckstrin | PLEK | 1,25E-07 | 5,495 |
| parvin gamma | PARVG | 1,67E-09 | 5,469 |
| cytohesin 1 interacting protein | CYTIP | 3,79E-07 | 5,465 |
| zinc finger protein 705A | ZNF705A | 1,29E-09 | 5,453 |
| complement C1q C chain | C1QC | 2,30E-09 | 5,449 |
| C-X-C motif chemokine ligand 13 | CXCL13 | 6,96E-07 | 5,449 |
| synaptotagmin like 3 | SYTL3 | 7,98E-08 | 5,416 |
| FCH domain only 1 | FCHO1 | 1,25E-09 | 5,386 |
| glia maturation factor gamma | GMFG | 1,16E-08 | 5,385 |
| SLAM family member 7 | SLAMF7 | 5,73E-11 | 5,385 |
| ecotropic viral integration site 2A | EVI2A | 3,74E-07 | 5,366 |
| C-type lectin domain family 9 member A | CLEC9A | 1,94E-08 | 5,36 |
| membrane spanning 4-domains A6A | MS4A6A | 1,15E-07 | 5,348 |
| WDFY family member 4 | WDFY4 | 1,36E-08 | 5,325 |
| tumor necrosis factor | TNF | 3,36E-08 | 5,313 |
| semaphorin 4A | SEMA4A | 1,84E-09 | 5,307 |
| pannexin 1 | PANX1 | 3,68E-09 | 5,305 |
| TNF receptor superfamily member 18 | TNFRSF18 | 1,70E-10 | 5,276 |
| ubiquitin conjugating enzyme E2 L6 | UBE2L6 | 1,68E-09 | 5,253 |
| tumor necrosis factor superfamily member 10 | TNFSF10 | 3,27E-07 | 5,247 |
| leupaxin | LPXN | 2,42E-07 | 5,238 |
| phospholipase A2 group VII | PLA2G7 | 3,63E-07 | 5,237 |
| Fc fragment of IgG receptor Ia | FCGR1A | 3,24E-09 | 5,23 |
| mitogen-activated protein kinase kinase kinase kinase 1 | MAP4K1 | 2,65E-11 | 5,227 |
| Thy-1 cell surface antigen | THY1 | 1,19E-08 | 5,226 |
| colony stimulating factor 2 receptor beta common subunit | CSF2RB | 5,26E-07 | 5,217 |
| protein tyrosine phosphatase, non-receptor type 18 | PTPN18 | 1,42E-07 | 5,19 |
| myosin heavy chain 7 | MYH7 | 4,87E-05 | 5,185 |
| MX dynamin-like GTPase 1 | Mx1/Mx2 | 2,38E-07 | 5,164 |
| hematopoietic cell-specific Lyn substrate 1 | HCLS1 | 1,72E-10 | 5,16 |
| tubulin beta 3 class III | TUBB3 | 7,13E-06 | 5,122 |
| fermitin family member 3 | FERMT3 | 3,07E-10 | 5,093 |
| toll like receptor 2 | TLR2 | 2,30E-09 | 5,085 |
| interferon induced protein 44 | IFI44 | 8,51E-09 | 5,079 |
| transporter 2, ATP binding cassette subfamily B member | TAP2 | 2,09E-10 | 5,078 |
| tripartite motif containing 21 | TRIM21 | 2,26E-10 | 5,054 |
| MEFV, pyrin innate immunity regulator | MEFV | 3,06E-07 | 5,038 |
| phosphatidylinositol-4,5-bisphosphate 3-kinase catalytic subunit delta | PIK3CD | 2,24E-07 | 5,035 |
| NCK associated protein 1 like | NCKAP1L | 1,26E-08 | 5,011 |
| tetraspanin 32 | TSPAN32 | 1,05E-09 | 5,002 |
| Rho GTPase activating protein 45 | ARHGAP45 | 9,64E-10 | 4,979 |
| poly(ADP-ribose) polymerase family member 9 | PARP9 | 8,46E-10 | 4,971 |
| interferon regulatory factor 5 | IRF5 | 1,37E-09 | 4,921 |
| apolipoprotein L 7e | Apol7e (includes others) | 6,99E-11 | 4,913 |
| C-X-C motif chemokine ligand 16 | CXCL16 | 9,29E-09 | 4,912 |
| leucine rich repeat containing 25 | LRRC25 | 9,36E-08 | 4,882 |
| Rho GTPase activating protein 4 | ARHGAP4 | 2,98E-09 | 4,847 |
| TNF receptor associated factor 1 | TRAF1 | 2,02E-08 | 4,82 |
| ubiquitin like modifier activating enzyme 7 | UBA7 | 2,99E-09 | 4,82 |
| FYVE, RhoGEF and PH domain containing 2 | FGD2 | 7,52E-09 | 4,815 |
| guanylate binding protein family member 6 | GBP6 | 3,86E-09 | 4,797 |
| signal transducer and activator of transcription 2 | STAT2 | 9,25E-11 | 4,792 |
| beta-ureidopropionase 1 | UPB1 | 5,43E-10 | 4,789 |
| membrane-spanning 4-domains, subfamily A, member 6C | Ms4a6c | 1,13E-06 | 4,789 |
| N-acylethanolamine acid amidase | NAAA | 5,85E-09 | 4,778 |
| olfactory receptor family 51 subfamily A member 7 | OR51A7 | 5,73E-11 | 4,746 |
| unc-93 homolog B1 (C. elegans) | UNC93B1 | 1,52E-09 | 4,743 |
| galectin 3 binding protein | LGALS3BP | 2,31E-10 | 4,715 |
| schlafen family member 13 | SLFN13 | 8,59E-10 | 4,708 |
| C-C motif chemokine receptor 2 | CCR2 | 8,16E-09 | 4,698 |
| potassium calcium-activated channel subfamily N member 4 | KCNN4 | 3,68E-09 | 4,696 |
| GTPase, IMAP family member 7 | GIMAP7 | 6,10E-08 | 4,681 |
| inducible T-cell costimulator | ICOS | 4,69E-06 | 4,667 |
| protein tyrosine phosphatase, non-receptor type 7 | PTPN7 | 9,46E-10 | 4,654 |
| CD69 molecule | CD69 | 4,01E-10 | 4,645 |
| GTPase, very large interferon inducible 1 | Gvin1 (includes others) | 4,81E-05 | 4,645 |
| histone cluster 1, H1b | Hist1h1b | 3,52E-07 | 4,623 |
| schlafen 2 | Slfn2 | 5,09E-08 | 4,612 |
| histocompatibility 2, M region locus 11 | H2-M11 | 2,09E-06 | 4,598 |
| 2'-5'-oligoadenylate synthetase like | OASL | 4,70E-07 | 4,569 |
| deltex E3 ubiquitin ligase 3L | DTX3L | 1,57E-08 | 4,541 |
| interferon gamma inducible protein 16 | IFI16 | 9,24E-10 | 4,54 |
| beta-1,4-N-acetyl-galactosaminyltransferase 1 | B4GALNT1 | 1,74E-09 | 4,533 |
| polypeptide N-acetylgalactosaminyltransferase 6 | GALNT6 | 4,84E-06 | 4,526 |
| complement C1r | C1R | 1,51E-10 | 4,521 |
| regulator of G-protein signaling 14 | RGS14 | 2,97E-10 | 4,52 |
| zinc finger MYND-type containing 15 | ZMYND15 | 3,07E-09 | 4,512 |
| programmed cell death 1 ligand 2 | PDCD1LG2 | 3,57E-06 | 4,504 |
| tumor necrosis factor superfamily member 13b | TNFSF13B | 2,23E-09 | 4,503 |
| transmembrane protein 173 | TMEM173 | 3,59E-08 | 4,495 |
| suppressor of cytokine signaling 3 | SOCS3 | 1,86E-06 | 4,492 |
| Wiskott-Aldrich syndrome | WAS | 5,10E-10 | 4,479 |
| histone cluster 1 H2A family member b | HIST1H2AB | 1,58E-08 | 4,451 |
| lymphocyte antigen 9 | LY9 | 3,04E-07 | 4,411 |
| signal transducer and activator of transcription 1 | STAT1 | 1,58E-09 | 4,407 |
| ras homolog family member F, filopodia associated | RHOF | 3,69E-09 | 4,385 |
| cyclin B2 | CCNB2 | 2,41E-06 | 4,377 |
| C-type lectin domain family 4 member D | CLEC4D | 5,05E-04 | 4,368 |
| ring finger protein 213 | RNF213 | 8,77E-08 | 4,344 |
| DENN domain containing 1C | DENND1C | 1,48E-08 | 4,334 |
| uridine phosphorylase 1 | UPP1 | 1,22E-05 | 4,29 |
| Fc fragment of IgG receptor IIa | FCGR2A | 1,03E-07 | 4,289 |
| toll-like receptor 13 | Tlr13 | 2,43E-07 | 4,288 |
| unc-13 homolog D | UNC13D | 1,72E-08 | 4,287 |
| pleckstrin and Sec7 domain containing 4 | PSD4 | 9,65E-09 | 4,281 |
| CD86 molecule | CD86 | 4,78E-10 | 4,279 |
| villin like | VILL | 3,95E-08 | 4,279 |
| dedicator of cytokinesis 10 | DOCK10 | 2,88E-09 | 4,272 |
| fibrinogen like 2 | FGL2 | 2,63E-09 | 4,272 |
| tryptophanyl-tRNA synthetase | WARS | 1,99E-10 | 4,271 |
| LY6/PLAUR domain containing 6B | LYPD6B | 1,63E-09 | 4,264 |
| C-type lectin domain family 4 member C | CLEC4C | 6,24E-07 | 4,253 |
| Rho GTPase activating protein 9 | ARHGAP9 | 6,54E-09 | 4,249 |
| Fc fragment of IgG receptor IIb | FCGR2B | 1,90E-06 | 4,247 |
| SH3 domain binding protein 2 | SH3BP2 | 1,43E-09 | 4,238 |
| TRAF interacting protein with forkhead associated domain | TIFA | 6,72E-06 | 4,233 |
| CD68 molecule | CD68 | 5,72E-08 | 4,231 |
| polo like kinase 1 | PLK1 | 2,01E-07 | 4,224 |
| lymphoid restricted membrane protein | LRMP | 2,62E-10 | 4,221 |
| granzyme C | Gzmc | 1,78E-07 | 4,199 |
| amyloid beta precursor protein binding family B member 1 interacting protein | APBB1IP | 1,41E-08 | 4,196 |
| p21 (RAC1) activated kinase 1 | PAK1 | 4,35E-10 | 4,194 |
| CD44 molecule (Indian blood group) | CD44 | 1,69E-06 | 4,191 |
| poly(ADP-ribose) polymerase family member 10 | PARP10 | 7,53E-11 | 4,178 |
| TNF alpha induced protein 3 | TNFAIP3 | 1,92E-06 | 4,178 |
| tripartite motif-containing 30A | Trim30a/Trim30d | 1,46E-08 | 4,169 |
| dipeptidase 2 | DPEP2 | 3,97E-06 | 4,153 |
| C-type lectin domain family 6 member A | CLEC6A | 3,16E-07 | 4,121 |
| protein C receptor | PROCR | 4,75E-08 | 4,102 |
| dedicator of cytokinesis 2 | DOCK2 | 4,35E-10 | 4,083 |
| napsin A aspartic peptidase | NAPSA | 2,28E-08 | 4,083 |
| Rho GTPase activating protein 30 | ARHGAP30 | 4,03E-08 | 4,047 |
| cathepsin C | CTSC | 6,55E-09 | 4,038 |
| NFAT activating protein with ITAM motif 1 | NFAM1 | 1,15E-06 | 4,032 |
| CD53 molecule | CD53 | 2,13E-09 | 4,03 |
| FYVE, RhoGEF and PH domain containing 3 | FGD3 | 6,72E-08 | 4,023 |
| C-C motif chemokine ligand 19 | CCL19 | 1,02E-06 | 4,022 |
| NFKB inhibitor epsilon | NFKBIE | 2,14E-08 | 4,022 |
| CD300 molecule like family member d | CD300LD | 2,31E-07 | 4,006 |
| toll like receptor 1 | TLR1 | 3,27E-07 | 3,977 |
| immunoglobulin superfamily member 6 | IGSF6 | 2,45E-06 | 3,967 |
| DENN domain containing 2D | DENND2D | 4,42E-11 | 3,961 |
| cyclin A2 | CCNA2 | 1,63E-07 | 3,949 |
| hydrogen voltage gated channel 1 | HVCN1 | 1,08E-06 | 3,903 |
| family with sequence similarity 105 member A | FAM105A | 7,99E-08 | 3,894 |
| neuralized E3 ubiquitin protein ligase 3 | NEURL3 | 1,26E-06 | 3,893 |
| AT-hook transcription factor | AKNA | 2,67E-09 | 3,892 |
| triggering receptor expressed on myeloid cells 2 | TREM2 | 1,31E-06 | 3,857 |
| BH3 interacting domain death agonist | BID | 2,98E-09 | 3,85 |
| activating transcription factor 3 | ATF3 | 1,46E-04 | 3,849 |
| TNF alpha induced protein 8 like 2 | TNFAIP8L2 | 9,57E-09 | 3,844 |
| gap junction protein delta 3 | GJD3 | 6,83E-08 | 3,837 |
| purinergic receptor P2X 7 | P2RX7 | 3,51E-07 | 3,837 |
| FXYD domain containing ion transport regulator 5 | FXYD5 | 7,99E-08 | 3,836 |
| RAB19, member RAS oncogene family | RAB19 | 1,49E-08 | 3,833 |
| zinc finger CCCH-type containing 12D | ZC3H12D | 5,80E-07 | 3,827 |
| peptidyl arginine deiminase 2 | PADI2 | 3,47E-09 | 3,807 |
| NFKB inhibitor delta | NFKBID | 1,37E-07 | 3,805 |
| CD84 molecule | CD84 | 8,76E-06 | 3,8 |
| docking protein 2 | DOK2 | 1,41E-07 | 3,796 |
| NLR family, pyrin domain containing 1A | Nlrp1a | 6,55E-09 | 3,782 |
| lymphocyte activating 3 | LAG3 | 3,96E-09 | 3,779 |
| POU domain, class 3, transcription factor 1 | Pou3f1 | 1,38E-07 | 3,775 |
| growth factor independent 1 transcriptional repressor | GFI1 | 1,84E-09 | 3,772 |
| early growth response 2 | EGR2 | 9,04E-07 | 3,764 |
| basic leucine zipper ATF-like transcription factor | BATF | 1,39E-08 | 3,74 |
| formyl peptide receptor 1 | FPR1 | 1,90E-06 | 3,725 |
| T cell receptor beta, variable 13-2 | Trbv13-2 | 1,45E-09 | 3,723 |
| interferon induced with helicase C domain 1 | IFIH1 | 3,38E-09 | 3,719 |
| ATP binding cassette subfamily G member 1 | ABCG1 | 2,50E-07 | 3,716 |
| interleukin 18 receptor 1 | IL18R1 | 8,76E-08 | 3,714 |
| purinergic receptor P2Y10 | P2RY10 | 4,65E-09 | 3,698 |
| transmembrane protein 106A | TMEM106A | 8,71E-07 | 3,697 |
| Rho GDP dissociation inhibitor beta | ARHGDIB | 4,09E-08 | 3,696 |
| C-C motif chemokine ligand 3 like 3 | CCL3L3 | 3,46E-04 | 3,681 |
| CD40 molecule | CD40 | 3,62E-07 | 3,676 |
| chromosome 16 open reading frame 54 | C16orf54 | 8,40E-09 | 3,674 |
| transforming growth factor beta induced | TGFBI | 5,84E-05 | 3,674 |
| nucleoporin 210 | NUP210 | 1,55E-08 | 3,647 |
| colony stimulating factor 2 receptor alpha subunit | CSF2RA | 6,55E-09 | 3,638 |
| coronin 2A | CORO2A | 5,01E-08 | 3,634 |
| centromere protein E | CENPE | 1,45E-07 | 3,628 |
| intercellular adhesion molecule 1 | ICAM1 | 6,09E-08 | 3,628 |
| ATPase phospholipid transporting 8B4 (putative) | ATP8B4 | 5,21E-08 | 3,613 |
| sorting nexin 10 | SNX10 | 9,63E-07 | 3,612 |
| pyrimidinergic receptor P2Y6 | P2RY6 | 1,48E-07 | 3,605 |
| linker for activation of T-cells family member 2 | LAT2 | 2,69E-07 | 3,599 |
| arrestin beta 2 | ARRB2 | 7,40E-08 | 3,595 |
| topoisomerase (DNA) II alpha | TOP2A | 2,30E-08 | 3,587 |
| acyloxyacyl hydrolase | AOAH | 3,62E-07 | 3,586 |
| arachidonate 5-lipoxygenase activating protein | ALOX5AP | 7,79E-08 | 3,579 |
| G-protein signaling modulator 3 | GPSM3 | 3,65E-08 | 3,567 |
| B-cell CLL/lymphoma 3 | BCL3 | 1,01E-06 | 3,563 |
| TNF receptor superfamily member 4 | TNFRSF4 | 3,66E-06 | 3,558 |
| G protein-coupled receptor 18 | GPR18 | 6,05E-09 | 3,557 |
| transmembrane protein 56 | TMEM56 | 6,12E-07 | 3,556 |
| histidine decarboxylase | HDC | 7,49E-05 | 3,542 |
| argininosuccinate synthase 1 | ASS1 | 2,23E-07 | 3,54 |
| BCL2 like 14 | BCL2L14 | 4,31E-10 | 3,537 |
| inositol polyphosphate-5-phosphatase D | INPP5D | 2,06E-08 | 3,529 |
| DExD/H-box helicase 58 | DDX58 | 1,58E-08 | 3,526 |
| neutrophil cytosolic factor 1 | NCF1 | 8,58E-10 | 3,511 |
| insulin receptor related receptor | INSRR | 3,59E-08 | 3,493 |
| Src like adaptor 2 | SLA2 | 2,25E-08 | 3,49 |
| histone cluster 1 H2A family member g | HIST1H2AG | 4,70E-08 | 3,486 |
| cell adhesion molecule 1 | CADM1 | 3,97E-09 | 3,484 |
| Rho GTPase activating protein 15 | ARHGAP15 | 1,91E-06 | 3,482 |
| phosphatidylinositol-3,4,5-trisphosphate dependent Rac exchange factor 1 | PREX1 | 1,86E-07 | 3,469 |
| glycine amidinotransferase | GATM | 8,51E-07 | 3,465 |
| aldolase, fructose-bisphosphate C | ALDOC | 4,98E-07 | 3,457 |
| EF-hand domain family member D2 | EFHD2 | 2,28E-07 | 3,453 |
| absent in melanoma 1 | AIM1 | 4,45E-09 | 3,449 |
| adenylate cyclase 7 | ADCY7 | 2,04E-05 | 3,446 |
| Ras and Rab interactor like | RINL | 1,15E-07 | 3,443 |
| aldehyde dehydrogenase 1 family member A2 | ALDH1A2 | 3,76E-05 | 3,42 |
| ubiquitin like with PHD and ring finger domains 1 | UHRF1 | 5,12E-06 | 3,418 |
| RB transcriptional corepressor like 1 | RBL1 | 1,16E-08 | 3,398 |
| Rac GTPase activating protein 1 | RACGAP1 | 2,62E-08 | 3,385 |
| radical S-adenosyl methionine domain containing 2 | RSAD2 | 3,19E-05 | 3,382 |
| SP140 nuclear body protein | SP140 | 2,25E-07 | 3,382 |
| proteasome activator subunit 2 | PSME2 | 2,53E-10 | 3,372 |
| tripartite motif containing 14 | TRIM14 | 7,82E-08 | 3,361 |
| interferon induced protein 35 | IFI35 | 2,47E-10 | 3,34 |
| paired immunoglobin-like type 2 receptor beta | PILRB | 2,14E-08 | 3,338 |
| GLI pathogenesis related 2 | GLIPR2 | 2,39E-07 | 3,327 |
| GTPase, IMAP family member 4 | GIMAP4 | 1,34E-04 | 3,317 |
| signal transducer and activator of transcription 4 | STAT4 | 8,10E-09 | 3,308 |
| leukocyte specific transcript 1 | Lst1 | 2,45E-06 | 3,295 |
| cell division cycle associated 5 | CDCA5 | 2,13E-06 | 3,289 |
| ribosomal protein S6 kinase A1 | RPS6KA1 | 4,47E-09 | 3,284 |
| gasdermin D | GSDMD | 2,71E-10 | 3,272 |
| septin 1 | SEPT1 | 3,47E-09 | 3,27 |
| fucosyltransferase 7 | FUT7 | 4,76E-08 | 3,262 |
| phorbol-12-myristate-13-acetate-induced protein 1 | Pmaip1 | 5,47E-07 | 3,26 |
| X-C motif chemokine ligand 1 | XCL1 | 2,80E-07 | 3,258 |
| activating transcription factor 7 | ATF7 | 2,21E-07 | 3,252 |
| protein regulator of cytokinesis 1 | PRC1 | 4,40E-06 | 3,249 |
| killer cell lectin-like receptor, subfamily A, member 15 | Klra15 | 2,65E-07 | 3,241 |
| NLR family apoptosis inhibitory protein | NAIP | 7,13E-08 | 3,24 |
| kelch repeat and BTB domain containing 11 | KBTBD11 | 5,40E-07 | 3,235 |
| eukaryotic translation initiation factor 2 alpha kinase 2 | EIF2AK2 | 1,08E-09 | 3,233 |
| ADP ribosylation factor like GTPase 5C | ARL5C | 8,29E-05 | 3,231 |
| complement C1r | C1R | 6,05E-06 | 3,228 |
| thymosin, beta 4, X chromosome | Tmsb4x (includes others) | 2,01E-06 | 3,223 |
| SH3 domain binding glutamate rich protein like 3 | SH3BGRL3 | 5,97E-08 | 3,214 |
| small proline rich protein 1A | SPRR1A | 1,27E-04 | 3,214 |
| DNA damage inducible transcript 3 | DDIT3 | 8,56E-09 | 3,212 |
| regulator of G-protein signaling 16 | RGS16 | 4,65E-05 | 3,206 |
| FGR proto-oncogene, Src family tyrosine kinase | FGR | 1,01E-06 | 3,202 |
| insulin like 6 | INSL6 | 1,31E-06 | 3,2 |
| T cell receptor alpha constant | Trac | 6,06E-09 | 3,199 |
| cysteinyl leukotriene receptor 2 | CYSLTR2 | 9,78E-08 | 3,182 |
| protein tyrosine kinase 2 beta | PTK2B | 8,68E-07 | 3,18 |
| chromosome 19 open reading frame 38 | C19orf38 | 2,26E-05 | 3,178 |
| perforin 1 | PRF1 | 6,86E-08 | 3,174 |
| lymphocyte antigen 6 complex, locus A | Ly6a (includes others) | 1,67E-04 | 3,168 |
| secreted frizzled related protein 2 | SFRP2 | 4,88E-05 | 3,147 |
| protein kinase C beta | PRKCB | 2,80E-07 | 3,14 |
| syntaxin binding protein 2 | STXBP2 | 3,14E-07 | 3,139 |
| hepatitis A virus cellular receptor 2 | HAVCR2 | 7,46E-07 | 3,134 |
| docking protein 1 | DOK1 | 2,85E-07 | 3,127 |
| CD200 receptor 1 like | CD200R1L | 1,64E-06 | 3,126 |
| ubiquitin associated and SH3 domain containing B | UBASH3B | 3,21E-05 | 3,125 |
| C-type lectin domain family 2, member d | Clec2d (includes others) | 1,72E-03 | 3,106 |
| TNF alpha induced protein 2 | TNFAIP2 | 2,38E-05 | 3,104 |
| ubiquitin conjugating enzyme E2 C | UBE2C | 3,43E-07 | 3,103 |
| cathepsin Z | CTSZ | 1,56E-07 | 3,101 |
| C-type lectin domain family 10 member A | CLEC10A | 2,24E-08 | 3,099 |
| xanthine dehydrogenase | XDH | 2,99E-06 | 3,082 |
| T cell receptor beta, variable 16 | Trbv16 | 3,42E-05 | 3,061 |
| Bruton tyrosine kinase | BTK | 1,94E-08 | 3,052 |
| D-box binding PAR bZIP transcription factor | DBP | 7,01E-04 | 3,048 |
| capping actin protein, gelsolin like | CAPG | 1,63E-08 | 3,045 |
| microsomal glutathione S-transferase 2 | MGST2 | 4,90E-07 | 3,042 |
| cell division cycle associated 3 | CDCA3 | 5,81E-05 | 3,039 |
| C-C motif chemokine receptor like 2 | CCRL2 | 6,65E-09 | 3,025 |
| protein tyrosine phosphatase, receptor type, f polypeptide (PTPRF), interacting protein (liprin), alpha 4 | Ppfia4 | 2,12E-06 | 3,02 |
| transmembrane protein 229B | TMEM229B | 7,53E-07 | 3,019 |
| minichromosome maintenance complex component 5 | MCM5 | 6,98E-07 | 3,007 |
| kinesin family member 22 | KIF22 | 5,71E-05 | 2,994 |
| neutrophil cytosolic factor 2 | NCF2 | 3,02E-06 | 2,986 |
| major histocompatibility complex, class II, DO alpha | HLA-DOA | 1,30E-07 | 2,983 |
| phosphoinositide-3-kinase regulatory subunit 5 | PIK3R5 | 1,61E-07 | 2,97 |
| phospholipase C gamma 2 | PLCG2 | 2,20E-07 | 2,965 |
| apolipoprotein B mRNA editing enzyme catalytic subunit 1 | APOBEC1 | 4,06E-05 | 2,961 |
| proline rich 5 like | PRR5L | 1,02E-05 | 2,955 |
| apolipoprotein L 11a | Apol10a (includes others) | 6,42E-07 | 2,951 |
| protein phosphatase 2 regulatory subunit Bgamma | PPP2R2C | 4,59E-06 | 2,949 |
| methylenetetrahydrofolate dehydrogenase (NADP+ dependent) 1-like | MTHFD1L | 6,59E-05 | 2,935 |
| sorting nexin 22 | SNX22 | 3,72E-04 | 2,928 |
| TAP binding protein | TAPBP | 2,56E-07 | 2,928 |
| F-box and WD-40 domain protein 17 | Fbxw17 | 3,74E-08 | 2,926 |
| CD226 molecule | CD226 | 2,01E-07 | 2,924 |
| archaelysin family metallopeptidase 1 | AMZ1 | 4,35E-05 | 2,916 |
| poly(ADP-ribose) polymerase family member 12 | PARP12 | 2,07E-07 | 2,915 |
| podocan like 1 | PODNL1 | 6,16E-07 | 2,894 |
| mitochondrial calcium uniporter dominant negative beta subunit | MCUB | 3,13E-06 | 2,893 |
| expressed sequence AI447881 | AI447881 | 5,93E-09 | 2,887 |
| killer cell lectin-like receptor subfamily B member 1F | Klrb1f | 2,78E-06 | 2,883 |
| CD300c molecule | CD300C | 1,27E-06 | 2,88 |
| lymphocyte cytosolic protein 2 | LCP2 | 1,07E-05 | 2,879 |
| N-myc and STAT interactor | NMI | 2,93E-09 | 2,872 |
| fidgetin like 1 | FIGNL1 | 3,85E-05 | 2,865 |
| sterile alpha motif domain containing 9 like | SAMD9L | 3,26E-06 | 2,865 |
| DNA damage regulated autophagy modulator 1 | DRAM1 | 2,17E-06 | 2,861 |
| centromere protein J | CENPJ | 6,42E-07 | 2,855 |
| receptor interacting serine/threonine kinase 3 | RIPK3 | 6,25E-06 | 2,845 |
| programmed cell death 1 | PDCD1 | 3,15E-07 | 2,844 |
| ribonuclease A family member k6 | RNASE6 | 6,11E-07 | 2,844 |
| apolipoprotein C2 | APOC2 | 2,08E-03 | 2,837 |
| C-C motif chemokine ligand 1 | CCL1 | 2,85E-05 | 2,835 |
| growth differentiation factor 15 | GDF15 | 2,21E-03 | 2,831 |
| chloride intracellular channel 1 | CLIC1 | 6,84E-06 | 2,827 |
| T cell receptor beta, variable 13-3 | Trbv13-3 | 1,74E-04 | 2,824 |
| embigin | EMB | 6,22E-03 | 2,822 |
| transmembrane protein 140 | TMEM140 | 5,71E-06 | 2,821 |
| nucleolar and spindle associated protein 1 | NUSAP1 | 2,70E-07 | 2,815 |
| AXL receptor tyrosine kinase | AXL | 3,95E-08 | 2,807 |
| protein kinase C delta | PRKCD | 6,83E-08 | 2,805 |
| adaptor related protein complex 1 sigma 3 subunit | AP1S3 | 1,54E-08 | 2,801 |
| capping protein (actin filament), gelsolin-like pseudogene | 5730416F02Rik | 5,87E-07 | 2,795 |
| src kinase associated phosphoprotein 2 | SKAP2 | 1,18E-06 | 2,792 |
| minichromosome maintenance complex component 6 | MCM6 | 1,82E-08 | 2,786 |
| transmembrane and tetratricopeptide repeat containing 4 | TMTC4 | 2,11E-08 | 2,785 |
| epithelial membrane protein 3 | EMP3 | 1,77E-05 | 2,784 |
| E2F transcription factor 1 | E2F1 | 1,12E-05 | 2,782 |
| serum amyloid A1 | SAA1 | 3,73E-02 | 2,78 |
| dickkopf WNT signaling pathway inhibitor 3 | DKK3 | 2,45E-06 | 2,767 |
| acyl-CoA synthetase long-chain family member 5 | ACSL5 | 2,37E-09 | 2,759 |
| SPC25, NDC80 kinetochore complex component | SPC25 | 3,56E-06 | 2,758 |
| interleukin 1 beta | IL1B | 1,01E-02 | 2,757 |
| MYB proto-oncogene like 2 | MYBL2 | 3,76E-06 | 2,756 |
| protein tyrosine phosphatase, receptor type O | PTPRO | 2,35E-06 | 2,745 |
| family with sequence similarity 107 member B | FAM107B | 2,87E-07 | 2,743 |
| interferon regulatory factor 9 | IRF9 | 6,11E-08 | 2,732 |
| Lck interacting transmembrane adaptor 1 | LIME1 | 1,08E-06 | 2,729 |
| cyclin dependent kinase inhibitor 2A | CDKN2A | 1,72E-06 | 2,728 |
| SH2 domain containing 2A | SH2D2A | 3,47E-05 | 2,722 |
| synaptotagmin like 1 | SYTL1 | 1,22E-07 | 2,72 |
| cytochrome b-245 alpha chain | CYBA | 1,28E-07 | 2,718 |
| T-cell immune regulator 1, ATPase H+ transporting V0 subunit a3 | TCIRG1 | 8,91E-07 | 2,717 |
| mucolipin 2 | MCOLN2 | 2,58E-04 | 2,716 |
| cytokine receptor-like factor 2 | CRLF2 | 7,05E-07 | 2,713 |
| T cell receptor gamma, constant 1 | Tcrg-C1 | 2,41E-05 | 2,71 |
| signal regulatory protein alpha | SIRPA | 1,74E-05 | 2,709 |
| adenosine deaminase, RNA specific | ADAR | 1,58E-08 | 2,707 |
| SAM domain, SH3 domain and nuclear localization signals 1 | SAMSN1 | 1,11E-05 | 2,699 |
| guanine deaminase | GDA | 1,09E-04 | 2,693 |
| macrophage scavenger receptor 1 | MSR1 | 7,38E-07 | 2,692 |
| heme oxygenase 1 | HMOX1 | 1,90E-03 | 2,686 |
| serpin family G member 1 | SERPING1 | 4,70E-08 | 2,685 |
| translocator protein | TSPO | 8,77E-08 | 2,676 |
| T cell receptor beta, variable 15 | Trbv15 | 2,21E-03 | 2,673 |
| tripartite motif containing 59 | TRIM59 | 9,39E-07 | 2,671 |
| mixed lineage kinase domain like pseudokinase | MLKL | 7,94E-07 | 2,668 |
| RNA binding motif protein 47 | RBM47 | 4,39E-06 | 2,668 |
| tumor necrosis factor superfamily member 14 | TNFSF14 | 2,25E-07 | 2,667 |
| brain abundant membrane attached signal protein 1 | BASP1 | 3,33E-05 | 2,663 |
| regulator of G-protein signaling 10 | RGS10 | 2,21E-05 | 2,661 |
| lamin B1 | LMNB1 | 4,22E-05 | 2,657 |
| transmembrane protein 51 | TMEM51 | 1,19E-05 | 2,653 |
| IKAROS family zinc finger 1 | IKZF1 | 1,67E-08 | 2,651 |
| vimentin | VIM | 3,79E-05 | 2,645 |
| syntaxin 11 | STX11 | 1,06E-04 | 2,643 |
| dipeptidase 2 | DPEP2 | 4,62E-05 | 2,638 |
| signaling threshold regulating transmembrane adaptor 1 | SIT1 | 2,94E-08 | 2,635 |
| potassium two pore domain channel subfamily K member 13 | KCNK13 | 1,36E-06 | 2,622 |
| serine/threonine kinase 17b | STK17B | 1,69E-05 | 2,618 |
| methylenetetrahydrofolate dehydrogenase (NADP+ dependent) 2, methenyltetrahydrofolate cyclohydrolase | MTHFD2 | 3,47E-06 | 2,616 |
| Rho family GTPase 1 | RND1 | 2,84E-05 | 2,616 |
| interleukin 3 receptor subunit alpha | IL3RA | 3,07E-06 | 2,612 |
| proteasome activator subunit 1 | PSME1 | 1,20E-08 | 2,61 |
| alanyl aminopeptidase, membrane | ANPEP | 3,53E-05 | 2,608 |
| C-type lectin domain family 4 member C | CLEC4C | 1,98E-04 | 2,601 |
| SH2 domain containing 1A | SH2D1A | 5,99E-06 | 2,601 |
| B-cell CLL/lymphoma 11B | BCL11B | 1,70E-07 | 2,598 |
| interferon gamma inducible protein 16 | IFI16 | 3,22E-04 | 2,597 |
| high mobility group box 2 | HMGB2 | 5,90E-04 | 2,594 |
| T cell receptor beta, variable 12-2 | Trbv12-2 | 1,57E-07 | 2,593 |
| killer cell lectin-like receptor family E member 1 | Klre1 | 1,15E-03 | 2,592 |
| serglycin | Srgn | 1,05E-05 | 2,592 |
| ring finger protein 19B | RNF19B | 1,83E-03 | 2,587 |
| adenylate cyclase associated protein 1 | CAP1 | 2,15E-06 | 2,581 |
| killer cell lectin like receptor B1 | KLRB1 | 4,23E-07 | 2,579 |
| DExH-box helicase 58 | DHX58 | 4,82E-05 | 2,566 |
| Ras association domain family member 2 | RASSF2 | 6,58E-05 | 2,55 |
| peptidoglycan recognition protein 2 | PGLYRP2 | 1,99E-07 | 2,547 |
| phospholipase A2 group IVA | PLA2G4A | 9,76E-06 | 2,547 |
| shisa family member 5 | SHISA5 | 5,56E-08 | 2,538 |
| chromatin licensing and DNA replication factor 1 | CDT1 | 1,14E-03 | 2,537 |
| complement C5a receptor 1 | C5AR1 | 2,44E-06 | 2,537 |
| T cell receptor beta, variable 14 | Trbv14 | 9,78E-04 | 2,533 |
| SERTA domain containing 2 | SERTAD2 | 6,34E-04 | 2,528 |
| mab-21 like 3 | MAB21L3 | 1,89E-06 | 2,525 |
| SH3 domain binding protein 1 | SH3BP1 | 1,68E-05 | 2,525 |
| T cell receptor beta, variable 29 | Trbv29 | 2,27E-03 | 2,525 |
| GEM interacting protein | GMIP | 1,72E-06 | 2,516 |
| 2-cell-stage, variable group, member 3 | Tcstv3 | 2,72E-06 | 2,512 |
| abnormal spindle microtubule assembly | ASPM | 2,85E-06 | 2,511 |
| T cell receptor beta, variable 10 | Trbv4 | 1,11E-04 | 2,511 |
| 2'-5' oligoadenylate synthetase 1D | Oas1d (includes others) | 3,74E-06 | 2,51 |
| RAB43, member RAS oncogene family | RAB43 | 3,53E-05 | 2,507 |
| cyclin B1 | CCNB1 | 1,39E-03 | 2,506 |
| glucose-6-phosphate dehydrogenase | G6PD | 1,18E-08 | 2,504 |
| Ras association domain family member 4 | RASSF4 | 7,85E-04 | 2,499 |
| UDP glucuronosyltransferase family 1 member A6 | UGT1A6 | 2,89E-03 | 2,498 |
| lymphocyte antigen 6 complex pseudogene | 9030619P08Rik | 6,95E-07 | 2,493 |
| unc-5 family C-terminal like | UNC5CL | 4,19E-06 | 2,488 |
| CD160 molecule | CD160 | 2,43E-06 | 2,485 |
| interleukin 21 | IL21 | 3,17E-03 | 2,484 |
| C-C motif chemokine receptor 7 | CCR7 | 5,91E-05 | 2,483 |
| cytidine/uridine monophosphate kinase 2 | CMPK2 | 3,67E-06 | 2,479 |
| C-C motif chemokine ligand 17 | CCL17 | 1,06E-04 | 2,477 |
| deltex E3 ubiquitin ligase 3L | DTX3L | 4,31E-05 | 2,472 |
| TGFB induced factor homeobox 1 | TGIF1 | 2,14E-06 | 2,466 |
| RNA binding motif protein 43 | RBM43 | 2,30E-08 | 2,455 |
| RNA binding motif (RNP1, RRM) protein 3 | RBM3 | 6,16E-04 | 2,446 |
| CD82 molecule | CD82 | 1,80E-06 | 2,445 |
| phosphoserine aminotransferase 1 | PSAT1 | 2,51E-05 | 2,444 |
| transmembrane protein 176A | TMEM176A | 1,55E-06 | 2,443 |
| lymphocyte antigen 6 complex, locus E | LY6E | 3,38E-07 | 2,442 |
| asparagine synthetase (glutamine-hydrolyzing) | ASNS | 4,50E-05 | 2,436 |
| actin beta | ACTB | 2,26E-06 | 2,434 |
| glycoprotein nmb | GPNMB | 2,20E-03 | 2,433 |
| phosphoglycerate dehydrogenase | PHGDH | 1,13E-05 | 2,432 |
| POU class 2 homeobox 2 | POU2F2 | 7,56E-05 | 2,43 |
| TNF receptor superfamily member 1B | TNFRSF1B | 4,93E-07 | 2,424 |
| interleukin 18 | IL18 | 1,43E-05 | 2,423 |
| CD68 molecule | CD68 | 4,20E-02 | 2,419 |
| aldehyde dehydrogenase 3 family member B1 | ALDH3B1 | 5,99E-06 | 2,409 |
| minichromosome maintenance complex component 3 | MCM3 | 7,58E-06 | 2,404 |
| T cell receptor beta, variable 5 | Trbv5 | 6,81E-06 | 2,404 |
| ankyrin repeat domain 1 | ANKRD1 | 2,61E-03 | 2,4 |
| CCAAT/enhancer binding protein alpha | CEBPA | 2,83E-05 | 2,399 |
| phosphoglycerate dehydrogenase | PHGDH | 2,00E-05 | 2,395 |
| PQ loop repeat containing 3 | PQLC3 | 3,22E-05 | 2,385 |
| transmembrane 6 superfamily member 1 | TM6SF1 | 5,98E-05 | 2,381 |
| transmembrane protein 176B | TMEM176B | 8,33E-07 | 2,38 |
| collapsin response mediator protein 1 | CRMP1 | 6,70E-07 | 2,378 |
| apolipoprotein L 7e | Apol7e (includes others) | 1,06E-04 | 2,375 |
| colony stimulating factor 1 receptor | CSF1R | 5,60E-05 | 2,371 |
| CNDP dipeptidase 2 (metallopeptidase M20 family) | CNDP2 | 2,55E-06 | 2,37 |
| pleckstrin homology domain containing O2 | PLEKHO2 | 1,01E-05 | 2,369 |
| cytochrome P450 family 4 subfamily V member 2 | CYP4V2 | 3,83E-07 | 2,368 |
| AT-rich interaction domain 5A | ARID5A | 6,70E-03 | 2,367 |
| interferon gamma inducible protein 16 | IFI16 | 5,03E-08 | 2,363 |
| ER degradation enhancing alpha-mannosidase like protein 1 | EDEM1 | 2,69E-05 | 2,362 |
| G protein-coupled receptor 174 | GPR174 | 5,31E-07 | 2,359 |
| T cell receptor associated transmembrane adaptor 1 | TRAT1 | 2,64E-05 | 2,359 |
| BCL2 antagonist/killer 1 | BAK1 | 3,89E-06 | 2,358 |
| IQ motif containing GTPase activating protein 1 | IQGAP1 | 3,80E-06 | 2,355 |
| TPX2, microtubule nucleation factor | TPX2 | 1,25E-05 | 2,354 |
| TXK tyrosine kinase | TXK | 1,51E-04 | 2,354 |
| calcium/calmodulin dependent protein kinase ID | CAMK1D | 1,31E-06 | 2,352 |
| FES proto-oncogene, tyrosine kinase | FES | 1,12E-06 | 2,351 |
| complement C3a receptor 1 | C3AR1 | 1,13E-06 | 2,347 |
| histocompatibility 2, T region locus 24 | H2-T24 | 5,46E-07 | 2,347 |
| interleukin 27 receptor subunit alpha | IL27RA | 2,12E-04 | 2,338 |
| nitric oxide synthase 2 | NOS2 | 5,37E-03 | 2,337 |
| IKAROS family zinc finger 1 | IKZF1 | 2,66E-06 | 2,336 |
| complement factor properdin | CFP | 3,20E-04 | 2,326 |
| dedicator of cytokinesis 11 | DOCK11 | 4,12E-06 | 2,323 |
| phosphoinositide-3-kinase adaptor protein 1 | PIK3AP1 | 1,11E-05 | 2,319 |
| actin related protein 2/3 complex subunit 1B | ARPC1B | 7,78E-06 | 2,316 |
| ATP binding cassette subfamily C member 3 | ABCC3 | 2,04E-05 | 2,315 |
| killer cell lectin-like receptor subfamily K, member 1 | Klrk1 | 2,95E-07 | 2,313 |
| lymphocyte-specific protein 1 | LSP1 | 5,99E-07 | 2,313 |
| LFNG O-fucosylpeptide 3-beta-N-acetylglucosaminyltransferase | LFNG | 7,95E-06 | 2,311 |
| family with sequence similarity 111 member A | FAM111A | 4,66E-04 | 2,309 |
| runt related transcription factor 1 | RUNX1 | 6,34E-03 | 2,309 |
| fibronectin type III domain containing 1 | Fndc1 | 5,42E-03 | 2,301 |
| solute carrier family 7 member 7 | SLC7A7 | 1,79E-06 | 2,298 |
| olfactomedin 1 | OLFM1 | 1,15E-05 | 2,295 |
| CD83 molecule | CD83 | 3,95E-02 | 2,293 |
| G protein-coupled receptor 84 | GPR84 | 1,88E-03 | 2,281 |
| NPC intracellular cholesterol transporter 2 | NPC2 | 5,90E-06 | 2,281 |
| transient receptor potential cation channel subfamily V member 2 | TRPV2 | 1,66E-06 | 2,278 |
| PDZ binding kinase | PBK | 5,42E-07 | 2,277 |
| ribonucleotide reductase regulatory subunit M2 | RRM2 | 4,71E-05 | 2,276 |
| tropomyosin 3 | TPM3 | 1,92E-06 | 2,275 |
| cyclin F | CCNF | 2,15E-06 | 2,274 |
| phosphatidylinositol-4,5-bisphosphate 3-kinase catalytic subunit gamma | PIK3CG | 1,08E-06 | 2,274 |
| potassium voltage-gated channel subfamily J member 10 | KCNJ10 | 1,03E-05 | 2,273 |
| ABI family member 3 | ABI3 | 5,53E-04 | 2,271 |
| CDP-diacylglycerol synthase 1 | CDS1 | 3,89E-07 | 2,27 |
| apolipoprotein E | APOE | 3,22E-07 | 2,264 |
| caspase 12 | Casp12 | 7,75E-06 | 2,258 |
| schlafen 1 | Slfn1 | 3,85E-05 | 2,257 |
| profilin 1 | PFN1 | 3,00E-05 | 2,254 |
| receptor interacting serine/threonine kinase 1 | RIPK1 | 1,32E-07 | 2,253 |
| RAB8B, member RAS oncogene family | RAB8B | 2,55E-06 | 2,25 |
| extra spindle pole bodies like 1, separase | ESPL1 | 1,22E-07 | 2,248 |
| shugoshin 1 | SGO1 | 1,18E-05 | 2,248 |
| family with sequence similarity 46 member C | FAM46C | 8,84E-05 | 2,247 |
| TBC1 domain family member 9 | TBC1D9 | 3,46E-07 | 2,247 |
| ZFP36 ring finger protein like 2 | ZFP36L2 | 3,01E-05 | 2,247 |
| zinc finger CCCH-type containing, antiviral 1 | ZC3HAV1 | 2,23E-04 | 2,243 |
| thymosin, beta 4, X chromosome | Tmsb4x (includes others) | 8,57E-06 | 2,242 |
| SHC binding and spindle associated 1 | SHCBP1 | 9,47E-05 | 2,241 |
| interleukin 7 receptor | IL7R | 4,09E-07 | 2,233 |
| membrane spanning 4-domains A8 | MS4A8 | 1,00E-02 | 2,233 |
| histone cluster 2 H2B family member f | HIST2H2BF | 5,70E-04 | 2,232 |
| cDNA sequence BC028789 | BC028789 | 2,68E-06 | 2,227 |
| major histocompatibility complex, class II, DO beta | HLA-DOB | 1,64E-06 | 2,226 |
| BTG anti-proliferation factor 1 | BTG1 | 7,47E-05 | 2,221 |
| major vault protein | MVP | 2,97E-05 | 2,22 |
| CKLF like MARVEL transmembrane domain containing 7 | CMTM7 | 1,15E-06 | 2,218 |
| interleukin 6 | IL6 | 1,23E-02 | 2,216 |
| GTPase, IMAP family member 9 | Gimap9 | 3,41E-04 | 2,214 |
| class II major histocompatibility complex transactivator | CIITA | 1,50E-03 | 2,213 |
| RAS p21 protein activator 4 | RASA4 | 3,47E-06 | 2,213 |
| paired immunoglobin-like type 2 receptor beta | PILRB | 1,57E-04 | 2,212 |
| keratin 85 | Krt85 | 1,48E-03 | 2,208 |
| potassium two pore domain channel subfamily K member 5 | KCNK5 | 2,53E-05 | 2,207 |
| serine peptidase inhibitor, Kunitz type 1 | SPINT1 | 9,49E-07 | 2,205 |
| MARCKS like 1 | MARCKSL1 | 7,93E-04 | 2,204 |
| lymphocyte antigen 6 complex, locus G5B | LY6G5B | 4,62E-05 | 2,201 |
| piggyBac transposable element derived 5 | PGBD5 | 1,68E-05 | 2,201 |
| adenosine deaminase | ADA | 1,07E-04 | 2,2 |
| regulator of G-protein signaling 19 | RGS19 | 1,42E-04 | 2,197 |
| ubiquitin associated and SH3 domain containing A | UBASH3A | 8,77E-08 | 2,192 |
| phosphatase and actin regulator 1 | PHACTR1 | 5,12E-06 | 2,19 |
| cytokine dependent hematopoietic cell linker | CLNK | 4,54E-06 | 2,187 |
| CD101 molecule | CD101 | 7,62E-05 | 2,175 |
| chromosome 14 open reading frame 80 | C14orf80 | 2,55E-06 | 2,174 |
| high mobility group AT-hook 2, pseudogene 1 | Hmga2-ps1 | 1,18E-04 | 2,171 |
| serine carboxypeptidase 1 | SCPEP1 | 3,45E-06 | 2,168 |
| B and T lymphocyte associated | BTLA | 3,37E-06 | 2,164 |
| ring finger protein 31 | RNF31 | 1,94E-06 | 2,162 |
| SH2 domain containing 1B | SH2D1B | 1,09E-05 | 2,16 |
| GRB2-related adaptor protein 2 | GRAP2 | 5,90E-05 | 2,158 |
| interleukin 17 receptor A | IL17RA | 1,60E-03 | 2,156 |
| component of Sp100-rs | Csprs (includes others) | 1,42E-05 | 2,155 |
| interferon induced transmembrane protein 3 | IFITM3 | 5,43E-06 | 2,155 |
| RAD51 recombinase | RAD51 | 3,16E-06 | 2,154 |
| caspase 8 | CASP8 | 3,15E-07 | 2,153 |
| B-cell linker | BLNK | 5,97E-03 | 2,151 |
| cell division cycle 20 | CDC20 | 1,25E-04 | 2,147 |
| immediate early response 3 | IER3 | 1,50E-04 | 2,147 |
| WNT1 inducible signaling pathway protein 2 | WISP2 | 1,88E-03 | 2,147 |
| TRAF3 interacting protein 3 | TRAF3IP3 | 7,95E-06 | 2,145 |
| acyl-CoA synthetase bubblegum family member 1 | ACSBG1 | 1,19E-03 | 2,144 |
| tubulin beta 2B class IIb | TUBB2B | 1,44E-03 | 2,143 |
| F-box protein 5 | FBXO5 | 9,09E-06 | 2,136 |
| thyroid hormone receptor interactor 13 | TRIP13 | 2,05E-04 | 2,135 |
| serpin family B member 9 | SERPINB9 | 2,41E-05 | 2,134 |
| spindle and kinetochore associated complex subunit 1 | SKA1 | 1,60E-04 | 2,133 |
| sushi repeat containing protein, X-linked | SRPX | 1,80E-04 | 2,13 |
| transforming acidic coiled-coil containing protein 3 | TACC3 | 2,12E-04 | 2,129 |
| secreted and transmembrane 1 | SECTM1 | 1,59E-06 | 2,124 |
| interleukin 6 receptor | IL6R | 1,23E-04 | 2,123 |
| SLC9A3 regulator 1 | SLC9A3R1 | 5,20E-05 | 2,123 |
| Rho GTPase activating protein 22 | ARHGAP22 | 1,55E-03 | 2,122 |
| C-X3-C motif chemokine ligand 1 | CX3CL1 | 4,18E-04 | 2,12 |
| uncoupling protein 2 | UCP2 | 4,59E-03 | 2,12 |
| torsin family 3 member A | TOR3A | 4,22E-06 | 2,117 |
| toll-like receptor 11 | Tlr11 | 9,59E-05 | 2,113 |
| Cas scaffolding protein family member 4 | CASS4 | 6,60E-06 | 2,11 |
| aurora kinase A | AURKA | 4,60E-04 | 2,109 |
| toll like receptor 6 | TLR6 | 4,93E-05 | 2,106 |
| inhibitor of kappa light polypeptide gene enhancer in B-cells, kinase epsilon | IKBKE | 7,49E-06 | 2,104 |
| DEF6, guanine nucleotide exchange factor | DEF6 | 1,41E-05 | 2,103 |
| RAB5C, member RAS oncogene family | RAB5C | 2,00E-04 | 2,102 |
| interferon stimulated exonuclease gene 20 | ISG20 | 4,31E-04 | 2,101 |
| CD37 molecule | CD37 | 7,75E-05 | 2,097 |
| myeloid differentiation primary response 88 | MYD88 | 1,02E-05 | 2,097 |
| GIMAP1-GIMAP5 readthrough | GIMAP1-GIMAP5 | 7,76E-04 | 2,096 |
| RAB3A interacting protein like 1 | RAB3IL1 | 3,63E-04 | 2,094 |
| calcium/calmodulin dependent protein kinase IG | CAMK1G | 4,71E-05 | 2,093 |
| germ cell associated 2, haspin | GSG2 | 5,40E-07 | 2,085 |
| syndecan 3 | SDC3 | 4,90E-06 | 2,083 |
| sterol O-acyltransferase 2 | SOAT2 | 4,77E-06 | 2,082 |
| pyrophosphatase (inorganic) 1 | PPA1 | 1,11E-04 | 2,072 |
| B-box and SPRY domain containing | BSPRY | 6,11E-06 | 2,071 |
| ADP ribosylation factor 3 | ARF3 | 4,72E-05 | 2,07 |
| lipin 2 | LPIN2 | 8,00E-05 | 2,07 |
| polypeptide N-acetylgalactosaminyltransferase 3 | GALNT3 | 5,41E-06 | 2,069 |
| docking protein 3 | DOK3 | 5,47E-05 | 2,068 |
| sortilin related receptor 1 | SORL1 | 1,66E-05 | 2,068 |
| contactin 5 | CNTN5 | 4,67E-04 | 2,066 |
| polypeptide N-acetylgalactosaminyltransferase 12 | GALNT12 | 3,62E-07 | 2,066 |
| non-SMC condensin I complex subunit H | NCAPH | 2,78E-03 | 2,063 |
| prostaglandin-endoperoxide synthase 2 | PTGS2 | 4,78E-04 | 2,063 |
| NLR family, apoptosis inhibitory protein 1 | Naip1 (includes others) | 7,30E-05 | 2,058 |
| structural maintenance of chromosomes 2 | SMC2 | 2,05E-04 | 2,058 |
| TNF receptor superfamily member 11b | TNFRSF11B | 1,69E-04 | 2,058 |
| lipopolysaccharide induced TNF factor | LITAF | 4,31E-05 | 2,056 |
| janus kinase and microtubule interacting protein 1 | JAKMIP1 | 2,08E-05 | 2,054 |
| transgelin 2 | TAGLN2 | 1,37E-04 | 2,052 |
| phosphofructokinase, platelet | PFKP | 3,27E-07 | 2,05 |
| carcinoembryonic antigen related cell adhesion molecule 16 | CEACAM16 | 8,30E-06 | 2,042 |
| ras homolog family member G | RHOG | 3,36E-06 | 2,042 |
| myelin associated glycoprotein | MAG | 1,55E-06 | 2,04 |
| family with sequence similarity 49 member A | FAM49A | 3,91E-05 | 2,035 |
| phosphotyrosine interaction domain containing 1 | PID1 | 5,96E-06 | 2,033 |
| Mov10 RISC complex RNA helicase | MOV10 | 1,70E-04 | 2,031 |
| plasminogen activator, urokinase receptor | PLAUR | 2,13E-03 | 2,03 |
| immediate early response 5 | IER5 | 1,65E-03 | 2,028 |
| protein tyrosine phosphatase, receptor type E | PTPRE | 2,77E-04 | 2,027 |
| small G protein signaling modulator 2 | SGSM2 | 1,78E-04 | 2,027 |
| proline rich protein HaeIII subfamily 1 | Prh1/Prp2 | 3,78E-04 | 2,019 |
| zinc finger NFX1-type containing 1 | ZNFX1 | 1,74E-06 | 2,018 |
| 5'-aminolevulinate synthase 2 | ALAS2 | 9,01E-03 | 2,017 |
| membrane-spanning 4-domains, subfamily A, member 6B | Ms4a6b | 1,61E-04 | 2,017 |
| transketolase | TKT | 9,05E-06 | 2,014 |
| C-X-C motif chemokine ligand 2 | CXCL2 | 3,68E-03 | 2,01 |
| CD200 receptor 1 | CD200R1 | 1,38E-05 | 2,008 |
| cofilin 1 | CFL1 | 7,19E-04 | 2,008 |
| GATA binding protein 3 | GATA3 | 1,20E-04 | 2,007 |
| N-acylsphingosine amidohydrolase 1 | ASAH1 | 8,70E-05 | 2,007 |
| CREB binding protein | CREBBP | 1,46E-05 | 2,005 |
| zinc finger CCCH-type containing, antiviral 1 | ZC3HAV1 | 1,31E-06 | 2,005 |
| interferon gamma inducible protein 16 | IFI16 | 5,42E-03 | 2,003 |
| claudin 5 | CLDN5 | 7,63E-04 | -2,001 |
| glutathione S-transferase mu 2 | GSTM2 | 5,04E-04 | -2,001 |
| pleckstrin homology domain containing B1 | PLEKHB1 | 1,07E-04 | -2,001 |
| RIKEN cDNA B330016D10 gene | B330016D10Rik | 1,00E-05 | -2,002 |
| chromosome 15 open reading frame 52 | C15orf52 | 1,56E-03 | -2,003 |
| cholinergic receptor nicotinic alpha 2 subunit | CHRNA2 | 8,83E-03 | -2,004 |
| ependymin related 1 | EPDR1 | 1,16E-05 | -2,006 |
| DnaJ heat shock protein family (Hsp40) member C28 | DNAJC28 | 2,36E-03 | -2,007 |
| yippee like 2 | YPEL2 | 1,36E-04 | -2,008 |
| proenkephalin | PENK | 4,51E-04 | -2,013 |
| glutathione S-transferase, mu 6 | Gstm6 | 1,56E-04 | -2,016 |
| ficolin A | Fcna | 1,25E-03 | -2,017 |
| nitrilase 1 | NIT1 | 2,17E-02 | -2,017 |
| Y-box binding protein 2 | YBX2 | 1,74E-04 | -2,017 |
| nephrocan | Nepn | 1,90E-02 | -2,025 |
| granzyme M | GZMM | 1,35E-04 | -2,031 |
| family with sequence similarity 222 member A | FAM222A | 2,94E-03 | -2,033 |
| protein tyrosine phosphatase, receptor type R | PTPRR | 8,17E-05 | -2,035 |
| serine/arginine repetitive matrix 4 | SRRM4 | 1,85E-02 | -2,037 |
| prostaglandin D2 synthase | PTGDS | 1,65E-03 | -2,038 |
| solute carrier family 36 member 2 | SLC36A2 | 6,11E-06 | -2,04 |
| armadillo repeat containing 2 | ARMC2 | 1,06E-03 | -2,046 |
| ADP-ribosyltransferase 5 | ART5 | 4,15E-03 | -2,047 |
| ATP binding cassette subfamily B member 1 | ABCB1 | 2,81E-05 | -2,047 |
| family with sequence similarity 131 member A | FAM131A | 3,22E-04 | -2,048 |
| syntaxin binding protein 4 | STXBP4 | 6,84E-03 | -2,051 |
| GTP cyclohydrolase I feedback regulator | GCHFR | 7,87E-04 | -2,056 |
| activin A receptor type 2B | ACVR2B | 1,44E-03 | -2,059 |
| very low density lipoprotein receptor | VLDLR | 2,66E-06 | -2,061 |
| RAB4A, member RAS oncogene family | RAB4A | 1,42E-02 | -2,063 |
| hydroxyprostaglandin dehydrogenase 15-(NAD) | HPGD | 3,72E-04 | -2,069 |
| talin 2 | TLN2 | 9,08E-06 | -2,073 |
| myosin light chain 1 | MYL1 | 8,02E-04 | -2,076 |
| tumor protein D52-like 1 | TPD52L1 | 2,24E-04 | -2,078 |
| stathmin 2 | STMN2 | 7,09E-05 | -2,079 |
| glutathione S-transferase alpha 5 | GSTA5 | 5,77E-03 | -2,085 |
| perilipin 4 | PLIN4 | 1,20E-02 | -2,087 |
| zinc finger protein pseudogene | BC025920 | 2,47E-04 | -2,096 |
| leukotriene C4 synthase | LTC4S | 1,91E-05 | -2,105 |
| EPH receptor B1 | EPHB1 | 2,92E-05 | -2,107 |
| heat shock factor binding protein 1 like 1 | HSBP1L1 | 3,49E-05 | -2,107 |
| retinol saturase | RETSAT | 1,06E-04 | -2,107 |
| dihydrolipoamide branched chain transacylase E2 | DBT | 7,79E-05 | -2,109 |
| EMI domain containing 1 | EMID1 | 5,33E-04 | -2,125 |
| alcohol dehydrogenase 1C (class I), gamma polypeptide | ADH1C | 1,57E-02 | -2,13 |
| endonuclease/exonuclease/phosphatase family domain containing 1 | EEPD1 | 1,08E-05 | -2,13 |
| Rho GTPase activating protein 20 | ARHGAP20 | 5,61E-03 | -2,13 |
| fibronectin type III domain containing 4 | FNDC4 | 1,40E-04 | -2,131 |
| malonyl-CoA decarboxylase | MLYCD | 3,35E-05 | -2,131 |
| RIKEN cDNA 1700042O10 gene | 1700042O10Rik | 1,36E-04 | -2,133 |
| DNA polymerase theta | POLQ | 8,49E-04 | -2,135 |
| amylase, alpha 2A (pancreatic) | AMY2A | 3,29E-03 | -2,136 |
| trophinin | TRO | 4,89E-05 | -2,14 |
| syntaxin binding protein 6 | STXBP6 | 8,32E-06 | -2,141 |
| RIKEN cDNA 9330102E08 gene | 9330102E08Rik | 6,60E-06 | -2,143 |
| phospholipase A2 group V | PLA2G5 | 6,67E-05 | -2,146 |
| zinc finger protein 467 | ZNF467 | 1,42E-07 | -2,147 |
| neurotrophin 3 | NTF3 | 8,04E-05 | -2,15 |
| 4-aminobutyrate aminotransferase | ABAT | 3,76E-05 | -2,156 |
| oxoglutarate dehydrogenase-like | OGDHL | 3,32E-03 | -2,16 |
| MAGI family member, X-linked | MAGIX | 6,68E-04 | -2,171 |
| carbohydrate sulfotransferase 1 | CHST1 | 1,86E-05 | -2,174 |
| mal, T-cell differentiation protein | MAL | 5,31E-06 | -2,174 |
| arachidonate 5-lipoxygenase | ALOX5 | 1,56E-03 | -2,181 |
| polo like kinase 5 | PLK5 | 3,32E-05 | -2,183 |
| RIKEN cDNA 2310001K24 gene | 2310001K24Rik | 8,94E-04 | -2,183 |
| secreted frizzled related protein 5 | SFRP5 | 7,61E-04 | -2,183 |
| solute carrier family 7 member 4 | SLC7A4 | 4,61E-04 | -2,184 |
| complement factor D | CFD | 1,91E-02 | -2,188 |
| KIAA1456 | KIAA1456 | 3,38E-07 | -2,188 |
| ST6 N-acetylgalactosaminide alpha-2,6-sialyltransferase 2 | ST6GALNAC2 | 4,53E-06 | -2,189 |
| chromosome 14 open reading frame 37 | C14orf37 | 1,30E-05 | -2,197 |
| fibrinogen like 1 | FGL1 | 1,77E-05 | -2,197 |
| insulin receptor substrate 1 | IRS1 | 1,30E-05 | -2,203 |
| carnitine palmitoyltransferase 2 | CPT2 | 1,91E-06 | -2,211 |
| frequently rearranged in advanced T-cell lymphomas 1 | FRAT1 | 4,20E-03 | -2,213 |
| von Willebrand factor A domain containing 3A | VWA3A | 1,05E-04 | -2,217 |
| predicted gene 1673 | Gm1673 | 1,70E-04 | -2,222 |
| perilipin 5 | PLIN5 | 1,37E-06 | -2,224 |
| cartilage oligomeric matrix protein | COMP | 4,08E-04 | -2,229 |
| kelch like family member 34 | KLHL34 | 5,38E-06 | -2,229 |
| protein tyrosine phosphatase, receptor type B | PTPRB | 1,31E-04 | -2,237 |
| methionine sulfoxide reductase B2 | MSRB2 | 2,56E-04 | -2,246 |
| pyruvate dehyrogenase phosphatase catalytic subunit 2 | PDP2 | 2,43E-05 | -2,251 |
| glucokinase | GCK | 6,42E-03 | -2,253 |
| predicted gene 10638 | Gm10638 | 5,77E-04 | -2,255 |
| erythropoietin receptor | EPOR | 8,27E-07 | -2,258 |
| C-X-C motif chemokine ligand 14 | CXCL14 | 3,55E-04 | -2,263 |
| cytokine like 1 | CYTL1 | 4,49E-05 | -2,27 |
| distal-less homeobox 3 | DLX3 | 6,51E-04 | -2,273 |
| synaptopodin 2 | SYNPO2 | 6,87E-07 | -2,284 |
| solute carrier family 26 member 6 | SLC26A6 | 6,05E-07 | -2,286 |
| MLX interacting protein like | MLXIPL | 3,37E-06 | -2,31 |
| transforming acidic coiled-coil containing protein 2 | TACC2 | 7,62E-03 | -2,312 |
| catenin alpha like 1 | CTNNAL1 | 4,65E-03 | -2,313 |
| ephrin B3 | EFNB3 | 5,87E-03 | -2,337 |
| fin bud initiation factor homolog (zebrafish) | FIBIN | 1,28E-04 | -2,34 |
| dopamine beta-hydroxylase | DBH | 1,21E-03 | -2,343 |
| aquaporin 4 | AQP4 | 1,71E-04 | -2,379 |
| ADAMTS like 2 | ADAMTSL2 | 8,04E-05 | -2,396 |
| calreticulin 3 | CALR3 | 3,28E-04 | -2,4 |
| C-type lectin domain family 18 member B | CLEC18B | 2,72E-03 | -2,401 |
| anoctamin 10 | ANO10 | 6,33E-05 | -2,406 |
| leucine rich repeat and Ig domain containing 3 | LINGO3 | 1,29E-04 | -2,413 |
| synaptopodin | SYNPO | 1,65E-05 | -2,422 |
| transmembrane protein 82 | TMEM82 | 9,02E-04 | -2,431 |
| brain derived neurotrophic factor | BDNF | 5,15E-05 | -2,436 |
| TIMP metallopeptidase inhibitor 4 | TIMP4 | 3,41E-03 | -2,489 |
| feline leukemia virus subgroup C cellular receptor family member 2 | FLVCR2 | 2,29E-03 | -2,492 |
| sodium voltage-gated channel beta subunit 4 | SCN4B | 5,25E-03 | -2,492 |
| adenosine A1 receptor | ADORA1 | 8,68E-04 | -2,505 |
| acyl-CoA thioesterase 1 | Acot1 | 4,95E-03 | -2,511 |
| DDB1 and CUL4 associated factor 12-like 1 | Dcaf12l1 | 1,86E-06 | -2,543 |
| enoyl-CoA hydratase and 3-hydroxyacyl CoA dehydrogenase | EHHADH | 2,39E-03 | -2,585 |
| meiosis/spermiogenesis associated 1 | MEIG1 | 7,81E-05 | -2,627 |
| adipogenin | Adig | 1,21E-07 | -2,631 |
| CD163 molecule | CD163 | 6,70E-07 | -2,654 |
| acyl-CoA thioesterase 5 | Acot5 | 1,48E-03 | -2,685 |
| DNA polymerase N | Poln | 2,48E-03 | -2,686 |
| lymphatic vessel endothelial hyaluronan receptor 1 | LYVE1 | 7,14E-04 | -2,686 |
| solute carrier family 22 member 1 | SLC22A1 | 2,27E-04 | -2,691 |
| coagulation factor XIII A chain | F13A1 | 3,73E-05 | -2,692 |
| cDNA sequence BC024137 | BC024137 | 3,38E-07 | -2,776 |
| myotilin | MYOT | 1,05E-04 | -2,883 |
| immunoglobulin superfamily member 1 | IGSF1 | 1,01E-04 | -2,904 |
| amphiphysin | AMPH | 2,63E-07 | -2,957 |
| cyclin-dependent kinase inhibitor 1C (P57) | Cdkn1c | 1,40E-07 | -2,959 |
| stearoyl-coenzyme A desaturase 4 | Scd4 | 1,12E-04 | -2,961 |
| H19, imprinted maternally expressed transcript (non-protein coding) | H19 | 4,46E-06 | -2,989 |
| actin, alpha 1, skeletal muscle | ACTA1 | 2,69E-03 | -2,996 |
| predicted gene 10635 | Gm10635 | 2,05E-05 | -3,01 |
| cold shock domain containing C2 | CSDC2 | 1,84E-04 | -3,046 |
| HR, lysine demethylase and nuclear receptor corepressor | HR | 1,22E-06 | -3,11 |
| potassium voltage-gated channel modifier subfamily V member 2 | KCNV2 | 4,47E-07 | -3,147 |
| whirlin | WHRN | 7,07E-07 | -3,162 |
| predicted gene 4956 | Gm4956 | 2,16E-06 | -3,176 |
| potassium voltage-gated channel subfamily A member 1 | KCNA1 | 3,99E-06 | -3,212 |
| aquaporin 1 (Colton blood group) | AQP1 | 8,27E-07 | -3,234 |
| phosphorylase kinase catalytic subunit gamma 1 | PHKG1 | 2,41E-05 | -3,28 |
| dopachrome tautomerase | DCT | 6,16E-04 | -3,291 |
| RAS like family 10 member A | RASL10A | 1,54E-06 | -3,418 |
| solute carrier family 40 member 1 | SLC40A1 | 8,16E-06 | -3,582 |
| myosin binding protein C, fast type | MYBPC2 | 2,75E-08 | -3,637 |
| C-C motif chemokine ligand 24 | CCL24 | 5,65E-07 | -3,656 |
| 6-phosphofructo-2-kinase/fructose-2,6-biphosphatase 1 | PFKFB1 | 1,34E-05 | -3,7 |
| fumarylacetoacetate hydrolase | FAH | 1,00E-04 | -3,725 |
| 3-hydroxy-3-methylglutaryl-CoA synthase 2 | HMGCS2 | 5,44E-03 | -3,794 |
| Fc receptor-like S, scavenger receptor | Fcrls | 8,28E-07 | -3,879 |
| aryl hydrocarbon receptor nuclear translocator like | ARNTL | 3,62E-07 | -4,047 |
| pyruvate dehydrogenase kinase 4 | PDK4 | 7,93E-03 | -4,368 |
| aldolase, fructose-bisphosphate B | ALDOB | 1,11E-04 | -4,781 |
| carbonyl reductase 2 | Cbr2 | 1,66E-09 | -5,012 |
| angiopoietin like 4 | ANGPTL4 | 8,18E-04 | -5,25 |
| RIKEN cDNA 2210407C18 gene | 2210407C18Rik | 6,42E-07 | -5,774 |
| resistin like alpha | Retnla | 3,35E-06 | -8,716 |

**Supplemental table 4 :** Description of the DEGs shared or not shared between the various time points

| **Exclusive 15DPI up-regulated genes** |
| --- |
| Ifi27l2a/Ifi27l2b, IL4I1, SIGLEC1, ZBTB32, OAS2, IDO1, MMP25, 9530082P21Rik, MCM10, FANCA, Prm1, TLR3, PARP3, VWA5A, DEPDC1B, OGFR, HELZ2, HSPA1A/HSPA1B, MITD1, INPP1, DBNL, ERAP1, NEFH, ATP10A, NOD1, PRIM1, ECE2, LIG1, PRKCQ, NGFR, EXO1, TREX1, KNTC1, ZBTB5, ITPKA, BAZ1A, DAXX, RELB, PKMYT1, OSMR, ADAMTS4, CFLAR, BARD1, TIMD4, MELK, CCND1, ANGPT2, NUF2, STAT3, POMP, TRAFD1, CNN3, JUNB, TK1, Trbv2, TUBB, ACTG1, LRRC4, RCN1, IKZF4, PARP11, CDCA7, H2AFJ, AI662270, CLIC4, EIF4E3, MXD3, HIST2H2AA3/HIST2H2AA4, ARHGAP8/PRR5-ARHGAP8, CTLA4, CSF1, TUBA1B, CKS1B, DCTN6, CD47, LYN, GINS2, Tpm4, ALOX15, PLEKHA4, CKAP2L, SIDT1, EIF1AX, FEN1, HIST1H3C, KIF11, ACSM5, STXBP1, PSMA5, GIMAP8, ZWILCH, CA13, P2RY13, GADD45G, Gdap10, INPP5B, IL10, IPCEF1 |
|  |
| **Exclusive 15DPI down-regulated genes** |
| TF, IGFBP6, PLEKHG5, KCNH2, SNORA74A, PTGS1, 4631405J19Rik, TCEAL7, TSPAN18, Pcp4l1, ACE2, SCARA5, KCNK3, MYRIP, RGS5, SOX4, ARL4D, FXYD6, RASD1, GRM1, FAM181B, BMP6, SOX17, SGK1, SEPT8, RNASE10, STAB2, GUCY1A3, Abca8a, KAZALD1, IL17D, Akr1c19, ATP1B2, GPR22, RBP7, P2RX3, SPRY1, IGFBP3, APOE, SMOC2, GNMT, CMTM8, CST6, LRRC4B, Slc26a10, CAMK2N1, DENND5B, WNT5B, NANOG, NLGN3, 2310002L09Rik, FBLN5, ITGA11, DUSP1, PTPRU, Scgb1c1, 6230415J03Rik, MAP6D1, C10orf10, CYP26B1, ZNF488, DDIT4 |
|  |
| **Exclusive 30DPI up-regulated genes** |
| SERPINA3, CRLF1, SLPI, RNF149, ARRDC4, Nppb, LCN2, SAT1, PLA1A, ATP1A3, CLCA1, CSTB, POSTN, 2900076A13Rik, FKBP1B, ANLN, CTSH, FAM46B, LTBP2, MERTK, Ccl6, UGT1A7 , FAM64A, CXCR4, WDR92, Stfa2/Stfa2l1, MAN2B1, CLCA2, ORAI2, GM2A, MMP14, HSD11B1, FN1, PTPRJ, GPX1, Ccl9, CYP1B1, ARHGAP25, ECM1, DPEP1, CPXM1, TSPAN33, NPPA, CD5L, TMEM128, ASPRV1, FTL, NEK6, 1500011B03Rik, MAFB, ANXA1, MSLN, PRCP, MFAP5, SLC7A8, KLHL6, CRTAM, SGPL1, CILP, ADAP2, SH2B2, TMPRSS4, RENBP, Gm6548, MAPK13, 9330199G10Rik, PAMR1, TUBA1C, SDC4, MAN2A1, TMEM119, COL3A1, UAP1L1, CD38, ZYX, SLC25A45, CPNE2, CHPF2, GPR141, FAM189B, MFAP4, LOX, SLC41A2, GBA, KRT18, EPSTI1, COL1A1, 6330566A10Rik, SERINC2, NT5C, EEF1A1, CRCT1, IL33, VASP, WFDC8, LSR, COL1A2, MFSD1, CD14, FRMD8, STX3, FLT3, CD302, TNFRSF12A, ROPN1L, BIRC3, PTPN1, CA9, TMEM45A, PLAT, C4orf32, PLK3, BMP2K, LRCH4, ARRDC1, NCAPG2, SLC39A11, PTGER4, THBS3, ADORA3, TPD52, VDR, KCNA3, EIF1AD, CKS2, ANXA8/ANXA8L1, MMP19, LYL1, GLTP, PDK3, TARM1, CORO7/CORO7-PAM16, Akr1c12/Akr1c13, IL1RN, ANKRD44, ALCAM, GNGT2, Ighg2c, YPEL5, BGN, NOTCH2, TTYH3, Xlr4a , Ifitm10, ARF6, PABPC1, TMEM37, SPP1, 2610524H06Rik, CKAP2, NLRP1, 2010016I18Rik, SDCBP, GRIN2C, TSPAN4, ITGB3, ARMC7, FAM129A, ARHGAP17, PGLYRP1, CAMK4, HEXB, ARPC4, RARRES2, ATP6AP2, AUH, CMTM3, AGPAT4, MKRN1, LOC728392, NUAK2, ACER3, LAD1, EYA2, Gm4907, SNCA, SRI, PDPN, TEP1, PPT1, 4833422C13Rik, TNS3, SLC43A2, SLC4A1, COL14A1, PIP4K2A |
|  |
| **Exclusive 30DPI down-regulated genes** |
| 30DPI, CMBL, PRDM16, ACAA2, FASTK, A430046D13Rik, DNMT3A, SLC22A5, VARS2, TMEM201, ZNF606, HOMER2, IVD, MACROD2, ZSCAN22, AARS2, SRP54, GPT2, BRWD1, GSG1L, TTC21B, TESC, ACSL1, SORD, Gprasp2, NDUFA1, PF4, REEP6, Lgals6, SLC39A3, MECR, COQ9, HEATR5B, MYLK4, SCRN2, REEP5, RNF207, UBE2N, HADHB, SLC24A3, NHLRC1, NRN1, RGS4, ABRA, ADHFE1, CNBP, Tha1, WNT9A, CHCHD7, RNF150, C8orf4, KLHL30, SLC5A6, ZBTB20, CRYBB1, PRSS36, HIBCH, ABHD14A, MYO18B, RHOBTB3, GFRA3, MCEE, AOX1, GPAM, LYNX1, ABCB6, C7orf49, BC030870, FAHD2B, RHOT2, PDZRN4, RIC8B, SVIP, KCND3, DIP2C, PRLR, DUS4L, CYB5D2, KY, CCDC28A, OBSCN, TMEM25, DHODH, BPHL, OSBP2, RAB3A, CLASP1, Mrgprh, POLG, TMEM143, RGL3, EXOC8, GPRASP1, SUOX, TUFM, HADH, PDE4A, MCCC1, TTC30B, LACE1, INTU, PDP1, C10orf107, ASB5, CUX2, ALPK2, TMEM41B, RDM1, KLF12, SNED1, Hsdl2, TFB1M, E330011O21Rik, Gm826, ACAD11, RPL3L, ETFDH, Airn, CENPV, HSPD1, PKIA, ALDH4A1, L2HGDH, ECH1, ALDH6A1, ZNF536, APBB1, CKM, PLEKHH1, ANGPTL3, CAPRIN2, HAND2, DHDH, SLC25A33, CHAC1, NCBP2-AS2, KCNA7, MDGA1, CCNH, PLP1, DDC, ECHDC3, LRRC39, ABCC9, Sh3bgr, FN3K, AGL, TMEM132A, LGALS4, 1110019D14Rik, TMEM65, Ppbp, KLHL31, CDK5, FNDC5, PKD2L2, MUT, FBP2, BCL2L13, DCAF4, 1810014B01Rik, RGMA, DLAT, A630039O03Rik, Mfsd4b3, TSC22D1, HADHA, B3GALT2, GRB14, C19orf68, DCUN1D2, A930018M24Rik, ACOT2, Lypd2, CYB5RL, AI426330, LRTM1, LDHD, SPEF1, OXCT1, NOTCH1, FKBP4, IFT81, PEX6, FGF9, POMGNT2, KLHL38, PAQR9, TMC7, FAM179A, RMND1, CPOX, NME4, TBC1D4, HIST1H2BJ, TNNT1, TRDN, JOSD2, CHADL, C1QTNF4, RAB6B, PES1, C4orf33, PEX11A, Gm14290, HRASLS, TRPM1, CRYBA4, UROS, 2700097O09Rik, GSTK1, GPAT3, Hrc, GCDH, PCP4, PXMP2, CDNF, BC062258, C14orf159, CARS2, ECM2, COX6B2, UNC45B, IGDCC4, MCCC2, EPHA4, SLC25A26, TMEM177, MMAA, KLHDC1, Rbm33, MKKS, PPP1R9A, MAPT, DCAKD, BOLA1, SLC25A42, ZNF23, REEP1, BCHE, ITGB6, PIGR, DDO, DCC, P2RY1, Rhox8 |
|  |
| **Exclusive 45DPI up-regulated genes** |
| XCL1, DBP, Tcrg-C1, Klrk1, CD83, HLA-DOB, CIITA, CLNK, IER3, Tlr11, CAMK1G, GALNT3, IER5, ALAS2 |
|  |
| **Exclusive 45DPI down-regulated genes** |
| 45DPI, CLDN5, B330016D10Rik, YPEL2, Gstm6, Fcna, FAM222A, PTGDS, ART5, RAB4A, GSTA5, PLIN4, LTC4S, EMID1, 1700042O10Rik, TRO, PLA2G5, ZNF467, 2310001K24Rik, CFD, PLIN5, Gm10638, AQP4, ADORA1, LYVE1, F13A1, Scd4, ARNTL |
|  |
| **Common 30DPI and 15DPI up-regulated genes** |
| LTB4R, PTX3, ARG1, HIST1H2AA, SELL, HIST1H2AH, FAM167B, ASF1B, CCNE1, IL4R, HIST1H2AJ, INCENP, HIST2H2AC, SBNO2, IL18RAP, LGALS9B, PYCARD, SLAMF1, SPAG5, KIFC1, CDK1, LEF1, Gm8096, Ifitm1, Tyms-ps, RCC2, CD247, MNS1, KIF20A, SMPDL3B, B4GALT5, BUB1B, CENPH, SYCE2, CENPK, S100A6, ANXA2, CDCA2, CHAF1B, CDC25B, TNFAIP6, APOD, ARL6IP1, GRN, THEMIS, GNA13, C17orf62, SELP, S1PR4, GLRX, A630081D01Rik, MCTP2, PIM1, NUPR1, ARPC5, PRELID1, SERP1, CKLF, FLNA, Tnfsf9, MAF, AI413582, ANXA4, CP, TIGIT, S100a11, PASK, 4930469K13Rik, NUBP1, CDC42SE1, NEK2, TRA, 2200002D01Rik, ESYT1, Trbv13-1, C7orf43, APOBEC3B, LGMN, KIF20B, RTN4, RAP1B, SLCO4A1, ECT2, PTAFR, TNFRSF1A, SLAIN1, IQGAP3, ACTR3, SYTL2, TRERF1, BIN1, LASP1 |
|  |
| **Common 30DPI and 15DPI down-regulated genes** |
| PXMP4, SLC38A3, GCAT, FAM19A5, Gm428 , PCDH12, HS3ST5, AQP8, Hrk, AKAP5, PNMA2, GAB3, C4orf19, B020031M17Rik, FOXO6, SYNM, HEYL, GCM1, LTBP4, ERMN, WIF1, Bex1, TAC1, INMT |
|  |
| **Common 45DPI and 15DPI up-regulated genes** |
| CENPJ, TMEM56, H2-T24, ISG20, TMTC4, PHACTR1, MOV10, RNF31, SERPINB9, ZNFX1, IL27RA, CNTN5, SGSM2, Klre1, CDS1 |
|  |
| **Common 45DPI and 15DPI down-regulated genes** |
| KIAA1456, CHST1, FRAT1, PTPRR, TIMP4, VWA3A, ABCB1, CXCL14, PTPRB, ADAMTSL2, CSDC2, TPD52L1, ADH1C, STMN2, MAL, |
|  |
| **Common 45DPI and 30DPI up-regulated genes** |
| CLEC4D, APOC2, TREM2, GPNMB, SPRR1A, WISP2, ABCC3, ATF7, SFRP2, HLA-DOA, RBM3, APOE, CEBPA, CSF1R, RGS10, NPC2, DKK3, Trbv14, Ppfia4, MYH7, PTPRO, CYP4V2, SYTL1, Fndc1, ACSBG1, IL6R, CAMK1D, IL7R, DOK3, CD160, FAM46C, PID1, ZFP36L2, CMTM7, TM6SF1, AXL, A530099J19Rik, Naip1 , Prh1/Prp2, TBC1D9, IL1B, TRAT1, SH2D1B, RAB5C, ASAH1, SORL1, PPP2R2C, CD101, KCNJ10, FAM49A, TMEM176A, PGBD5, CD200R1, TMEM176B, ARHGAP22, CASS4, LY6G5B, C130050O18Rik, RASSF4, LPIN2, CREBBP, Klra15, BLNK, CCL17, SRPX |
|  |
| **Common 45DPI and 30DPI down-regulated genes** |
| MYL1, IRS1, TACC2, C15orf52, FGL1, SLC26A6, CHRNA2, VLDLR, EPDR1, Poln, 9330102E08Rik, OGDHL, DBT, CALR3, MEIG1, MLYCD, RETSAT, C14orf37, ACVR2B, BC025920, DNAJC28, PLEKHB1, ARHGAP20, SLC7A4, FNDC4, SYNPO2, Nepn, PHKG1, SLC36A2, POLQ, TMEM82, GZMM, EEPD1, Acot5, SFRP5, TLN2, FAM131A, CPT2, Acot1, DLX3, PDK4, ALOX5, ANGPTL4, 2210407C18Rik, Cbr2, |
|  |
| **Common 15DPI,30DPI 45DPI up-regulated genes** |
| Gzmb, CXCL10, GZMA, Gm12250, Serpina3g , Cxcl9, CCL5, UBD, Apol9a/Apol9b, Ccl8, F830016B08Rik, 9930111J21Rik1/Gm12185, Igtp, CFB, GBP2, Ms4a4b , ZBP1, BATF2, Ccl7, Ifi47, PLAC8, CD274, Tgtp1/Tgtp2, IFNG, Ccl2, IFIT3, LCK, Irgm1, GBP4, Trbc2, CCL4, SOCS1, Trbc1, IRGM, FCGR3A/FCGR3B, GBP6, ISG15, Iigp1, Saa3, CH25H, CD6, PSMB8, C2, ITGB7, IRF7, GBP5, SLAMF8, CD3D, CD2, CD5, Gm9706, Gm4951, ZAP70, HLA-DMB, TAP1, HIST1H2AD, C15orf48, Cd52, PSMB9, LOC547349, ITGAX, IL12RB1, LGALS3, BCL2A1, LTB, IFIT2, SPN, GIMAP1-GIMAP5, RAC2, CD72, SLC2A6, CXCR6, CCL2, H2-Q8, IFIT1B, IRF1, GZMK, HCK, IL2RB, CTSW, DUSP2, NLRC5, CD3G, PTPRCAP, TBX21, FAM26F, CCR5, MARCO, CORO1A, HK3, PARP14, SKAP1, Gm6252, Oasl2, IRF8, HLA-DMA, Serpina3h, HLA-A, Acp5, SCN10A, CTSS, AIF1, PTPRC, ICOS, BIRC5, MYO1G, NKG7, XAF1, SAMHD1, CD74, ITK, LAT, CXCR3, Tlr12, H2-Q5, ITGB2, IL18BP, HLA-E, PSMB10, BST1, HLA-DQA1, HLA-DQB1, CDCA8, TAPBPL, RTP4, Bst2, KLRG1, HLA-DRB5, USP18, H2-T9, MPEG1, Mx1/Mx2, CST7, CASP4, OAS1, OASL, SOCS3, CARD11, ITGAL, Gm4759, LCP1, MKI67, IFI44, ADAM8, H2-T10, Gbp8, CCDC88B, VAV1, IFI30, Klra2, HIST1H2AB, PTPN6, NCF4, LY86, CD8B, TNF, E2F2, LILRB3, PARP9, THEMIS2, MYO1F, VCAM1, S100A4, Oas1f, RSAD2, Hist1h1b, Apol10a , DTX3L, IL2RG, PFKFB3, TIMP1, Pira7, FCER1G, RNF19B, PLK1, CASP1, CCNB2, TNFRSF4, OR51A7, C3, UBA7, SELPLG, SLC15A3, CYTIP, SLC16A3, COTL1, CXCL6, OAS3, RNF213, SLAMF9, CD300LF, LIMD2, TMEM173, GPR132, GPR171, TNFRSF18, GALNT6, CD40, C4A/C4B, PARP10, Gm8369, MS4A6A, HIST1H2AG, GLIPR1, FYB, ITGA4, TAP2, BATF3, LGALS3BP, SIRPB1, SASH3, FPR2, TOP2A, SNX20, UBE2L6, GNG2, Bcl2a1c, PTPN22, Pira11, HCST, CSF2RB, RGS1, DNASE1L3, CLEC4E, FCHO1, CD48, TRIM21, CD3E, IL21R, IFI16, LRRC25, TNFSF10, PLEK, CCR2, ID2, RASGRP1, B2M, CCNA2, CENPE, CYP4F2, GJD3, RHOF, MAP4K1, STAT2, HDC, KIF21B, LAIR1, CD8A, UPP1, THY1, PSTPIP1, DOK2, SLAMF7, SYTL3, IL10RA, CYTH4, Slfn2, FCGR1A, NFKBID, TRAF1, HCLS1, Trim30a/Trim30d, JAML, IFIH1, SLC11A1, SLFN13, WARS, PTPN7, DDX58, WDFY1, ARHGAP4, TUBB3, CD300E, MLKL, NAAA, KLRC1, GMFG, BATF, BCL3, PROCR, GIMAP7, CCL3L3, STAT1, ICAM1, UHRF1, Gvin1 , HLA-G, PANX1, RACGAP1, PILRA, Ly6a , ARHGAP45, CDCA5, RND1, CD300C, LAPTM5, SLA, PARVG, FASLG, KIF22, CCL19, CYBB, RAB32, SEMA4D, CTSC, NFKBIE, C1R, GLIPR2, SEMA4A, Ms4a6c, PLD4, PIK3CD, Clec4a3, ZNF705A, MCM5, EIF2AK2, CDCA3, EVI2A, FERMT3, IFI35, PSME2, PRC1, UBE2C, GPR68, NUP210, IL21, DHX58, FGL2, CD69, PDCD1LG2, SNX22, LPXN, H2-M11, NEURL3, RAB19, ZMYND15, CD68, RASSF5, HMOX1, GPR65, Gm8995, C1QA, NCKAP1L, TLR2, CMPK2, INSRR, CORO2A, NMI, TMEM140, UNC13D, C1QB, MYBL2, TYROBP, MS4A7, PARP12, B4GALNT1, ARHGDIB, MEFV, RGS14, B430306N03Rik, GFI1, CD7, Klra22, IL18R1, CXCL16, Gzmc, MTHFD2, WAS, SPC25, XDH, HPSE, KLRD1, RBL1, CD53, SEPT1, C1QC, AIM1, SP140, PSD4, EBI3, DENND2D, PRF1, TAPBP, BCL2L14, FGD2, MBP, PTPN18, DENND1C, Klra7 , ASS1, KCNN4, FXYD5, TNFAIP3, PHGDH, FIGNL1, CCNB1, GIMAP4, Tmsb4x , IRF5, Klra16, CAPG, APBB1IP, CX3CR1, PSAT1, TPX2, TLR1, BID, ASNS, LAG3, SNX10, STAT4, RGS16, VILL, FCGR2B, SH2D1A, STX11, LRMP, Pmaip1, CD44, RRM2, LYPD6B, FGR, Apol7e , RIPK3, TGFBI, SH3BP2, CCRL2, CFP, PIK3R5, ARID5A, IFITM3, PODNL1, CCNF, EGR2, RINL, DOCK2, AKNA, ATF3, HMGB2, Trbv13-2, TIFA, ARHGAP30, 9030619P08Rik, TMEM106A, AI661384, SERPING1, GSDMD, AOAH, DDIT3, Fbxw17, ADAR, KBTBD11, PSME1, TBXAS1, C16orf54, FPR1, UBASH3B, HIST2H2BF, MCM6, PLAUR, Tlr13, UNC93B1, Csprs , IL6, AURKA, BAK1, TNFAIP2, MSR1, NUSAP1, TSPAN32, Nlrp1a, GATM, UPB1, Trac, GPSM3, LAT2, SH3BGRL3, SGO1, CLEC9A, SAMSN1, SHCBP1, MGST2, 4933412E12Rik, MYD88, TACC3, SLC39A4, MAB21L3, GPR18, ATP8B4, CD86, CD84, DPEP2, EFHD2, PADI2, CDT1, ASPM, ACTB, DOCK10, BASP1, FBXO5, LCP2, ZC3H12D, E2F1, PPA1, CD96, STXBP2, LIME1, SHISA5, HP, 5730416F02Rik, ACSL5, NCF1, FGD3, Gm5150, MTHFD1L, IKZF1, DRAM1, FCGR2A, MCM3, TSPO, TMEM229B, ARHGAP9, NCAPH, SAA1, BCL11B, RBM43, CTSZ, INSL6, GPR84, PREX1, HAVCR2, TRIM59, INPP5D, SLA2, NAPSA, Gm10134, PDCD1, Slfn1, P2RX7, SH2D2A, WDFY4, BTK, NFAM1, PRR5L, FAM107B, EMP3, AP1S3, MS4A8, LY6E, RAD51, ARL5C, P2RY10, SKAP2, ALDH1A2, ABCG1, TRIP13, FAM105A, SMC2, CXCL2, TNFAIP8L2, PLA2G7, PAK1, C14orf80, PLA2G4A, Clec2d , TOR3A, TRIM14, TPM3, RUNX1, SKA1, PTK2B, ALOX5AP, CSF2RA, MARCKSL1, CDC20, SAMD9L, Tcstv3, ARRB2, LMNB1, CYSLTR2, GRAP2, CLEC6A, Trbv16, CLEC10A, LY9, CLIC1, 9330175E14Rik, CAP1, Gimap9, ALDOC, LITAF, CX3CL1, ETV6, CEACAM16, SERTAD2, MCUB, Trbv5, ARPC1B, EMB, KLRB1, IRF9, DOK1, CYBA, CRLF2, PGLYRP2, ARHGAP15, P2RY6, CCL1, Klra23, RPS6KA1, ESPL1, FAM111A, FUT7, CLEC4C, Srgn, TCIRG1, ALDH3B1, C19orf38, RIPK1, ADCY7, Lst1, POU2F2, PRKCB, ANKRD1, Pou3f1, SLC13A3, CASP8, TXK, CLEC7A, SLC7A7, SIT1, CFL1, Trbv13-3, Gm6377, TUBB2B, MCOLN2, VIM, GPR174, APOBEC1, GDA, RBM47, C5AR1, AI447881, NCF2, DOCK11, TRPV2, GMIP, MVP, ZC3HAV1, CD200R1L, IGSF6, GSG2, RAB43, SLC9A3R1, BC028789, PBK, A630001G21Rik, NAIP, G6PD, PLEKHO2, Oas1d , RGS19, TAGLN2, JAKMIP1, IQGAP1, E230029C05Rik, SH3BP1, CD300LD, CCR7, PFKP, PILRB, CADM1, IL3RA, DEF6, LSP1, PLCG2, SLC2A3, ADA, CNDP2, LFNG, CRMP1, CDKN2A, Casp12, PIK3AP1, C130026I21Rik , TNFSF14, PIK3CG, UGT1A6, GATA3, EDEM1, SDC3, IKBKE, SECTM1, LYZ, GDF15, SCPEP1, TNFSF13B, Klrb1f, KCNK5, Trbv4, PTGS2, TMEM51, HVCN1, C3AR1, PRKCD, SOAT2, RAB8B, PTPRE, PQLC3, IL17RA, PFN1, STK17B, Trbv29, ANPEP, RASA4, TNFRSF1B, IL18, RHOG, Hmga2-ps1, CXorf21, NOS2, TRAF3IP3, SPINT1, KCNK13, GALNT12, CD82, RAB3IL1, Krt85, SIRPA, FES, Ms4a6b, UBASH3A, BTG1, TLR6, CXCL13, Trbv12-2, AMZ1, TKT, ARF3, H2-M2, BSPRY, TGIF1, UCP2, UNC5CL, ABI3, MAG, CD226, BTLA, RASSF2, RNASE6, Trbv15, CD37, TNFRSF11B, OLFM1, |
|  |
| **Common 15DPI, 30DPI and DPI45 down-regulated genes** |
| CLEC18B, BC024137, WHRN, ABAT, NIT1, HSBP1L1, SCN4B, LINGO3, MAGIX, EPHB1, GCK, MLXIPL, ARMC2, YBX2, Adig, SLC40A1, STXBP6, PLK5, CTNNAL1, KCNV2, CD163, BDNF, ACTA1, CCL24, SYNPO, DCT, SLC22A1, Gm10635, Gm1673, EHHADH, HR, AMY2A, ANO10, EPOR, IGSF1, KLHL34, PENK, FIBIN, GSTM2, PDP2, MYBPC2, ST6GALNAC2, COMP, FLVCR2, Dcaf12l1, RASL10A, STXBP4, DBH, MSRB2, MYOT, EFNB3, FAH, AMPH, KCNA1, SRRM4, HMGCS2, HPGD, Gm4956, PFKFB1, NTF3, CYTL1, AQP1, Cdkn1c, Fcrls, GCHFR, Retnla, ALDOB, H19 |

| **Supplemental Table 5.** Pathways shared or not shared between different time points | | | |
| --- | --- | --- | --- |
|  |  | **Induced pathways** | **Repressed Pathways** |
| **Exclusive** | **15 DPI** | TWEAK Signaling | VDR/RXR Activation |
|  |  | Protein Kinase A Signaling | Angiopoietin Signaling |
|  |  | Role of BRCA1 in DNA Damage Response | Regulation of Cellular Mechanics by Calpain Protease |
|  |  | IGF-1 Signaling |  |
|  |  | Sumoylation Pathway |  |
|  |  | Role of NANOG in Mammalian Embryonic StemCell Pluripotency |  |
|  |  | Neuregulin Signaling |  |
|  |  | Melatonin Signaling |  |
|  |  | Androgen Signaling |  |
|  |  |  |  |
|  | **30DPI** | B Cell Activating Factor Signaling | none |
|  |  | April Mediated Signaling |  |
|  |  |  |  |
|  | **45DPI** | none | Acute Myeloid Leukemia Signaling |
|  |  |  | Wnt/β-catenin Signaling |
|  |  |  | Gαi Signaling |
|  |  |  | IGF-1 Signaling |
|  |  |  | EIF2 Signaling |
|  |  |  |  |
| **Common** | **30DPI and 15DPI** | Remodeling of Epithelial Adherens Junctions | STAT3 Pathway |
|  |  | Acute Myeloid Leukemia Signaling |  |
|  |  | Cell Cycle Regulation by BTG Family Proteins |  |
|  |  | Wnt/β-catenin Signaling |  |
|  |  | Gαi Signaling |  |
|  |  | Synaptic Long Term Potentiation |  |
|  |  |  |  |
|  | **45DPI and 15DPI** | Synaptic Long Term Depression | ErbB2-ErbB3 Signaling |
|  |  | Aldosterone Signaling in Epithelial Cells | Insulin Receptor Signaling |
|  |  | Nitric Oxide Signaling in the Cardiovascular System |  |
|  |  |  |  |
|  | **45DPI and 30DPI** | Angiopoietin Signaling | TWEAK Signaling |
|  |  | VDR/RXR Activation | Protein Kinase A Signaling |
|  |  | MIF-mediated Glucocorticoid Regulation | Role of BRCA1 in DNA Damage Response |
| **Common** | **45DPI and 30DPI** | Cardiac Hypertrophy Signaling | Sumoylation Pathway |
| (continued) | (continued) | cAMP-mediated signaling |  |
|  |  | TGF-β Signaling |  |
|  |  | Calcium Signaling |  |
|  |  |  |  |
|  | **15DPI, 30DPI and DPI45** | Th1 Pathway | OX40 Signaling Pathway |
|  |  | Role of Pattern Recognition Receptors in Recognition of Bacteria and Viruses | LXR/RXR Activation |
|  |  | Type I Diabetes Mellitus Signaling | NF-κB Activation by Viruses |
|  |  | Th2 Pathway | Cell Cycle: G2/M DNA Damage Checkpoint Regulation |
|  |  | CD28 Signaling in T Helper Cells | RhoGDI Signaling |
|  |  | Dendritic Cell Maturation | Antioxidant Action of Vitamin C |
|  |  | iCOS-iCOSL Signaling in T Helper Cells | PTEN Signaling |
|  |  | TREM1 Signaling | Ephrin B Signaling |
|  |  | Fcγ Receptor-mediated Phagocytosis in Macrophages and Monocytes | PPAR Signaling |
|  |  | Tec Kinase Signaling | Huntington's Disease Signaling |
|  |  | Cdc42 Signaling | Role of p14/p19ARF in Tumor Suppression |
|  |  | Death Receptor Signaling | Cell Cycle: G1/S Checkpoint Regulation |
|  |  | Role of NFAT in Regulation of the Immune Response | p53 Signaling |
|  |  | PKCθ Signaling in T Lymphocytes | ATM Signaling |
|  |  | Production of Nitric Oxide and Reactive Oxygen Species in Macrophages | Role of CHK Proteins in Cell Cycle Checkpoint Control |
|  |  | Activation of IRF by Cytosolic Pattern Recognition Receptors | PPARα/RXRα Activation |
|  |  | Interferon Signaling |  |
|  |  | Acute Phase Response Signaling |  |
|  |  | NF-κB Signaling |  |
|  |  | Complement System |  |
|  |  | Calcium-induced T Lymphocyte Apoptosis |  |
|  |  | Cytotoxic T Lymphocyte-mediated Apoptosis of Target Cells |  |
|  |  | Induction of Apoptosis by HIV1 |  |
|  |  | B Cell Receptor Signaling |  |
|  |  | IL-8 Signaling |  |
|  |  |  |  |
| **Common** | **15DPI, 30DPI and DPI45** | Phospholipase C Signaling |  |
| (continued) | (continued) | Inflammasome pathway |  |
|  |  | Retinoic acid Mediated Apoptosis Signaling |  |
|  |  | GM-CSF Signaling |  |
|  |  | Apoptosis Signaling |  |
|  |  | Growth Hormone Signaling |  |
|  |  | IL-6 Signaling |  |
|  |  | PI3K Signaling in B Lymphocytes |  |
|  |  | Mitotic Roles of Polo-Like Kinase |  |
|  |  | fMLP Signaling in Neutrophils |  |
|  |  | Tumoricidal Function of Hepatic Natural Killer Cells |  |
|  |  | Estrogen-mediated S-phase Entry |  |
|  |  | TNFR1 Signaling |  |
|  |  | Type II Diabetes Mellitus Signaling |  |
|  |  | Prolactin Signaling |  |
|  |  | Renin-Angiotensin Signaling |  |
|  |  | IL-9 Signaling |  |
|  |  | HMGB1 Signaling |  |
|  |  | RhoA Signaling |  |
|  |  | Endothelin-1 Signaling |  |
|  |  | Eicosanoid Signaling |  |
|  |  | TNFR2 Signaling |  |
|  |  | CD40 Signaling |  |
|  |  | FcγRIIB Signaling in B Lymphocytes |  |
|  |  | Role of RIG1-like Receptors in Antiviral Innate Immunity |  |
|  |  | Signaling by Rho Family GTPases |  |
|  |  | Paxillin Signaling |  |
|  |  | UVA-Induced MAPK Signaling |  |
|  |  | Lymphotoxin β Receptor Signaling |  |
|  |  | SAPK/JNK Signaling |  |
|  |  | Macropinocytosis Signaling |  |
|  |  | Actin Cytoskeleton Signaling |  |
|  |  | Chemokine Signaling |  |
|  |  |  |  |
| **Common** | **15DPI, 30DPI and DPI45** | JAK/Stat Signaling |  |
| (continued) | (continued) | Sphingosine-1-phosphate Signaling |  |
|  |  | ILK Signaling |  |
|  |  | Role of IL-17F in Allergic Inflammatory Airway Diseases |  |
|  |  | iNOS Signaling |  |
|  |  | Glioma Invasiveness Signaling |  |
|  |  | LPS/IL-1 Mediated Inhibition of RXR Function |  |
|  |  | Glioma Signaling |  |
|  |  | Corticotropin Releasing Hormone Signaling |  |
|  |  | ERK/MAPK Signaling |  |
|  |  | Neuropathic Pain Signaling In Dorsal Horn Neurons |  |
|  |  | Ephrin Receptor Signaling |  |
|  |  | IL-2 Signaling |  |
|  |  | PDGF Signaling |  |
|  |  | IL-17A Signaling in Airway Cells |  |
|  |  | Cyclins and Cell Cycle Regulation |  |
|  |  | Actin Nucleation by ARP-WASP Complex |  |
|  |  | Agrin Interactions at Neuromuscular Junction |  |
|  |  | eNOS Signaling |  |
|  |  | PEDF Signaling |  |
|  |  | LPS-stimulated MAPK Signaling |  |
|  |  | Glioblastoma Multiforme Signaling |  |
|  |  | Cholecystokinin/Gastrin-mediated Signaling |  |
|  |  | G Beta Gamma Signaling |  |
|  |  | Role of PI3K/AKT Signaling in the Pathogenesis of Influenza |  |
|  |  | Neurotrophin/TRK Signaling |  |
|  |  | Ceramide Signaling |  |
|  |  | PI3K/AKT Signaling |  |
|  |  | UVB-Induced MAPK Signaling |  |
|  |  | MIF Regulation of Innate Immunity |  |
|  |  | p70S6K Signaling |  |
|  |  | ErbB4 Signaling |  |
|  |  | NGF Signaling |  |
|  |  |  |  |
| **Common** | **15DPI, 30DPI and DPI45** | CXCR4 Signaling |  |
| (continued) | (continued) | VEGF Family Ligand-Receptor Interactions |  |
|  |  | Antiproliferative Role of Somatostatin Receptor 2 |  |
|  |  | Non-Small Cell Lung Cancer Signaling |  |
|  |  | CNTF Signaling |  |
|  |  | Melanocyte Development and Pigmentation Signaling |  |
|  |  | NRF2-mediated Oxidative Stress Response |  |
|  |  | mTOR Signaling |  |
|  |  | P2Y Purigenic Receptor Signaling Pathway |  |
|  |  | PAK Signaling |  |
|  |  | Leptin Signaling in Obesity |  |
|  |  | Relaxin Signaling |  |
|  |  | Sperm Motility |  |
|  |  | GDNF Family Ligand-Receptor Interactions |  |
|  |  | Gα12/13 Signaling |  |
|  |  | CREB Signaling in Neurons |  |
|  |  | Thrombin Signaling |  |
|  |  | EGF Signaling |  |
|  |  | α-Adrenergic Signaling |  |
|  |  | Mouse Embryonic Stem Cell Pluripotency |  |
|  |  | IL-1 Signaling |  |
|  |  | Role of NFAT in Cardiac Hypertrophy |  |
|  |  | Renal Cell Carcinoma Signaling |  |
|  |  | RANK Signaling in Osteoclasts |  |
|  |  | FGF Signaling |  |
|  |  | AMPK Signaling |  |
|  |  | Telomerase Signaling |  |
|  |  |  |  |
|  |  |  |  |
|  |  |  |  |
|  |  |  |  |

**Supplementary table 6 :** Differentially expressed genes belonging to the IFNG- modulated, Nrf2-modulated or mitochondrial genes in each time point

| **Gene expression analysis** | | |
| --- | --- | --- |
| **DEG**  **15 dpi vs CTL (n=1128)** | **169/1067 IFNG induced genes** | |
|  | Acp5*, ACTB*, ADA*, ADAM8*, ADAR*, AIM1*, ALDOC*, ANXA2*, AOAH*, APOD*, ARL4D, ASS1*, ATF3*, B2M*, BATF3*, BST1*, BTG1*, C1QA*, C1R*, C2*, C3*, C3AR1*, C4A/C4B*, CASP1*, Ccl2*, CCL4*, CCL5*, CCR5*, CCRL2*, CD40*, CD44*, CD47*, CD74*, CD86*, CDKN2A*, CFB*, CLIC1*, CMPK2*, CORO1A*, CTSC*, CTSS*, CTSZ*, CXCL10*, Cxcl9*, CYBB*, DAXX*, DBNL*, FAM105A*, FAM107B*, FCGR1A*, FCGR2A*, FCHO1*, Fcrls, FGR*, FLNA*, FXYD5*, G6PD*, GBP2*, GBP4*, GNA13*, GPR65*, HLA-A*, HLA-DMA*, HLA-DMB*, HLA-DQA1*, HLA-DQB1*, HLA-DRB5*, HLA-E*, HLA-G*, HMOX1*, HPGD, ICAM1*, IFI16*, IFI30*, IFI35*, Ifi47*, IFIH1*, IFIT1B*, IFIT2*, IFIT3*, IFITM3*, Igtp*, Iigp1*, IL10*, IL12RB1*, IL17RA*, IL18*, IL2RG*, IL4R*, INPP1*, IRF1*, IRF5*, IRF7*, IRF8*, Irgm1*, ISG15*, ITGB7*, Klra2*, LGALS3BP*, LILRB3*, LY6E*, LY86*, MOV10*, MPEG1*, Ms4a6b*, Mx1/Mx2*, NMI*, NOS2*, OGFR*, PFKP*, PIM1*, PLA2G4A*, PLA2G7*, PLAC8*, PLAUR*, PLD4*, PRC1*, PROCR*, PSMA5*, PSMB10*, PSMB8*, PSMB9*, PSME1*, PSME2*, PTGS2*, PTPN22*, RCC2*, RGS1*, RGS14*, RGS19*, S100A4*, Saa3*, SAMHD1*, SDC3*, SELL*, SEMA4A*, SERPINB9*, SERPING1*, SGO1*, SLC11A1*, SOAT2*, SOCS1*, Srgn*, STAT1*, STAT3*, STXBP1*, TAP1*, TAP2*, TAPBP*, TBXAS1*, Tgtp1/Tgtp2*, TKT*, TNFAIP2*, TNFRSF1A*, TRAF1*, TRAFD1*, TRIM21*, Trim30a/Trim30d*, TSPO*, TUBA1B*, TUBB*, TUBB3*, UBA7*, UBE2L6*, UPP1*, USP18*, VCAM1*, WARS*, ZNFX1* | |
|  | **56/126 Mitochondrial expressed genes** | |
|  | ABAT, ABCG1*, ACSL5*, ACSM5*, ALDOC*, BAK1*, BID*, CASP4*, CASP8*, CDK1*, CDKN2A*, CLIC1*, CLIC4*, CMPK2*, CYBA*, CYBB*, DDIT4, EHHADH, FEN1*, GATM*, GCAT, GCK, GIMAP8*, GLRX*, GRN*, HCLS1*, HK3*, HMGCS2, Hrk, IFIT3*, LIG1*, MSRB2, MTHFD1L*, MTHFD2*, NEFH*, OAS2*, PDP2, Pmaip1*, PRELID1*, PRKCD*, PRR5L*, PYCARD*, RAB32*, RAB8B*, RAD51*, RIPK1*, RIPK3*, RSAD2*, SGK1, SOX4, STXBP1*, TAP1*, TCIRG1*, TSPO*, UCP2*, XAF1* | |
|  | **25/341 Nrf2-modulated genes** | |
|  | ASNS*, B4GALNT1*, CYP4F2*, DOCK10*, FEN1*, G6PD*, GCK, GPR68*, HIST1H2AB*, HMOX1*, Hrk, MCM10*, MTHFD2*, NCF2*, NUPR1*, P2RY6*, PARP10*, PHGDH*, PSAT1*, RRM2*, Srgn*, TKT*, TLR6*, TREX1*, UGT1A6* | |
|  |  | |
|  | **201/1067 IFNG induced genes** | |
| **DEG**  **30 dpi vs CTL (n=1479)** | ABHD14A, Acp5**, ACSL1, ACTB**, ADA**, ADAM8**, ADAR**, AIM1**, Akr1c12/Akr1c13**, ALCAM**, ALDOC**, ANXA1**, ANXA2**, AOAH**, APBB1, APOD**, ARF6**, ASS1**, ATF3**, AUH**, AXL**, B2M**, BATF3**, BCL2L13, BST1**, BTG1**, C1QA**, C1R**, C2**, C3**, C3AR1**, C4A/C4B**, CASP1**, Cbr2, Ccl2**, CCL4**, CCL5**, Ccl9**, CCNH, CCR5**, CCRL2**, CD14**, CD38**, CD40**, CD44**, CD74**, CD86**, CDKN2A**, CFB**, CHCHD7, CLEC4D**, CLIC1**, CMPK2**, CORO1A**, CTSC**, CTSH**, CTSS**, CTSZ**, CXCL10**, Cxcl9**, CXCR4**, CYBB**, DLAT, EXOC8, FAM105A**, FAM107B**, FASTK, FCGR1A**, FCGR2A**, FCHO1**, Fcrls, FGR**, FKBP4, FLNA**, FXYD5**, G6PD**, GBA**, GBP2**, GBP4**, GFRA3, GNA13**, GPR65**, HLA-A**, HLA-DMA**, HLA-DMB**, HLA-DOA**, HLA-DQA1**, HLA-DQB1**, HLA-DRB5**, HLA-E**, HLA-G**, HMOX1**, HPGD, ICAM1**, IFI16**, IFI30**, IFI35**, Ifi47**, IFIH1**, IFIT1B**, IFIT2**, IFIT3**, IFITM3**, Igtp**, Iigp1**, IL12RB1**, IL17RA**, IL18**, IL2RG**, IL4R**, IRF1**, IRF5**, IRF7**, IRF8**, Irgm1**, ISG15**, ITGB7**, Klra2**, LCN2**, LGALS3BP**, LILRB3**, LPIN2**, LY6E**, LY86**, MAFB**, MAN2A1**, MECR, MERTK**, MPEG1**, Ms4a6b**, Mx1/Mx2**, NMI**, NOS2**, NOTCH2**, PES1, PF4, PFKP**, PIM1**, PLA2G4A**, PLA2G7**, PLAC8**, PLAUR**, PLD4**, POSTN**, PPT1**, PRC1**, PROCR**, PSMB10**, PSMB8**, PSMB9**, PSME1**, PSME2**, PTGS2**, PTPN22**, RCC2**, REEP5, RENBP**, RETSAT, RGS1**, RGS14**, RGS19**, S100A4**, Saa3**, SAMHD1**, SDC3**, SELL**, SEMA4A**, SERPINA3**, SERPING1**, SGO1**, SLC11A1**, SLC5A6, SOAT2**, SOCS1**, SPP1**, Srgn**, SRPX**, STAT1**, TAP1**, TAP2**, TAPBP**, TBXAS1**, Tgtp1/Tgtp2**, TKT**, TNFAIP2**, TNFRSF1A**, TRAF1**, TRIM21**, Trim30a/Trim30d**, TSPAN33**, TSPO**, TUBB3**, UBA7**, UBE2L6**, UPP1**, USP18**, VASP**, VCAM1**, WARS**, WDR92**, ZYX** | |
|  | **110/126 Mitochondrial expressed genes** |  |
|  | AARS2, ABAT, ABCB6, ABCG1**, ACAA2, ACOT2, ACSL1, ACSL5**, ADHFE1, ALDH6A1, ALDOC**, BAK1**, BCL2L13, BID**, BOLA1, BPHL, CAPRIN2, CARS2, CASP4**, CASP8**, CDK1**, CDKN2A**, CLIC1**, CMPK2**, COQ9, CPOX, CPT2, CYBA**, CYBB**, CYP1B1**, DBT, DCAKD, DLAT, ECH1, ECHDC3, EHHADH, EYA2**, FASTK, FKBP4, GATM**, GCAT, GCDH, GCK, GLRX**, GM2A**, GPX1**, GRN**, HADH, HADHA, HADHB, HCLS1**, HIBCH, HK3**, HMGCS2, Hrk, Hsdl2, HSPD1, IFIT3**, L2HGDH, LACE1, LDHD, MCCC1, MCCC2, MCEE, MECR, MLYCD, MSRB2, MTHFD1L**, MTHFD2**, MUT, NDUFA1, NME4, NT5C**, OGDHL, OXCT1, PDK3**, PDK4, PDP1, PDP2, Pmaip1**, POLG, PRELID1**, PRKCD**, PRR5L**, PXMP2, PYCARD**, RAB32**, RAB8B**, RAD51**, REEP1, RIPK1**, RIPK3**, RMND1, RSAD2**, SLC25A26, SLC25A42, SLC25A45**, SNCA**, SORD, SUOX, TAP1**, TCIRG1**, TMEM143, TMEM65, TSPO**, TUFM, UCP2**, UROS, VARS2, XAF1** | |
|  | **39/341 Nrf2 genes** | |
|  | ABCB6, ASNS**, B4GALNT1**, CCL17**, CHAC1, CYP4F2**, DDC, DOCK10**, ECM2, FTL**, G6PD**, GCK, GPNMB**, GPR68**, GPT2, HIST1H2AB**, HMOX1**, Hrk, MTHFD2**, NCF2**, NUPR1**, P2RY6**, PARP10**, PHGDH**, POLQ, PPP2R2C**, PSAT1**, RDM1, REEP1, RRM2**, SORD, SPP1**, Srgn**, SRI**, TKT**, TLR6**, TMEM177, UGT1A6**, VLDLR | |
|  | **35/150 NF-KB target genes** | |
|  | ARHGAP22**, BCL3**, CASP4**, Ccl2**, CCL4**, Ccl7**, CCR5**, CCR7**, CD3G**, CD40**, CD48**, CXCL10**, CXCL2**, Cxcl9**, FUT7**, HMOX1**, ICAM1**, IL1B**, IRF1**, IRF7**, LTB**, NOS2**, PIM1**, PLA2G4A**, PRF1**, PSMB9**, PTGS2**, S100A6**, SELP**, TAP1**, TNF**, TNFAIP3**, TRAF1**, VCAM1**, VIM** | |
|  |  | |
|  |  | |
|  |  | |
| **DEG**  **45 dpi vs CTL (n=1003)** |  | |
|  | **45/126 Mitochondrial expressed genes** |  |
|  | ABAT, ABCG1***, ACSL5***, ALAS2***, ALDOC***, BAK1***, BID***, CASP4***, CASP8***, CDKN2A***, CLIC1***, CMPK2***, CPT2, CYBA***, CYBB***, DBT, EHHADH, GATM***, GCK, HCLS1***, HK3***, HMGCS2, IFIT3***, MLYCD, MSRB2, MTHFD1L***, MTHFD2***, OGDHL, PDK4, PDP2, PLIN5, Pmaip1***, PRKCD***, PRR5L***, RAB32***, RAB8B***, RAD51***, RIPK1***, RIPK3***, RSAD2***, TAP1***, TCIRG1***, TSPO***, UCP2***, XAF1*** | |
|  | **29/341 Nrf2 genes** | |
|  | ASNS***, B4GALNT1***, CAMK1G***, CCL17***, CYP4F2***, DOCK10***, G6PD***, GCK, GPNMB***, GPR68***, GSTA5, HIST1H2AB***, HMOX1***, MTHFD2***, NCF2***, P2RY6***, PARP10***, PHGDH***, PHGDH***, POLQ, PPP2R2C***, PSAT1***, RRM2***, Srgn***, TKT***, TLR6***, UGT1A6***, VLDLR, ZNF467 | |
|  | **36/150 NF-KB target genes** | |
|  | ARHGAP22***, BCL3***, CASP4***, CCL2***, Ccl2***, CCL4***, Ccl7***, CCR5***, CCR7***, CD3G***, CD40***, CD48***, CD83***, CXCL10***, CXCL2***, Cxcl9***, FUT7***, HMOX1***, ICAM1***, IER3***, IL1B***, IRF1***, IRF7***, LTB***, NOS2***, PLA2G4A***, PRF1***, PSMB9***, PTGDS, PTGS2***, TAP1***, TNF***, TNFAIP3***, TRAF1***, VCAM1***, VIM*** | |
|  |  | |

*DEG that are up regulated between 15 dpi mice and CTL mice.

**DEG that are up regulated between 30 dpi mice and CTL mice.

***DEG that are up regulated between 45 dpi mice and CTL mice.

**Supplemental table 7 :** DEGs shared between IFNγ-,Nrf2-modulated genes or mitochondrial genes.

15 dpi vs CTL

|  | **IFNγ- modulated genes** | **Mitochondrial genes** |
| --- | --- | --- |
| **Nrf2-modulated genes** | **n=4**  G6PD*, HMOX1*, Srgn*, TKT* | **n=4**  FEN1*, GCK, Hrk, MTHFD2* |
| **Mitochondrial genes** | **n=9**  ALDOC*, CDKN2A*, CLIC1*, CMPK2*, CYBB*, IFIT3*, STXBP1*, TAP1*, TSPO* |  |

*DEG that are up regulated between 15 dpi mice and CTL mice.

30 dpi vs CTL

|  | **IFNγ- modulated genes** | **Mitochondrial genes** |
| --- | --- | --- |
| **Nrf2-modulated genes** | **n=5**  G6PD**, HMOX1**, SPP1**,  Srgn**, TKT** | **n=6**  ABCB6, GCK, Hrk, MTHFD2**,  REEP1, SORD |
| **Mitochondrial genes** | **n=14**  ACSL1, ALDOC**, BCL2L13, CDKN2A**, CLIC1**, CMPK2**, CYBB**, DLAT,  FASTK, FKBP4, IFIT3**, MECR,  TAP1**, TSPO** |  |

**DEG genes that are up regulated between 30 dpi mice and CTL mice.

**45 dpi vs CTL**

|  | **IFNγ- modulated genes** | **Mitochondrial genes** |
| --- | --- | --- |
| **Nrf2-modulated genes** | **n=4**  G6PD***, HMOX1***, Srgn***, TKT*** | **n=2**  GCK, MTHFD2*** |
| **Mitochondrial genes** | **n=8**  ALDOC***, CDKN2A***, CLIC1***, CMPK2***, CYBB***, IFIT3***,  TAP1***, TSPO*** |  |

***DEG genes that are up regulated between 45 dpi mice and CTL mice.

**Supplemental table 8:** Target genes of miR-149-5p, the miR with most targets at 15 dpi.

| **15dpi** | | | | |
| --- | --- | --- | --- | --- |
| **miR-149-5p (MIMAT0000159): Fold change: -1,612** | | | | |
| **Target DEGs** | | | **DEM-DEG targeting information** | |
| **Entrez Name** | **ID** | **Fold Change** | **Confidence** | **Source** |
| CD48 molecule | CD48 | 6,661 | High (predicted) | TargetScan Human |
| CD68 molecule | CD68 | 4,929 | High (predicted) | TargetScan Human |
| CNDP dipeptidase 2 | CNDP2 | 2,404 | High (predicted) | TargetScan Human |
| colony stimulating factor 1 | CSF1 | 2,119 | High (predicted) | TargetScan Human |
| E2F transcription factor 1 | E2F1 | 3,473 | Exp. Observed | IPA Expert Findings |
| Fas ligand | FASLG | 5,385 | High (predicted) | TargetScan Human |
| Fc fragment of IgG receptor IIIa | FCGR3A/3B | 32,501 | High (predicted) | TargetScan Human |
| interleukin 6 | IL6 | 3,774 | High (predicted) | TargetScan Human |
| leukotriene B4 receptor | LTB4R | 4,264 | High (predicted) | TargetScan Human |
| MARCKS like 1 | MARCKSL1 | 3,003 | High (predicted) | TargetScan Human |
| NLR family CARD domain containing 5 | NLRC5 | 15,242 | High (predicted) | TargetScan Human |
| RAB3A interacting protein like 1 | RAB3IL1 | 2,162 | High (predicted) | TargetScan Human |
| member of RAS oncogene family | RAP1B | 2,046 | Exp. Observed | IPA Expert Findings |
| SLAM family member 7 | SLAMF7 | 6,097 | High (predicted) | TargetScan Human |

**Supplemental table 9 :** Target genes of miR-138-5p, the miR with most targets at 30 dpi.

| **30 dpi** | | | | |
| --- | --- | --- | --- | --- |
| **miR-138-5p (MIMAT0000150): Fold change: -1,81** | | | | |
| **Target DEGs** | | | **DEM-DEG targeting info** | |
| **Entrez Name** | **ID** | **Fold Change** | **Confidence** | **Source** |
| aldehyde dehydrogenase 1 family member A2 | ALDH1A2 | 3,744 | Exp. Observed | miRecords |
| apolipoprotein B mRNA editing enzyme catalytic subunit 3B | APOBEC3B | 2,030 | High (predicted) | TargetScan |
| CKLF like MARVEL transmembrane domain containing 3 | CMTM3 | 2,042 | High (predicted) | TargetScan |
| cathepsin H | CTSH | 3,005 | High (predicted) | TargetScan |
| Fc fragment of IgE receptor Ig | FCER1G | 10,786 | High (predicted) | TargetScan |
| FES proto-oncogene, tyrosine kinase | FES | 2,767 | High (predicted) | TargetScan |
| glucose-6-phosphate dehydrogenase | G6PD | 3,454 | High (predicted) | TargetScan |
| GM2 ganglioside activator | GM2A | 2,849 | High (predicted) | TargetScan |
| G protein subunit gamma 2 | GNG2 | 8,224 | High (predicted) | TargetScan |
| G protein-coupled receptor 171 | GPR171 | 7,302 | High (predicted) | TargetScan |
| integrin subunit alpha L | ITGAL | 9,489 | High (predicted) | TargetScan |
| myeloid differentiation primary response 88 | MYD88 | 2,372 | High (predicted) | TargetScan |
| neutrophil cytosolic factor 4 | NCF4 | 11,326 | High (predicted) | TargetScan |
| podoplanin | PDPN | 2,013 | High (predicted) | TargetScan |
| shisa family member 5 | SHISA5 | 3,133 | High (predicted) | TargetScan |
| thymocyte selection associated family member 2 | THEMIS2 | 7,957 | High (predicted) | TargetScan |
| transmembrane protein 119 | TMEM119 | 2,378 | High (predicted) | TargetScan |
| translocator protein | TSPO | 3,399 | High (predicted) | TargetScan |
| ubiquitin specific peptidase 18 | USP18 | 4,485 | High (predicted) | TargetScan |
| vimentin | VIM | 3,827 | High (predicted) | TargetScan |
| ZFP36 ring finger protein like 2 | ZFP36L2 | 2,698 | High (predicted) | TargetScan |
| zinc finger protein 705A | ZNF705A | 7,741 | High (predicted) | TargetScan |

**Supplemental Table 10:** Target genes of miR16-5p, the miR with most targets at 45 dpi.

| **45 dpi** | | | | |
| --- | --- | --- | --- | --- |
| **miR-16-5p (MIMAT0000225): Fold change: -2,144** | | | | |
| **Target DEGs** | | | **DEM-DEG targeting information** | |
| **Entrez Name** | **Fold Change** | **ID** | **Confidence** | **Source** |
| B and T lymphocyte associated | 2,164 | BTLA | High (predicted) | TargetScan |
| cell adhesion molecule 1 | 3,484 | CADM1 | Exp. Observed | miRecords |
| cyclin F | 2,274 | CCNF | Exp. Observed | IPA Expert Findings |
| centromere protein J | 2,855 | CENPJ | Exp. Observed | miRecords |
| G protein-coupled receptor 171 | 6,057 | GPR171 | High (predicted) | TargetScan |
| heme oxygenase 1 | 2,686 | HMOX1 | Exp. Observed | TarBase |
| potassium calcium-activated channel subfamily N 4 | 4,696 | KCNN4 | Exp. Observed | TarBase,TargetScan |
| lipopolysaccharide induced TNF factor | 2,056 | LITAF | High (predicted) | TargetScan |
| lymphocyte antigen 9 | 4,411 | LY9 | High (predicted) | TargetScan |
| pannexin 1 | 5,305 | PANX1 | Exp. Observed | TarBase |
| polo like kinase 1 | 4,224 | PLK1 | Exp. Observed | TarBase |
| phosphoserine aminotransferase 1 | 2,444 | PSAT1 | Exp. Observed, | TarBase,TargetScan |
| prostaglandin-endoperoxide synthase 2 | 2,063 | PTGS2 | Exp. Observed | TarBase |
| src kinase associated phosphoprotein 2 | 2,792 | SKAP2 | Exp. Observed | miRecords |
| solute carrier family 16 member 3 | 6,068 | SLC16A3 | Exp. Observed | TarBase |
| solute carrier family 2 member 3 | 2,119 | SLC2A3 | High (predicted) | TargetScan |
| sushi repeat containing protein, X-linked | 2,130 | SRPX | High (predicted) | TargetScan |
| tumor necrosis factor superfamily member 13b | 4,503 | TNFSF13B | High (predicted) | TargetScan |
| tropomyosin 3 | 2,275 | TPM3 | Exp. Observed | TarBase,TargetScan |
| uncoupling protein 2 | 2,120 | UCP2 | Exp. Observed | IPA Expert Findings |

**Supplemental table 11:** DEM-DEG interaction prediction for IFNγ-, Nrf2- modulated genes, and mitochondrial genes.

| **15 dpi IFNγ- modulated genes** | **ID** | **Symbol** | **Expr Fold Change** | **Source** | **Confidence** | **Symbol** | **Expr p-value** | **Expr Fold Change** |
| --- | --- | --- | --- | --- | --- | --- | --- | --- |
|  | MIMAT0000656 | miR-139-5p | -1,633 | TargetScan Human | High predicted | CTSC | 3,48E-10 | 5,349 |
|  | MIMAT0000157 | miR-145-5p | -1,693 | TargetScan Human | High predicted | ACTB | 1,35E-08 | 3,496 |
|  | MIMAT0000157 | miR-145-5p | -1,693 | TargetScan Human | High predicted | IFI30 | 3,47E-09 | 9,393 |
|  |  |  |  |  |  |  |  |  |
| **15 dpi mitochondrial genes** | **ID** | **Symbol** | **Expr Fold Change** | **Source** | **Confidence** | **Symbol** | **Expr p-value** | **Expr Fold Change** |
|  | MIMAT0000670 | miR-221-3p | 1,999 | Ingenuity Expert Findings,TargetScan Human,miRecords | Experimentally Observed | DDIT4 | 1,49E-04 | -3,550 |
|  |  |  |  |  |  |  |  |  |
| **15 dpi**  **nrf2-modulated genes** | **ID** | **Symbol** | **Expr Fold Change** | **Source** | **Confidence** | **Symbol** | **Expr p-value** | **Expr Fold Change** |
|  | none | none | none | none | none | none | none | none |
|  |  |  |  |  |  |  |  |  |
|  |  |  |  |  |  |  |  |  |
| **30dpi IFNγ- modulated genes** | **ID** | **Symbol** | **Expr Fold Change** | **Source** | **Confidence** | **Symbol** | **Expr p-value** | **Expr Fold Change** |
|  | MIMAT0000138 | miR-126a-3p | -1,800 | TarBase,miRecords | Experimentally Observed | VCAM1 | 4,33E-10 | 6,580 |
|  | MIMAT0000150 | miR-138-5p | -1,810 | TargetScan Human | High predicted | CTSH | 1,69E-09 | 3,005 |
|  | MIMAT0000150 | miR-138-5p | -1,810 | TargetScan Human | High predicted | G6PD | 4,43E-11 | 3,454 |
|  | MIMAT0000150 | miR-138-5p | -1,810 | TargetScan Human | High predicted | TSPO | 1,08E-09 | 3,399 |
|  | MIMAT0000150 | miR-138-5p | -1,810 | TargetScan Human | High predicted | USP18 | 2,68E-09 | 4,485 |
|  | MIMAT0000656 | miR-139-5p | -2,540 | TargetScan Human | High predicted | CTSC | 2,39E-10 | 5,027 |
|  | MIMAT0000435 | miR-143-3p | -2,070 | TargetScan Human | High predicted | HLA-DOA | 7,66E-10 | 4,172 |
|  | MIMAT0000435 | miR-143-3p | -2,070 | TargetScan Human | High predicted | IFIT3 | 1,74E-13 | 18,800 |
|  | MIMAT0000435 | miR-143-3p | -2,070 | TargetScan Human | High predicted | IL18 | 8,08E-07 | 2,750 |
|  | MIMAT0000435 | miR-143-3p | -2,070 | Ingenuity Expert Findings | Experimentally Observed | PRC1 | 1,02E-08 | 5,285 |
|  | MIMAT0000435 | miR-143-3p | -2,070 | TargetScan Human | High predicted | SELL | 5,46E-04 | 3,076 |
|  | MIMAT0000157 | miR-145-5p | -2,720 | TargetScan Human | High predicted | ACTB | 7,95E-08 | 2,835 |
|  | MIMAT0000157 | miR-145-5p | -2,720 | TargetScan Human | High predicted | IFI30 | 8,98E-10 | 10,046 |
|  | MIMAT0000159 | miR-149-5p | -4,720 | TargetScan Human | High predicted | SRPX | 1,12E-04 | 2,032 |
|  | MIMAT0003212 | miR-17-3p | 3,140 | TargetScan Human | High predicted | REEP5 | 4,00E-06 | -2,045 |
|  | MIMAT0000214 | miR-185-5p | -1,590 | TargetScan Human | High predicted | SDC3 | 1,26E-07 | 2,417 |
|  | MIMAT0000237 | miR-204-5p | -2,810 | miRecords | Experimentally Observed | CTSC | 2,39E-10 | 5,027 |
|  | MIMAT0000237 | miR-204-5p | -2,810 | TargetScan Human | High predicted | HLA-DRB5 | 2,66E-08 | 16,501 |
|  | MIMAT0000237 | miR-204-5p | -2,810 | TargetScan Human | High predicted | PSME1 | 6,24E-09 | 2,525 |
|  | MIMAT0000534 | miR-26a-5p | -1,530 | Ingenuity Expert Findings,TargetScan Human | Experimentally Observed | PTGS2 | 2,80E-05 | 2,326 |
|  | MIMAT0000088 | miR-30a-3p | -2,130 | TargetScan Human | High predicted | ADA | 2,63E-06 | 2,642 |
|  | MIMAT0000514 | miR-30c-5p | -2,440 | TargetScan Human | High predicted | GNA13 | 1,17E-03 | 2,926 |
|  | MIMAT0000514 | miR-30c-5p | -2,440 | TargetScan Human | High predicted | SOCS1 | 6,82E-11 | 15,114 |
|  | MIMAT0000514 | miR-30c-5p | -2,440 | TarBase | Experimentally Observed | TNFAIP2 | 7,04E-06 | 3,119 |
|  | MIMAT0000514 | miR-30c-5p | -2,440 | TarBase | Experimentally Observed | WDR92 | 1,76E-03 | 2,913 |
|  | MIMAT0000565 | miR-328-3p | -1,730 | TargetScan Human | High predicted | ALDOC | 1,12E-08 | 4,417 |
|  | MIMAT0000565 | miR-328-3p | -1,730 | miRecords | Experimentally Observed | CD44 | 2,84E-06 | 3,549 |
|  | MIMAT0000766 | miR-335-5p | -2,960 | TargetScan Human | High predicted | PTPN22 | 1,66E-10 | 9,746 |
|  | MIMAT0000595 | miR-345-5p | -2,100 | TargetScan Human | High predicted | CCR5 | 6,08E-12 | 15,345 |
|  | MIMAT0000595 | miR-345-5p | -2,100 | TargetScan Human | High predicted | CLIC1 | 7,91E-08 | 3,758 |
|  | MIMAT0000595 | miR-345-5p | -2,100 | TargetScan Human | High predicted | PIM1 | 9,95E-04 | 2,008 |
|  | MIMAT0000595 | miR-345-5p | -2,100 | TargetScan Human | High predicted | TRAF1 | 8,23E-09 | 4,685 |
|  | MIMAT0001542 | miR-34a-5p | 25,380 | TargetScan Human | High predicted | BCL2L13 | 4,35E-06 | -2,275 |
|  | MIMAT0001542 | miR-34a-5p | 25,380 | TargetScan Human | High predicted | GFRA3 | 5,49E-05 | -2,087 |
|  | MIMAT0003130 | miR-486-5p | -3,410 | TargetScan Human | High predicted | PIM1 | 9,95E-04 | 2,008 |
|  | MIMAT0003482 | miR-499-5p | -2,590 | TargetScan Human | High predicted | CD38 | 2,98E-06 | 2,370 |
|  |  |  |  |  |  |  |  |  |
| **30 dpi mitochondrial genes** | **ID** | **Symbol** | **Expr Fold Change** | **Source** | **Confidence** | **Symbol** | **Expr p-value** | **Expr Fold Change** |
|  | MIMAT0000150 | miR-138-5p | -1,810 | TargetScan Human | High predicted | TSPO | 1,08E-09 | 3,399 |
|  | MIMAT0000435 | miR-143-3p | -2,070 | TargetScan Human | High predicted | IFIT3 | 1,74E-13 | 18,800 |
|  | MIMAT0000565 | miR-328-3p | -1,730 | TargetScan Human | High predicted | ALDOC | 1,12E-08 | 4,417 |
|  | MIMAT0000595 | miR-345-5p | -2,100 | TargetScan Human | High predicted | CLIC1 | 7,91E-08 | 3,758 |
|  | MIMAT0001542 | miR-34a-5p | 25,380 | TargetScan Human | High predicted | BCL2L13 | 4,35E-06 | -2,275 |
|  | MIMAT0000523 | let-7a-5p | 1,690 | TargetScan Human | High predicted | NME4 | 7,83E-07 | -2,410 |
|  | MIMAT0000387 | miR-130a-3p | 3,150 | TargetScan Human | High predicted | HADHA | 2,54E-05 | -2,292 |
|  | MIMAT0000150 | miR-138-5p | -1,810 | TargetScan Human | High predicted | GM2A | 3,97E-09 | 2,849 |
|  | MIMAT0000435 | miR-143-3p | -2,070 | TargetScan Human | High predicted | GATM | 2,90E-07 | 3,452 |
|  | MIMAT0000165 | miR-155-5p | 4,070 | TargetScan Human | High predicted | CARS2 | 1,12E-07 | -2,593 |
|  | MIMAT0000124 | miR-16-5p | 2,700 | miRecords | Experimentally Observed | HSDL2 | 1,11E-06 | -2,162 |
|  | MIMAT0000124 | miR-16-5p | 2,700 | TargetScan Human | High predicted | PDK4 | 1,18E-02 | -3,419 |
|  | MIMAT0003187 | miR-17-5p | 4,130 | TargetScan Human | High predicted | CAPRIN2 | 2,12E-07 | -2,329 |
|  | MIMAT0000214 | miR-185-5p | -1,590 | TargetScan Human | High predicted | GLRX | 2,02E-05 | 2,315 |
|  | MIMAT0000214 | miR-185-5p | -1,590 | TargetScan Human | High predicted | GPX1 | 4,75E-08 | 2,746 |
|  | MIMAT0000670 | miR-221-3p | 2,730 | TargetScan Human | High predicted | ADHFE1 | 3,85E-06 | -2,060 |
|  | MIMAT0000670 | miR-221-3p | 2,730 | TargetScan Human | High predicted | NDUFA1 | 4,83E-06 | -2,030 |
|  | MIMAT0000534 | miR-26a-5p | -1,530 | Ingenuity Expert Findings | Experimentally Observed | BAK1 | 9,42E-08 | 2,813 |
|  | MIMAT0000534 | miR-26a-5p | -1,530 | TargetScan Human | High predicted | BID | 2,65E-10 | 4,337 |
|  | MIMAT0000514 | miR-30c-5p | -2,440 | TargetScan Human | High predicted | RAB32 | 2,56E-11 | 8,267 |
|  | MIMAT0000565 | miR-328-3p | -1,730 | TargetScan Human | High predicted | GLRX | 2,02E-05 | 2,315 |
|  | MIMAT0000142 | miR-9-5p | -2,930 | TargetScan Human | High predicted | MTHFD2 | 3,89E-08 | 3,392 |
|  |  |  |  |  |  |  |  |  |
| **30 dpi**  **nrf2 genes** | **ID** | **Symbol** | **Expr Fold Change** | **Source** | **Confidence** | **Symbol** | **Expr p-value** | **Expr Fold Change** |
|  | MIMAT0000142 | miR-9-5p | -2,930 | TargetScan Human | High predicted | MTHFD2 | 3,89E-08 | 3,392 |
|  | MIMAT0000150 | miR-138-5p | -1,810 | TargetScan Human | High predicted | G6PD | 4,43E-11 | 3,454 |
|  | MIMAT0000145 | miR-133a-3p | -2,070 | TargetScan Human | High predicted | FTL | 3,99E-08 | 2,607 |
|  | MIMAT0000124 | miR-16-5p | 2,700 | TargetScan Human | High predicted | CHAC1 | 2,26E-02 | -2,216 |
|  | MIMAT0003187 | miR-17-5p | 4,130 | TargetScan Human | High predicted | VLDLR | 1,66E-07 | -2,259 |
|  | MIMAT0000211 | miR-182-5p | 34,780 | TargetScan Human | High predicted | VLDLR | 1,66E-07 | -2,259 |
|  |  |  |  |  |  |  |  |  |
|  |  |  |  |  |  |  |  |  |
| **45 dpi ifng induced genes** | **ID** | **Symbol** | **Expr Fold Change** | **Source** | **Confidence** | **Symbol** | **Expr p-value** | **Expr Fold Change** |
|  | MIMAT0000219 | miR-24-3p | -1,466 | TarBase,miRecords | Experimentally Observed | CDKN2A | 1,72E-06 | 2,728 |
|  | MIMAT0000538 | miR-31-5p | -5,458 | miRecords | Experimentally Observed | CDKN2A | 1,72E-06 | 2,728 |
|  | MIMAT0000247 | miR-143-3p | -1,795 | TargetScan Human | High predicted | IFIT3 | 2,69E-13 | 22,321 |
|  | MIMAT0000225 | miR-16-5p | -2,144 | TarBase | Experimentally Observed | HMOX1 | 1,90E-03 | 2,686 |
|  | MIMAT0000219 | miR-24-3p | -1,466 | TargetScan Human | High predicted | IRF1 | 3,32E-11 | 9,195 |
|  | MIMAT0004585 | let-7i-3p | -1,850 | TargetScan Human | High predicted | ACTB | 2,26E-06 | 2,434 |
|  | MIMAT0000157 | miR-145-5p | -1,853 | TargetScan Human | High predicted | ACTB | 2,26E-06 | 2,434 |
|  | MIMAT0000088 | miR-30a-3p | -1,940 | TargetScan Human | High predicted | ADA | 1,07E-04 | 2,200 |
|  | MIMAT0000161 | miR-151-3p | 1,840 | TargetScan Human | High predicted | AQP4 | 1,71E-04 | -2,379 |
|  | MIMAT0004620 | let-7a-3p | -2,116 | TargetScan Human | High predicted | BST1 | 3,88E-11 | 10,273 |
|  | MIMAT0000147 | miR-135a-5p | -2,321 | TargetScan Human | High predicted | CASP1 | 5,21E-10 | 7,856 |
|  | MIMAT0004627 | miR-20a-3p | -1,964 | TargetScan Human | High predicted | CCL4 | 1,95E-11 | 25,992 |
|  | MIMAT0000656 | miR-139-5p | -3,009 | TargetScan Human | High predicted | CTSC | 6,55E-09 | 4,038 |
|  | MIMAT0000237 | miR-204-5p | -3,355 | miRecords | Experimentally Observed | CTSC | 6,55E-09 | 4,038 |
|  | MIMAT0000147 | miR-135a-5p | -2,321 | TargetScan Human | High predicted | CXCL10 | 1,95E-11 | 62,955 |
|  | MIMAT0000148 | miR-136-5p | -6,400 | TargetScan Human | High predicted | FCGR2A | 1,03E-07 | 4,289 |
|  | MIMAT0000247 | miR-143-3p | -1,795 | TargetScan Human | High predicted | HLA-DOA | 1,30E-07 | 2,983 |
|  | MIMAT0000237 | miR-204-5p | -3,355 | TargetScan Human | High predicted | HLA-DRB5 | 1,34E-07 | 15,161 |
|  | MIMAT0000157 | miR-145-5p | -1,853 | TargetScan Human | High predicted | IFI30 | 7,95E-08 | 6,325 |
|  | MIMAT0000539 | miR-92a-3p | -1,330 | TargetScan Human | High predicted | IFIT2 | 2,93E-09 | 8,148 |
|  | MIMAT0000247 | miR-143-3p | -1,795 | TargetScan Human | High predicted | IL18 | 1,43E-05 | 2,423 |
|  | MIMAT0004946 | miR-744-3p | -3,040 | TargetScan Human | High predicted | IL18 | 1,43E-05 | 2,423 |
|  | MIMAT0000616 | miR-101b-3p | -1,407 | TargetScan Human | High predicted | NMI | 2,93E-09 | 2,872 |
|  | MIMAT0000148 | miR-136-5p | -6,400 | TargetScan Human | High predicted | NOS2 | 5,37E-03 | 2,337 |
|  | MIMAT0000247 | miR-143-3p | -1,795 | Ingenuity Expert Findings | Experimentally Observed | PRC1 | 4,40E-06 | 3,249 |
|  | MIMAT0000219 | miR-24-3p | -1,466 | TargetScan Human | High predicted | PSMB8 | 3,48E-14 | 17,652 |
|  | MIMAT0000237 | miR-204-5p | -3,355 | TargetScan Human | High predicted | PSME1 | 1,20E-08 | 2,610 |
|  | MIMAT0000225 | miR-16-5p | -2,144 | TarBase | Experimentally Observed | PTGS2 | 4,78E-04 | 2,063 |
|  | MIMAT0000534 | miR-26a-5p | -1,877 | Ingenuity Expert Findings,TargetScan Human | Experimentally Observed | PTGS2 | 4,78E-04 | 2,063 |
|  | MIMAT0000214 | miR-185-5p | -2,004 | TargetScan Human | High predicted | SDC3 | 4,90E-06 | 2,083 |
|  | MIMAT0000159 | miR-149-5p | -2,980 | TargetScan Human | High predicted | SRPX | 1,80E-04 | 2,130 |
|  | MIMAT0000225 | miR-16-5p | -2,144 | TargetScan Human | High predicted | SRPX | 1,80E-04 | 2,130 |
|  | MIMAT0004627 | miR-20a-3p | -1,964 | TargetScan Human | High predicted | TRAF1 | 2,02E-08 | 4,820 |
|  | MIMAT0000223 | miR-193a-3p | -7,877 | TargetScan Human | High predicted | UBE2L6 | 1,68E-09 | 5,253 |
|  | MIMAT0000219 | miR-24-3p | -1,466 | TargetScan Human | High predicted | USP18 | 8,98E-10 | 5,762 |
|  | MIMAT0000138 | miR-126a-3p | -1,562 | TarBase,miRecords | Experimentally Observed | VCAM1 | 1,22E-09 | 6,703 |
|  |  |  |  |  |  |  |  |  |
| **45 dpi mitochondrial genes** | **ID** | **Symbol** | **Expr Fold Change** | **Source** | **Confidence** | **Symbol** | **Expr p-value** | **Expr Fold Change** |
|  | MIMAT0000534 | miR-26a-5p | -1,877 | Ingenuity Expert Findings | Experimentally Observed | BAK1 | 3,89E-06 | 2,358 |
|  | MIMAT0000534 | miR-26a-5p | -1,877 | TargetScan Human | High predicted | BID | 2,98E-09 | 3,850 |
|  | MIMAT0000219 | miR-24-3p | -1,466 | TarBase,miRecords | Experimentally Observed | CDKN2A | 1,72E-06 | 2,728 |
|  | MIMAT0000538 | miR-31-5p | -5,458 | miRecords | Experimentally Observed | CDKN2A | 1,72E-06 | 2,728 |
|  | MIMAT0000247 | miR-143-3p | -1,795 | TargetScan Human | High predicted | GATM | 8,51E-07 | 3,465 |
|  | MIMAT0000647 | miR-103-3p | 20,370 | TargetScan Human | High predicted | HMGCS2 | 5,44E-03 | -3,794 |
|  | MIMAT0000247 | miR-143-3p | -1,795 | TargetScan Human | High predicted | IFIT3 | 2,69E-13 | 22,321 |
|  | MIMAT0004627 | miR-20a-3p | -1,964 | TargetScan Human | High predicted | RAD51 | 3,16E-06 | 2,154 |
|  | MIMAT0000225 | miR-16-5p | -2,144 | Ingenuity Expert Findings,TargetScan Human | Experimentally Observed | UCP2 | 4,59E-03 | 2,120 |
|  |  |  |  |  |  |  |  |  |
| **45 dpi**  **nrf2 genes** | **ID** | **Symbol** | **Expr Fold Change** | **Source** | **Confidence** | **Symbol** | **Expr p-value** | **Expr Fold Change** |
|  | MIMAT0000225 | miR-16-5p | -2,144 | TarBase | Experimentally Observed | HMOX1 | 1,90E-03 | 2,686 |
|  | MIMAT0000225 | miR-16-5p | -2,144 | TarBase,TargetScan Human | Experimentally Observed | PSAT1 | 2,51E-05 | 2,444 |
|  | MIMAT0003187 | miR-17-5p | 3,159 | TargetScan Human | High predicted | VLDLR | 2,66E-06 | -2,061 |
|  | MIMAT0000211 | miR-182-5p | 12,990 | TargetScan Human | High predicted | VLDLR | 2,66E-06 | -2,061 |
